# Synthesis, characterization, and biomedical evaluation of ethylene-bridged tetra-NHC Pd(ii), Pt(ii) and Au(iii) complexes, with apoptosis-inducing properties in cisplatin-resistant neuroblastoma cells†

**DOI:** 10.1039/d4ra01195c

**Published:** 2024-03-27

**Authors:** Wolfgang R. E. Büchele, Tim P. Schlachta, Andreas L. Gebendorfer, Jenny Pamperin, Leon F. Richter, Michael J. Sauer, Aram Prokop, Fritz E. Kühn

**Affiliations:** a Technical University of Munich, School of Natural Sciences, Department of Chemistry and Catalysis Research Center, Molecular Catalysis Lichtenbergstraße 4 85748 Garching Germany fritz.kuehn@ch.tum.de +49 89 289 13477; b Department of Pediatric Hematology/Oncology, Children's Hospital Cologne Amsterdamer Straße 59 50735 Cologne Germany; c Department of Pediatric Oncology/Hematology, Helios Clinics Schwerin Wismarsche Straße 393-397 19055 Schwerin Germany aram.prokop@helios-gesundheit.de; d Department of Human Medicine, MSH Medical School Hamburg Am Kaiserkai 1 20457 Hamburg Germany

## Abstract

Synthesis and characterization of the first two cyclic ethylene-bridged tetradentate NHC ligands, with an unsaturated (imidazole) and saturated backbone (2-imidazoline), are described. Complexes of both ligands containing palladium(ii) have been obtained. For platinum(ii) and gold(iii), only the unsaturated tetracarbene complexes could be isolated. The attempts to synthesize a methylene-bridged 2-imidazoline macrocycle are also described. Furthermore, a novel bisimidazolinium ligand precursor and its open-chain Pd^II^ and Pt^II^ tetracarbene complexes are obtained. Finally, it is shown that the unsaturated gold(iii) tetracarbene is able to induce apoptosis in malignant SK-N-AS neuroblastoma cells *via* the mitochondrial and ROS pathway and overcomes resistance to cisplatin *in vitro*.

## Abstract 2

New ethylene-bridged N-heterocyclic carbene ligand systems and their respective transition metal complexes are characterized and the electronic properties are discussed.

## Graphical abstract

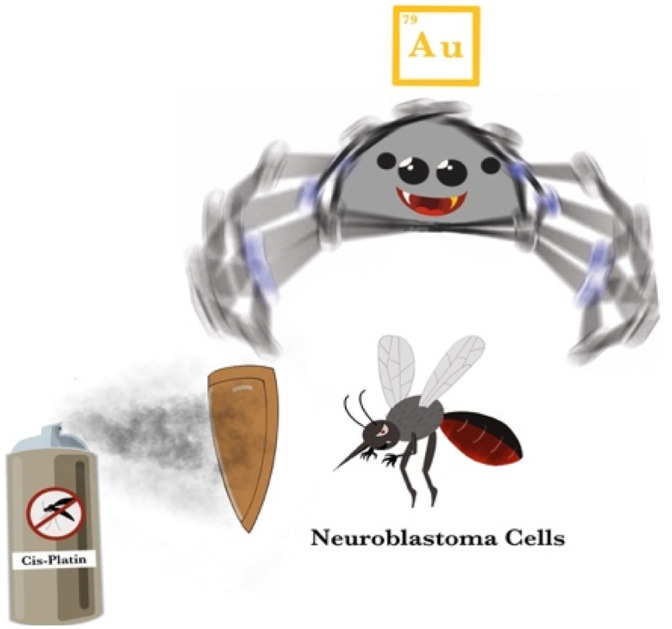

## Introduction

N-heterocyclic carbenes (NHCs), first described in 1991,^1^ have found many applications.^2^ There are several structural features that allow the tuning of their electronic properties. Ring size, the adjacent heteroatoms, *N*-substituents, and the backbone can be modified. Changing one or more structural properties of a NHC ligand can lead to significantly different reactivities and stabilities of the resulting complexes.^3^ Often several NHC units are combined in multidentate ligands, making use of the chelating effect, and a plethora of multidentate NHC metal complexes has been reported.^4,5^

**Fig. 1:**
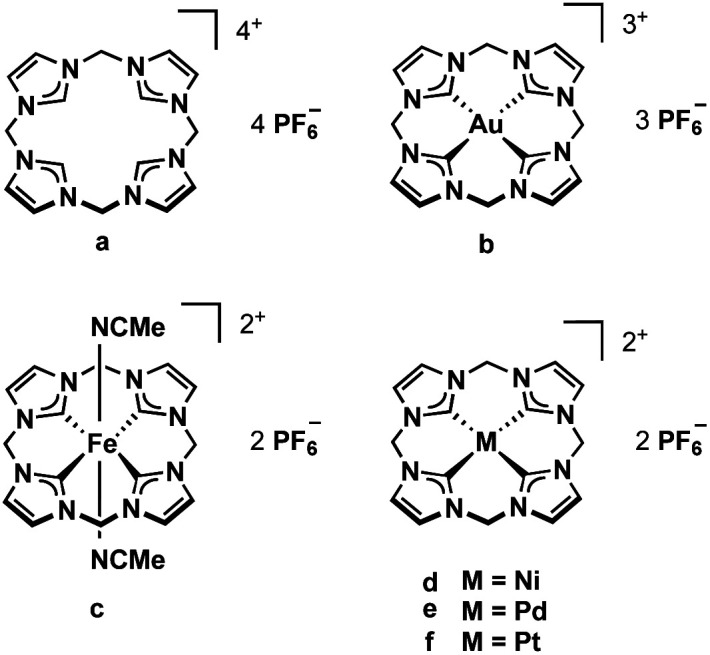
Tetracarbene ligand precursor a and derived transition metal complexes b–f.

Our group has developed several bidentate and cyclic tetradentate NHC ligands. The respective transition metal complexes have been applied *e.g.* in medicinal chemistry^6,7^ and epoxidation catalysis.^3^ While the bidentate ligands can form open-chain tetracarbene complexes,^8,9^ the tetradentate ligands give cyclic tetracarbene compounds. Most commonly applied in our recent examinations is the calix[4]imidazolium ligand precursor (a, Fig. 1).^10^ Its iron complex (c) can be used as olefin epoxidation catalyst achieving unprecedented activity.^3^ Coinage metal tetracarbene complexes (and metal NHC complexes in general^11–17^) have been investigated regarding their antiproliferative activity and selectivity against cancer cells (b, d–f, Fig. 1).^6,7^

In this study, the scope of multidentate NHC ligands is extended with an ethylene-bridged bisimidazolinium ligand precursor and two cyclic ethylene-bridged tetradentate NHC ligands, with an unsaturated (imidazole) and saturated backbone (2-imidazoline). Pd^II^, Pt^II^ and Au^III^ tetracarbene complexes containing the novel ligands are synthesized, characterized and applied in preliminary medicinal studies regarding their activity in inducing apoptosis in malignant cells. Finally, the synthetic attempts to a calix[4]imidazolinium macrocycle (structurally analog to c but with a saturated backbone) are described, because there is an increasing demand for reliable training data, including data on negative outcomes, for machine learning systems in chemistry.^18^

Especially the two new macrocyclic ligand precursors are intended to lay the foundation for electronic comparisons induced by the different backbone in future studies. The unsaturated backbone of the imidazole moiety causes partial aromaticity, increasing NHC stability by *ca.* 100 kJ mol^−1^.^19–21^ A saturated backbone, in turn, can lead to higher basicity because the electron density is more concentrated on the C2 carbene carbon atom due to the lack of π-interactions.^22^

## Results and discussion

The synthesis of a saturated macrocyclic ligand precursor similar to c, but containing 2-imidazoline moieties instead of imidazole, calix[4]imidazolinium, was pursued parallel to the synthesis of the other ligand precursors. However, the synthesis was not successful with the chosen synthetic approaches as described in the ESI.†

### Synthesis and characterization of H_2_L3

**Fig. 2:**
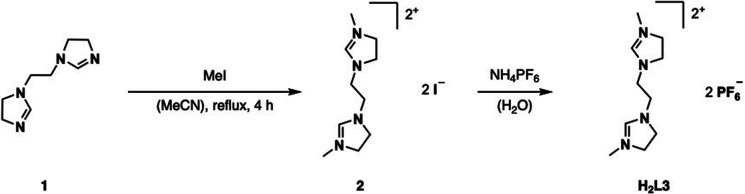
Synthesis of ligand precursor H_2_L3*via* alkylation and anion exchange.

H_2_L3 is based on the literature known ethylene-bridged imidazoline moiety (1).^23^ Alkylation of 1 with MeI in MeCN at 82 °C, followed by an anion exchange with NH_4_PF_6_ in water, gives H_2_L3 in 91% yield (Fig. 2).

### Synthesis and characterization of H_4_L5/6 and H_4_L8/L9

**Fig. 3:**
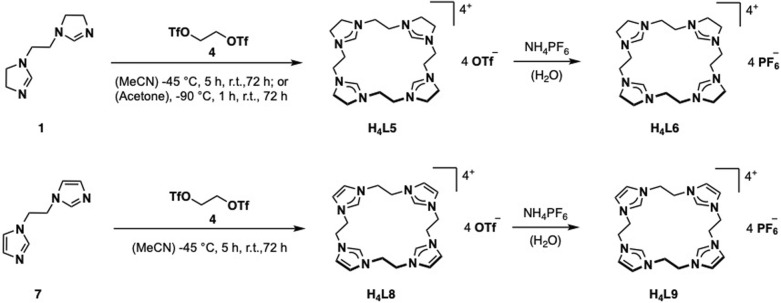
Synthesis of ligand precursors H_4_L5/6 and H_4_L8/9*via* ring closure with ethylene bistriflate and anion exchange.

For the preparation of H_4_L5/6 and H_4_L8/9, a slightly modified literature procedure for similar macrocycles was used (Fig. 3).^10^ Ring closure to form the macrocyclic imidazolium salt a is commonly achieved with CH_2_(OTf)_2_,^3^ but also CH_2_Br_2_ is reported.^24^ Here the ethylene-bridged imidazoline 1 (ref. 23) and the ethylene-bridged imidazole 7 (ref. 25) are reacted with ethylene bistriflate (4) under dry conditions at −45 °C over a period of 5 h in dry MeCN for H_4_L5 and H_4_L8 (see ESI†).

The synthesis of H_4_L5 and H_4_L8 yields a mixture of a 20-membered macrocycle (87% H_4_L5 and 90% H_4_L8, as determined by NMR), consisting of four imidazole (C[4]) units, and a 30-membered macrocycle (13% H_4_L5, 10% H_4_L8) consisting of six imidazole (C[6]) units (see ESI†). Separation attempts of C[4] and C[6] *via* column chromatography, precipitation or sublimation were not successful. However, by increasing the cooling period during the addition of the ethylene bistriflate at −45 °C to a total of 5 h, the purity of the kinetically preferred C[4] unit could be easily increased up to 98% C[4] for H_4_L5 and in case of H_4_L8 an increase up to 100% (ESI†). Due to the absence of similar macrocyclic imidazolinium compounds, H_4_L5 is compared to a and H_4_L8 in the following.^3^

Relative to a, all signals of H_4_L5 and H_4_L8 are upfield shifted, indicating a higher electronic density due to the +I effect of the ethylene bridge leading to an increased shielding effect in the NMR.^10,26^ The higher upfield shift of H_4_L5 compared to H_4_L8 can be explained by the electronic inducing effect of the saturated bond.^20,26,27^

Unlike in ^1^H-NMR, each individual ^13^C signal of H_4_L8 in DMSO-d_6_ is in the same range as the signals obtained for the macrocyclic compound a.^10^ However, in case of H_4_L5, opposite to the ^1^H-NMR, the C2 carbon resonance at 159.16 ppm is downfield shifted compared to H_4_L8 and a (H_4_L8, Δ*δ* ≤ 22.08 ppm, a, Δ*δ* ≤ 22.0 ppm), thus contradicting expectations. According to literature and as described by H. V. Huynh, the hypothetical free carbene of the imidazoline ligand H_4_L5 should be a stronger σ-donor than H_4_L8, so an enhanced upfield shift of the C2 signal of H_4_L5 should have been detectable.^20,28,29^ Interestingly this expectation is not met here, and apparently other factors play a role. Every other resonance in the ^13^C-NMR is upfield shifted.^10^

Salt metathesis of the formed macrocyclic salts can be performed with NH_4_PF_6_ to increase the solubility in organic solvents and as additional purification step.^3,30^ Thus, an anion exchange in water towards PF_6_^−^ is conducted with H_4_L5 and H_4_L8, resulting in H_4_L6 (81%) and H_4_L9 (88%).

### Synthesis and characterization of complexes (Pd/PtL3, PdL5/6, Pd/PtL8, Pd/AuL9)

**Fig. 4:**
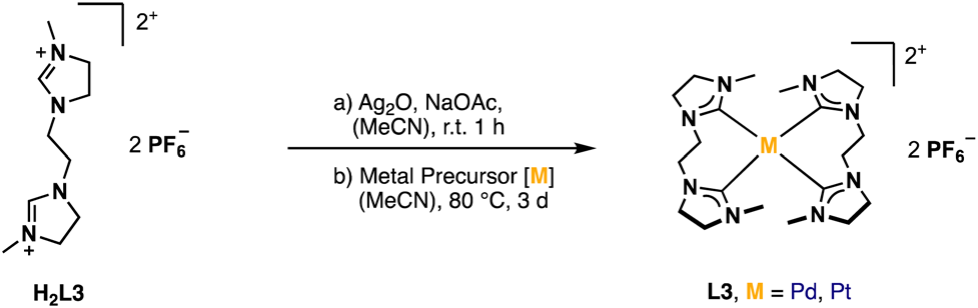
General synthesis of Pd/PtL3.

A well-established route to obtain NHC complexes is to convert the corresponding imidazolium salts with group 10 metal acetates. In this reaction, the acetate serves as an internal base capable of deprotonating imidazolium- and imidazolinium salts to form NHCs, which subsequently coordinate to the metal.^6,31–33^ An alternative route is *via* a silver transmetalation.^34^ In the first approaches, attempts were made to synthesize the respective Ag^I^ complex with H_2_L3 to obtain a dinuclear structure similar to already published open chain bis-NHC-complexes.^9,25,35^ However, no product formation was observed in our case. Either no reaction took place or complex signals were observed in the aliphatic region of *δ* = 1.9–4.4 ppm in the ^1^H-NMR after purification, indicating the decomposition of H_2_L3. Several other conditions with different Ag^I^-salts and addition of sodium acetate as internal base at different temperatures were tested without success. A possible problem might be the stability of the Ag^I^-complex. Another issue might be hydrolysis of imidazolines under acidic and basic conditions.^36,37^ It has been proposed in literature that the moisture in the solvent can react with sodium acetate to generate hydroxide ions which can attack the electrophilic center of the C2 carbon and lead to ring-opening products, rather than nucleophilic attacking the acidic proton at the C2 carbon.^38^ Therefore, the next attempts were conducted under moisture-free reaction conditions by using dried solvents. Even the direct metalation with palladium(ii) acetate or palladium(ii) chloride under dry reaction conditions did not lead to the desired product. The focus was then shifted to a combination of the transmetalation route using Ag_2_O *in situ* with the direct metalation, by applying the respective metal precursor and sodium acetate as a mild base in dry solvents (Fig. 4).

The absence of the acidic imidazolinium proton signal and appearance of characteristic carbene carbon signals confirms the successful formation of PdL3 and PtL3. Unfortunately, despite several attempts, a clean elemental analysis for PtL3 could not be obtained. Also, the ^1^H-NMR of PtL3 shows some impurities, which could not be identified and no ^195^Pt isotope coupling phenomena was observed in the ^13^C-NMR.

**Fig. 5:**
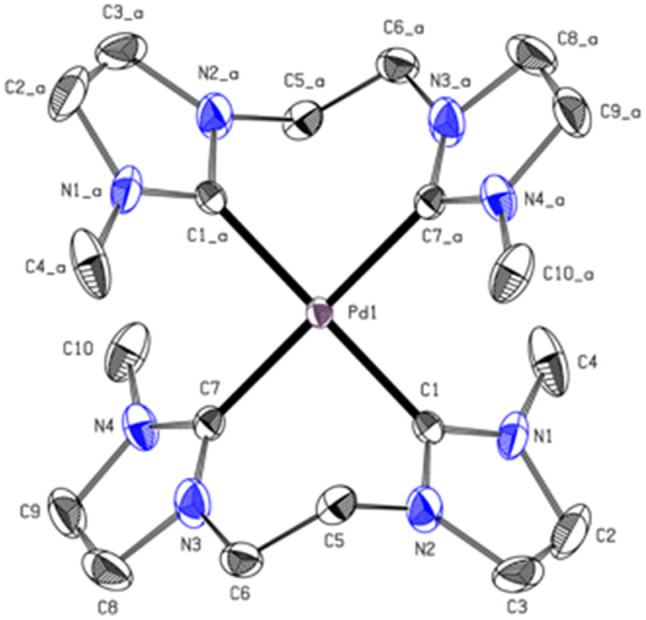
ORTEP-style representation of the cationic fragment of complex PdL3. Hydrogen atoms and hexafluorophosphate anions are omitted for clarity. Thermal ellipsoids are shown at a 50% probability level. Selected bond lengths (Å) and angles (°): C1–Pd1 2.039(2); C7–Pd1 2.038(2); C7_a–Pd1–C7 180.0; C7–Pd1–C1_a 91.64(9); C7–Pd1–C1 88.36(9), C7_a–Pd1–C1_a–N2_a 55.30.

The carbene carbon signal of PdL3 at 194.29 ppm in DMSO-d_6_ [PtL3; 188.38 ppm in CD_3_CN], is surprisingly downfield shifted compared to other Pd(ii) bis-NHCs reported in literature.^39,40^ Due to the theoretically stronger σ-donation of the imidazolinylidene ligand H_2_L3 compared to its unsaturated analog, an upfield shift of the ^13^C_NHC_ signal was expected. Literature indicates that the significant downfield shift of the carbene carbon resonance from imidazolium to imidazolinium compounds is a general phenomenon.^38,41,42^ Another interesting fact is that the analytic data, including HR-ESI-MS and elemental analysis, are not supporting a dinuclear complex or a mono-carbene complex as expected, but indicate that PdL3 has rather a [Pd(L3)_2_](PF_6_)_2_ structure similar to e. This is further confirmed by single-crystal X-ray diffraction (SC-XRD). The PdL3 complex displays a distorted square planar structure. Two L3 ligands coordinate to the Pd center, resulting in an open-chain tetracarbene complex of similar geometry like the cyclic complex e.^34^ The Pd–C (2.039 Å, 2.038 Å) distances are in good accord with palladium(ii) NHC complexes reported in literature.^34,39^ The alkyl groups of the ligand L3 adopt a *syn* conformation in the solid state, while the imidazole rings are tilted by 55.30° out of the palladium square plane (Fig. 5).

**Fig. 6:**
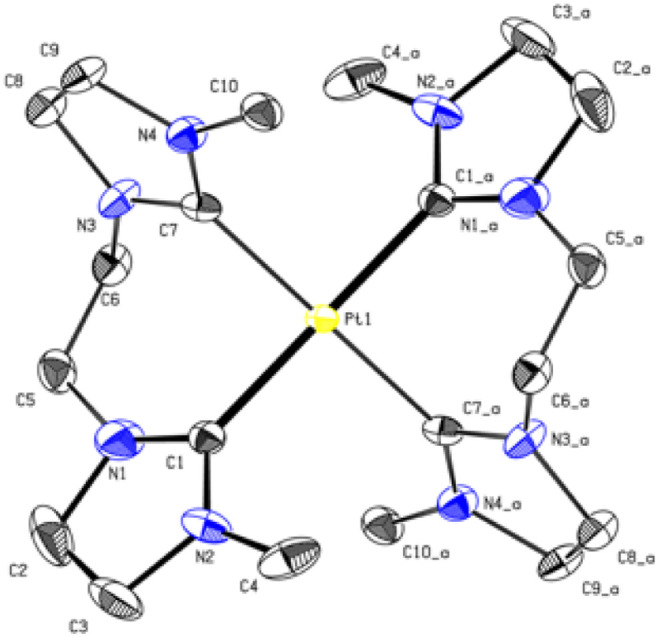
ORTEP-style representation of the cationic fragment of complex PtL3. Hydrogen atoms and hexafluorophosphate anions are omitted for clarity. Thermal ellipsoids are shown at a 50% probability level. Selected bond lengths (Å) and angles (°): Pt1–C1 2.0337 (18); Pt1–C7 2.039 (6), C1_a–Pt1–C1 180.00(7); C1–Pt1–C7_a 91.2(5); C1–Pt1–C7 88.8(5), C7_a–Pd1–C1_a–N2_a 50.53°.

The PtL3 complex exhibits a similarly distorted square planar structure compared to PdL3. The Pt–C (2.033 Å, 2.039 Å) distances are comparable to similar literature known group 10 NHC compounds.^43–47^ The alkyl groups of L3 also adopt a *syn* conformation, while the imidazole rings are tilted by 50.53° out of the palladium square plane as in PdL3 (Fig. 6).

### Complex PdL5/6 and PdL8/9

**Fig. 7:**
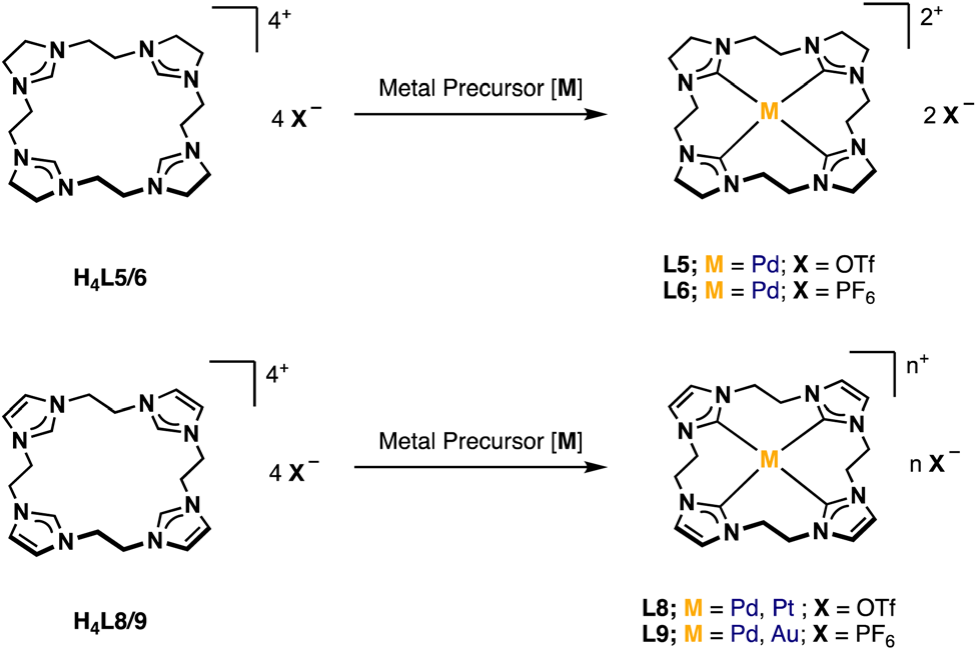
General synthesis of PdL5/6, Pd/PtL8 and Pd/AuL9.

Since H_4_L5 and H_4_L8 are quite similar to other macrocycles (a), it seemed suitable to synthesize PdL5 and PdL8 according to alike compounds *via* the direct metalation route.^32^ Therefore, H_4_L8 was first converted with Pd(OAc)_2_ in a mixture of dry DMSO/MeCN (1 : 1) at 40 °C for 16 h.^34^ However, no product formation was observed after work-up. Several other conditions such as increasing temperature and reaction time led to the absence of the imidazolium protons and the formation of new product signals in the ^1^H-NMR after 4 d at 80 °C. Still these intensities were very low, and no product could be isolated. Another approach was tried *via* the transmetalation route with Ag^I^ salts, but this also led to no product formation. Finally, both PdL8 (50%, Fig. 7) and PdL5 (3%) could be obtained by applying the same reaction conditions as for the already synthesized complexes PdL3 and PtL3. The yield of the imidazolinylidene tetracarbene complex could be increased to 46%, by using H_4_L6 instead of H_4_L5, resulting in PdL6.

**Fig. 8:**
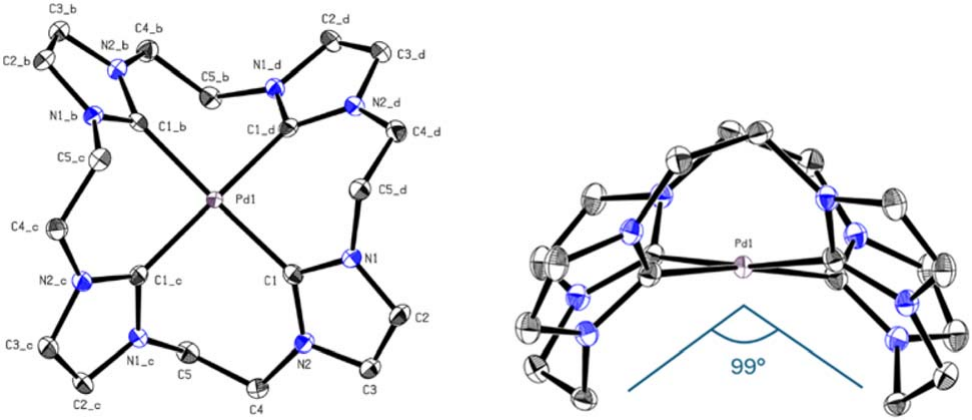
ORTEP-style representation of the cationic fragment of complex PdL9. Hydrogen atoms and hexafluorophosphate anions are omitted for clarity. Thermal ellipsoids are shown at a 50% probability level. Selected bond lengths (Å) and angles (°): C1–Pd 2.019(2), C1–Pd1–C1_b 172.03(12), C1–Pd1–C1_d–N2_d 53.62, C5–Pd1–C5_b 98.97°.

Again, the absence of the acidic position 2 proton signals in the ^1^H-NMR and appearance of the carbene carbon peaks confirm the formation of Pd(ii) carbene complexes. The observed chemical shift of PdL8 is in the typical range of Pd(ii) tetra-NHC compounds and indicates the formation of a complex with similar coordination sphere as e.^6,34,45^ The ^1^H-NMR of PdL5 in CD_3_CN shows three signals, with two of them in a similar range to PdL8 and one upfield shifted signal of the backbone protons. As already mentioned in the discussion of H_4_L5, the ^13^C-NMR of PdL5 is contrary to expectations. The carbene carbon of PdL5 (191.30 ppm) is surprisingly strong downfield shifted compared to PdL8 (165.84 ppm) and in a similar range to the carbene carbon of PdL3 (195.6 ppm in CD_3_CN). Literature indicates that the significant downfield shift of the carbene carbon resonance from imidazole to imidazoline compounds is a general phenomenon.^38,41,42^ The uncertainty of a ^13^C-NMR measurement is expected to be below 0.1 ppm; by using three times the weighted standard deviation, a difference of >0.4 ppm is required for a significant difference that exceeds the statistic uncertainty.^3,48^ Therefore, PdL5 (191.30 ppm in CD_3_CN) and PdL8 (165.84 ppm in CD_3_CN) show a sufficiently different chemical shift to allow its discussion. In general, the normal NHC unit (without any modification) of the tetracarbene ligands is in a range of rather low to negligible π-backdonation, hence the changes in electronic properties are dominated by the σ-donation of the tetracarbenes.^3,29^ According to literature, the imidazoline ligand L5 should be in general a stronger σ-donor than L8, so an enhanced upfield shift of the carbene signal would have been detectable.^3,20,28,29^ Interestingly, this expectation is also not met here, and apparently other factors may play a role, as already observed with PdL3 and PtL3. Therefore, further investigations on this subject, *e.g.* by means of DFT calculations, have to be carried out, since only conjectures can be made with the present analytical data. The elemental analysis and HR-ESI-MS for PdL8 are in accord with a composition [Pd(L8)](OTf)_2_ similar to e. It needs to be noted that no clean elemental analysis of PdL6 could be obtained. However, the elemental analysis and HR-ESI-MS of PdL6 are in accordance with the composition [PdL6](PF_6_)_2_. Due to unsatisfying results in crystallization of PdL8, an anion exchange in water towards PF_6_^−^ was conducted, resulting in PdL9 (41%). Single crystals suitable for SC-XRD were obtained by slow diffusion of Et_2_O into MeCN solution of PdL9. As expected, the Pd(ii) ion is coordinated in a nearly square planar fashion with C–Pd–C angles deviating from 180° by ∼8°, thus lifting the metal slightly above the carbene carbon atom plane (Fig. 8). However, due to the C_2_-bridge, the ligand is strongly bent (C5–Pd1–C5_b = 98.97°) and adopts a crisp-shape, while tilting the imidazole rings 53.62° in an alternating pattern out of the palladium square plane.^49^ The Pd–C distance (2.019 Å) is comparable to those of other cyclic Pd(ii) tetracarbene compounds reported in literature.^6,34,43,45–47^

In the following Table 1 the M–C_carbene_ bond lengths [Å], the C_carbene_–M–C_carbene_ angle [°], the tilt of the NCN unit [°] of the complexes Pd/PtL3 and PdL9 and additionally the C_bridge_–M–C_bridge_ angle [°] for PdL9 are summarized.

**Table 1:** Summary of the M–C_carbene_ bond lengths [Å], the C_carbene_–M–C_carbene_ angle [°], the tilt of the NCN unit [°] of the complexes Pd/PtL3 and PdL9 and the C_bridge_–M–C_bridge_ angle [°] for PdL9

Compound	PdL3	PtL3	PdL9
M–C_carbene_ [Å]	2.038|2.039	2.033|2.039	2.019
C_carbene_–M–C_carbene_ [°]	180	180	172.03
Tilt NCN unit [°]	55.30	50.53	53.62
C_bridge_–M–C_bridge_ [°]	—	—	98.97

### Complex PtL8

Applying the same reaction conditions and work-up methods to Pt(MeCN)_2_Cl_2_ instead of Pd(OAc)_2_ results in the formation of PtL8 (25%,Fig. 7). The absence of acidic proton signals in the ^1^H-NMR and the appearance of the carbene ^13^C-peak at 159.39 ppm in CD_3_CN confirms the formation of the respective Pt(ii) complex. The chemical shift of the carbene carbon is in accordance with Pt(ii) tetra-NHC complexes previously reported in literature and is slightly shifted to the upfield compared to PdL8 (^13^C_NHC_ in CD_3_CN at 165.84 ppm) by 6.45 ppm.^43,46,50^ No ^195^Pt isotope coupling was observed. The ^1^H-NMR in CD_3_CN shows similar signals compared to PdL8, where the bridge protons also split into two multiplets at 5.01 and 4.44 ppm. In addition, HR-ESI-MS is in accordance to a similar composition as PdL8. Despite multiple attempts, no single crystals suitable for SC-XRD were obtained. However, the discussed analytical data strongly support a similar structure compared to PdL8 and similarly structured tetracarbene ligand.^6,34^

### Complex AuL9

For the synthesis of AuL9 (Fig. 7), the same reaction conditions were applied as reported in the literature for similar complexes.^51^ Therefore, H_4_L8 was converted with KAuCl_4_ and NaOAc in dry DMSO under exclusion of light at 100 °C for 5 h. After the work-up, including an ion exchange to PF_6_^−^ as a purification step, AuL9 (47%) was obtained. The absence of acidic proton signals in the ^1^H-NMR and the appearance of a new ^13^C-peak at 146.03 ppm in CD_3_CN confirm the formation of the respective Au(iii) complex. The chemical shift of the carbene carbon is in accord with Au(iii) tetracarbene complex (e) previously reported in literature and slightly downfield shifted by 1.79 ppm when compared to e.^51^ Furthermore, the backbone carbons are also slightly downfield shifted by 0.68 ppm. The ^1^H-NMR in CD_3_CN shows similar signals compared to complex PdL8 and PtL8 with the backbone protons at 7.47 ppm and the bridge protons as two multiplets in close proximity at 4.83 and 4.71 ppm. Both elemental analysis^52^ and HR-ESI-MS are in agreement with the composition [Au(L15)](PF_6_)_3_. Although no single crystals suitable for SC-XRD were obtained, the discussed analytical data strongly support the coordination of one tetracarbene ligand similar to PdL9.

## Biological evaluation

### Induction of apoptosis as cell death type

**Fig. 9:**
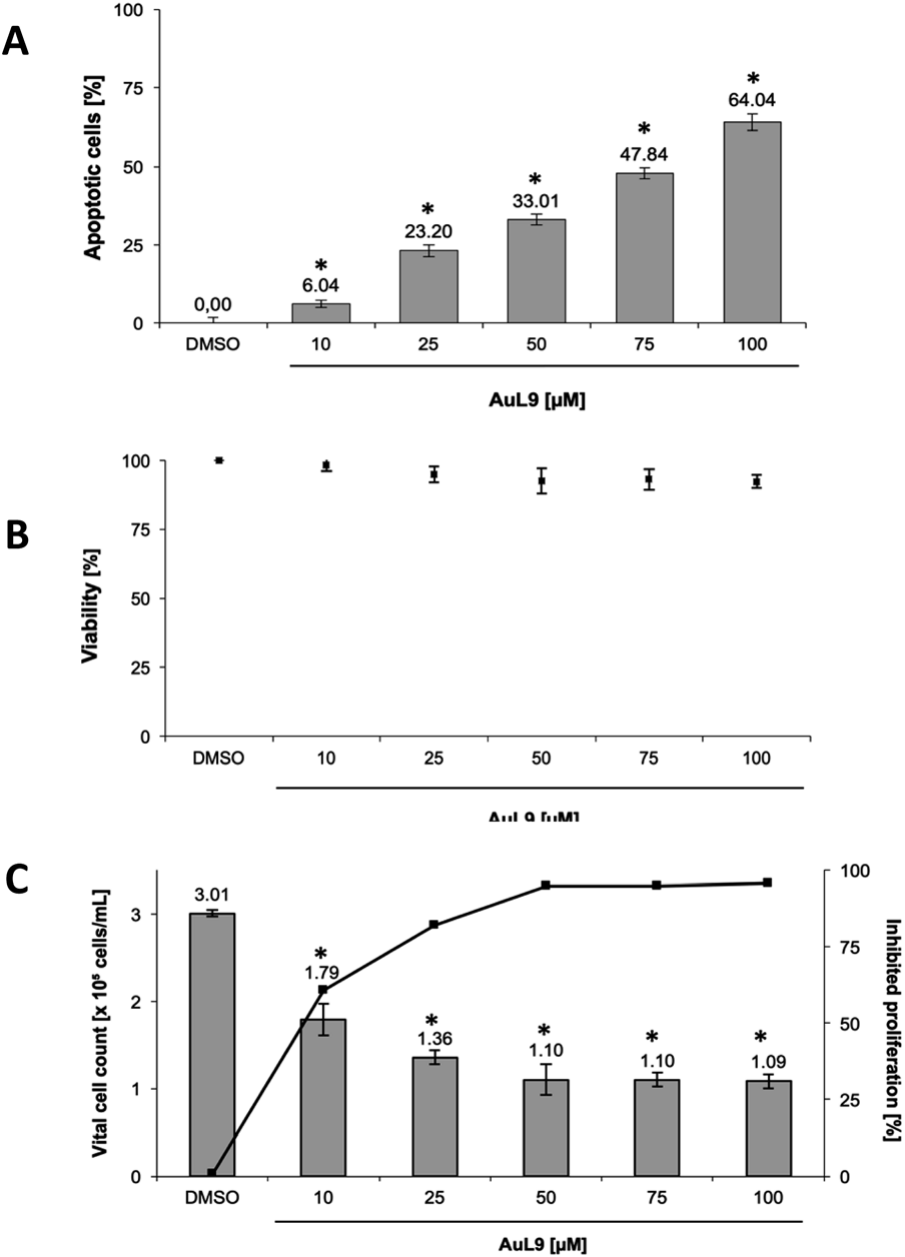
(A) AuL9 induces apoptosis in SK-N-AS cells. The cells were treated with different concentrations of AuL9 and incubated for 96 h. Nuclear DNA fragmentation was analyzed. (B) To exclude unspecific cytotoxic effects, such as necrotic cell death, the viability of SK-N-AS cells was determined by measurement of LDH release into the medium after 2 h of incubation with different concentrations of AuL9. No significant LDH release could be detected in cells treated with AuL9 up to a concentration of 100 μM. Values are given as mean% of DMSO control ± SD (*n* = 3). (C) The inhibition of proliferation of AuL9 treated SK-N-AS cells was measured after 48 h using the CASY Cell-Counter System. A significant inhibition of cell growth was observed at concentrations as low as 10 μM. Inhibition of proliferation is given in mean% of control ± SD (*n* = 3); *: *p* < 0.05 *vs.* DMSO, *t*-test.

PdL3, PdL8, AuL9 and their respective protonated ligand precursors were tested for their apoptotic effects on Nalm-6 cells (human B cell precursor leukemia cell line) and SK-N-AS cells (human neuroblastoma cell line) at different concentrations and quantified by the nuclear DNA fragmentation by flow cytometry analysis. PdL3 and PdL8 as well as the ligand precursors do not show any apoptosis inducing effects in Nalm-6 cells and SK-N-AS cells (see ESI†). AuL9 shows no apoptotic effect in Nalm-6 cells, but significant apoptosis induction by AuL9 is detected in SK-N-AS cells (Fig. 9A); therefore, the effect of AuL9 in SK-N-AS cells was further characterized.

To exclude necrotic effects of AuL9, lactate dehydrogenase (LDH) leakage from SK-N-AS cells after 2 h incubation with AuL9 was measured. LDH is released from the cell in case of necrosis and can be detected in the cell culture medium in case of loss of cell integrity and thus serves as a necrosis indicator.^53^AuL9 shows no significant non-specific cytotoxic effects on SK-N-AS cells in the relevant concentration range up to 100 μM (Fig. 9B).

In addition to apoptosis induction, it was tested whether AuL9 can inhibit the proliferation of malignant cells. For this purpose, SK-N-AS cells were incubated with different concentrations of AuL9 for 48 hours. The proliferation inhibition was determined by comparing the total cell number of vital cells of the DMSO control with the total cell number of vital cells of the treated cells. The results show that AuL9 inhibits cell proliferation of SK-N-AS cells in a dose-dependent manner (Fig. 9C). A concentration of 50 μM AuL9 causes nearly 100% inhibition of proliferation, indicating G1 arrest.

**Fig. 10:**
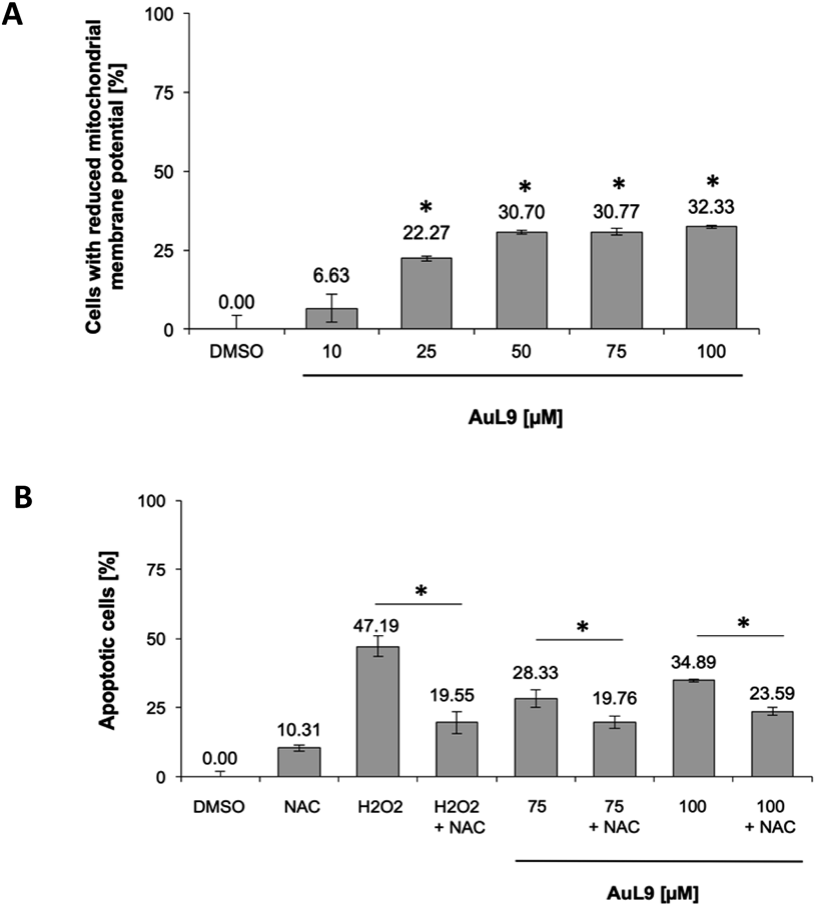
(A) The mitochondrial membrane potential in SK-N-AS cells was impaired by AuL15 treatment, which implicates mitochondrial pathway involvement in apoptosis induction. The mitochondrial membrane potential was measured by flow cytometric analysis in SK-N-AS cells after 48 h of incubation with different concentrations of AuL15 and staining with the cationic dye JC-1. Values are mean% of cells with low mitochondrial membrane potential ± SD (*n* = 3); *: *p* < 0.05 *vs.* DMSO, *t*-test. B The induction of apoptosis in SK-N-AS cells in response to AuL15 treatment was shown to be dependent on the ROS mediated pathway. The cells were incubated for 72 h with 50 μM H_2_O_2_ as a positive control or different concentrations of AuL15 with or without pretreatment of the cells with the ROS inhibitor *N*-acetylcysteine (NAC, 5 mM) 1.5 h prior to substance addition. Nuclear DNA fragmentation was analyzed by flow cytometric analysis. Values are mean% of apoptotic cells ± SD (*n* = 3); *: *p* < 0.05 *vs.* DMSO, *t*-test.

For the investigation of the mechanism of action of AuL9, the mitochondrial membrane potential of SK-N-AS cells was measured after 48 h incubation with AuL9. It was shown that the mitochondrion and thus the intrinsic apoptosis pathway plays at least a partial role in the effect of AuL9 (Fig. 10A).

To further characterize the role of mitochondria in AuL9-induced apoptosis, the apoptosis pathway mediated by reactive oxygen species (ROS) was investigated. Therefore, *N*-acetylcysteine (NAC) as a known ROS inhibitor and H_2_O_2_, which belongs to the ROS, as a positive control was investigated. It was shown that apoptosis induction could be significantly inhibited by NAC. It can therefore be concluded that the generation of ROS plays a role in the AuL9-induced apoptosis (Fig. 10B). However, it is not possible in the present state to be sure how the ROS are generated and whether AuL9 directly leads to an increased ROS production or triggers pathways that result in the generation of ROS.

### Overcoming cisplatin resistance

**Fig. 11:**
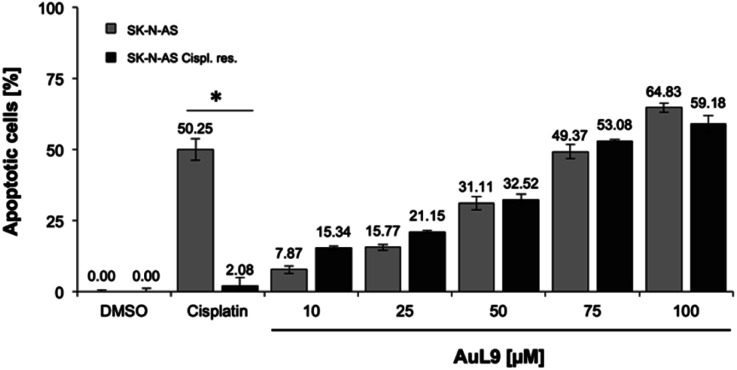
SK-N-AS and SK-N-AS cisplatin resistant cells were treated with different concentrations of AuL15 and incubated for 96 h. It is shown that AuL9 was also effective in inducing apoptosis in cisplatin resistant cells, thus overcoming resistance. 8.25 μM cisplatin has been used as a positive control to prove resistance. Nuclear DNA fragmentation was analyzed by flow cytometric analysis. Values are mean% of apoptotic cells ± SD (*n* = 3); *: *p* < 0.05 *vs.* DMSO, *t*-test.

Cisplatin is a well-known chemotherapeutic agent for the treatment of many different types of cancer.^54^ The development of resistance in tumor cells is a major problem in therapy and is usually the limiting factor in the cure of cancer patients.^55^ Therefore, it is of great importance for drug development that new agents are able to overcome cytostatic drug resistance. In addition to SK-N-AS cells, AuL9 was tested on cisplatin resistant SK-N-AS cells and cisplatin resistance overcoming could be demonstrated (Fig. 11). In a previous characterization of the cisplatin resistant SK-N-AS cells, procaspase-8 under expression was shown.^56^ The cisplatin resistance overcoming of SK-N-AS cells indicates that procaspase-8 has a minor role in AuL9-induced apoptosis.

## Conclusion and outlook

A synthetic approach to a calix[4]imidazolinium macrocycle as saturated analog to a is presented. The synthesis of two new macrocyclic ligand systems, being bridged by ethylene groups and containing imidazoline (H_4_L5/6) and imidazole moieties (H_4_L8/9) are discussed. In addition, a novel bisimidazolinium ligand precursor (H_2_L3) is described. All complexes (Pd/PtL3, PdL5/6, Pd/PtL8, Au/PdL9) with their respective ligands synthesized in this work are not accessible *via* the direct metalation of the respective ligand, due to irreproducible or unreliable results, except for AuL9. Even the route *via* the silver salt transmetalation does not lead to reliable results. The silver complexes of the respective ligands could not be isolated, probably due to instability of the respective complexes. Therefore, a modified synthetic method has been established. Here, *in situ* transmetalation with silver oxide is used in combination with the metal precursor and an excess of sodium acetate as a mild base, resulting in the corresponding complexes. Furthermore, the complexes PdL3, PdL8, AuL9 and their respective ligands were tested for their ability to induce apoptosis on Naml-6 and SK-N-AS cells. According to the experiments performed, the data suggest that AuL9 is capable of inducing apoptosis in malignant cells *via* the mitochondrial and ROS pathway. However, so far, an effect could only be observed on SK-N-AS neuroblastoma cells. In addition, a relatively high dose of AuL9 is required to induce apoptosis in neuroblastoma cells, which could be challenging for clinical applicability. AuL9 is able to overcome resistance to cisplatin in neuroblastoma cells (SK-N-AS) *in vitro*. Further characterization experiments would be required to determine the exact mechanism of action of AuL9, for example identification of molecular targets that are involved in the AuL9 induced apoptosis, as well as the selectivity for cancer cells.

## Experimental section

### General procedures and analytical methods

Unless otherwise stated, all manipulations were performed under normal atmosphere without dried and degassed chemicals. All syntheses regarding the complexes were conducted under the exclusion of light. Every work-up was performed under normal atmosphere without dried and degassed chemicals; the complexes' work-ups were conducted in addition under the exclusion of light unless otherwise stated. Purification, in case of the Pt and Pd complexes, is achieved by dissolving the crude product in MeCN and filtering it through basic aluminum oxide to remove impurities. Acidic aluminum oxide promotes the decomposition of the complexes while pH-neutral aluminum oxide leads in smaller yields.^6^ All obtained complexes are air- and water stable; however, PtL3, PtL8 and AuL9 decompose after extensive exposition to light. Solvents were obtained water-free from a MBraun solvent purification system and stored over molecular sieves (3 Å). The procedures for novel compounds obtained during the synthetic approaches to the saturated macrocyclic ligand precursor, containing 2-imidazoline moieties instead of imidazole, calix[4]imidazolinium, (2-imidazoline, *N*-benzyl-2-imidazoline, 3,3′-methylenebis(1-benzyl-2-imidazolinium)dibromide, *N*^1^,*N*^1^,*N*^2^,*N*^2^-tetrabenzylethane-1,2-diamine, *tert*-butyl (2-aminoethyl)carbamate, *tert*-butyl 2-imidazoline-1-carboxylate) are stated in the ESI.†*N*-Benzylethylenediamine (12),^57–59^ ethylenebis(trifluoromethanesulfonate) (4),^60^ 1,1′-ethylene-di-2-imidazoline (1)^23^ and 1,1′-ethylenebis-1*H*-imidazolyl (7)^25,61^ were synthesized according to literature procedures. All other reagents were purchased from commercial suppliers and used without further purification. NMR spectra were recorded on a Bruker Avance DPX 400 (^1^H-NMR, 400 MHz; ^13^C-NMR, 100 MHz; ^19^F-NMR, 376 MHz) and chemical shifts are given in *δ* values in ppm (parts per million) relative to TMS (tetramethylsilane) and reported relative to the residual signal of the deuterated solvent.^62^ Elemental analysis (C/H/N) were obtained by the Microanalytical Laboratory at Technische Universität München. Electrospray ionization mass spectrometry (ESI-MS) data were acquired on a Thermo Fisher Ultimate 3000 and with higher resolution (HR-ESI-MS) on Exactive Plus Orbitrap from Thermo Fisher.

### Synthetic procedures

#### Alkylbisimidazoline diiodide (2)

1 (5.00 g, 30.0 mmol, 1.00 eq.) is dissolved in MeCN (300 mL) and MeI (213 g, 1.50 mol, 50.0 eq.) is added. The resulting reaction mixture is heated to reflux for 4 h. After cooling to ambient temperature, all volatile compounds are removed *in vacuo*. The resulting crude material is redissolved in a small amount of MeCN (5 mL) and an off-white solid is precipitated after the addition of Et_2_O (40 mL). The crude material is collected *via* centrifugation and washed with (3 × 5 mL) Et_2_O. After removal of all volatile compounds *in vacuo*, 2 is obtained as an off-white solid (11.1 g, 24.7 mmol, 82%). ^1^H-NMR (400 MHz, DMSO-d_6_) *δ* (ppm) = 8.54 (s, 2H, N–C*H*–N), 3.91 (s, 8H, CH_3_–N–C*H*_2_–C*H*_2_), 3.70 (s, 4H, C*H*_2_–C*H*_2_), 3.12 (s, 6H, C*H*_3_). ^13^C-NMR (101 MHz, DMSO-d_6_) *δ* (ppm) = 159.12 (N–*C*H–N), 50.96 (*C*_(backbone)_), 48.70 (*C*_(backbone)_), 45.02 (*C*H_2_–*C*H_2_), 35.08 (*C*H_3_). Elemental analysis: for C_10_H_20_I_2_N_4_ (%) anal. calc.: C: 26.68, H: 4.48, N: 12.45, found: C: 26.66, H: 4.48, N: 12.39.

#### Alkylbisimidazolinium hexafluorophosphate (H_2_L3)

2 (100 mg, 222 μmol, 1.00 eq.) is dissolved in H_2_O (1 mL) and added to a solution of NH_4_PF_6_ (217 mg, 1.33 mmol, 6.00 eq.) in H_2_O (1 mL). The resulting white precipitate is collected, washed three times with H_2_O (2 mL, 2 mL, 1 mL) and dried subsequently *in vacuo*. Without further purification, the titled compound H_2_L3 is obtained as a white solid (98 mg, 202 mmol, 91%). ^1^H-NMR (400 MHz, DMSO-d_6_) *δ* (ppm) = 8.39 (s, 2H, N–C*H*–N), 3.88 (s, 8H, CH_3_–N–C*H*_2_–C*H*_2_), 3.67 (s, 4H, C*H*_2_–C*H*_2_), 3.11 (s, 6H, C*H*_3_). ^13^C-NMR (101 MHz, DMSO-d_6_) *δ* (ppm) = 158.77 (N–*C*H–N), 50.36 (*C*_(backbone)_), 48.12 (*C*_(backbone)_), 44.55 (*C*H_2_–*C*H_2_), 34.43 (*C*H_3_). ^19^F-NMR (376 MHz, DMSO-d_6_) *δ* (ppm) = −70.15 (d, ^1^*J*_P–F_ = 713 Hz, PF_6_^−^). Elemental analysis: for C_10_H_20_F_12_N_4_P_2_ (%) anal. calc.: C: 24.70, H: 4.15, N: 11.52, found: C: 24.28, H: 4.01, N: 11.17.

#### Calix[4](–Et–Et–)imidazoliniumtrifluoromethanesulfonate (H_4_L5)

1 (1.00 g, 6.17 mmol, 2.00 eq.) is dissolved in dry MeCN (1.5 L), cooled to −45 °C and a solution of 4 (2.02 g, 6.20 mmol, 2.01 eq.) in dry MeCN (50 mL) is added dropwise over 6 h. After the addition, the reaction mixture is stirred for 72 h at ambient temperature. All volatile compounds are removed *in vacuo* and the resulting crude material is dried subsequently *in vacuo*. Without further purification the titled compound H_4_L5 is obtained as an off-white solid (1.50 g, 1.54 mmol, 50%). Note: everything is conducted under inert conditions. ^1^H-NMR (400 MHz, DMSO-d_6_) *δ* (ppm) = 8.46 (s, 4H, N–C*H*–N), 3.95 (s, 16H, C*H*_2(bridge/backbone)_), 3.74 (s, 16H, C*H*_2(bridge/backbone)_). ^13^C-NMR (101 MHz, DMSO-d_6_) *δ* (ppm) = 159.16 (N–*C*H–N), 120.80 (q, ^1^*J*_19F_–_13C_ = 320 Hz, OTf^−^), 48.15 (*C*H_2(bridge/backbone)_), 44.54 (*C*H_2_–*C*H_2(bridge/backbone)_). ^19^F-NMR (376 MHz, DMSO-d_6_) *δ* (ppm) = −77.74 (C*F*_3_). Elemental analysis for C_24_H_36_N_8_O_12_F_12_S_4_ (%) anal. calc.: C 29.27; H 3.68; N 11.38; S 13.02 found: C 29.37; H 3.67; N 11.01; S 13.12.

#### Calix[4](–Et–Et–)imidazoliniumhexafluorophosphate (H_4_L6)

H_4_L5 (300 mg, 304 μmol, 1.00 eq.) is dissolved in H_2_O (50 mL) and added to a solution of NH_4_PF_6_ (223 mg, 1.37 mmol, 4.50 eq.) in H_2_O (50 mL). The resulting white precipitate is collected, washed three times with H_2_O (10 mL, 7 mL, 5 mL), Et_2_O (3 mL, 2 mL) and dried subsequently *in vacuo*. Without further purification, the titled compound H_4_L6 is obtained as a white solid (240 mg, 248 μmol, 81%). However, a small amount of OTf^−^ is still detectable in the ^19^F-NMR. ^1^H-NMR (400 MHz, DMSO-d_6_): *δ* (ppm) = 8.44 (s, 4H, N–C*H*–N), 3.93 (s, 16H, C*H*_2(backbone)_/C*H*_2,(bridge)_), 3.72 (s, 16H, C*H*_2,(bridge)_/C*H*_2,(backbone)_). ^1^H-NMR (400 MHz, CD_3_CN): *δ* (ppm) = 7.98–7.82 (m, 4H, N–C*H*–N), 4.00–3.82 (m, 16H, C*H*_2,(backbone)_/C*H*_2,(bridge)_), 3.73–3.65 (m, 16H, C*H*_2,(bridge)_/C*H*_2,(backbone)_). ^19^F-NMR (376 MHz, CD_3_CN): *δ* (ppm) = −72.45 (d, ^1^*J*_P–F_ = 713 Hz, PF_6_^−^). ESI-MS: *m*/*z* = calc. for [H_4_L6–PF_6_^−^]^+^: 823.20 ([M–PF_6_^−^]^+^); found: 822.94; calc. for [H_4_L6-2PF_6_^−^]^+^: 339.11 ([H_4_L6-2PF_6_^−^]^+^); found: 339.12.

#### Calix[4](–Et–Et–)imidazoliumtrifluoromethanesulfonate (H_4_L8)

7 (1.00 g, 6.17 mmol, 2.00 eq.) is dissolved in dry MeCN (1.5 L), cooled to −30 °C and a solution of 4 (2.02 g, 6.20 mmol, 2.01 eq.) in dry MeCN (100 mL) is added dropwise over 5 h. After the addition, the reaction mixture is stirred for 72 h at ambient temperature. All volatile compounds are removed *in vacuo* and the resulting crude material is washed eight times with cold acetone (10 mL, 5 mL, 5 mL, 3 mL, 3 mL, 2 mL, 2 mL, 1 mL) and dried subsequently *in vacuo*. Without further purification, the titled compound H_4_L8 is obtained as a white solid (1.50 g, 1.54 mmol, 50%). ^1^H-NMR (400 MHz, CD_3_CN) *δ* (ppm) = 8.57 (t, ^4^*J* = 1.6 Hz, 4H, N–C*H*–N), 7.39 (d, ^4^*J* = 1.7 Hz, 8H, C*H*), 4.71 (s, 16H, C*H*_2_). ^13^C-NMR (101 MHz, CD_3_CN) *δ* (ppm) = 138.49 (N–*C*H–N), 124.49 (H*C*=*C*H), 121.80 (q, ^1^*J*_19F_–_13C_ = 320 Hz, OTf^−^), 50.14 (*C*H_2_–*C*H_2_). ^19^F-NMR (376 MHz, CD_3_CN) *δ* (ppm) = −79.32 (C*F*_3_). ^1^H-NMR (400 MHz, DMSO-d_6_) *δ* (ppm) = 9.00 (t, ^4^*J* = 1.7 Hz, 4H, N–C*H*–N), 7.57 (d, ^4^*J* = 1.6 Hz, 8H, C*H*), 4.74 (s, 16H, C*H*_2_). ^13^C-NMR (101 MHz, DMSO-d_6_) *δ* (ppm) = 137.08 (N–*C*H–N), 123.28 (H*C*=*C*H), 120.66 (q, ^1^*J*_19F_–_13C_ = 320 Hz, OTf^−^), 49.24 (*C*H_2_–*C*H_2_). Elemental analysis for C_24_H_28_N_8_O_12_F_12_S_4_ (%) anal. calc.: C 29.54; H 2.84; N 11.54; S 13.13 found: C 29.54; H 2.84; N 11.54; S 13.25. ESI-MS: *m*/*z* [H_4_L8–4OTf^−^]^4+^ calc.: 95.06, found: 94.91, [H_4_L8–3OTf^−^]^3+^ calc.: 176.39, found 176.36, [H_4_L8–2OTf^−^]^2+^ calc.: 339.07, found: 339.22, [H_4_L8–1OTf^−^]^1+^ calc.: 827.03, found 826.93.

#### Calix[4](–Et–Et–)imidazoliumhexafluorophosphate (H_4_L9)

H_4_L8 (3.20 g, 3.28 mmol, 1.00 eq.) is dissolved in H_2_O (300 mL) and added to a solution of NH_4_PF_6_ (3.20 g, 19.66 mmol, 6.00 eq.) in H_2_O (50 mL). The resulting white precipitate is collected, washed three times with H_2_O (10 mL, 7 mL, 5 mL) and dried subsequently *in vacuo*. Without further purification, the titled compound H_4_L9 is obtained as a white solid (2.80 g, 2.85 mmol, 88%). ^1^H-NMR (400 MHz, CD_3_CN) *δ* (ppm) = 8.44 (t, ^4^*J* = 1.6 Hz, 4H, N–C*H*–N), 7.33 (d, ^4^*J* = 1.7 Hz, 8H, C*H*), 4.70 (s, 16H, C*H*_2_). ^19^F-NMR (376 MHz, CD_3_CN) *δ* (ppm) = −72.30 (d, ^1^*J*_FP_ = 713 Hz, PF_6_^−^). ^1^H-NMR (400 MHz, DMSO-d_6_) *δ* (ppm) = 9.00 (s, 4H, N–C*H*–N), 7.55 (d, ^4^*J* = 1.6 Hz, 8H, C*H*), 4.73 (s, 16H, C*H*_2_). ^13^C-NMR (101 MHz, DMSO-d_6_) *δ* (ppm) = 137.26 (N–*C*H–N), 123.49 (H*C*=*C*H), 49.44 (*C*H_2_–*C*H_2_). Elemental analysis for C_20_H_28_N_8_F_24_P_4_ (%) anal. calc.: C 25.01; H 2.94; N 11.67; S 0.00 found: C 25.08; H 2.90; N 11.31; S 0.00.

#### Pd[C^Et^C_imi_(Me)_2_C^Et^C_imi_(Me)_2_]hexafluorophosphate (PdL3)

Ag_2_O (150 mg, 648 μmol, 1.05 eq.) is added to a solution of H_2_L3 (300 mg, 617 μmol, 1.00 eq.) and NaOAc (202 mg, 2.47 mmol, 4.00 eq.) in dry MeCN (15 mL) and stirred for 1 h at ambient temperature, followed by the addition of Pd(OAc)_2_ (145 mg, 648 μmol, 1.05 eq.). The resulting reaction mixture is heated to 80 °C for 3 d. After cooling to ambient temperature, the reaction mixture is filtered over a short plug of basic aluminum oxide. The filter column is eluted with MeCN (20 mL) and all volatile compounds are removed *in vacuo*. The resulting crude material is resuspended in MeCN (5 mL) and centrifuged. Upon the addition of Et_2_O (20 mL) to the supernatant, a white solid is precipitated. The crude material is collected *via* centrifugation, washed with Et_2_O (3 × 5 mL), redissolved in MeCN (5 mL) and precipitated with Et_2_O (15 mL). After drying *in vacuo*, the titled compound PdL3 is obtained as an off-white solid (140 mg, 178 μmol, 29%). Single crystals suitable for SC-XRD were obtained by slow diffusion of Et_2_O into MeCN solution of PdL3. ^1^H-NMR (400 MHz, CD_3_CN) *δ* (ppm) = 4.32–4.22 (m, 4H, C*H*_2(backbone)_), 3.71–3.51 (m, 20H, C*H*_2_–C*H*_2_, C*H*_2(backbone)_), 2.97 (s, 12H, C*H*_3_). ^13^C-NMR (101 MHz, CD_3_CN) *δ* (ppm) = 195.6 (N–*C*–N), 51.72 (*C*_(bridge)_, *C*_(backbone)_), 51.26 (*C*_(bridge)_, *C*_(backbone)_), 46.5 (*C*H_2_), 37.62 (CH_3_). ^19^F-NMR (376 MHz, CD_3_CN): *δ* (ppm) = −72.94 (d, ^1^*J*_P–F_ = 706 Hz, P*F*_6_). ^1^H-NMR (400 MHz, DMSO-d_6_) *δ* (ppm) = 4.35–4.13 (m, 4H, C*H*_2(backbone)_), 3.82–3.57 (m, 20H, C*H*_2_–C*H*_2_, C*H*_2(backbone)_), 2.95 (s, 12H, C*H*_3_). ^13^C-NMR (101 MHz, DMSO-d_6_) *δ* (ppm) = 194.29 (N–*C*–N), 50.94 (*C*_(bridge)_, *C*_(backbone)_), 50.56 (*C*_(bridge)_, *C*_(backbone)_), 45.76 (*C*H_2_), 37.17 (CH_3_). Elemental analysis: for C_20_H_36_F_24_N_8_P_4_Pd_1_ (%) anal. calc.: C: 30.60, H: 4.62, N: 14.28, found: C: 30.93, H: 4.55, N: 14.14, S: 0.00. HR-ESI-MS: *m*/*z* [PdL3–2PF_6_^−^]^2+^ calc.: 247.1044, found: 247.1039, [PdL3–PF_6_^−^]^+^ calc.: 639.1735, found: 639.1720.

#### Pt[C^Et^C_imi_(Me)_2_C^Et^C_imi_(Me)_2_]hexafluorophosphate (PtL3)

Ag_2_O (150 mg, 648 μmol, 1.05 eq.) is added to a solution of H_2_L3 (300 mg, 617 μmol, 1.00 eq.) and NaOAc (202 mg, 2.47 mmol, 4.00 eq.) in dry MeCN (15 mL) and stirred for 1 h at ambient temperature, followed by the addition of PtCl_2_ (145 mg, 648 μmol, 1.05 eq.). The resulting reaction mixture is heated to 80 °C for 3 d. After cooling to ambient temperature, the reaction mixture is filtered over a short plug of basic aluminum oxide. The filter column is eluted with MeCN (20 mL) and all volatile compounds are removed *in vacuo*. The resulting crude material is resuspended in MeCN (5 mL) and centrifuged. Upon the addition of Et_2_O (20 mL) to the supernatant, a white solid is precipitated. The crude material is collected *via* centrifugation, washed with Et_2_O (3 × 5 mL) and redissolved in MeCN (5 mL) and precipitated with Et_2_O (15 mL). After drying *in vacuo*, the titled compound PtL3 is obtained as an off-white solid (23 mg, 26 μmol, 4%). Single crystals suitable for SC-XRD were obtained by slow diffusion of Et_2_O into MeCN solution of PtL3. Note; a clean EA could not be obtained, and the NMR includes impurities. ^1^H-NMR (400 MHz, CD_3_CN) *δ* (ppm) = 4.35–4.26 (m, 4H, C*H*_2(backbone)_), 3.72–3.51 (m, 20H, C*H*_2_–C*H*_2_, C*H*_2(backbone)_), 2.94 (s, 12H, C*H*_3_). ^13^C-NMR (101 MHz, CD_3_CN) *δ* (ppm) = 188.38 (N–*C*–N), 51.47 (d, *C*_(bridge)_, *C*_(backbone)_), 46.39 (*C*H_2_), 37.44 (CH_3_).

#### Pd[(cC^Et^CC^Et^C_imi_)OTf] (PdL5)

Ag_2_O (155 mg, 670 μmol, 2.20 eq.) is added to a solution of H_4_L5 (300 mg, 305 μmol, 1.00 eq.) and NaOAc (200 mg, 2.42 mmol, 8.00 eq.) in dry MeCN/DMSO (12 mL 1 : 1) and stirred for 1 h at ambient temperature, followed by the addition of Pd(OAc)_2_ (71.8 mg, 320 μmol, 1.05 eq.). The resulting reaction mixture is heated to 80 °C for 3 d and is filtered, after cooling to ambient temperature, over a short plug of basic aluminum oxide. The filter column is eluted with MeCN (100 mL) and all volatile compounds are removed *in vacuo*. The resulting oily solution (still approx. 6 mL of DMSO remaining) is resuspended in MeCN (6 mL) and centrifuged. Upon the addition of Et_2_O (25 mL) to the supernatant, a brown/black solid is precipitated. After another addition of Et_2_O (120 mL) a white solid is precipitated. The white crude material is collected *via* centrifugation, washed with Et_2_O (3 × 5 mL) and redissolved in MeCN (5 mL). After purification [3 times dissolving in MeCN (4 mL) and precipitating with Et_2_O (∼15 mL)] and removing all volatile compounds *in vacuo*, the titled compound PdL5 is obtained as an off-white solid (7.00 mg, 8.87 μmol, 3%). ^1^H-NMR (400 MHz, CD_3_CN): *δ* (ppm) = 4.10–4.00 (m, 8H, C*H*_2,(bridge)_), 3.76–3.54 (m, 16H, C*H*_2,(backbone)_), 3.52–3.45 (m, 8H, C*H*_2,(bridge)_). ^13^C-NMR (101 MHz, CD_3_CN): *δ* (ppm) = 191.3 (N–*C*–N), 51.0 (*C*H_2,(bridge)_/*C*H_2,(backbone)_), 47.4 (*C*H_2,(backbone)_/*C*H_2,(bridge)_). ^19^F-NMR (376 MHz, CD_3_CN): *δ* (ppm) = −79.33 (C*F*_3_). ESI-MS: *m*/*z* = calc. for [PdL5–OTf^−^]^+^: 639.13 ([PdL5–OTf^−^]^+^); found: 639.44; calc. for [PdL5–2OTf^−^]^+^: 245.09 ([M–2OTf^−^]^+^); found: 245.20.

#### Pd[(cC^Et^CC^Et^C_imi_)PF_6_] (PdL6)

PdL6 is synthesized analog to PdL5; by converting H_4_L6 (230 mg, 238 μmol, 1.00 eq.) with Ag_2_O (121 mg, 522 μmol, 2.20 eq.) in dry MeCN (4 mL) while stirring for 1 h at ambient temperature, followed by the addition of NaOAc (156 mg, 1.90 mmol, 8.00 eq.), Pd(OAc)_2_ (56.0 mg, 249 μmol, 1.05 eq.) and is heated at 75 °C for 4 d. After purification [3 times dissolving in MeCN (4 mL) and precipitating with Et_2_O (∼15 mL)] and removing all volatile compounds, PdL6 is obtained as a pale-yellow solid (85.0 mg, 109 μmol, 46%). ^1^H-NMR (400 MHz, CD_3_CN): *δ* (ppm) = 4.13–4.02 (m, 8H, C*H*_2,(bridge)_), 3.78–3.60 (m, 16H, C*H*_2,(backbone)_), 3.52–3.46 (m, 8H, C*H*_2,(bridge)_). ^19^F-NMR (376 MHz, CD_3_CN): *δ* (ppm) = −72.78 (d, ^1^*J*_P31–F19_ = 707 Hz, P*F*_6_) elemental analysis for C_20_H_32_F_12_N_8_P_2_Pd (%) anal. calc.: C 30.76; H 4.13; N 14.13; found: C 29.50; H 4.07; N 14.35. ESI-MS: *m*/*z* = calc. for [PdL6–PF_6_^−^]^+^: 635.14 ([M–PF_6_^−^]^+^); found: 635.21. HR-ESI-MS: *m*/*z* [PdL6–2PF_6_^−^]^2+^ calc.: 245.0887, found: 245.0890, [PdL6 + H_2_O–2PF_6_^−^]^2+^ calc.: 254.0940, found: 254.0944, [PdL6–PF_6_^−^]^+^ calc.: 635.1422, found: 635.1425, [PdL6 + H_2_O–PF_6_^−^]^+^ calc.: 653.1527, found: 653.1534.

#### Pd[(cC^Et^CC^Et^C)OTf] (PdL8)

Ag_2_O (74.7 mg, 322 μmol, 1.05 eq.) is added to a solution of H_4_L8 (320 mg, 307 μmol, 1.00 eq.) and NaOAc (202 mg, 2.46 mmol, 4.00 eq.) in dry MeCN (15 mL) and stirred for 1 h at ambient temperature, followed by the addition of Pd(OAc)_2_ (72.4 mg, 322 μmol, 1.05 eq.). The resulting reaction mixture is heated to 80 °C for 4 d. After cooling to ambient temperature, the reaction mixture is filtered over a short plug of basic aluminum oxide. The filter column is eluted with MeCN (50 mL) and all volatile compounds are removed *in vacuo*. The resulting crude material is resuspended in MeCN (5 mL) and centrifuged. Upon the addition of Et_2_O (20 mL) to the supernatant, a white solid is precipitated. The crude material is collected *via* centrifugation, washed with Et_2_O (3 × 5 mL) and redissolved in MeCN (5 mL). After the precipitation with Et_2_O (15 mL) and drying *in vacuo*, the titled compound PdL8 is obtained as an off-white solid (119 mg, 153 μmol, 50%). ^1^H-NMR (400 MHz, CD_3_CN) *δ* (ppm) = 7.20 (s, 8H, C*H*), 5.02–4.93 (m, 8H, C*H*_2_), 4.47–4.39 (m, 8H, C*H*_2_). ^13^C-NMR (101 MHz, CD_3_CN) *δ* (ppm) = 165.84 (N–*C*–N), 123.77 (*C*H), 49.11 (s, *C*H_2_–*C*H_2_). ^1^H-NMR (400 MHz, DMSO-d_6_) *δ* (ppm) = 7.52 (s, 8H, C*H*), 5.05–4.95 (m, 8H, C*H*_2_), 4.52–4.42 (m, 8H, C*H*_2_). ^13^C-NMR (101 MHz, DMSO-d_6_) *δ* (ppm) = 163.80 (N–*C*–N), 123.32 (*C*H), 48.14 (*C*H_2_–*C*H_2_). Elemental analysis for C_20_H_28_N_8_F_24_P_4_ + 1 MeCN (%) anal. calc.: C 35.07; H 3.31; N 15.33; S 7.80 found: C 35.26; H 3.21; N 15.73; S 7.82. HR-ESI-MS: *m*/*z* [PdL8–2OTf^−^]^2+^ calc.: 241.0574, found: 241.0570, [PdL8–OTf^−^]^+^ calc.: 631.0674, found: 631.0658.

#### Pd[(cC^Et^CC^Et^C)PF_6_] (PdL9)

PdL8 (95 mg, 122 μmol, 1.00 eq.) is dissolved in H_2_O (35 mL), after the addition of NH_4_PF_6_ (50.0 mg, 305 μmol, 2.5 eq.) a white precipitate is collected *via* centrifuge and washed three times with H_2_O (5 mL, 3 mL, 3 mL) and Et_2_O (10 mL, 5 mL, 3 mL). After drying *in vacuo*, the titled compound PdL9 is obtained as an off-white solid (39 mg, 50 μmol, 41%). Single crystals suitable for SC-XRD were obtained by slow diffusion of Et_2_O into MeCN solution of PdL8. ^1^H-NMR (400 MHz, CD_3_CN) *δ* (ppm) = 7.22 (s, 8H, C*H*), 4.97 (m, 8H, C*H*_2_), 4.43 (m, 8H, C*H*_2_). ^19^F-NMR (376 MHz, CD_3_CN) *δ* (ppm) = −72.93 (d, ^1^*J*_FP_ = 713 Hz, PF_6_^−^). HR-ESI-MS: *m*/*z* [PdL9–2PF_6_^−^]^2+^ calc.: 241.0574, found: 241.0570, [PdL9–PF_6_^−^]^+^ calc.: 627.0796, found: 627.0782.

#### Pt[(cC^Et^CC^Et^C)OTf] (PtL8)

Ag_2_O (209 mg, 900 μmol, 2.20 eq.) is added to a solution of H_4_L8 (400 mg, 410 μmol, 1.00 eq.) and NaOAc (202 mg, 2.46 mmol, 4.00 eq.) in dry MeCN (30 mL) and stirred for 1 h at ambient temperature, followed by the addition of Pt(MeCN)_2_Cl_2_ (156 mg, 450 μmol, 1.10 eq.). The resulting reaction mixture is heated to 80 °C for 3 d and is filtered, after cooling to ambient temperature, over a short plug of basic aluminum oxide. The filter column is eluted with MeCN (50 mL) and all volatile compounds are removed *in vacuo*. The resulting crude material is resuspended in MeCN (5 mL) and centrifuged. Upon the addition of Et_2_O (20 mL) to the supernatant, a white solid is precipitated. The crude material is collected *via* centrifugation, washed with Et_2_O (3 × 5 mL) and redissolved in MeCN (5 mL). After the precipitation with Et_2_O (15 mL) and drying *in vacuo*, the titled compound PtL8 is obtained as an off-white solid (90 mg, 103 μmol, 25%).^1^H-NMR (400 MHz, CD_3_CN) *δ* (ppm) = 7.19 (s, 8H, C*H*), 5.11–4.94 (m, 8H, C*H*_2_), 4.50–4.38 (m, 8H, C*H*_2_). ^1^H-NMR (400 MHz, DMSO-d_6_) *δ* (ppm) = 7.49 (s, 8H, C*H*), 5.06–4.97 (m, 8H, C*H*_2_), 4.54–4.44 (m, 8H, C*H*_2_). ^13^C-NMR (101 MHz, CD_3_CN) *δ* (ppm) = 159.39 (N–*C*H–N), 123.58 (H*C*=*C*H), 48.86 (*C*H_2_–*C*H_2_). ^19^F-NMR (376 MHz, CD_3_CN) *δ* (ppm) = −79.27 (C*F*_3_). HR-ESI-MS: *m*/*z* [PtL8–2OTf^−^]^2+^ calc.: 285.5881, found: 285.5864, [PtL8–OTf^−^]^+^ calc.: 720.1287, found: 720.1264.

#### Au[(cC^Et^CC^Et^C)PF_6_] (AuL9)

H_4_L8 (500 mg, 458 μmol, 1.00 eq.), KAuCl_4_ × 2H_2_O (209 mg, 505 μmol, 1.05 eq.), and NaOAc (197 mg, 2.41 mmol, 5.00 eq.) are suspended in dry DMSO (5 mL). The resulting reaction mixture is stirred for 5 h at 100 °C and filtered at ambient temperature. MeCN (5 mL) is added to the filtrate. After the addition of Et_2_O (30 mL) to the solution, white solid precipitated. It is washed with MeCN (3 × 5 mL) and DCM (2 × 5 mL) and after the removal of all volatiles *in vacuo*, the solid is dissolved in H_2_O (2 mL) and added dropwise to a solution of NH_4_PF_6_ (353 mg, 2.17 mmol, 4.00 eq.) in H_2_O (5 mL). The resulting white precipitate is collected and washed with H_2_O (3 × 5 mL) and after removal of all volatiles *in vacuo*, the titled compound AuL9 (230 mg, 228 mmol, 47%) is obtained as a white solid. ^1^H-NMR (400 MHz, CD_3_CN) *δ* (ppm) = 7.47 (s, 8H, C*H*), 4.89–4.77 (m, 8H, C*H*_2_), 4.76–4.66 (m, 8H, C*H*_2_). ^13^C-NMR (101 MHz, CD_3_CN) *δ* (ppm) = 146.03 (N–*C*H–N), 125.92 (H*C*=*C*H), 48.58 (*C*H_2_–*C*H_2_). Elemental analysis for C_20_H_24_AuF_18_N_8_P_3_ × 0.1 MeCN (%) anal. calc.: C 24.62; H 2.70; N 11.11; S 0.00 found: C 24.82; H 2.78; N 11.15; S 0.57. HR-ESI-MS: *m*/*z* [AuL9–3PF_6_^−^]^3+^ calc.: 191.0591, found: 191.0587, [AuL9–PF_6_^−^]^+^ calc.: 863.1068, found: 863.1038.

## Conflicts of interest

There are no conflicts to declare.

## Supplementary Material

RA-014-D4RA01195C-s001

RA-014-D4RA01195C-s002
