# Supplementary material for: Synthesis, characterization, and biomedical evaluation of ethylene-bridged tetra-NHC Pd(ii), Pt(ii) and Au(iii) complexes, with apoptosis-inducing properties in cisplatin-resistant neuroblastoma cells
Source: RSC Adv. 2024 Mar 27;14(15):10244–54. doi: 10.1039/d4ra01195c (PMC10967698; doi:10.1039/d4ra01195c)
Supplement: RA-014-D4RA01195C-s001 [file RA-014-D4RA01195C-s001.pdf]

Electronic Supplementary Material (ESI) for RSC Advances.
This journal is © The Royal Society of Chemistry 2024

Supporting information (SI)

Synthesis, Characterization, and Biomedical Evaluation of Ethylene-bridged tetra-NHC Pd(II), Pt(II) and Au(III) Complexes, with Apoptosis-inducing Properties in Cisplatin-resistant Neuroblastoma Cells

Wolfgang R. E. Büchele,[#,a] Tim P. Schlachta,[#,a] Andreas L. Gebendorfer,[a] Jenny Pamperin,[c,d] Leon F. Richter,[a] Michael J. Sauer,[a] Aram Prokop,*[,b,c,d] Fritz E. Kühn*[,a]

[a] Technical University of Munich, School of Natural Sciences, Department of Chemistry and Catalysis Research Center, Molecular Catalysis, Lichtenbergstraße 4, 85748 Garching, Germany. E-mail: fritz.kuehn@ch.tum.de; Tel. +49 (0)89 289 13477.

[b] Department of Pediatric Hematology/Oncology, Children's Hospital Cologne, Amsterdamer Straße 59, 50735 Cologne, Germany

[c] Department of Pediatric Oncology/Hematology, Helios Clinics Schwerin, Wismarsche Straße 393-397, 19055 Schwerin, Germany. E-mail: aram.prokop@helios-gesundheit.de.

[d] Department of Human Medicine, MSH Medical School Hamburg, Am Kaiserkai 1, 20457 Hamburg, Germany.

[*] Corresponding Author

# These authors contributed equally to this work.

Table of contents

1. Synthetic procedures 3

1,1'-Ethylene-di-2-imidazoline (1) 3

Alkylbisimidazolinium diiodide (2) 4

Alkylbisimidazolinium hexafluorophosphate (H_2L3) 5

Pd[$C^{Et}C_{imi}(Me)_2C^{Et}C_{imi}(Me)_2$] hexafluorophosphate (PdL3) 6

Pt[$C^{Et}C_{imi}(Me)_2C^{Et}C_{imi}(Me)_2$]hexafluorophosphate (PtL3) 7

Ethylenebis(trifluoromethansulfonate) (4) 8

Calix[4](-Et-Et-)imidazoliniumtrifluoromethansulfonate (H_4L5) 9

Calix[4](-Et-Et-)imidazoliniumhexafluorophosphate (H_4L6) 10

Pd[($cC^{Et}CC^{Et}C_{imi}$)OTf] (PdL5) 11

Pd[($cC^{Et}CC^{Et}C_{imi}$)PF_6] (PdL6) 12

1,1’-Ethylenebis-1*H*-imidazolyl (7) 13

Calix[4](-Et-Et-)imidazoliumtrifluoromethansulfonate (H_4L8) 14

Calix[4](-Et-Et-)imidazoliumhexafluorophosphate (H_4L15) 15

Pd[(cC^{Et}CC^{Et}C)OTf] (PdL8) 16

Pd[(cC^{Et}CC^{Et}C)PF_6] (PdL9) 17

Au[(cC^{Et}CC^{Et}C)PF_6] (AuL9) 18

Pt[(cC^{Et}CC^{Et}C)OTf] (PtL8) 19

2. Synthetic Approach to Calix[4]imidazolinium hexafluorophosphate 20

2-Imidazoline (10) 24

Synthetic attempts: bis(2-imidazolin-1-yl)methane (11)[10] 26

N-Benzylethylenediamine (12)[37-39] 34

N-Benzyl-2-imidazoline (13) 35

3,3'-Methylenebis(1-benzyl-2-imidazolinium) dibromide (14) 36

N^1,N^1,N^2,N^2-Tetrabenzylethane-1,2-diamine (15) 37

tert-Butyl (2-aminoethyl)carbamate (16) 38

tert-Butyl 2-imidazoline-1-carboxylate (17) – Method A 39

tert-Butyl 2-imidazoline-1-carboxylate (17) – Method B 40

3. Biological Studies 41

4. Analytic data 47

5. Crystallographic data 102

6. References SI 107

1. Synthetic procedures

1,1'-Ethylene-di-2-imidazoline (1)

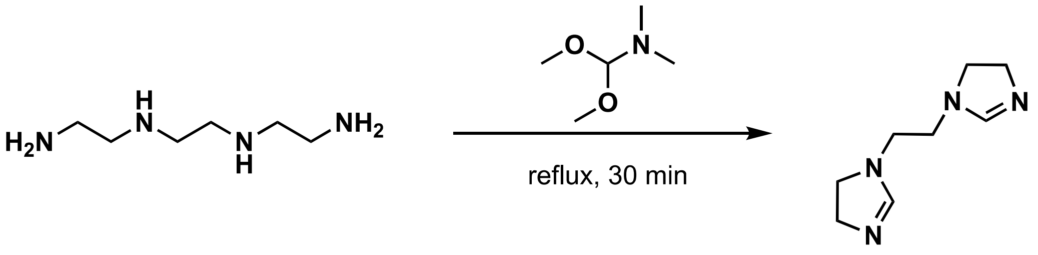

1

This compound is prepared according to a procedure, taken from literature.[1]

N,*N*-dimethylformamide dimethyl acetal (6.52 g, 0.55 mol, 2.00 eq.) is added to a solution of linear triethylenetetramine [TETA (4.00 g, 0.27 mol, 1.00 eq.)]. The resulting reaction mixture is heated to reflux for 30 min and all volatile compounds are removed *in vacuo*. The resulting off-white crude material is recrystallized from THF (20 mL). The resulting white solid is filtered under inert conditions and isolated. The titled compound **1** is obtained as a white solid (3.12 g, 0.18 mol, 69%).

^{1}H-NMR (400 MHz, $CDCl_3$) δ (ppm) = 6.79 (t, 2H, N-C*H*-N), 3.89-3.77 (m, 4H, CH_3-N-CH_2-CH_2), 3.26-3.22 (m, 7H, $CH_{2(backbone)}$), 3.21 (s, 1H, $CH_{2(backbone)}$).

^{13}C-NMR (101 MHz, $CDCl_3$) δ (ppm) = 157.44 (N-*C*H-N), 55.29 ($C_{(backbone)}$), 48.80 ($C_{(backbone)}$), 46.83 (*C*H_2-*C*H_2).

Elemental Analysis: for $C_8H_{14}N_4$ (%) anal. calc.: C: 57.80, H: 8.49, N: 33.71, found: C: 57.60, H: 8.72, N: 33.21.

Alkylbisimidazolinium diiodide (2)

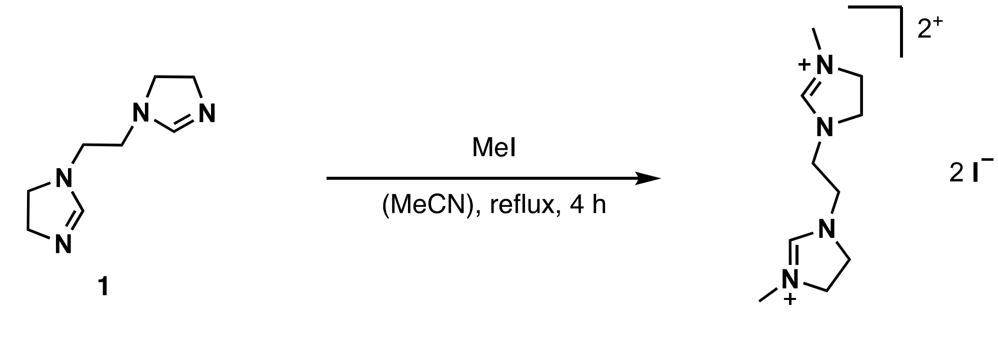

1 (5.00 g, 30.0 mmol, 1.00 eq.) is dissolved in MeCN (300 mL) and MeI (213 g, 1.50 mol, 50.0 eq.) is added. The resulting reaction mixture is heated to reflux for 4 h. After cooling to ambient temperature, all volatile compounds are removed *in vacuo*. The resulting crude material is redissolved in a small amount of MeCN (5 mL) and an off-white solid is precipitated after the addition of Et_2O (40 mL). The crude material is collected *via* centrifugation and washed with (3 × 5 mL) Et_2O. After removal of all volatile compounds *in vacuo*, **2** is obtained as an off-white solid (11.1 g, 24.7 mmol, 82%).

^{1}H-NMR (400 MHz, DMSO-d_6) δ (ppm) = 8.54 (s, 2H, N-C*H*-N), 3.91 (s, 8H, CH_3-N-CH_2-CH_2), 3.70 (s, 4H, CH_2-CH_2), 3.12 (s, 6H, CH_3).

^{13}C-NMR (101 MHz, DMSO-d_6) δ (ppm) = 159.12 (N-*C*H-N), 50.96 (*C*$_{(backbone)}$), 48.70 (*C*$_{(backbone)}$), 45.02 (*C*H_2-*C*H_2), 35.08 (*C*H_3).

Elemental Analysis: for $C_{10}H_{20}I_2N_4$ (%) anal. calc.: C: 26.68, H: 4.48, N: 12.45, found: C: 26.66, H: 4.48, N: 12.39.

Alkylbisimidazolinium hexafluorophosphate (H_2L3)

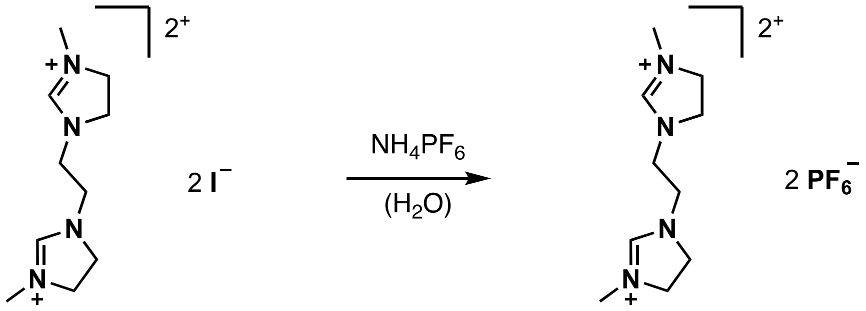

2 **H_2L3**

Analog to a procedure described by Kühn *et. al.*[2], **2** (100 mg, 222 µmol, 1.00 eq.) is dissolved in H_2O (1 mL) and added to a solution of NH_4PF_6 (217 mg, 1.33 mmol, 6.00 eq.) in H_2O (1 mL). The resulting white precipitate is collected, washed three times with H_2O (2 mL, 2 mL, 1 mL) and dried subsequently *in vacuo.* Without further purification, the titled compound **H_2L3** is obtained as a white solid (98 mg, 202 mmol, 91%).

^{1}H-NMR (400 MHz, DMSO-*d*$_6$) δ (ppm) = 8.39 (s, 2H, N-C*H*-N), 3.88 (s, 8H, CH_3-N-CH_2-CH_2), 3.67 (s, 4H, CH_2-CH_2), 3.11 (s, 6H, CH_3).

^{13}C-NMR (101 MHz, DMSO-*d*$_6$) δ (ppm) = 158.77 (N-*C*H-N), 50.36 ($C_{(backbone)}$), 48.12 ($C_{(backbone)}$), 44.55 (CH_2-CH_2), 34.43 (CH_3).

^{19}F-NMR (376 MHz, DMSO-*d*$_6$) δ (ppm) = -70.15 (d, $^1J_{P-F}$= 713 Hz, PF_6^-).

Elemental Analysis: for $C_{10}H_{20}F_{12}N_4P_2$ (%) anal. calc.: C: 24.70, H: 4.15, N: 11.52, found: C: 24.28, H: 4.01, N: 11.17.

Pd[C^{Et}C$_{imi}$(Me)$_2$C^{Et}C$_{imi}$(Me)$_2$] hexafluorophosphate (PdL3)

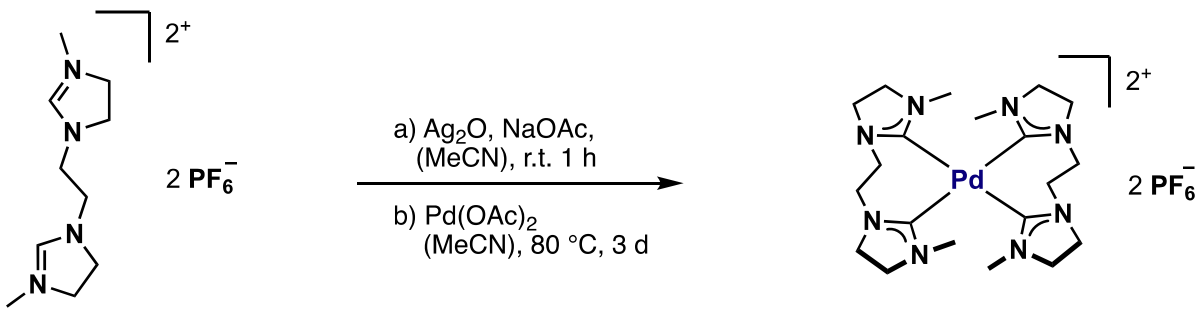

H_2L3 **PdL3**

Ag_2O (150 mg, 648 µmol, 1.05 eq.) is added to a solution of **H_2L3** (300 mg, 617 µmol, 1.00 eq.) and NaOAc (202 mg, 2.47 mmol, 4.00 eq.) in dry MeCN (15 mL) and stirred for 1 h at ambient temperature, followed by the addition of $Pd(OAc)_2$ (145 mg, 648 µmol, 1.05 eq.). The resulting reaction mixture is heated to 80 °C for 3 d. After cooling to ambient temperature, the reaction mixture is filtered over a short plug of basic aluminum oxide. The filter column is eluted with MeCN (20 mL) and all volatile compounds are removed *in vacuo*. The resulting crude material is resuspended in MeCN (5 mL) and centrifuged. Upon the addition of Et_2O (20 mL) to the supernatant, a white solid is precipitated. The crude material is collected *via* centrifugation, washed with Et_2O (3 × 5 mL), redissolved in MeCN (5 mL) and precipitated with Et_2O (15 mL). After drying *in vacuo*, the titled compound **PdL3** is obtained as an off-white solid (140 mg, 178 µmol, 29%).

^{1}H-NMR (400 MHz, CD_3CN) δ (ppm) = 4.32-4.22 (m, 4H, C$H_{2(backbone)}$), 3.71-3.51 (m, 20H, CH_2-CH_2, C$H_{2(backbone)}$), 2.97 (s, 12H, CH_3).

^{13}C-NMR (101 MHz, CD_3CN) δ (ppm) = 195.6 (N-*C*-N), 51.72 ($C_{(bridge)}$, $C_{(backbone)}$), 51.26 ($C_{(bridge)}$, $C_{(backbone)}$), 46.5 (CH_2), 37.62 (CH_3).

^{19}F-NMR (376 MHz, CD_3CN): δ (ppm) = -72.94 (d, $^1J_{P-F}$ = 706 Hz, PF_6).

^{1}H-NMR (400 MHz, DMSO-d$_6$) δ (ppm) = 4.35-4.13 (m, 4H, C$H_{2(backbone)}$), 3.82-3.57 (m, 20H, CH_2-CH_2, C$H_{2(backbone)}$), 2.95 (s, 12H, CH_3).

^{13}C-NMR (101 MHz, DMSO-d$_6$) δ (ppm) = 194.29 (N-*C*-N), 50.94 ($C_{(bridge)}$, $C_{(backbone)}$), 50.56 ($C_{(bridge)}$, $C_{(backbone)}$), 45.76 (CH_2), 37.17 (CH_3).

Elemental Analysis: for $C_{20}H_{36}F_{24}N_8P_4Pd_1$ (%) anal. calc.: C: 30.60, H: 4.62, N: 14.28, found: C: 30.93, H: 4.55, N: 14.14, S: 0.00.

HR-ESI-MS: m/z [**PdL3** – 2 PF_6^-]$^{2+}$ calc: 247.1044, found: 247.1039, [**PdL3** – PF_6^-]$^+$ calc: 639.1735, found: 639.1720.

Pt[C^{Et}C$_{imi}$(Me)$_2$C^{Et}C$_{imi}$(Me)$_2$]hexafluorophosphate (PtL3)

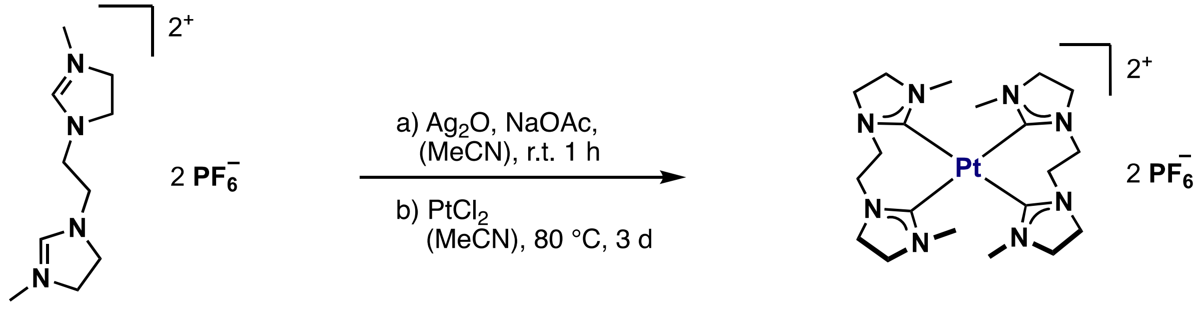

H$_2$L3 **PtL3**

Ag_2O (150 mg, 648 µmol, 1.05 eq.) is added to a solution of **H$_2$L3** (300 mg, 617 µmol, 1.00 eq.) and NaOAc (202 mg, 2.47 mmol, 4.00 eq.) in dry MeCN (15 mL) and stirred for 1 h at ambient temperature, followed by the addition of $PtCl_2$ (145 mg, 648 µmol, 1.05 eq.). The resulting reaction mixture is heated to 80 °C for 3 d. After cooling to ambient temperature, the reaction mixture is filtered over a short plug of basic aluminum oxide. The filter column is eluted with MeCN (20 mL) and all volatile compounds are removed *in vacuo*. The resulting crude material is resuspended in MeCN (5 mL) and centrifuged. Upon the addition of Et_2O (20 mL) to the supernatant, a white solid is precipitated. The crude material is collected *via* centrifugation, washed with Et_2O (3 × 5 mL) and redissolved in MeCN (5 mL) and precipitated with Et_2O (15 mL). After drying *in vacuo*, the titled compound **PtL3** is obtained as an off-white solid (23 mg, 26 µmol, 4%). Note; a clean EA could not be obtained, and the NMR includes impurities.

^{1}H-NMR (400 MHz, CD_3CN) δ (ppm) = 4.35-4.26 (m, 4H, $CH_{2(backbone)}$), 3.72-3.51 (m, 20H, CH_2-CH_2, $CH_{2(backbone)}$), 2.94 (s, 12H, CH_3).

^{13}C-NMR (101 MHz, CD_3CN) δ (ppm) = 188.38 (N-*C*-N), 51.47 (d, *C*$_{(bridge)}$, *C*$_{(backbone)}$), 46.39 (CH_2), 37.44 (CH_3).

Ethylenebis(trifluoromethansulfonate) (4)

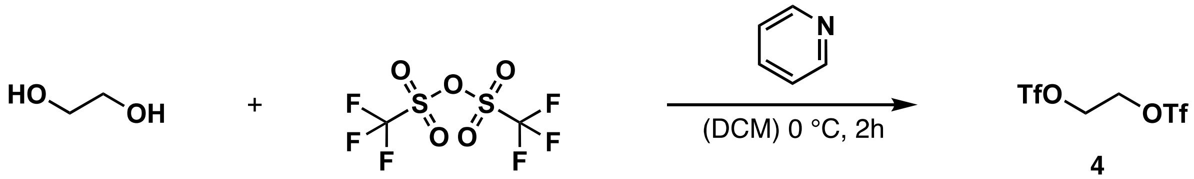

This compound is prepared according to a procedure taken from literature.[3]

A mixture of ethylene glycol (1.40 mL, 25.3 mmol, 1.00 eq.) and pyridine (4.09 mL, 50.5 mmol, 2.00 eq.) in DCM (25 mL) is added to a cooled solution of trifluoromethanesulfonic anhydride (14.25 g, 50.5 mmol, 2.00 eq.) in DCM (50 mL) at 0 °C over a period of 1 h. The resulting reaction mixture is stirred at 0 °C for 1 h and then washed with H_2O (3 × 10 mL). The organic layer is dried over Na_2SO_4, filtered through a plug of silica (3 cm) and washed with CH_2Cl_2 (100 mL). After removal of all volatile compounds *in vacuo,* the titled compound **4** (7 00 g, 21.5 mmol, 85%) is obtained as a brownish liquid.

^{1}H-NMR (400 MHz, $CDCl_3$) δ (ppm) = 4.77 (s, 4H, CH_2-CH_2).

Results are confirmed in accordance with those reported in literature.[3]

Calix[4](-Et-Et-)imidazoliniumtrifluoromethansulfonate (H_4L5)

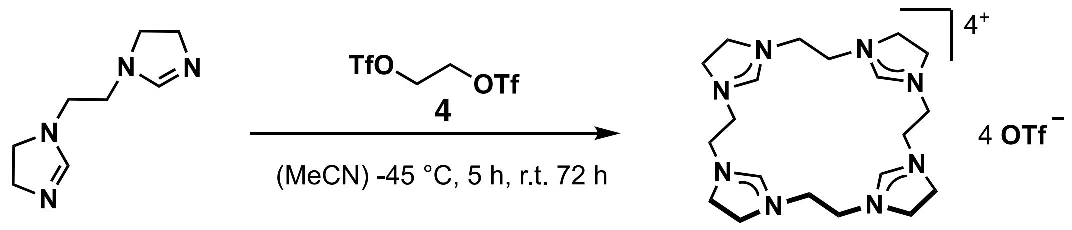

1 H4L5

Following a procedure known from literature for similar compounds,[33] **1** (1.00 g, 6.17 mmol, 2.00 eq.) is dissolved in dry MeCN (1.5 L) under inert conditions, cooled to -45 °C [dry Acetone (1.5 L); -90 °C, 1 h] and a solution of **4** (2.02 g, 6.20 mmol, 2.01 eq.) in dry MeCN (50 mL) [dry Acetone (50 mL)] is added dropwise over 6 h. After the addition, the reaction mixture is stirred for 72 h at ambient temperature. All volatile compounds are removed *in vacuo* and the resulting crude material is dried subsequently *in vacuo*. Without further purification the titled compound **H_4L5** is obtained as an off-white solid (1.50 g, 1.54 mmol, 50%). Note: Everything is conducted under inert conditions.

^{1}H-NMR (400 MHz, DMSO-d_6) δ (ppm) = 8.46 (s, 4H, N-C*H*-N), 3.95 (s, 16H, CH_2), 3.74 (s, 16H, CH_2).

^{13}C-NMR (101 MHz, DMSO-d_6) δ (ppm) = 159.16 (N-*C*H-N), 120.80 (q, $^1J_{19F-13C}$ = 320 Hz, OTf$^-$), 48.15 (*C*H$_2$), 44.54 (*C*H$_2$).

^{19}F-NMR (376 MHz, DMSO-d_6) δ (ppm) = -77.74 (CF_3).

Elemental analysis for $C_{24}H_{36}N_8O_{12}F_{12}S_4$ (%) anal. calc.: C 29.27; H 3.68; N 11.38; S 13.02 found: C 29.37; H 3.67; N 11.01; S 13.12.

Calix[4](-Et-Et-)imidazoliniumhexafluorophosphate (H_4L6)

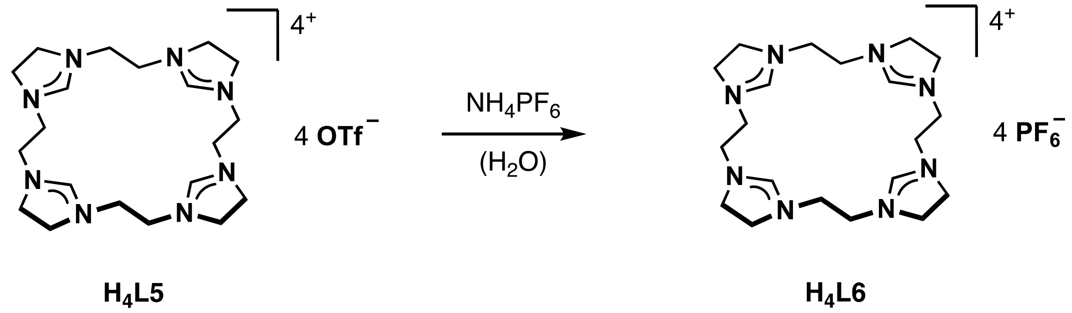

Analog to a procedure described by Kühn *et. al.*,[2] **H_4L5** (300 mg, 304 µmol, 1.00 eq.) is dissolved in H_2O (50 mL) and added to a solution of NH_4PF_6 (223 mg, 1.37 mmol, 4.50 eq.) in H_2O (50 mL). The resulting white precipitate is collected, washed three times with H_2O (10 mL, 7 mL, 5 mL), Et_2O (3 mL, 2 mL) and dried subsequently *in vacuo.* Without further purification, the titled compound **H_4L6** is obtained as a white solid (240 mg, 248 µmol, 81%). However, a small amount of OTf^- is still detectable in the ^{19}F-NMR.

^{1}H-NMR (400 MHz, DMSO-d_6): δ (ppm) = 8.44 (s, 4H, N-C*H*-N), 3.93 (s, 16H, C$H_{2(backbone)}$/ C$H_{2,(bridge)}$), 3.72 (s, 16H, C$H_{2,(bridge)}$/ C$H_{2,(backbone)}$).

^{1}H-NMR (400 MHz, CD_3CN): δ (ppm) = 7.98 – 7.82 (m, 4H, N-C*H*-N), 4.00 – 3.82 (m, 16H, C$H_{2,(backbone)}$/ C$H_{2,(bridge)}$), 3.73 – 3.65 (m, 16H, C$H_{2,(bridge)}$/ C$H_{2,(backbone)}$).

^{19}F-NMR (376 MHz, CD_3CN): δ (ppm) = -72.45 (d, $^1J_{P\text{-}F}$ = 713 Hz, PF_6^-).

ESI-MS: m/z = calc. for [**H_4L6**-PF_6^-]$^+$: 823.20 ([M-PF_6^-]$^+$); found: 822.94; calc. for [**H_4L6**-2PF_6^-]$^+$: 339.11 ([**H_4L6**-2PF_6^-]$^+$); found: 339.12.

Pd[($cC^{Et}CC^{Et}C_{imi}$)OTf] (PdL5)

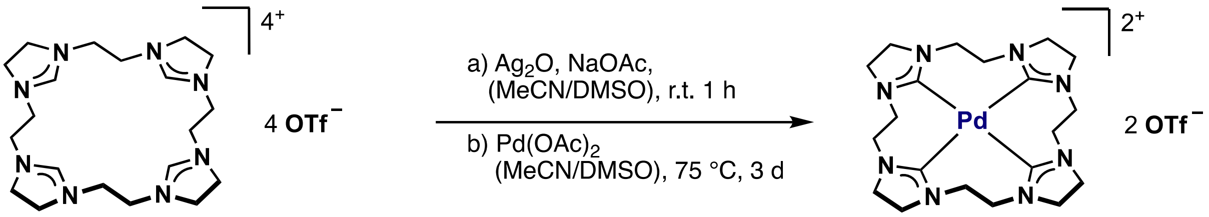

H_4L5 **PdL5**

Ag_2O (155 mg, 670 µmol, 2.20 eq.) is added to a solution of **H_4L5** (300 mg, 305 µmol, 1.00 eq.) and NaOAc (200 mg, 2.42 mmol, 8.00 eq.) in dry MeCN/DMSO (12 mL 1:1) and stirred for 1 h at ambient temperature, followed by the addition of $Pd(OAc)_2$ (71.8 mg, 320 µmol, 1.05 eq.). The resulting reaction mixture is heated to 80 °C for 3 d and is filtered, after cooling to ambient temperature, over a short plug of basic aluminum oxide. The filter column is eluted with MeCN (100 mL) and all volatile compounds are removed *in vacuo*. The resulting oily solution (still approx. 6 mL of DMSO remaining) is resuspended in MeCN (6 mL) and centrifuged. Upon the addition of Et_2O (25 mL) to the supernatant, a brown/black solid is precipitated. After another addition of Et_2O (120 mL) a white solid is precipitated. The white crude material is collected *via* centrifugation, washed with Et_2O (3 × 5 mL) and redissolved in MeCN (5 mL). After purification [3 times dissolving in MeCN (4 mL) and precipitating with Et_2O (~15 mL)] and removing all volatile compounds *in vacuo*, the titled compound **PdL5** is obtained as an off-white solid (7.00 mg, 8.87 µmol, 3%).

^{1}H-NMR (**PdL5**) (400 MHz, CD_3CN): δ (ppm) = 4.10 – 4.00 (m, 8H, C$H_{2,(bridge)}$), 3.76 – 3.54 (m, 16H, C$H_{2,(backbone)}$), 3.52 – 3.45 (m, 8H, C$H_{2,(bridge)}$).

^{13}C-NMR (**PdL5**) (101 MHz, CD_3CN): δ (ppm) = 191.3 (N-*C*-N), 51.0 (*C*$H_{2,(bridge)}$/ *C*$H_{2,(backbone)}$), 47.4 (*C*$H_{2,(backbone)}$/ *C*$H_{2,(bridge)}$).

^{19}F-NMR (**PdL5**) (376 MHz, CD_3CN): δ (ppm) = -79.33 (C*F*$_3$).

ESI-MS: m/z = calc. for [**PdL5**-OTf^-]$^+$: 639.13 ([M-OTf^-]$^+$); found: 639.44; calc. for [**PdL5**-2OTf^-]$^+$: 245.09 ([M-2OTf^-]$^+$); found: 245.20.

Pd[(cC^{Et}CC^{Et}C$_{imi}$)PF$_6$] (PdL6)

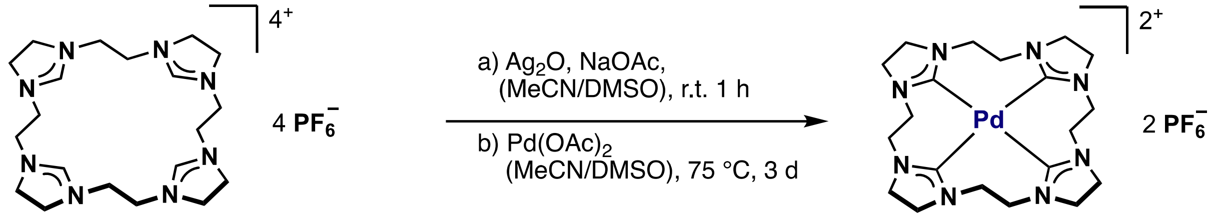

H$_4$L6 **PdL6**

PdL6 is synthesized analog to **PdL5**; by converting **H$_4$L6** (230 mg, 238 µmol, 1.00 eq.) with Ag_2O (121 mg, 522 µmol, 2.20 eq.) in dry MeCN (4 mL) while stirring for 1 h at ambient temperature, followed by the addition of NaOAc (156 mg, 1.90 mmol, 8.00 eq.), $Pd(OAc)_2$ (56.0 mg, 249 µmol, 1.05 eq.) and is heated at 75 °C for 4 d. After purification [3 times dissolving in MeCN (4 mL) and precipitating with Et_2O (~15 mL)] and removing all volatile compounds, **PdL6** is obtained as a pale-yellow solid (85.0 mg, 109 µmol, 46%).

^{1}H-NMR (**PdL6**) (400 MHz, CD_3CN): δ (ppm) = 4.13 – 4.02 (m, 8H, C$H_{2,(bridge)}$), 3.78 – 3.60 (m, 16H, C$H_{2,(backbone)}$), 3.52 – 3.46 (m, 8H, C$H_{2,(bridge)}$).

^{19}F–NMR (**PdL6**) (376 MHz, CD_3CN): δ (ppm) = -72.78 (d, $^1J_{P31\text{-}F19}$ = 707 Hz, PF_6)

Elemental Analysis for $C_{20}H_{32}F_{12}N_8P_2Pd$ (%) anal. calc.: C 30.76; H 4.13; N 14.13; found: C 29.50; H 4.07; N 14.35.

ESI-MS: m/z = calc. for [**PdL6**-PF_6^-]$^+$: 635.14 ([M-PF_6^-]$^+$); found: 635.21.

HR-ESI-MS: m/z [**PdL6** – 2 PF_6^-]$^{2+}$ calc: 245.0887, found: 245.0890, [**PdL6** + H_2O – 2 PF_6^-]$^{2+}$ calc: 254.0940, found: 254.0944, [**PdL6** – PF_6^-]$^+$ calc: 635.1422, found: 635.1425, [**PdL6** + H_2O – PF_6^-]$^+$ calc: 653.1527, found: 653.1534.

1,1'-Ethylenebis-1*H*-imidazolyl (7)

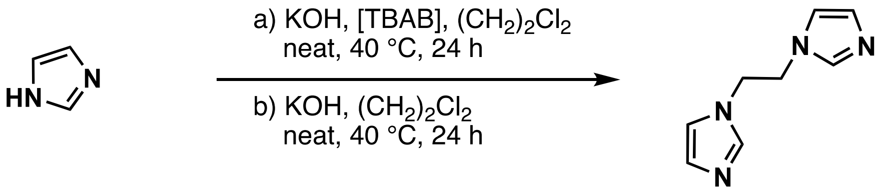

7

This compound is prepared according to a procedure, taken from literature.[4]

A mixture of imidazole (4.30 g, 63.4 mmol, 1.00 eq.), finely powdered KOH (85%, 2.17 g, 31.1 mmol, 0.50 eq.) and tetra-n-butylammonium bromide (168 mg, 631 µmol, 0.01 eq.) is stirred at RT for 1 h followed by the addition of 1,2-dichloroethane (2.50 mL, 31.1 mmol, 0.50 eq.). The resulting mixture is heated to 40 °C for 24 h followed by the addition of finely powdered KOH (2.50 g, 31.1 mmol) and 1,2-dichloroethane (2.50 mL, 31.1 mmol, 0.50 eq.). After 24 h, the mixture is extracted with chloroform (5 × 20 mL) and the combined organic fractions are concentrated to 20 mL *in vacuo* followed by the addition of hexane (5 mL). The titled compound **7** is obtained as a pale yellow crystalline solid (1.36 g, 8.4 mmol, 13.3%).

^{1}H NMR (300 MHz, DMSO-d_6) δ (ppm) = 7.38 (s, 2H, N-C*H*-N), 7.01 (s, 2H, C*H)*, 6.87 (s, 2H, C*H*), 4.33 (s, 4H, CH_2-CH_2).

^{13}C-NMR (101 MHz, DMSO-d_6) δ (ppm) = 137.59 (N-*C*H-N), 128.81 (*C*H=*C*H), 119.36 (*C*H=*C*H), 46.88 (*C*H_2-*C*H_2).

Results are confirmed in accordance with those reported in literature.[4]

Calix[4](-Et-Et-)imidazoliumtrifluoromethansulfonate (H_4L8)

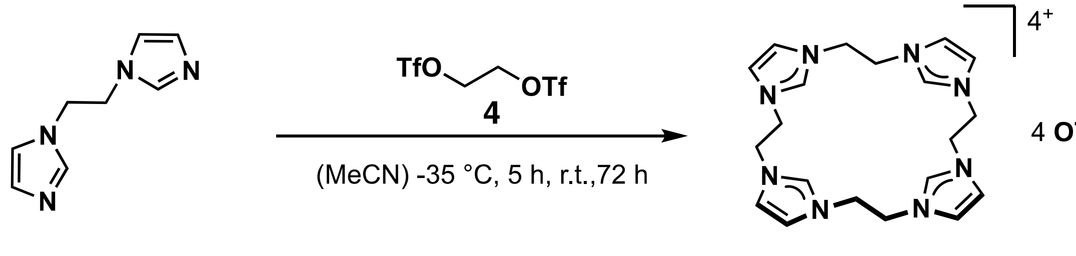

7 H_4L8

Following a procedure, which is based on the master thesis of *W.R.E. Büchele*.[5] **7** (1.00 g, 6.17 mmol, 2.00 eq.) is dissolved in dry MeCN (1.5 L) under inert conditions, cooled to -35 °C and a solution of **4** (2.02 g, 6.20 mmol, 2.01 eq.) in dry MeCN (100 mL) is added dropwise over 5 h. After the addition, the reaction mixture is stirred for 72 h at ambient temperature. All volatile compounds are removed *in vacuo* and the resulting crude material is washed eight times with cold acetone (10 mL, 5 mL, 5 mL, 3 mL, 3 mL, 2 mL, 2 mL, 1 mL) and dried subsequently *in vacuo*. Without further purification, the titled compound **H_4L8** is obtained as a white solid (1.50 g, 1.54 mmol, 50%).

^{1}H-NMR (400 MHz, CD_3CN) δ (ppm) = 8.57 (t, ^4J = 1.6 Hz, 4H, N-C*H*-N), 7.39 (d, ^4J = 1.7 Hz, 8H, C*H*), 4.71 (s, 16H, CH_2).

^{13}C-NMR (101 MHz, CD_3CN) δ (ppm) = 138.49 (N-*C*H-N), 124.49 (H*C*=*C*H), 121.80 (q, $^1J_{19F-13C}$ = 320 Hz, OTf^-), 50.14 (CH_2-CH_2).

^{19}F-NMR (376 MHz, CD_3CN) δ (ppm) = -79.32 (CF_3).

^{1}H-NMR (400 MHz, DMSO-d_6) δ (ppm) = 9.00 (t, ^4J = 1.7 Hz, 4H, N-C*H*-N), 7.57 (d, ^4J = 1.6 Hz, 8H, C*H*), 4.74 (s, 16H, CH_2).

^{13}C-NMR (101 MHz, DMSO-d_6) δ (ppm) = 137.08 (N-*C*H-N), 123.28 (H*C*=*C*H), 120.66 (q, $^1J_{19F-13C}$ = 320 Hz, OTf^-), 49.24 (CH_2-CH_2).

Elemental analysis for $C_{24}H_{28}N_8O_{12}F_{12}S_4$ (%) anal. calc.: C 29.54; H 2.84; N 11.54; S 13.13 found: C 29.54; H 2.84; N 11.54; S 13.25.

ESI-MS: m/z [**H_4L8**-4OTf$^-$]$^{4+}$ calc: 95.06, found: 94.91, [**H_4L8**-3OTf$^-$]$^{3+}$ calc: 176.39, found 176.36, [**H_4L8**-2OTf$^-$]$^{2+}$ calc: 339.07, found: 339.22, [**H_4L8**-1OTf$^-$]$^{1+}$ calc: 827.03, found 826.93.

Calix[4](-Et-Et-)imidazoliumhexafluorophosphate (H_4L15)

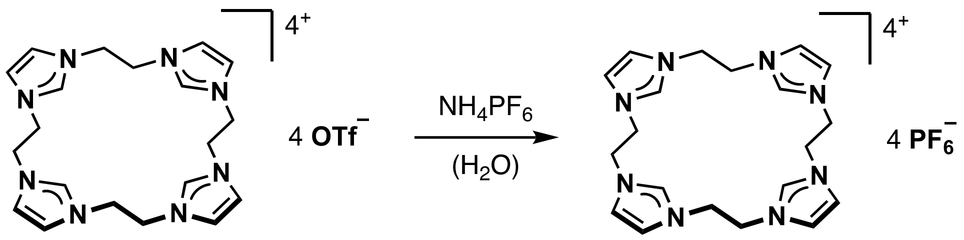

H_4L8 **H_4L9**

Based on the master thesis of *W.R.E. Büchele*.[5] **H_4L8** (3.20 g, 3.28 mmol, 1.00 eq.) is dissolved in H_2O (300 mL) and added to a solution of NH_4PF_6 (3.20 g, 19.66 mmol, 6.00 eq.) in H_2O (50 mL). The resulting white precipitate is collected, washed three times with H_2O (10 mL, 7 mL, 5 mL) and dried subsequently *in vacuo.* Without further purification, the titled compound **H_4L9** is obtained as a white solid (2.80 g, 2.85 mmol, 88%).

^{1}H-NMR (400 MHz, CD_3CN) δ (ppm) = 8.44 (t, ^4J = 1.6 Hz, 4H, N-C*H*-N), 7.33 (d, ^4J = 1.7 Hz, 8H, C*H*), 4.70 (s, 16H, CH_2).

^{19}F-NMR (376 MHz, CD_3CN) δ (ppm) = -72.30 (d, $^1J_{FP}$ = 713 Hz, PF_6^-).

^{1}H-NMR (400 MHz, DMSO-d_6) δ (ppm) = 9.00 (s, 4H, N-C*H*-N), 7.55 (d, ^4J = 1.6 Hz, 8H, C*H*), 4.73 (s, 16H, CH_2).

^{13}C-NMR (101 MHz, DMSO-d_6) δ (ppm) = 137.26 (N-*C*H-N), 123.49 (H*C*=*C*H), 49.44 (*C*H_2-*C*H_2).

Elemental analysis for $C_{20}H_{28}N_8F_{24}P_4$ (%) anal. calc.: C 25.01; H 2.94; N 11.67; S 0.00 found: C 25.08; H 2.90; N 11.31; S 0.00.

Pd[(cC^{Et}CC^{Et}C)OTf] (PdL8)

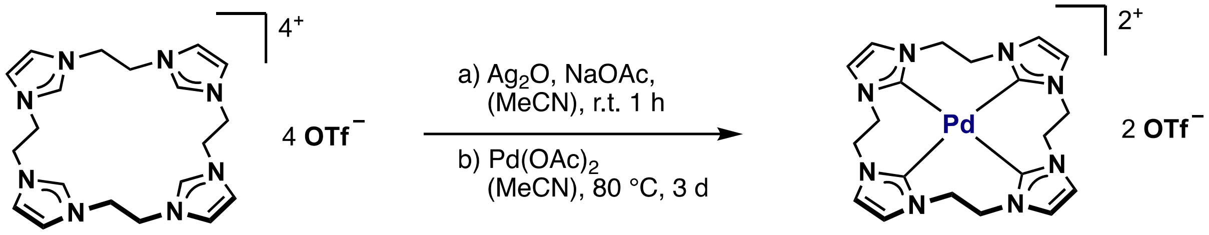

H_4L8 **PdL8**

Ag_2O (74.7 mg, 322 µmol, 1.05 eq.) is added to a solution of **H_4L8** (320 mg, 307 µmol, 1.00 eq.) and NaOAc (202 mg, 2.46 mmol, 4.00 eq.) in dry MeCN (15 mL) and stirred for 1 h at ambient temperature, followed by the addition of $Pd(OAc)_2$ (72.4 mg, 322 µmol, 1.05 eq.). The resulting reaction mixture is heated to 80 °C for 4 d. After cooling to ambient temperature, the reaction mixture is filtered over a short plug of basic aluminum oxide. The filter column is eluted with MeCN (50 mL) and all volatile compounds are removed *in vacuo*. The resulting crude material is resuspended in MeCN (5 mL) and centrifuged. Upon the addition of Et_2O (20 mL) to the supernatant, a white solid is precipitated. The crude material is collected *via* centrifugation, washed with Et_2O (3 × 5 mL) and redissolved in MeCN (5 mL). After the precipitation with Et_2O (15 mL) and drying *in vacuo*, the titled compound **PdL8** is obtained as an off-white solid (119 mg, 153 µmol, 50 %).

^{1}H-NMR (PdL8) (400 MHz, CD_3CN) δ (ppm) = 7.20 (s, 8H, C*H*), 5.02-4.93 (m, 8H, CH_2), 4.47-4.39 (m, 8H, CH_2).

^{13}C-NMR (PdL8) (101 MHz, CD_3CN) δ (ppm) = 165.84 (N-*C*-N), 123.77 (*C*H), 49.11 (s, *C*H_2-*C*H_2).

^{1}H-NMR (PdL8) (400 MHz, DMSO-d_6) δ (ppm) = 7.52 (s, 8H, C*H*), 5.05-4.95 (m, 8H, CH_2), 4.52-4.42 (m, 8H, CH_2).

^{13}C-NMR (PdL8) (101 MHz, DMSO-d_6) δ (ppm) = 163.80 (N-*C*-N), 123.32 (*C*H), 48.14 (*C*H_2-*C*H_2).

Elemental analysis for $C_{22}H_{24}F_6N_8O_6PdS_2$ * 0.1 MeCN (%) anal. calc.: C 35.07; H 3.31; N 15.33; S 7.80 found: C 35.26; H 3.21; N 15.73; S 7.82.

HR-ESI-MS: m/z [**PdL8** – 2 OTf^-]$^{2+}$ calc: 241.0574, found: 241.0570, [**PdL8** – OTf^-]$^+$ calc: 631.0674, found: 631.0658.

Pd[(cC^EtCC^EtC)PF$_6$] (PdL9)

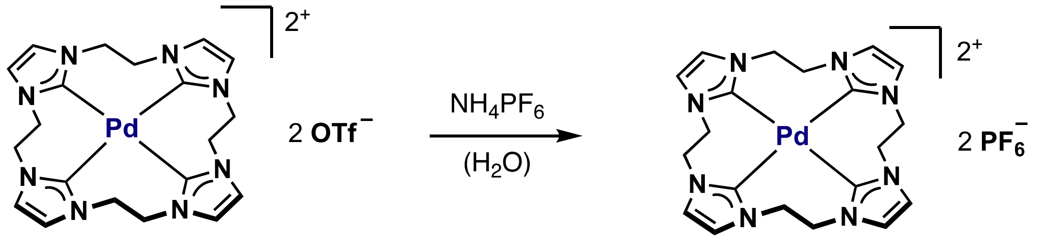

PdL8 **PdL9**

PdL8 (95 mg, 122 µmol, 1.00 eq.) is dissolved in H_2O (35 mL), after the addition of NH_4PF_6 50.0 mg, 305 µmol, 2.5 eq.) a white precipitate is collected *via* centrifuge and washed three times with H_2O (5 mL, 3 mL, 3 mL) and Et_2O (10 mL, 5 mL, 3 mL). After drying *in vacuo*, the titled compound **PdL9** is obtained as an off-white solid (39 mg, 50 µmol, 41%).

^{1}H-NMR (PdL9) (400 MHz, CD_3CN) δ (ppm) = 7.22 (s, 8H, C*H*), 4.97 (m, 8H, CH_2), 4.43 (m, 8H, CH_2).

^{19}F-NMR (PdL9) (376 MHz, CD_3CN) δ (ppm) = -72.93 (d, $^1J_{FP}$ = 713 Hz, PF_6^-).

HR-ESI-MS: m/z [**PdL9** – 2 PF_6^-]$^{2+}$ calc: 241.0574, found: 241.0570, [**PdL9** – PF_6^-]$^+$ calc: 627.0796, found: 627.0782.

Au[(cC^{Et}CC^{Et}C)PF$_6$] (AuL9)

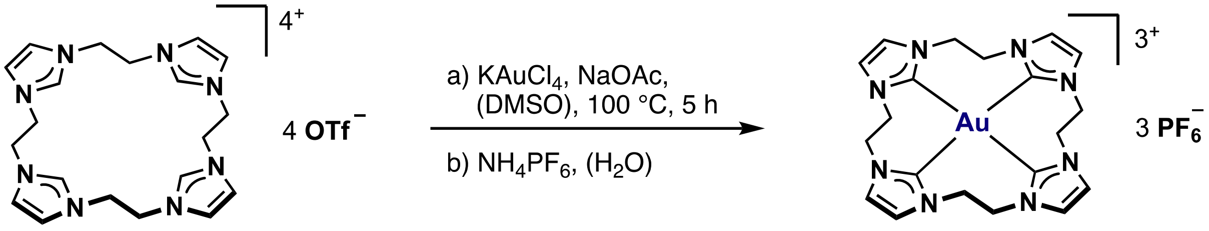

Following a literature procedure for similar compounds.[6] **H_4L8** (500 mg, 458 μmol, 1.00 eq.), $KAuCl_4$ × $2H_2O$ (209 mg, 505 μmol, 1.05 eq.), and NaOAc (197 mg, 2.41 mmol, 5.00 eq.) are suspended in dry DMSO (5 mL). The resulting reaction mixture is stirred for 5 h at 100 °C and filtered at ambient temperature. MeCN (5 mL) is added to the filtrate. After the addition of Et_2O (30 mL) to the solution, white solid precipitated. It is washed with MeCN (3 × 5 mL) and DCM (2 × 5 mL) and after the removal of all volatiles *in vacuo*, the solid is dissolved in H_2O (2 mL) and added dropwise to a solution of NH_4PF_6 (353 mg, 2.17 mmol, 4.00 eq.) in H_2O (5 mL). The resulting white precipitate is collected and washed with H_2O (3 × 5 mL) and after removal of all volatiles *in vacuo*, the titled compound **AuL9** (230 mg, 228 mmol, 47%) is obtained as a white solid.

^{1}H-NMR (400 MHz, CD_3CN) δ (ppm) = 7.47 (s, 8H, C*H*), 4.89-4.77 (m, 8H, C*H*$_2$), 4.76-4.66 (m, 8H, C*H*$_2$).

^{13}C-NMR (101 MHz, CD_3CN) δ (ppm) = 146.03 (N-*C*H-N), 125.92 (H*C*=*C*H), 48.58 (*C*H$_2$-*C*H$_2$).

Elemental analysis for $C_{20}H_{24}AuF_{18}N_8P_3$ * 0.1 MeCN (%) anal. calc.: C 24.62; H 2.70; N 11.11; S 0.00 found: C 24.82; H 2.78; N 11.15; S 0.57.

HR-ESI-MS: m/z [**AuL9** – 3 PF_6^-]$^{3+}$ calc: 191.0591, found: 191.0587, [**AuL9** – PF_6^-]$^+$ calc: 863.1068, found: 863.1038.

Pt[(cC^{Et}CC^{Et}C)OTf] (PtL8)

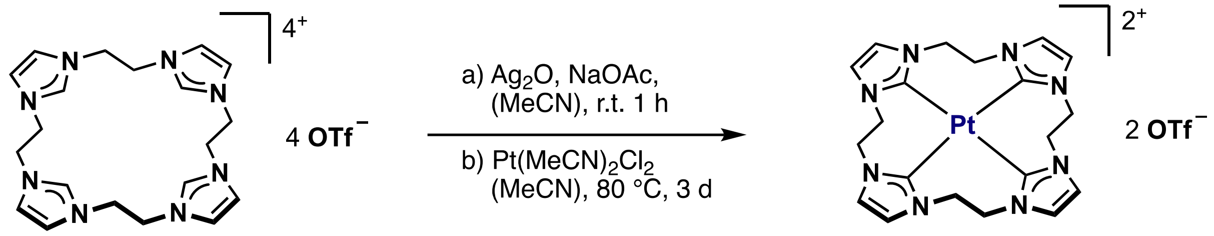

H$_4$L8 **PtL8**

Ag_2O (209 mg, 900 µmol, 2.20 eq.) is added to a solution of **H$_4$L8** (400 mg, 410 µmol, 1.00 eq.) and NaOAc (202 mg, 2.46 mmol, 4.00 eq.) in dry MeCN (30 mL) and stirred for 1 h at ambient temperature, followed by the addition of $Pt(MeCN)_2Cl_2$ (156 mg, 450 µmol, 1.10 eq.). The resulting reaction mixture is heated to 80 °C for 3 d and is filtered, after cooling to ambient temperature, over a short plug of basic aluminum oxide. The filter column is eluted with MeCN (50 mL) and all volatile compounds are removed *in vacuo*. The resulting crude material is resuspended in MeCN (5 mL) and centrifuged. Upon the addition of Et_2O (20 mL) to the supernatant, a white solid is precipitated. The crude material is collected *via* centrifugation, washed with Et_2O (3 × 5 mL) and redissolved in MeCN (5 mL). After the precipitation with Et_2O (15 mL) and drying *in vacuo*, the titled compound **PtL8** is obtained as an off-white solid (90 mg, 103 µmol, 25%).

^{1}H-NMR (400 MHz, CD_3CN) δ (ppm) = 7.19 (s, 8H, C*H*), 5.11-4.94 (m, 8H, C*H*$_2$), 4.50-4.38 (m, 8H, C*H*$_2$).

^{1}H-NMR (400 MHz, DMSO-d$_6$) δ (ppm) = 7.49 (s, 8H, C*H*), 5.06-4.97 (m, 8H, C*H*$_2$), 4.54-4.44 (m, 8H, C*H*$_2$).

^{13}C-NMR (101 MHz, CD_3CN) δ (ppm) = 159.39 (N-*C*H-N), 123.58 (H*C*=*C*H), 48.86 (*C*H$_2$-*C*H$_2$).

^{19}F-NMR (376 MHz, CD_3CN) δ (ppm) = -79.27 (C*F*$_3$)

HR-ESI-MS: m/z [**PtL8** – 2 OTf$^-$]$^{2+}$ calc: 285.5881, found: 285.5864, [**PtL8** – OTf$^-$]$^+$ calc: 720.1287, found: 720.1264.

2. Synthetic Approach to Calix[4]imidazolinium hexafluorophosphate

The synthesis of the saturated tetracarbene ligand **i** was designed analog to its unsaturated counterpart **a** (Figure 1).[2, 7] First, the *N*-heterocycle, 2-imidazoline (**10**), is generated, followed by the coupling of two equivalents of **10** to form bis(2-imidazolin-1-yl)methane (**11**), which subsequently can be used for the ring closure to give **i**.

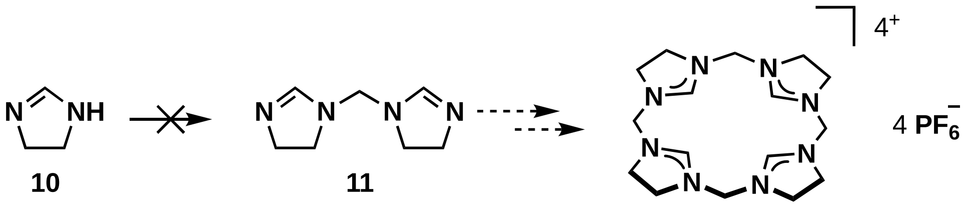

Figure 1. Proposed pathway for the synthesis of **i** starting from **10**.

The common way to produce imidazolines by reaction of ethylenediamine with a monocarboxylic acid fails when using formic acid, which would be required in the case of **10**.[8-10] Thus, **10** is synthesized according to the procedure leading to the larger six membered ring 1,4,5,6-tetrahydropyrimidine[11-12] by reaction of *N*,*N*-dimethylformamide dimethyl acetal and ethylenediamine to form the heterocycle (Figure 2). Various other, more difficult synthesis methods are described in the literature (see below).[8, 13-23]

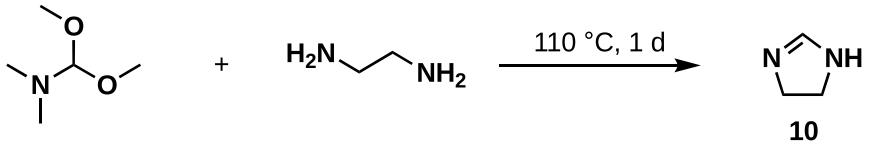

Figure 2. Synthesis of 2-imidazoline (**10**).

The synthesis of **11** (Figure 1) was attempted analog to **a** (see main text) in a mixture of DCM as coupling agent, concentrated NaOH as base and tertbutyl ammonium bromide (TBAB) as phase transfer catalyst.[24-25] However, no product formation was observed. Several other conditions with KOH as base, with and without solvent (DMF, MeOH, MeCN, DMSO) and different alkylating agents (DCM, CH_2Br_2, $CH_2(OTf)_2$) were tested without success (see below). A possible problem might be the hydrolysis of **10** to ethylenediamine: 2-imidazolines are known to be prone to hydrolysis under acidic and basic conditions.[26] It has been suggested that the hydrolysis is catalyzed by hydroxides. An amide is formed, which reacts to ethylenediamine and formic acid (Figure 3).[27] Electron donating substituents with +I effect such as tertiary, secondary or long primary alkyl groups can reduce the probability of hydrolysis at the C2 carbon atom and thus prolong the half-life of these compounds.[10, 27-28] The fully protonated analogs to 2-imidazolines, imidazolidines, are also often labile without substituents.[29] However,

no significant hydrolysis of **10** could be observed; in fact, unreacted **10** was found after reaction.

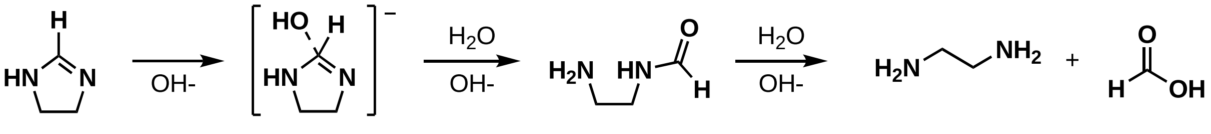

10

Figure 3. Proposed mechanism for the hydrolysis of 2-imidazoline (**10**) (adapted from[27]).

Nevertheless, application of protective groups was examined next to increase the stability of the heterocycle **10** before bridging to **11**, and for practical reasons in order to decrease the polarity of the respective compounds of **10** to increase the range of suitable organic solvents. Due to the basic nature of **10**, a benzyl group and Boc (*tert*-butyloxycarbonyl) group were selected – both being stable under basic conditions and being cleaved by catalytic hydrogenation (benzyl group)[30-32] or acid (Boc group)[33-34]. Direct formation of *N*-benzyl-2-imidazoline (**13**, Figure 4) from **10** with benzyl bromide did not give a selective reaction and bis(benzyl) substituted **10** was among the products (see SI). A similar reaction with a prior *in*-situ formation of the potassium 2-imidazolin-1-ide salt gave N^1,N^1,N^2,N^2-tetrabenzylethane-1,2-diamine (**15**, Figure 4) as ring-opening side product of **10**, underlining the lability of 2-imidazolines. Consequently, another approach to obtain **13** was pursued *via* the formation of *N*-benzylethylenediamine (**12**, Figure 4) and subsequent ring closure analog to the synthesis of **10**. A first synthesis with phenylmethanamine and 2-bromoethan-1-amine hydrobromide[35-36] was not successful but **12** could be obtained by reaction of benzyl chloride and ethylenediamine.[37-39] Ring closure of **12** was achieved by reaction with *N*,*N*-dimethylformamide dimethyl acetal based on a modified literature method[40-42] to give **13** (Figure 4). The benzyl substituted heterocycle **13** was coupled with CH_2Br_2 to form 3,3'-methylenebis(1-benzyl-2-imidazolinium) dibromide (**14**, Figure 4). However, selective removal of the benzyl groups by catalytic hydrogenation (5% Pd/C, 1 bar H_2) was not successful. **14** was mainly cleaved at the methylene bridge between the two 2-imidazoline rings without detection of **11** (see below). Attachment of the Boc group to **1** was possible through direct reaction of **10** with Boc_2O, and indirect reaction *via* protection of ethylenediamine with Boc_2O to form *tert*-butyl (2-aminoethyl)carbamate (**16**, modified procedure of [33, 43-44]), followed by the ring closure with *N*,*N*-dimethylformamide dimethyl acetal to obtain *tert*-butyl 2-imidazoline-1-carboxylate (**17**, Figure 4). However, alkylation of **17** with CH_2Br_2 was not successful.

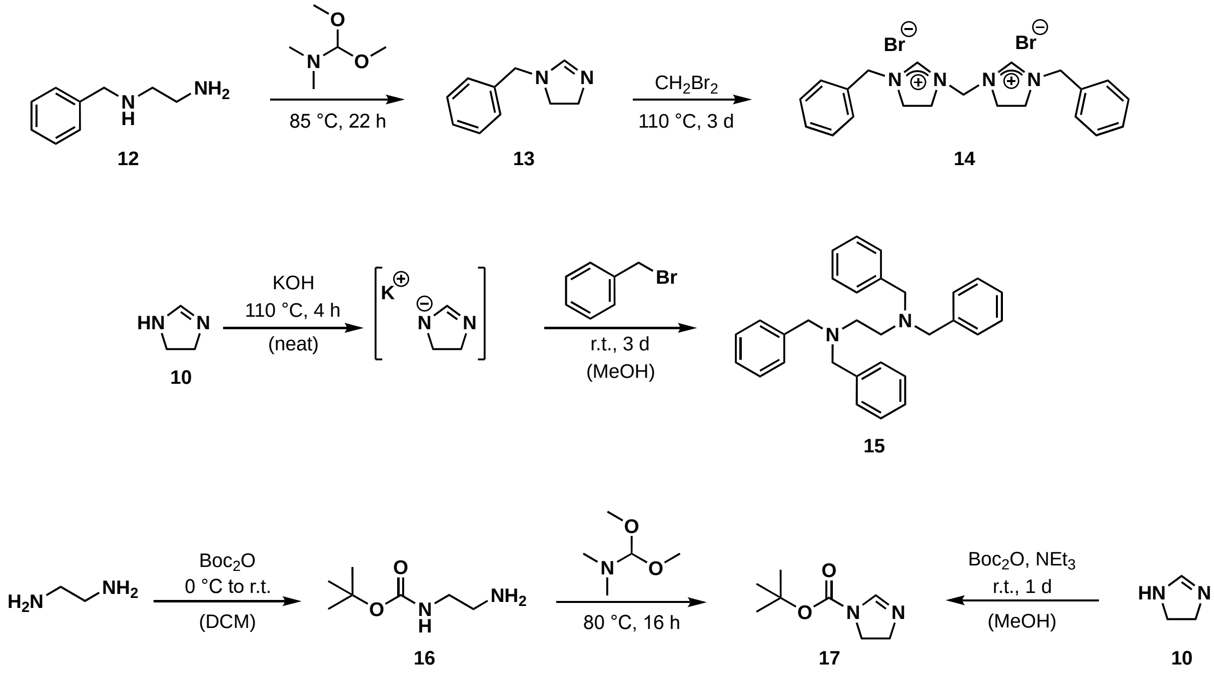

Figure 4. Synthetic studies towards **11** and macrocycle **i**. **12**: Benzyl chloride, ethylenediamine, 100 °C, 18 h.

Selective hydrogenation of imidazoles to imidazolines has been reported[45] but reaction of bis(imidazole-1-yl)methane up to 80 °C and 5 bar of hydrogen atmosphere using a Fisher-Porter tube with 5% Pd/C did not show any hydrogenation (see below). A series of Mannich type condensation reactions of **10** with formaldehyde (r.t., H_2O, analog to [46]) or paraformaldehyde (microwave, 120 °C, 200 W, THF, analog to [47-48]) did not give satisfactory results either. However, in another reaction with paraformaldehyde (*ACS* pressure tube, 85 °C, 16 h, MeOH), a product signal of **11** was present in the mass spectrum at m/z = 153.02 (calculated m/z [**11**+ H^+] = 153.11), but at low intensity and the majority of signals are unreacted starting material (**1**) and some side products (see below).

To avoid hydrolysis of **10**, other inorganic bases like NaH and K_2CO_3 and (steric hindered) organic bases like NEt_3, KO^{t}Bu, *N*,*N*-Diisopropylethylamine (DIPEA) and 1,8-bis(dimethylamino)naphthalene (“proton sponge”) were screened with DCM and CH_2Br_2 as alkylating agents in the original synthetic approaches with KOH (see below). The reactions using NEt_3 and DIPEA showed product signals of **11** in the mass spectra. However, the product could not be isolated as purification proved to be challenging (see below): after column chromatography, the batch from NEt_3 had peaks in NMR that were assignable to the product, but many impurities are also still present. The batch from DIPEA had a similar result after column chromatography and especially DIPEA hydrobromide salt and **10** are present. The product, the DIPEA hydrobromide salt and **10** all possess a similar high polarity and thus pose a difficult separation problem. Sublimation of the mixture was found as a possible method to remove the DIPEA hydrobromide salt from the crude. A possible method for purification in future studies might be

the sublimation of the crude product after reaction followed by column chromatography and subsequent conversion to form the tetracarbene precursor **i**. Impurities present at a low level should be acceptable in the ring closure of **i**. However, at this point the project was abandoned due to the more promising results achieved in the experiments regarding the macrocyclic ligands with ethylene bridges (**H_4L5/6,H_4L8/9**), and because satisfactory results could not be obtained in a reasonable time.

The new synthetic procedures of **10** to **17** were performed in normal atmosphere without dried and degassed chemicals and are based on the master's thesis of *T.P.S.*[10]

New synthetic procedures:

2-Imidazoline (10)

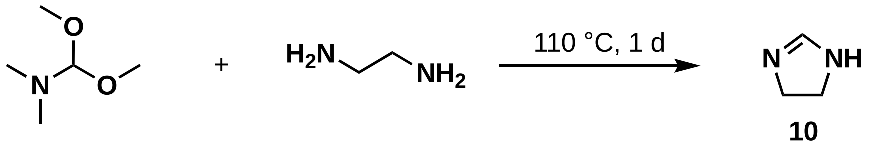

10

The synthesis of 2-imidazoline (**10**) is orientated on the formation of the larger six membered ring 1,4,5,6-tetrahydropyrimidine.[11-12] *N*,*N*-dimethylformamide dimethyl acetal (40.0 g, 44.7 mL, 336 mmol, 1.0 eq.) is added to ethylenediamine (20.2 g, 22.4 mL, 336 mmol, 1.0 eq.) and the mixture is stirred at 110 °C for 1 d. All volatiles are removed *in vacuo* resulting in a light yellow wax. A part of the raw product is sublimed from 50 °C to 90 °C at around 10^{-2} mbar to form colorless crystals. They are removed from the cooling finger in inert atmosphere to obtain **10** in a very hygroscopic white solid (7.19 g, 103 mmol, 54 % total yield based on the amount of received purified product). **10** forms into a glassy wax/ solid in contact to air moisture. ^{1}H-NMR analysis still shows the presence of an impurity, which could be assigned to dimethylformamide. With elemental analysis, the amount of dimethylformamide in the product is calculated to be around 3 %. The amine proton signal is barely visible in the ^{1}H-NMR spectrum at 7.44 ppm. The residual signals are still impurities after sublimation, assigned to DMF based on the signal at 7.98 ppm (C*H*)[49] and because DMF appears to be a usual byproduct when using *N*,*N*-dimethylformamide dimethyl acetal to form the imidazoline ring[36].

^{1}H-NMR (400 MHz, D_2O): δ (ppm) = 8.08 (s, 1H, C*H*), 7.44 (s, 1H, N*H*), 3.29 (t, 2H, NCH_2, ^{3}J = 6.2 Hz), 2.73 (t, 2H, NCH_2, ^{3}J = 6.2 Hz).

Anal. calcd. for $C_3H_6N_2$: C 51.41; H 8.63; N 39.97. Found: C 50.59; H 8.66; N 39.08

ESI-MS (m/z): [**10** +H$^+$] calcd., 71.06; found, 71.75 (100); [**10** +H_3O^+] calcd., 89.07; found, 88.78 (14).

2-imidazoline is a very hygroscopic white solid, making it difficult to handle. Therefore, in most syntheses in this work where 2-imidazoline is used, the crude product is applied without further purification. The main impurity is DMF, a common side product when using *N*,*N*-dimethylformamide dimethyl acetal.[36] However, DMF does not interfere in subsequent reactions due to its low reactivity and therefore constitutes an acceptable impurity.[10]

Alternative syntheses of 2-imidazoline in the literature include:[10]

- Reaction of *N*,*N'*-bis(trimethylsilyl)ethylenediamine with DMF: 2-imidazoline is formed after elimination of dimethylamine.[13-14] In the above showcased reaction of *N*,*N*-

dimethylformamide dimethyl acetal with ethylenediamine, after the cleavage of methanol, the elimination of dimethylamine is probably also the last step.

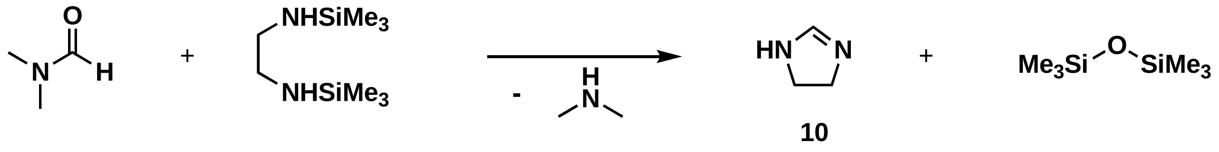

- oxidation of ethylenethiourea with dimethyldioxirane[15] or hydrogen peroxide[16-18]
- ring opening of *s*-triazine with ethlyenediamine[8, 19-20]
- ethylenediamine exchange from 1,4,5,6-tetrahydropyrimidine[21]
- synthesis from ethylenediamine with *tert*-butyl isocyanide using AgCN as catalyst[22-23].

Synthetic attempts: bis(2-imidazolin-1-yl)methane (11)[10]

In the following, the synthetic attempts to obtain bis(2-imidazolin-1-yl)methane (**11**) and its derivatives (Figure 5) on the synthetic pathway to **i** (Figure 6) are listed. Supporting analytics are given in Chapter 4.[10]

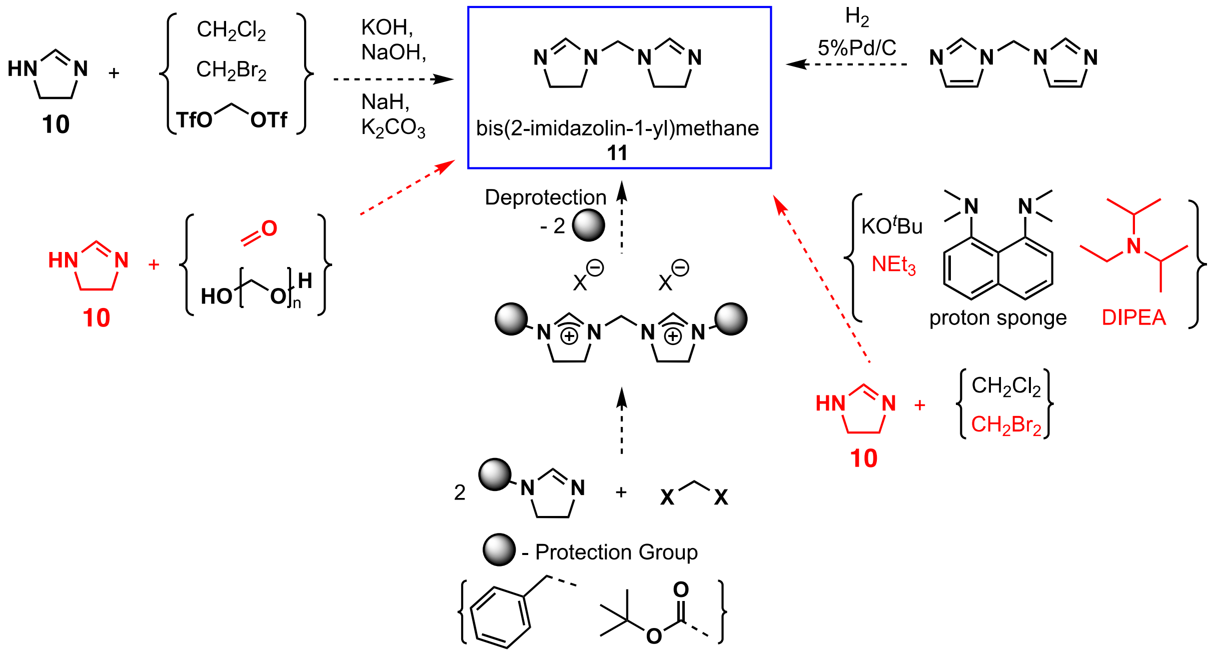

Figure 5. Overview of approaches to synthesize bis(2-imidazolin-1-yl)methane. Syntheses with detectable product marked red.[10]

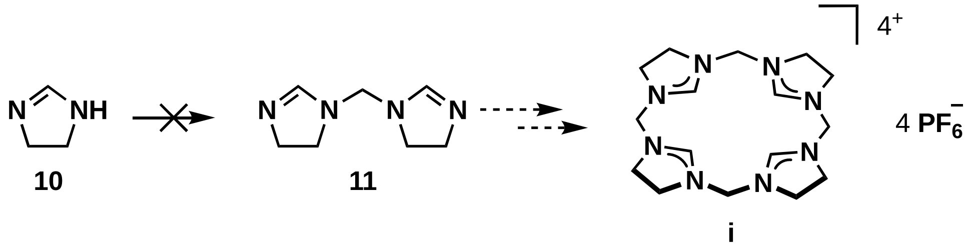

Figure 6. Proposed pathway for the synthesis of **i** starting from **10**.

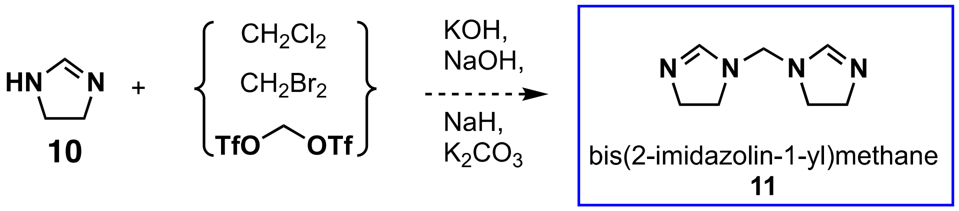

Alkylating agent/ electrophile	Base	Solvent	T [°C]	Reaction time [h]	Comment
DCM	NaOH	Conc. NaOH(aq)/ DCM	60	16	TBAB as phase catalyst[24-25]
CH_2Br_2	KOH	MeOH/ CH_2Br_2	60	4 d	TBAB as phase catalyst
CH_2Br_2	KOH	DMSO/ CH_2Br_2	70	3 d	
CH_2Br_2	KOH	DMF	90	16	Prio formation of the potassium 2-imidazolinide salt (KOH, 110 °C, 4 h, neat)
$CH_2(OTf)_2$	KOH	DMF	1) 0 to r.t. 2) 40	1) 16 2) 2	Prio formation of the potassium 2-imidazolinide salt (KOH, 110 °C, 4 h, neat)
CH_2Br_2	KOH	MeCN	95	17	oriented on a similar synthesis[50] hydrolysis of MeCN to acetamide detectable (Figure 67)[51]
DCM	KOH	MeCN	55	17	hydrolysis of MeCN to *N*-acetylamide detectable (Figure 68)[51]
CH_2Br_2	NaH (60%)	DMF	1) r.t. 2) 85	1) 5 d 2) 6	Dry conditions and **10** (sublimed, stored under Ar)
CH_2Br_2	K_2CO_3	- (neat)	130	4 d	*ACS* pressure tube, oriented on[52]

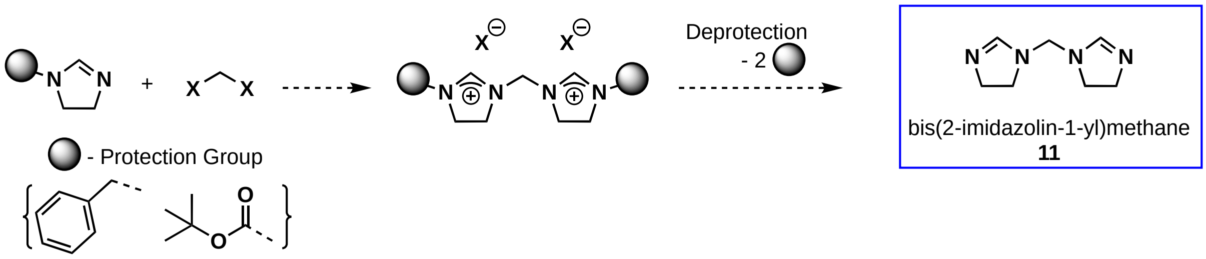

Alkylating agent/ electrophile	Base	Solvent	T [°C]	Reaction time [h]	Comment
Benzyl bromide	K_2CO_3	MeOH	75	40	Reaction with **10** oriented on [31]<br>Bis(benzyl) substituted **10** and **15** are present (Figure 69)
Benzyl bromide	KOH	MeOH	0 °C to r.t.	3 d	Reaction with potassium 2-imid-azolin-1-ide, synthesis of **15**
2-Bromoethan-1-amine hydrobromide	-	Toluene	120	18	Reaction with phenylmethana-mine, oriented on [35-36]<br>Unreacted phenylmethanamine after reaction (Figure 70)
Benzyl chloride	-	-	100	18	Reaction with ethylenediamine, synthesis of **12** [37-39]
N,*N*-Dimethyl-formamide di-methyl acetal	-	-	85	22	Reaction with **12**, synthesis of **13** (modified literature method[40-42])
CH_2Br_2	-	CH_2Br_2	110	3	Reaction with **13**, synthesis of **14**
-	-	MeOH	r.t.	20	Hydrogenation of **14**, 5%Pd/C<br>Bubbling H_2 for 4 h, rest H_2 at-mosphere (products see Figure 71)
di-*tert*-Butyl di-carbonate	-	DCM	0 °C to r.t.	1 d	Reaction with ethylenediamine, synthesis of **16** (modified litera-ture procedure[33, 43-44])

Alkylating agent/ electrophile	Base	Solvent	T [°C]	Reaction time [h]	Comment
N,*N*-Dimethyl-formamide di-methyl acetal	-	-	80	16	Reaction with **16**, synthesis of **17**
CH_2Br_2	-	CH_2Br_2	90	16	Conditions based on [53-54]
di-*tert*-Butyl di-carbonate	NEt_3	MeOH	r.t.	1 d	Reaction with **10**, synthesis of **17**
CH_2Br_2	-	CH_2Br_2	60	5	Reaction with **17**
CH_2Br_2	-	CH_2Br_2	80	18	Reaction with **17**

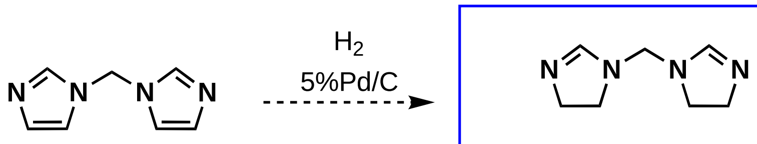

bis(2-imidazolin-1-yl)methane
11

Alkylating agent/ electrophile	Base	Solvent	T [°C]	Reaction time [h]	Comment
-	-	MeOH	60	3	Hydrogenation of bis(imidazole-1-yl)methane, 5%Pd/C H_2 Bubbling
-	-	MeOH	80	3 d	Hydrogenation of bis(imidazole-1-yl)methane, 5%Pd/C, 5 bar, Fisher-Porter tube

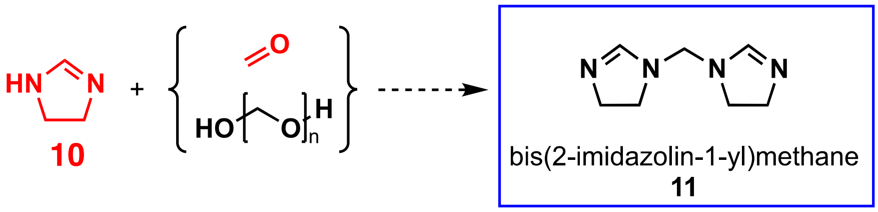

**Alkylating agent/ electrophile**	**Base**	**Solvent**	**T [°C]**	**Reaction time [h]**	**Comment**
Formaldehyde	-	H_2O	r.t.	3 d	pH ~ 4, conditions from[46]
Paraformalde-hyde	-	MeOH	85	16	*ACS* pressure tube<br>Product signal (**S1**, Figure 72) in ESI-MS
Paraformalde-hyde	-	THF	120	10 min	Analog to [47-48]<br>Microwave 200 W

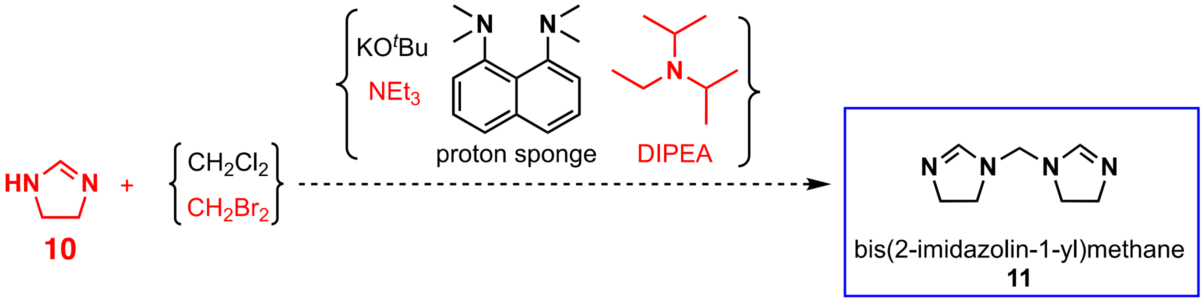

Alkylating agent/ electrophile	Base	Solvent	T [°C]	Reaction time [h]	Comment
CH_2Br_2	NEt_3	MeCN	r.t.	3 d	alkylated NEt_3 as side product (194.07 m/z)[55]
CH_2Br_2	NEt_3	MeCN	95	17	Batch from above reused, due to no conversion<br>Product signal (**S2**, Figure 73 to Figure 76)<br>alkylated NEt_3 as side product (194.07 m/z)[55]
CH_2Br_2	NEt_3	MeCN	95	3 d	Product signal (**S3**, Figure 77)<br>dry solvent<br>alkylated NEt_3 as side product (194.07 m/z)[55]
DCM	KO^{t}Bu	DCM	50	3 d	dry solvent
CH_2Br_2	KO^{t}Bu	MeCN	95	5 d	dry solvent<br>3-(imidazolin-1-yl)propanenitrile (123.95 m/z) as potential side product (Figure 78)[56]
CH_2Br_2	DIPEA	MeCN	95	4 d	Product signal (**S4**, Figure 79, Figure 80), dry solvent
CH_2Br_2	DIPEA	MeCN	95	3 d	dry solvent, crude sublimed *in vacuo* (4.1 · 10^{-2} mbar); from 150 °C to 190 °C DIPEA hydrobromide salt is collected (**S5**, Figure 81 to Figure 83)

Alkylating agent/ electrophile	Base	Solvent	T [°C]	Reaction time [h]	Comment
					Product signal (**S5**, Figure 84, Figure 85)
CH_2Br_2	Proton sponge	MeCN	95	6 d	dry solvent

The product **11** could not be isolated as purification proved to be challenging: After column chromatography, the batch from NEt_3 had peaks in NMR that were assignable to the product, but many impurities are also still present. The batch from DIPEA had a similar result after column chromatography and especially DIPEA hydrobromide salt and **10** are present. The product, the DIPEA hydrobromide salt and **10** all possess a similar high polarity and thus pose a difficult separation problem. Sublimation of the mixture was found as a possible method to remove the DIPEA hydrobromide salt from the crude. A possible method for purification in future studies might be the sublimation of the crude product after reaction followed by column chromatography and subsequent conversion to form the tetracarbene precursor **i**. Impurities present at a low level should be acceptable in the ring closure of **i**.

N-Benzylethylenediamine (12)[37-39]

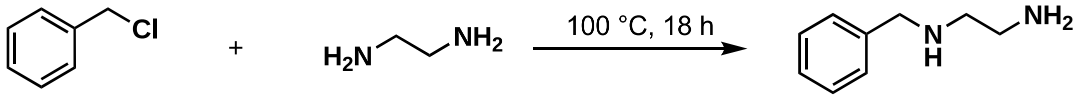

12

Benzyl chloride (23.1 mL, 25.4 g, 201 mmol, 1.0 eq.) is added to a cooled solution (0 °C) of ethylenediamine (67.0 mL, 60.3 g, 1.00 mol, 5.0 eq.). The ice bath is removed and the reaction mixture is stirred at 100 °C for 18 h to result in an orange solution. Then, 40 mL cooled NaOH (30 %) is added to the cooled reaction mixture to result in a white solid. The orange supernatant is decanted and extracted with Et_2O (4 × 20 mL). The combined organic layers are dried with anhydrous $MgSO_4$, filtered and concentrated under reduced pressure to result in a yellow oil. The yellow residue is purified by distillation under reduced pressure (5.2 × 10^{-2} mbar, 130 °C oil bath) to obtain **12** as a colorless liquid (15.6 g, 104 mmol, 52 %).

^{1}H-NMR (400 MHz, DMSO-*d_6*): δ (ppm) = 7.28 (m, 5H, C_6H_5), 3.68 (s, 2H, Ph–CH_2), 2.59 (t, 2H, NCH_2, ^3J = 6.2 Hz), 2.48 (m, 2H, NCH_2), 1.53 (bs, 3H, NH_2, N*H*). **^{1}H-NMR** (400 MHz, CD_3CN): δ (ppm) = 7.29 (m, 5H, C_6H_5), 3.74 (s, 2H, Ph–CH_2), 2.67 (t, 2H, NCH_2, ^3J = 6.0 Hz), 2.55 (m, 2H, NCH_2), 1.46 (bs, 3H, NH_2, N*H*).

^{1}H-NMR (400 MHz, $CDCl_3$): δ (ppm) = 7.23 (m, 5H, C_6H_5), 3.73 (s, 2H, Ph–CH_2), 2.74 (t, 2H, NCH_2, ^3J = 6.2 Hz), 2.62 (t, 2H, NCH_2, ^3J = 6.2 Hz), 1.57 (bs, 3H, NH_2, N*H*).

^{13}C-NMR (101 MHz, DMSO-*d_6*): δ (ppm) = 141.18 (C_{ar}–CH_2–NH), 128.01 (CH_{ar}), 127.83 (CH_{ar}), 126.37 (CH_{ar}), 52.96 (CH_2), 52.09 (CH_2), 41.50 (CH_2–CH_2–NH_2).

^{13}C-NMR (101 MHz, CD_3CN): δ (ppm) = 142.47 (C_{ar}–CH_2–NH), 129.17 (CH_{ar}), 128.97 (CH_{ar}), 127.52 (CH_{ar}), 54.26 (CH_2), 53.16 (CH_2), 42.68 (CH_2–CH_2–NH_2).

^{13}C-NMR (101 MHz, $CDCl_3$): δ (ppm) = 140.48 (C_{ar}–CH_2–NH), 128.45 (CH_{ar}), 128.16 (CH_{ar}), 126.98 (CH_{ar}), 53.91 (CH_2), 51.93 (CH_2), 41.76 (CH_2–CH_2–NH_2).

ESI-MS (m/z): [**12** +H^+] calcd., 151.12; found, 150.92 (100); [**12** –NH_2] calcd., 134.10; found, 133.94 (67); [**12** –$NHCH_2CH_2NH_2$] calcd., 91.05; found, 90.85 (77).

N-Benzyl-2-imidazoline (13)

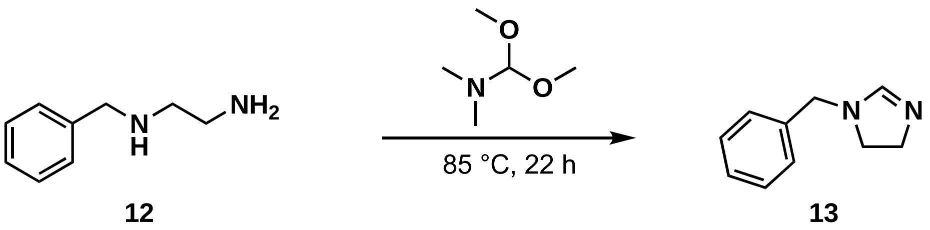

The synthesis of *N*-benzyl-2-imidazoline (**13**) is according to a modified literature procedure.[40-42] *N*,*N*-dimethylformamide dimethyl acetal (15.5 mL, 14.0 g, 117 mmol, 1.19 eq.) is added to **12** (14.8 g, 99 mmol, 1.00 eq.) and the mixture is stirred at 85 °C for 22 h. The volatiles are removed from the reaction mixture to result in a yellow liquid. The yellow residue is purified by distillation under reduced pressure (1.5 × 10^{-2} mbar, 145 °C oil bath, 89 °C thermometer) to obtain **13** as a light pink liquid (12.9 g, 81 mmol, 82 %).

^{1}H-NMR (400 MHz, $CDCl_3$): δ (ppm) = 7.28 (m, 5H, C_6H_5), 6.95 (s, 1H, C*H*), 4.26 (s, 2H, Ph–CH_2), 3.81 (tt, 2H, CH_2–CH_2–N–CH, ^3J = 9.9 Hz, ^4J = 1.9 Hz), 3.11 (td, 2H, Ph–CH_2–N–CH_2, ^3J = 9.9 Hz, ^4J = 2.5 Hz).

^{1}H-NMR (400 MHz, CD_3CN): δ (ppm) = 7.32 (m, 5H, C_6H_5), 6.91 (t, 1H, C*H*, ^4J = 1.8 Hz), 4.27 (s, 2H, Ph–CH_2), 3.66 (td, 2H, CH_2–CH_2–N–CH, ^3J = 9.7, ^4J = 1.8 Hz), 3.02 (t, 2H, Ph–CH_2–N–CH_2, ^3J = 9.7 Hz).

^{1}H-NMR (400 MHz, DMSO-*d*$_6$): δ (ppm) = 7.31 (m, 5H, C_6H_5), 7.09 (t, 1H, C*H*, ^4J = 1.8 Hz), 4.29 (s, 2H, Ph–CH_2), 3.60 (td, 2H, CH_2–CH_2–N–CH, ^3J = 9.8, ^4J = 1.8 Hz), 2.97 (t, 2H, Ph–CH_2–N–CH_2, ^3J = 9.8 Hz).

^{13}C-NMR (101 MHz, $CDCl_3$): δ (ppm) = 157.51 (*C*H), 137.06 (C_{ar}–CH_2–N), 128.73 (*C*H_{ar}), 127.79 (*C*H_{ar}), 127.65 (*C*H_{ar}), 55.23 (C_{ar}–CH_2–N), 51.78 (*C*H_2), 48.21 (*C*H_2).

^{13}C-NMR (101 MHz, CD_3CN): δ (ppm) = 158.19 (*C*H), 139.00 (C_{ar}–CH_2–N), 129.53 (*C*H_{ar}), 128.91 (*C*H_{ar}), 128.30 (*C*H_{ar}), 55.91 (C_{ar}–CH_2–N), 52.24 (*C*H_2), 48.81 (*C*H_2).

^{13}C-NMR (101 MHz, DMSO-*d*$_6$): δ (ppm) = 157.36 (*C*H), 137.71 (C_{ar}–CH_2–N), 128.45 (*C*H_{ar}), 127.82 (*C*H_{ar}), 127.18 (*C*H_{ar}), 54.65 (C_{ar}–CH_2–N), 50.68 (*C*H_2), 47.50 (*C*H_2).

Anal. calcd. for $C_{10}H_{12}N_2$: C 74.97; H 7.55; N 17.48. Found C 74.61; H 7.72; N 17.55.

ESI-MS (m/z): [**13** +H^+] calcd., 161.11; found, 160.98 (100); [**13** –(2-imidazoline)] calcd., 91.05; found, 90.86 (35).

3,3'-Methylenebis(1-benzyl-2-imidazolinium) dibromide (14)

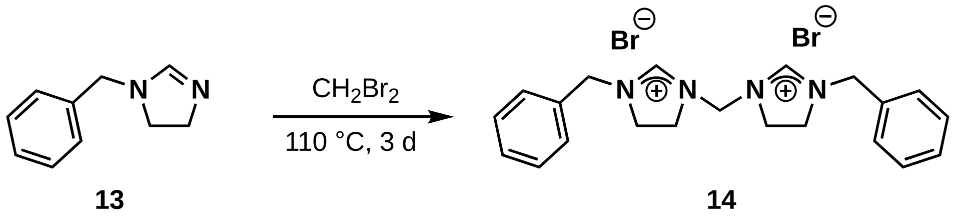

13 (2.00 g, 12.5 mmol, 2.00 eq.) is added to a solution of dibromomethane (10 mL, 24.9 g, 143 mmol, 23.0 eq.) and the mixture is stirred at 110 °C for 3 d. Et_2O (3 mL) is added to the dark orange/ brown solution, resulting in a brown precipitate, which is filtrated. To the brown residue, DCM (25 mL) is added and the suspension is stirred and heated to around 50 °C. The hot suspension is filtrated and the residue is washed with DCM (30 mL) resulting in **14** as an off-white solid (1.35 g, 2.73 mmol, 44 %).

^{1}H-NMR (400 MHz, DMSO-d_6): δ (ppm) = 8.89 (s, 2H, C*H*), 7.44 (m, 10H, C_6H_5), 5.22 (s, 2H, N–CH_2–N), 4.75 (s, 4H, Ph–CH_2), 3.95 (m, 4H, CH_2–CH_2–N–CH), 3.83 (m, 4H, Ph–CH_2–N–CH_2).**Anal. calcd**. for $C_{21}H_{26}Br_2N_4$: C 51.03; H 5.30; N 11.34. Found C 50.54; H 5.35; N 11.08.

ESI-MS (m/z): [**14** –Br^-] calcd., 413.13; found, 412.84 (34); [**14** –H^+ –$2Br^-$] calcd., 333.21; found, 333.20 (100); [**14** –CH_2Ph –$2Br^-$] calcd., 243.16; found, 243.03 (71); [**14** +H^+ –CH_2 –**13** –$2Br^-$] calcd., 161.11; found, 161.06 (29); [CH_2Ph] calcd., 91.05; found, 90.83 (85).

*N*1,*N*1,*N*2,*N*2-Tetrabenzylethane-1,2-diamine (15)

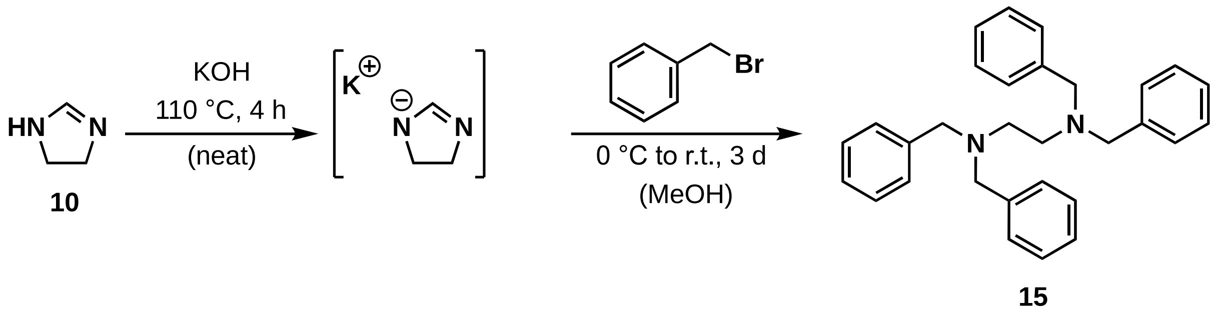

KOH (4.01 g, 71.5 mmol, 1.06 eq.) and **10** (4.74 g, 67.6 mmol, 1.00 eq.) are stirred at 110 °C with a drying tube on top of the flask, which is flushed several times with argon to remove the water vapor. After around 2 h, the potassium 2-imidazolin-1-ide salt begins to appear. After 4 h, the flask is cooled to r.t. and MeOH (100 mL) is added. Then, benzyl bromide (8.50 mL, 12.2 g, 71.6 mmol, 1.06 eq.) is added dropwise over a time of 20 min to the cooled mixture (0 °C) while stirring. The ice bath is removed and the mixture is stirred for 3 d at r.t. to result in a yellow suspension with a white solid. The mixture is filtrated, the volatiles are removed *in vacuo*, giving a yellow oil. The yellow oil is suspended in DCM (50 mL), washed with H_2O (4 × 50 mL) and the organic layer is dried *in vacuo*, yielding yellow crystals. The crystals are suspended in MeCN (5 mL), filtrated and washed with MeCN (3 × 3 mL), to obtain **15** as white crystals (around 0.63 g, yield ~8 %; this synthesis was originally designed to yield *N*-benzyl-2-imidazoline (**13**), hence no exact yield was measured).

^{1}H-NMR (400 MHz, CD_3CN): δ (ppm) = 7.27 (m, 20H, C_6H_5), 3.48 (s, 8H, Ph–CH_2), 2.55 (s, 4H, N–CH_2).

The signals in the ^{1}H-NMR spectrum are similar to the literature.[57]

Anal. calcd. for $C_{30}H_{32}N_2$: C 85.67; H 7.67; N 6.66. Found: C 85.55; H 7.62; N 6.60.

ESI-MS (m/z): [**15** +H$^+$] calcd., 421.26; found, 421.20 (100); [**15** –CH_2Ph +H +H$^+$] calcd., 331.22; found, 331.28 (44); [CH_2Ph] calcd., 91.05, found, 90.84 (11).

tert-Butyl (2-aminoethyl)carbamate (16)

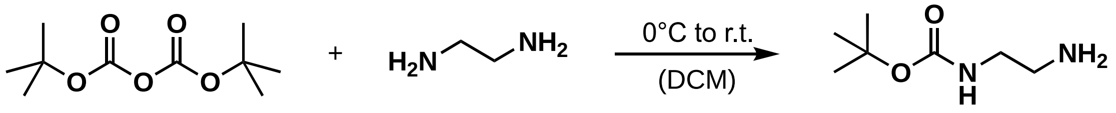

16

The synthesis of *tert*-butyl (2-aminoethyl)carbamate (**16**) is according to a modified literature procedure.[33, 43-44] Ethane-1,2-diamine (28 mL, 25.2 g, 419 mmol, 10.5 eq.) is dissolved in DCM (400 mL) in a 2 L round bottom flask and cooled with an ice bath (0 °C). Then, di-*tert*-butyl dicarbonate (8.70 g, 39.9 mmol, 1.00 eq.) dissolved in DCM (200 mL) is added dropwise to the cooled solution during 3 h while stirring. After stirring for additional 21 h at r.t., the reaction mixture is washed with H_2O (6 × 100 mL) and brine (100 mL). The organic phase is dried over anhydrous $MgSO_4$, filtered and concentrated under reduced pressure to obtain **16** as a slightly yellow oil (3.54 g, 22.1 mmol, 55 %). Since the yield is described as quantitative in the literature[33, 43-44], the used old di-*tert*-butyl dicarbonate was probably already decomposed to a certain amount. Indeed, signals of *tert*-butyl alcohol can be seen in the ^{1}H- and ^{13}C-NMR spectra as impurity.

^{1}H-NMR (400 MHz, $CDCl_3$): δ (ppm) = 4.93 (bs, 1H, N*H*), 3.16 (q, 2H, NHC*H*$_2$CH$_2$, ^3J = 5.9 Hz), 2.79 (t, 2H, C*H*$_2$NH$_2$, ^3J = 5.9 Hz), 1.57 (s, 2H, N*H*$_2$), 1.43 (s, 9H, C(C*H*$_3$)$_3$).

^{1}H-NMR (400 MHz, CD_3CN): δ (ppm) = 5.60 (bs, 1H, N*H*), 3.01 (q, 2H, NHC*H*$_2$CH$_2$, ^3J = 6.1 Hz), 2.63 (t, 2H, C*H*$_2$NH$_2$, ^3J = 6.1 Hz), 1.49 (s, 2H, N*H*$_2$), 1.39 (s, 9H, C(C*H*$_3$)$_3$).

^{13}C-NMR (101 MHz, $CDCl_3$): δ (ppm) = 156.26 (O*C*(O)NH), 78.93 (*C*(CH$_3$)$_3$), 43.31 (*C*H$_2$), 41.77 (*C*H$_2$), 28.33 (C(*C*H$_3$)$_3$).

ESI-MS (m/z): [**16** +H$^+$] calcd., 161.13; found, 160.79 (18); [**16** –C(CH$_3$)$_3$ +H$^+$] calcd., 104.06; found, 104.83 (100); [**16** –OC(CH$_3$)$_3$ +H$^+$] calcd., 88.06; found, 87.83 (95).

tert-Butyl 2-imidazoline-1-carboxylate (17) – Method A

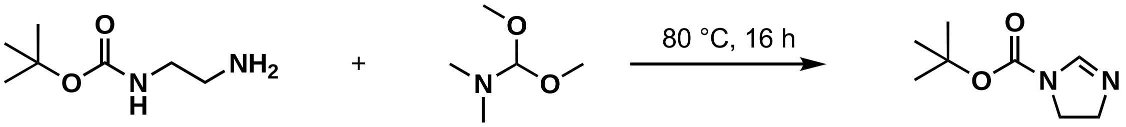

16 **17**

16[1] (1.01 g, 6.3 mmol, 1.00 eq.) and *N*,*N*-dimethylformamide dimethyl acetal (0.95 mL, 0.86 g, 7.2 mmol, 1.14 eq.) were stirred at 80 °C for 16 h. Then, the volatiles are removed *in vacuo*. The yellow oil is purified using column chromatography (2 % MeOH in DCM, 1 L, R_{f1} = 0.27, R_{f2} = 0.22) to result in a slightly yellow oil (40 mg, 0.2 mmol, 4 %). DMF is present as impurity based on the signal at 7.93 ppm (C*H*)[49]. It is a usual byproduct when using *N*,*N*-dimethylformamide dimethyl acetal to form the imidazoline ring[36].

^{1}H-NMR (400 MHz, CD_3CN): δ (ppm) = 7.40 (t, 1H, C*H*, ^4J = 1.8 Hz), 3.85 (td, 2H, CH_2, ^3J = 9.4 Hz, ^4J = 2.2 Hz), 3.53 (m, 2H, CH_2), 1.47 (s, 9H).

^{13}C-NMR (101 MHz, CD_3CN): δ (ppm) = 163.29 (O*C*(O)N), 149.42 (*C*H), 82.23 (*C*(CH_3)$_3$), 43.94 (*C*H_2), 43.86 (*C*H_2), 28.37 (C(*C*H_3)$_3$).

[1] For this synthesis commercially available *tert*-butyl (2-aminoethyl)carbamate **16** was used.

tert-Butyl 2-imidazoline-1-carboxylate (17) – Method B

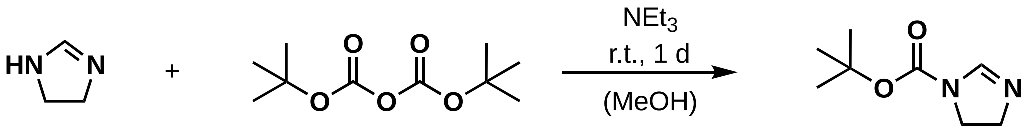

10 **17**

The synthesis of *tert*-butyl 2-imidazoline-1-carboxylate (**17**) is orientated on the synthesis of *tert*-butyl 5,6-dihydropyrimidine-1(4*H*)-carboxylate.[34] To a stirred solution of **10** (2.84 g, 40.5 mmol, 1.00 eq.) in MeOH (70 mL), triethylamine (28 mL, 20.4 g, 202 mmol, 4.99 eq.) is added under cooling in an ice bath (0 °C). Then, di-*tert*-butyl dicarbonate (17.7 g, 81.1 mmol, 2.00 eq.) is added to the cooled solution. The ice bath is removed and the reaction mixture is stirred at r.t. for 1 d. Next, the solvent is removed *in vacuo* and the slightly yellow residue is purified by column chromatography (2 % MeOH in DCM, 400 mL, R_{f1} = 0.10, R_{f2} = 0.41 – 0.21; 4 % MeOH in DCM, 400 mL, R_{f1} = 0.11, R_{f2} = 0.50 – 0.17) to result in a colorless oil (7.27 g raw product). About 1 g of raw product is sublimed at 55 °C at $4 \cdot 10^{-2}$ mbar to obtain **17** as colorless crystals around the cooling finger. At the tip of the cooling finger, a colorless oil is obtained, which is slightly impure product **17**.

^{1}H-NMR (400 MHz, DMSO-d_6): δ (ppm) = 7.47 (s, 1H, C*H*), 3.82 (td, 2H, CH_2, ^3J = 9.2 Hz, ^4J = 1.5 Hz), 3.52 (dd, 2H, CH_2, ^3J = 9.2 Hz, ^4J = 1.2 Hz), 1.45 (s, 9H, C(CH_3)$_3$).

3. Biological Studies

Substances

The substances **PdL3**, **PdL8** and **AuL9** were present as pure, crystalline materials, and were dissolved in DMSO (Serva, Heidelberg, Germany) as 40 mM stock solutions.

The conventional cytostatic drug *cis*-diamminedichloridoplatinum(II) (cisplatin) was provided by the pharmacy of *Helios Clinics Schwerin*.

Cell lines and cell cultivation

Human B cell precursor leukemia cell line (Nalm-6 cells) were kindly provided by Dr. Seeger (AG Henze, *Charité Berlin*, Germany) and human neuroblastoma cell line (SK-N-AS cells) were kindly provided by Prof. Dr. T. Simon (*University Cologne*). SK-N-AS cisplatin resistance cells were generated by Prof. Aram Prokop's group by treating SK-N-AS cells with increasing concentrations of cisplatin. Compared to the origin cell line, the resistant cells tolerate significant higher concentrations of cisplatin (up to 8.25 µM) without the loss of viability.

All cell lines were cultured in RPMI 1640 medium (GIBCO Invitrogen, Karlsruhe, Germany) supplemented with heat inactivated fetal bovine serum (FBS, 10% (v/v), GIBCO Invitrogen, Karlsruhe, Germany) and penicillin streptomycin (1% (v/v), GIBCO Invitrogen, Karlsruhe, Germany) at 37 °C and 5 % CO_2. The cells were subcultured every 3-4 d to a concentration of 0.5×10^5 cells/mL.

To ensure uniform growth conditions in the suspension cell line Nalm-6, the cells were brought to a concentration of 3×10^5 cells/mL 24 h prior to an experiment. On the day of the experiment, 2×10^5 Nalm-6 cells were seeded out per well in 12-well plates and incubated with the test substances immediately.

The adherent SK-N-AS cells and SK-N-AS cisplatin resistant cells were trypsinated for 3 min and 1.5×10^5 cell per well were seeded out in 6-well plates 24 h prior to the incubation with the test substances.

The incubation time of the test substances was dependent on the subsequent analysis and is described in the following sections. A DMSO control with a DMSO volume equivalent to the highest concentration of the corresponding test substances was always included and did not exceed 0.5 %.

Determination of cell concentration and cell viability

The cell concentrations as well as the cell viability were determined by using the CASY Cell-Counter and Analyzer System (OMNI Life Science GmbH, Bremen, Germany). The cells were analyzed in a defined setup according to their respective characteristics and differentiated in a single measurement between cell debris, dead cells and viable cells.

For the determination of cell proliferation, the cells were incubated with the test substances in different concentrations for 48 h. After 48 h, cells were trypsinated (in case of adherent cells) and properly resuspended. 100 µL of cell suspension of each sample was diluted in 10 mL CASYton (ready-to-use-isotonic saline solution, OMNI Life Science GmbH, Bremen, Germany) and the cell viability and concentration was measured. The proliferation of cells in the test samples was indicated relative to the DMSO control (100 %).

DNA Fragmentation

Apoptotic cell death was determined by a modified cell cycle analysis, which detects DNA fragmentation on the single cell level.[58]

The cells were treated with the respective substances (**PdL3**, **PdL8**, **AuL9**) in different concentrations and incubated for 72 h or 96 h. The cells were collected via centrifugation (8000 rpm, 4 °C, 5 min) and fixed on ice with formaldehyde (2 % (v/v) in 1x PBS) for 30 min. After incubation, the cells were centrifuged (1500 rpm, 4 °C, 5 min), the supernatant was discarded and the cells were resuspended and incubated with 1 × PBS overlaid with ethanol (1:2 (v/v)) on ice for 15 min. To eliminate RNA from the samples, the cells were centrifuged (1500 rpm, 4 °C, 5 min), the supernatant was removed, and the samples were resuspended and incubated with RNase A (40 µg/mL in 1x PBS; VWR, Darmstadt, Germany) for 30 min at 37 °C. The cells were centrifuged (1500 rpm, 4 °C, 5 min), the supernatant was discarded, the pellet was resuspended in propidium iodide (50 µg/mL in 1× PBS; Serva, Heidelberg, Germany). Nuclear DNA fragmentation was quantified and analyzed using a flow cytometry analysis by FACSLyric (Becton-Dickinson, Heidelberg, Germany) equipped with FASC Suit v.1.5 software. Data are given in percent of hypodiploidy (subG1), which reflects the number of apoptotic cells. The induced apoptosis was determined by subtracting the background apoptosis measured in the DMSO control cells from the measured apoptosis of the treated cells.

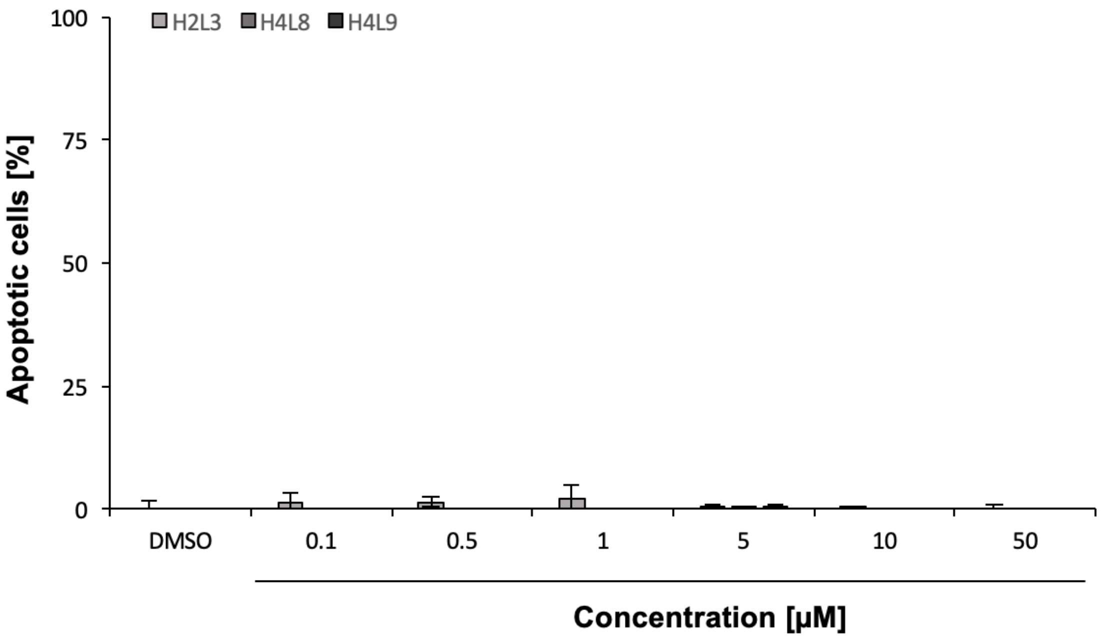

Figure 7. Nalm-6 cells were treated with different concentrations of **H_2L3**, **H_4L8** and **H_4L9** and incubated for 72 h. There was no significant induction of apoptosis. Nuclear DNA fragmentation was analyzed by flow cytometric analysis. Values are mean % of apoptotic cells ± SD (n = 3), error bars might not be visible due to low mean and SD values; * : $p < 0.05$ vs. DMSO, t-test.

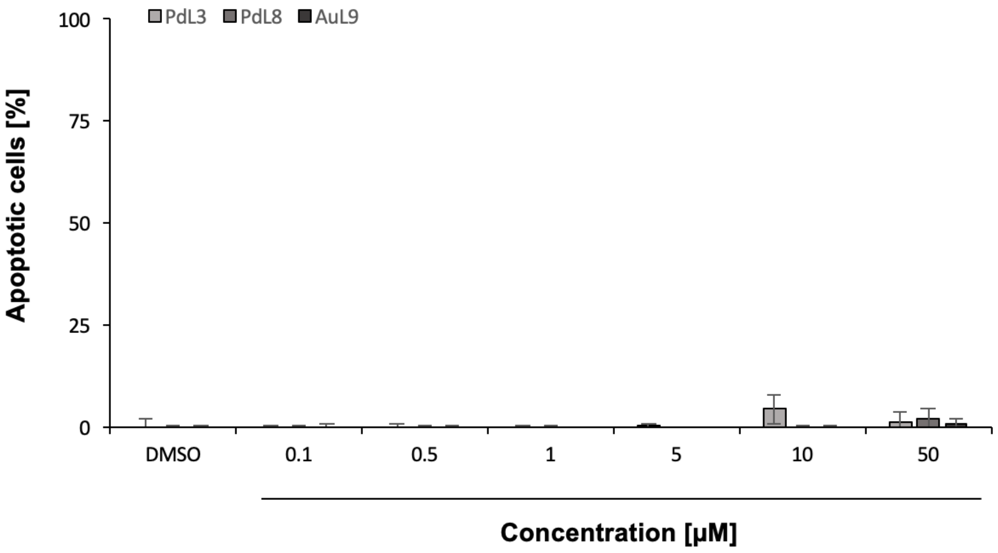

Figure 8. Nalm-6 cells were treated with different concentrations of **PdL3**, **PdL8** and **AuL9** and incubated for 72 h. There was no significant induction of apoptosis. Nuclear DNA fragmentation was analyzed by flow cytometric analysis. Values are mean % of apoptotic cells ± SD (n = 3), error bars might not be visible due to low mean and SD values; *: $p < 0.05$ vs. DMSO, t-test.

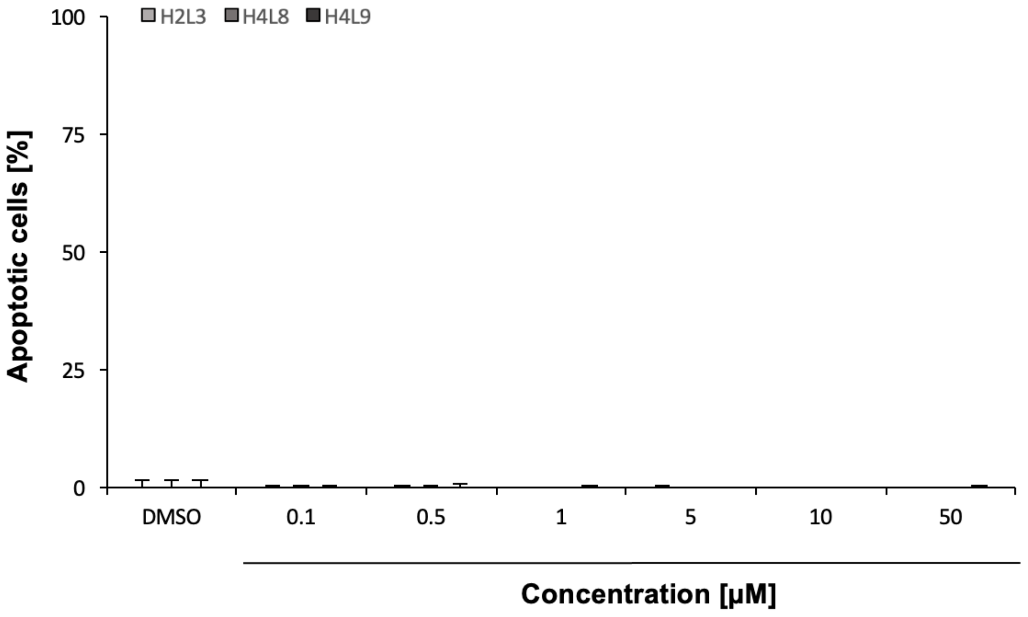

Figure 9. SK-N-AS cells were treated with different concentrations of **H_2L3**, **H_4L8** and **H_4L9** and incubated for 72 h. There was no significant induction of apoptosis. Nuclear DNA fragmentation was analyzed by flow cytometric analysis. Values are mean % of apoptotic cells ± SD (n = 3), error bars might not be visible due to low mean and SD values; * : $p < 0.05$ vs. DMSO, t-test.

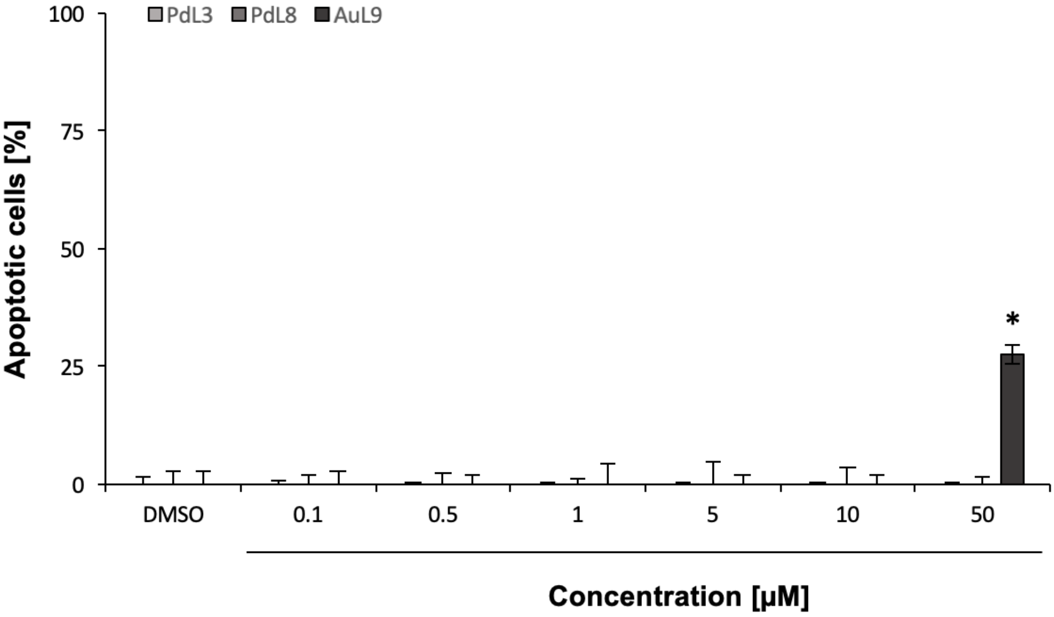

Figure 10. Only **AuL9** induces significantly apoptosis in SK-N-AS cells. The cells were treated with different concentrations of **PdL3**, **PdL8** and **AuL9** and incubated for 72 h. Nuclear DNA fragmentation was analyzed by flow cytometric analysis. Values are mean % of apoptotic cells ± SD (n = 3), error bars might not be visible due to low mean and SD values; * : $p < 0.05$ vs. DMSO, t-test.

LDH Release Assay

The measurement of lactate dehydrogenase (LDH) in the medium, which leaks out of the cells when cell integrity is lost, is a reasonable way to rule out necrosis.[59]

The Cytotoxicity Detection Kit (Roche, Mannheim, Germany) was used, and the experiment was performed according to the manual. For the detection of LDH, the cells were incubated for 2 h with the test substances. The medium was changed just before adding the test substances to remove the LDH released overnight. A positive control treated with Triton-X 100 (0.1 %, Sigma Aldrich, St. Louis, MO, USA) was carried out and was defined as 100 % cell necrosis. The samples were related to this.

Measurement of the mitochondrial transmembrane potential

The mitochondrial permeability transition was determined by staining the cells with 5,5′, 6,6′-tetrachloro-1,1′,3,3′-tetraethylbenzimidazolylcarbo-cyanine iodide (JC-1; Molecular Probes, Leiden, the Netherlands). The cells were treated with the test substances for 48 h. After the incubation, the cells were collected by trypsination and centrifugation (3000 rpm, 4 °C, 5 min). The pellets were resuspended in phenol red-free RPMI 1640 medium (GIBCO Invitrogen, Karlsruhe, Germany) without supplements and JC-1 was added to a final concentration of 2.5 µg/mL (except the negative control). The samples were briefly vortexed and incubated at 37 °C for 30 min in a thermal mixer with continuous shaking (300 rpm). After the incubation period, the cells were centrifuged (4000 rpm, 4 °C, 5 min) and the pellet was resuspended in 1 × PBS. The mitochondrial transmembrane potential was analyzed by flow cytometry using FACSLyric and the FASC Suit v.1.5 software.

Statistical analysis

Experiments were performed in triplicates and the mean and standard deviation (SD) were calculated. The SD is shown as error bars in the figures, some error bars might not be visible due to low mean and SD values. The significance of differences in the comparison of data was calculated using a two-tailed t-test, and significance was assumed at $p < 0.05$. All results are given as means ± SD, and significant differences have been indicated with an asterisk (*). Microsoft Office Excel was used to generate graphs and statistics.

4. Analytic data

1,1-Ethylene-di-2-imidazoline (1)

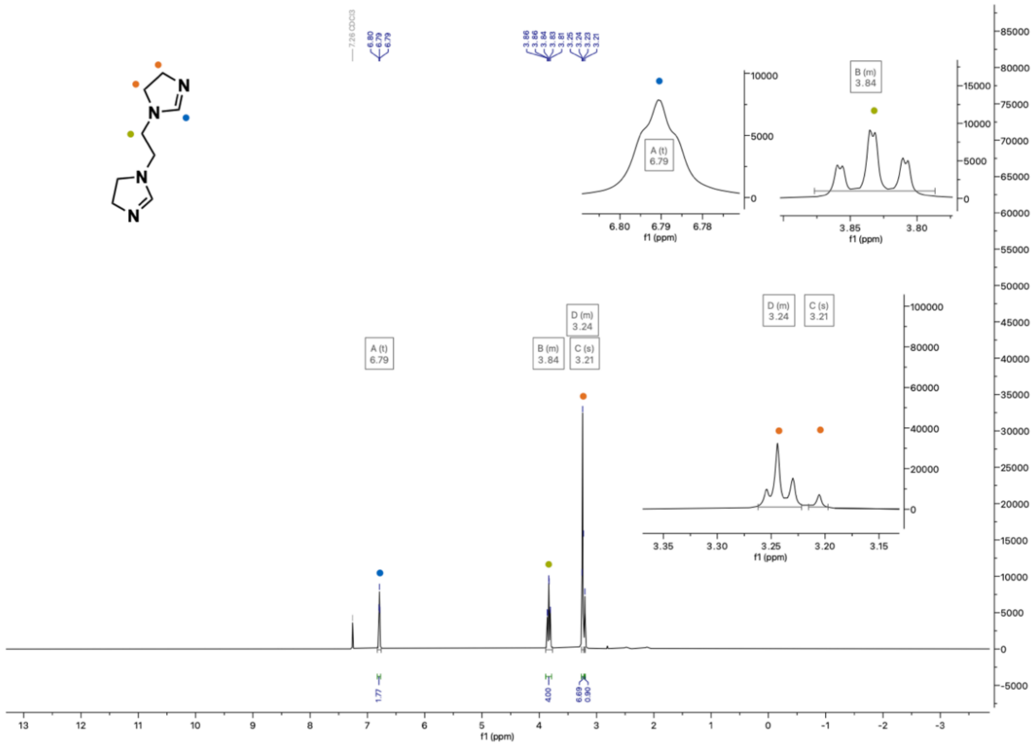

Figure 11: ^{1}H-NMR spectrum of **1** in C*D*Cl$_3$.

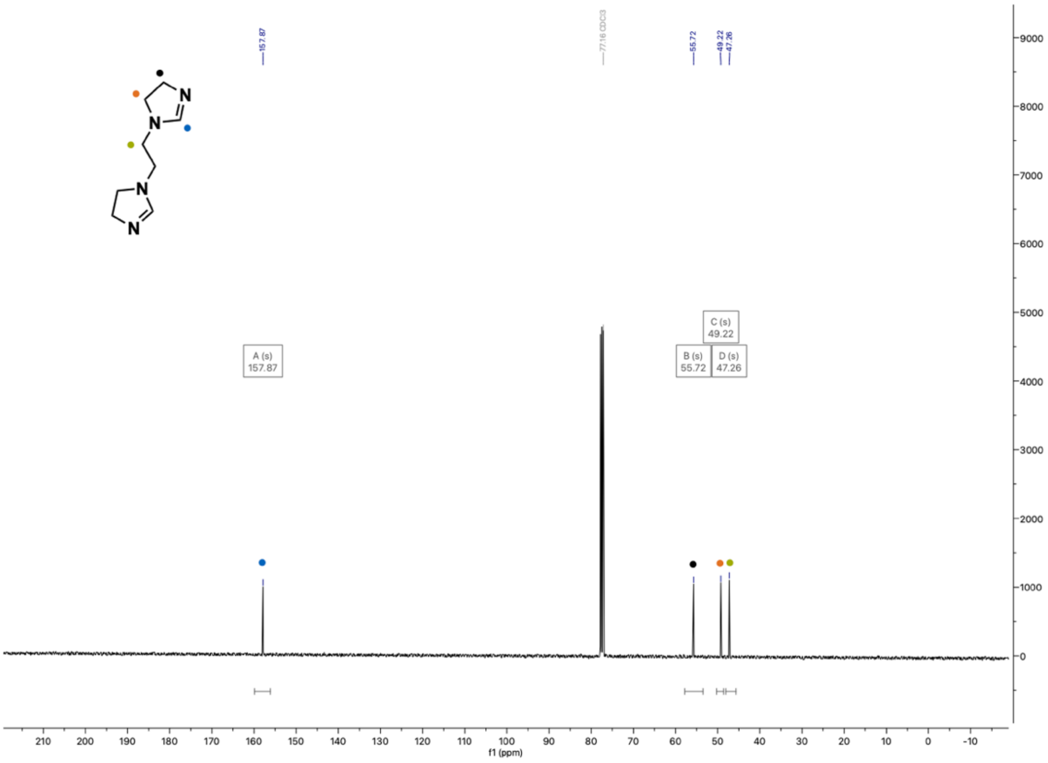

Figure 12: ^{13}C-NMR spectrum of **1** in C*D*Cl$_3$.

Alkylbisimidazolinium diiodide (2)

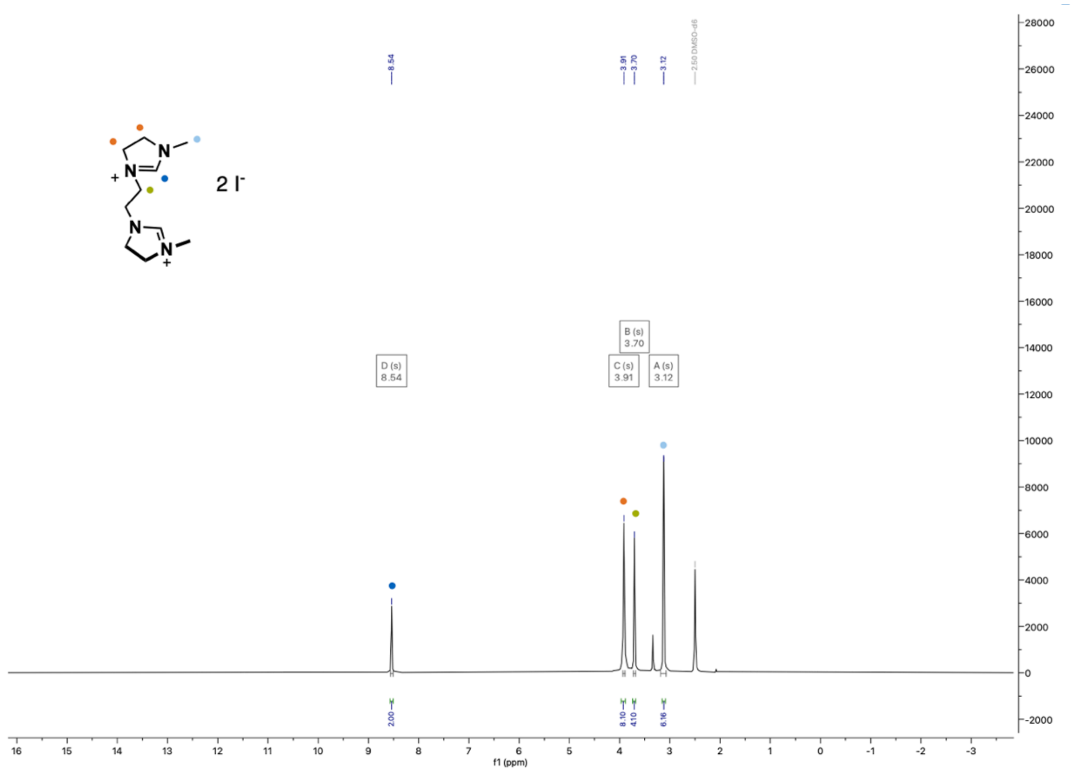

Figure 13: ^{1}H-NMR spectrum of **2** in DMSO-d_6.

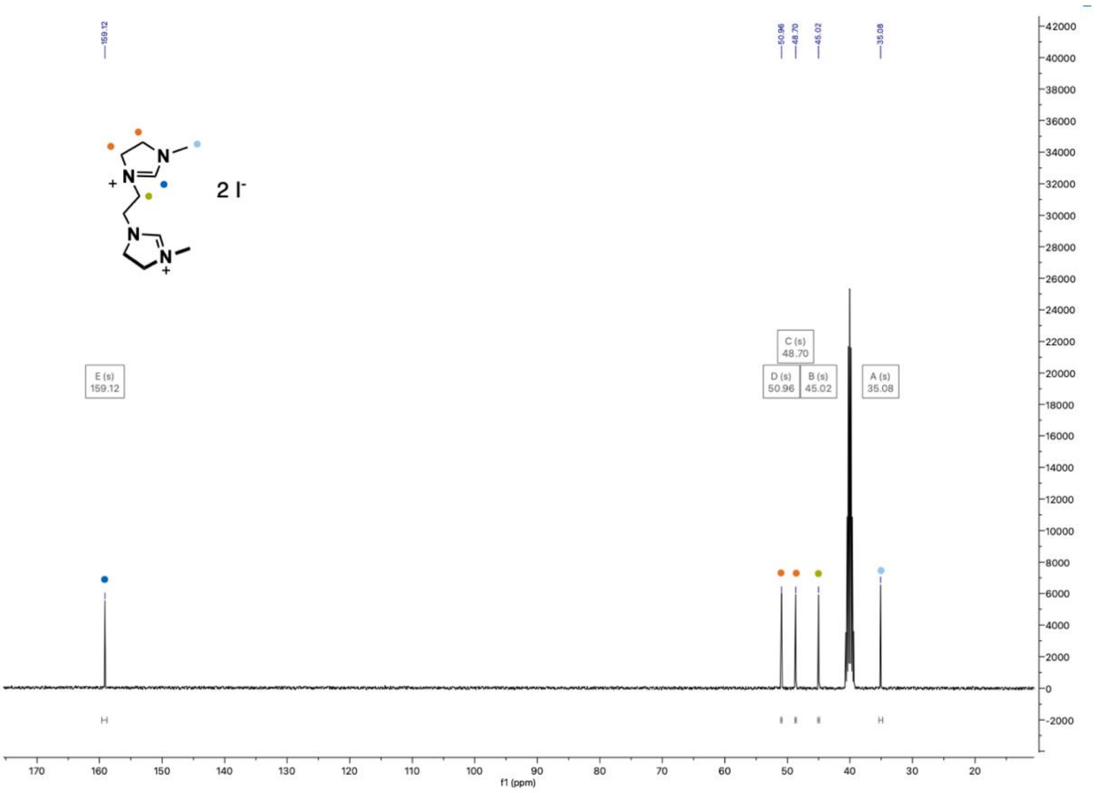

Figure 14: ^{13}C-NMR spectrum of **2** in DMSO-d_6.

Alkylbisimidazolinium hexafluorophosphate (H_2L3)

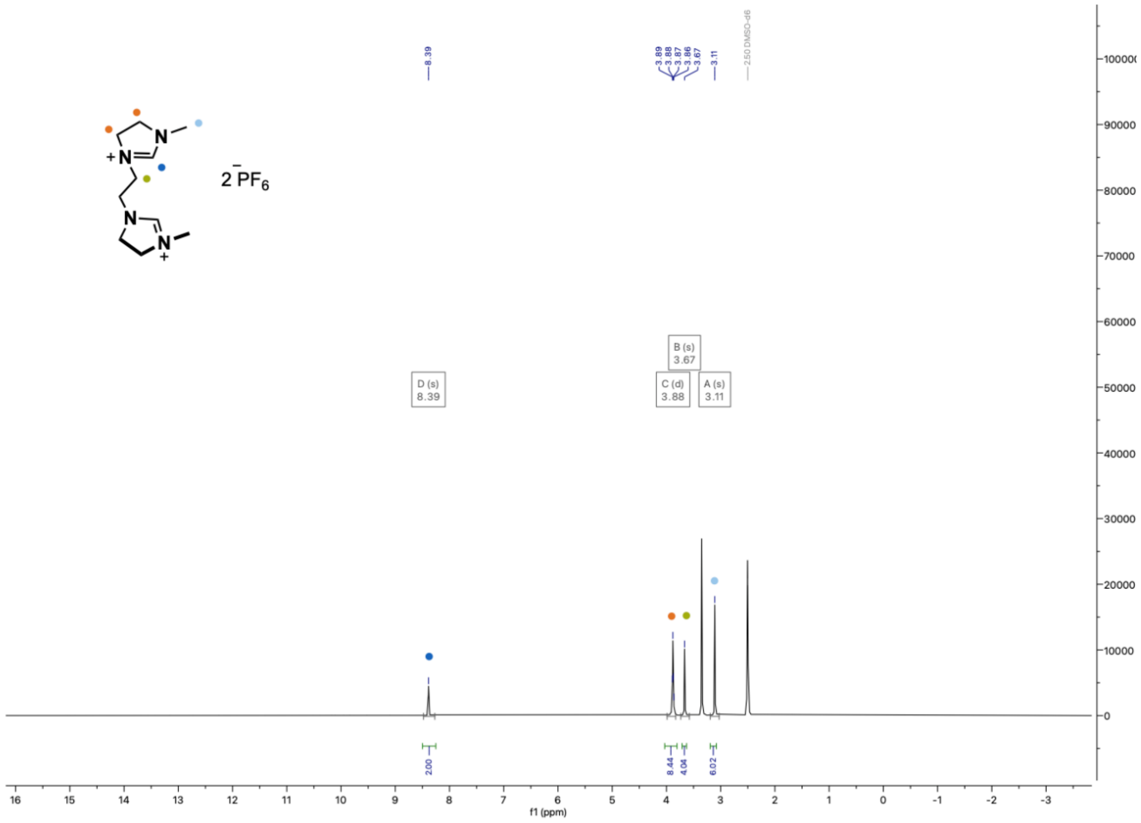

Figure 15: ^{1}H-NMR spectrum of $\mathbf{H_2L3}$ in DMSO-d_6.

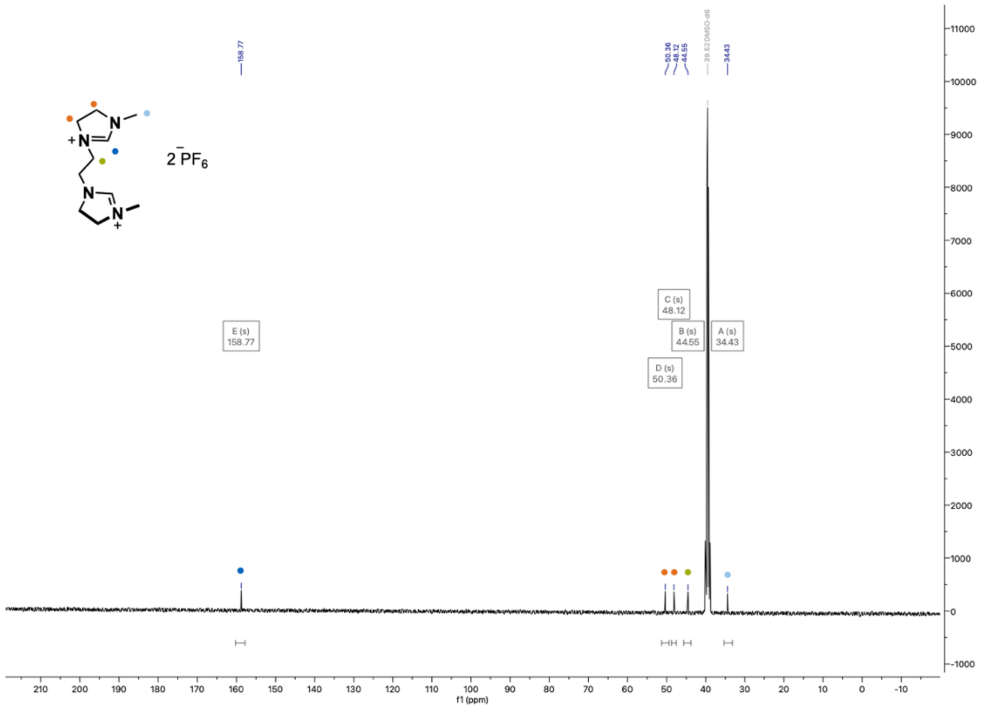

Figure 16: ^{13}C-NMR spectrum of $\mathbf{H_2L3}$ in DMSO-d_6.

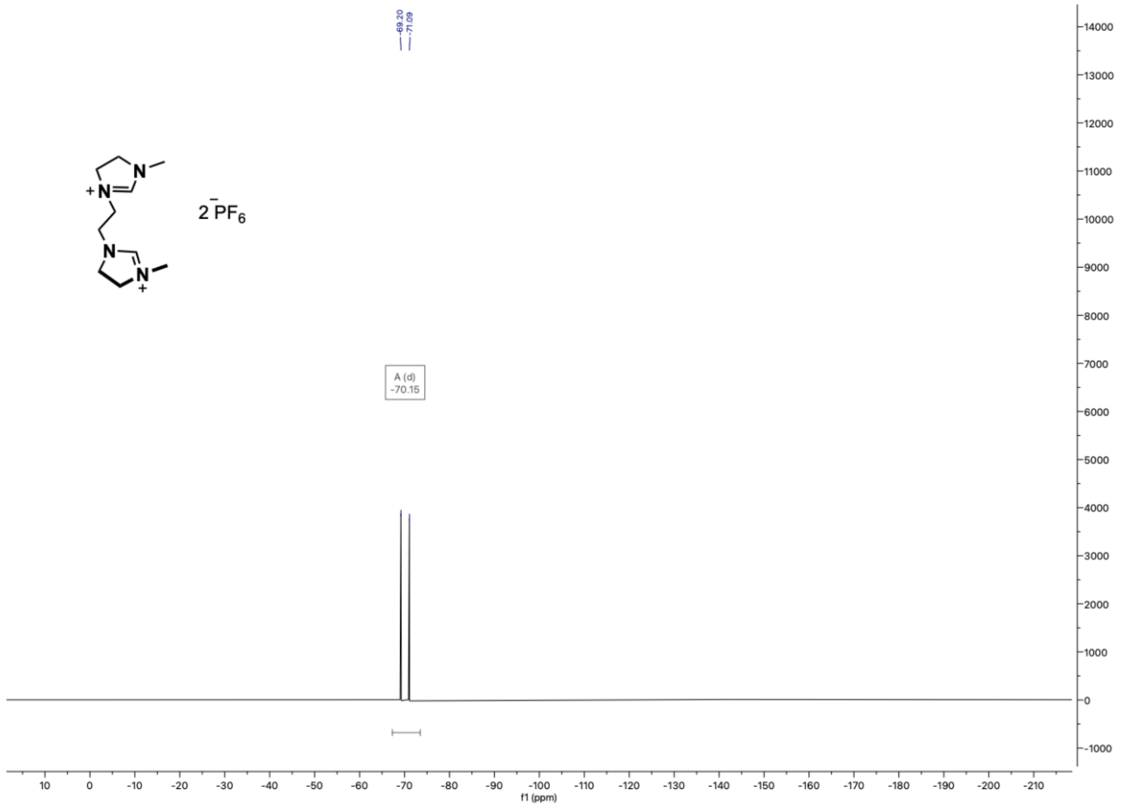

Figure 17: ^{19}F-NMR spectrum of **H_2L3**.

Pd[$C^{Et}C(Me)_2C^{Et}C(Me)_2$] hexafluorophosphate (PdL3)

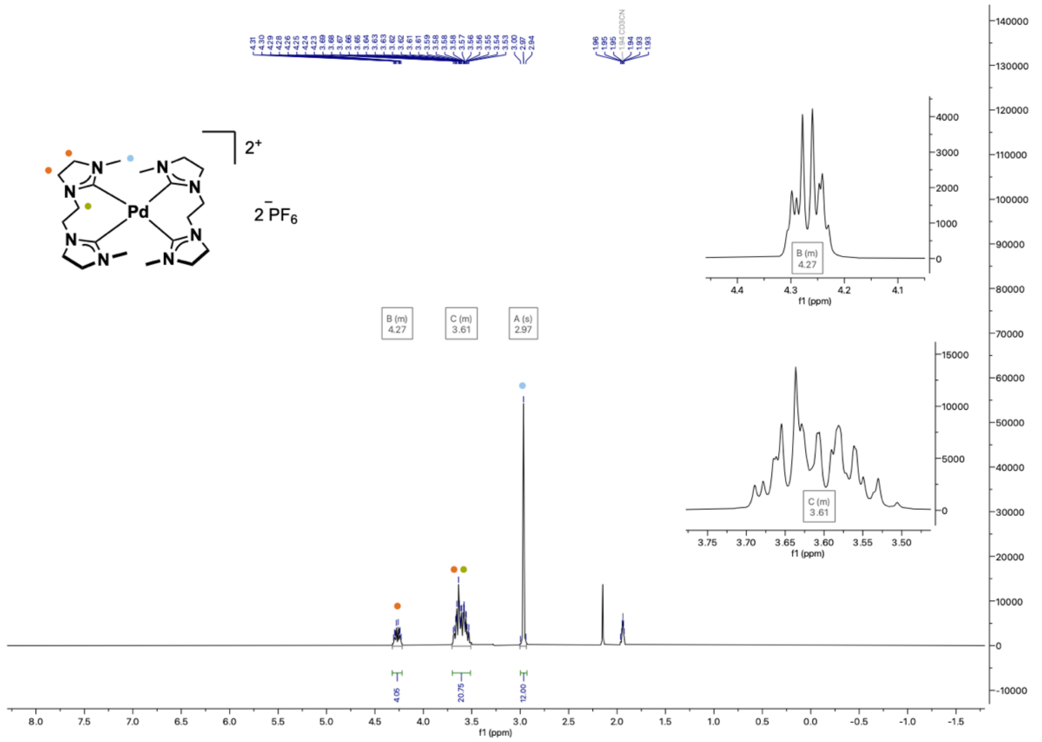

Figure 18: ¹H-NMR spectrum of **PdL3** in CD_3CN.

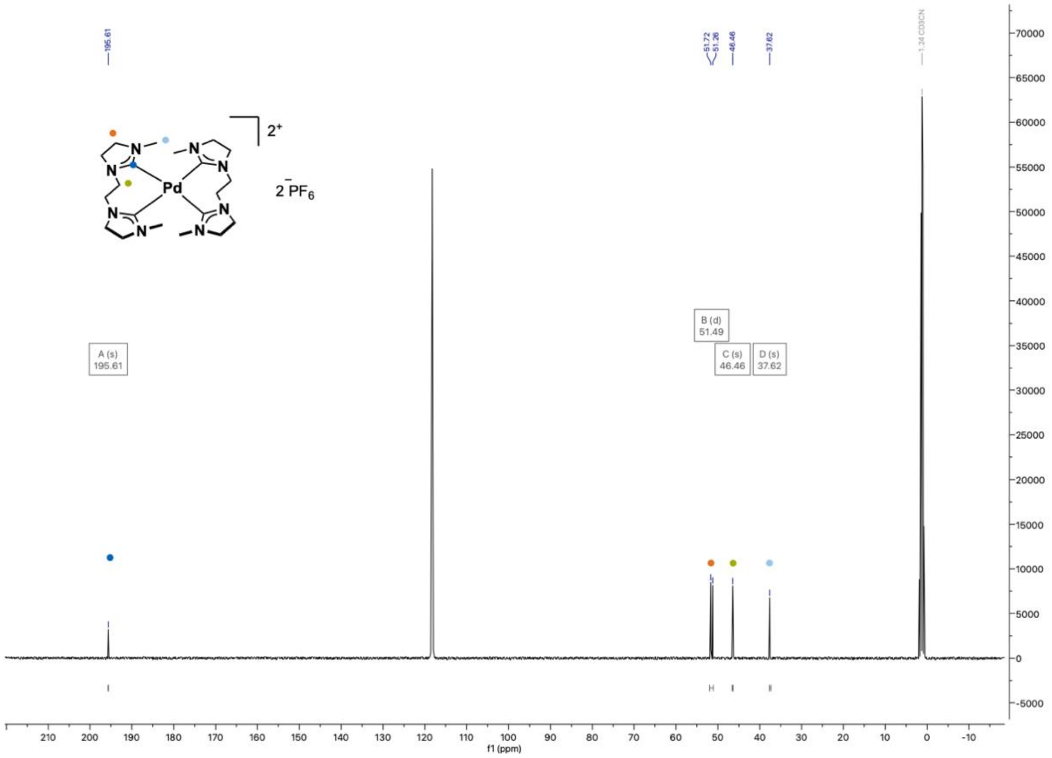

Figure 19: ¹³C-NMR spectrum of **PdL3** in CD_3CN.

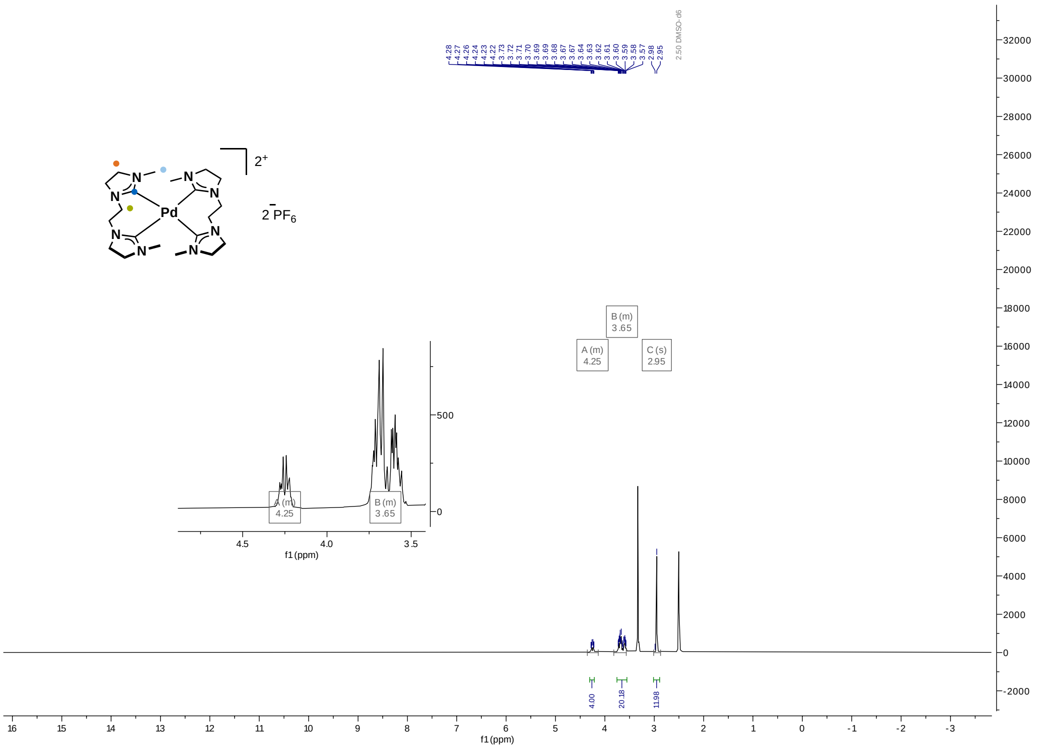

Figure 20: ^{1}H-NMR spectrum of **PdL3** in DMSO-d6.

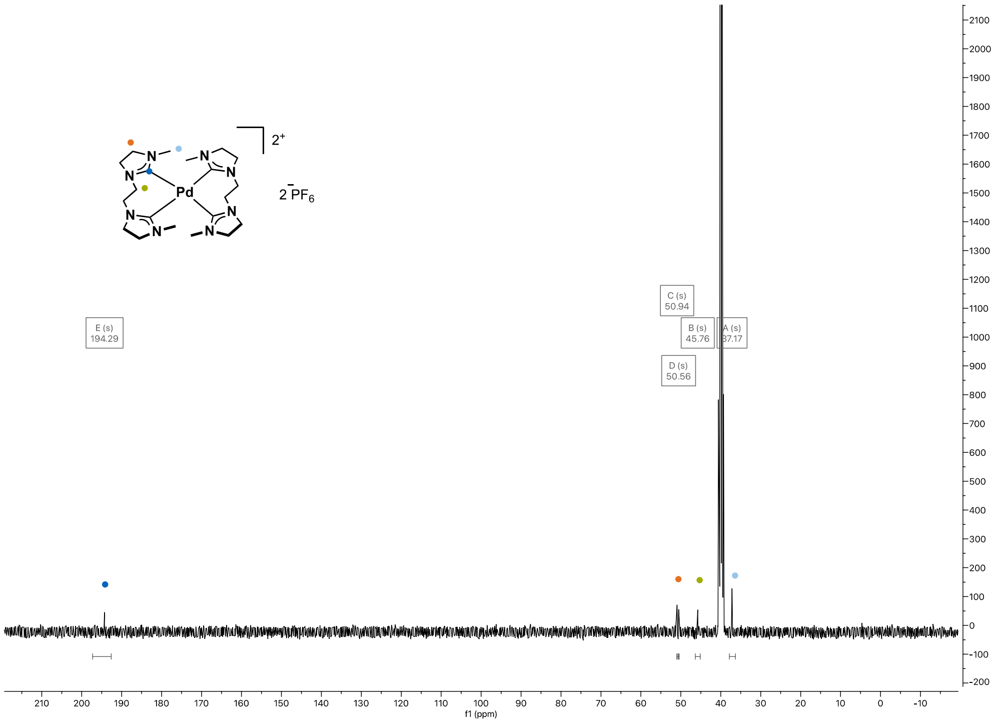

Figure 21: ^{13}C-NMR spectrum of **PdL3** in DMSO-d6.

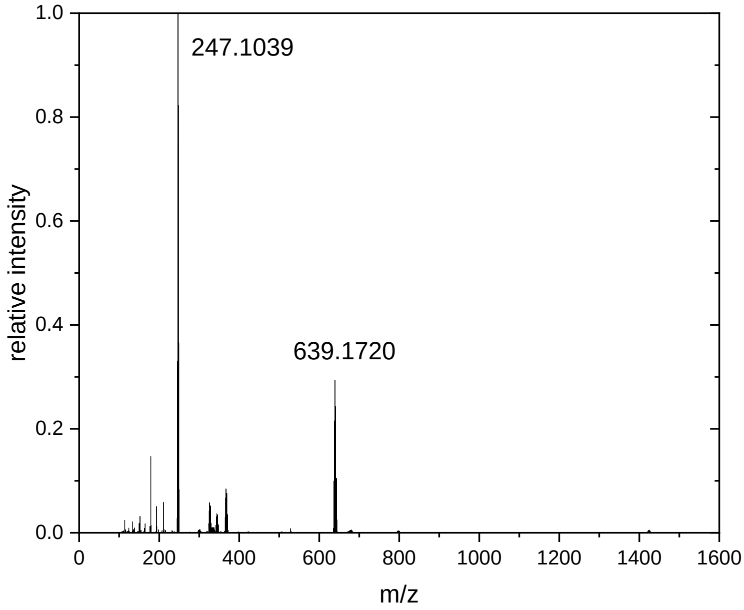

Figure 22. HR-ESI-MS spectrum of **PdL3**.

Pt[$C^{Et}C(Me)_2C^{Et}C(Me)_2$] hexafluorophosphate (PtL3)

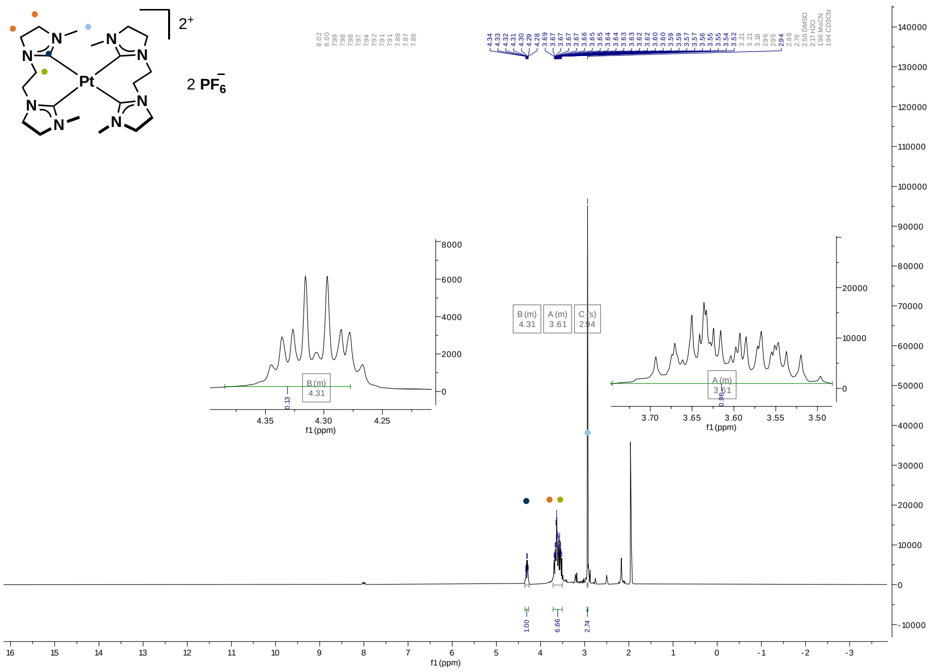

Figure 23: ^{1}H-NMR spectrum of **PtL3** in CD_3CN, with undefined impuritys.

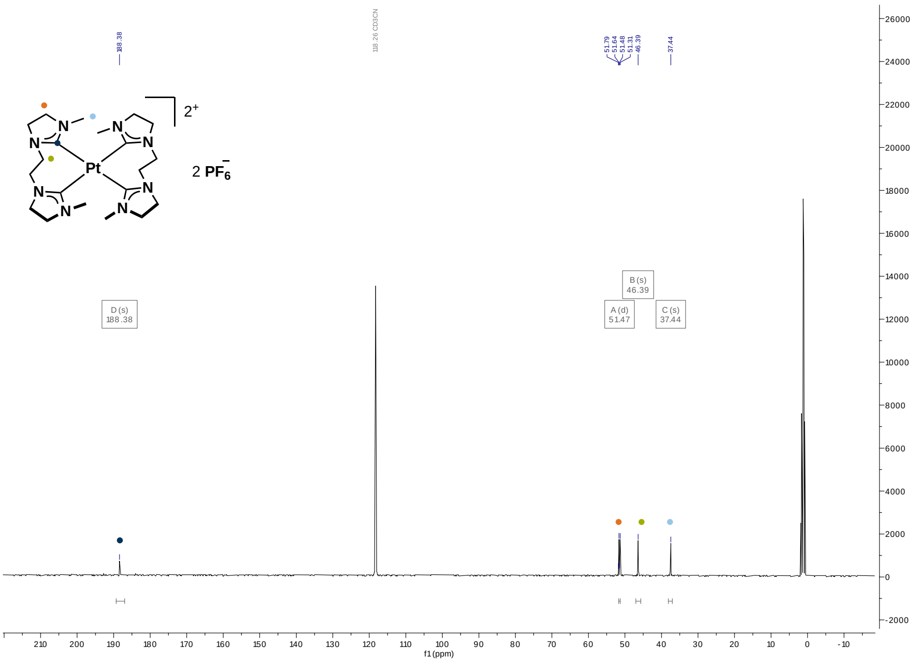

Figure 24: ^{13}C-NMR spectrum of **PtL3** in CD_3CN.

Ethylenebis(trifluormethynsulfonate) (4)

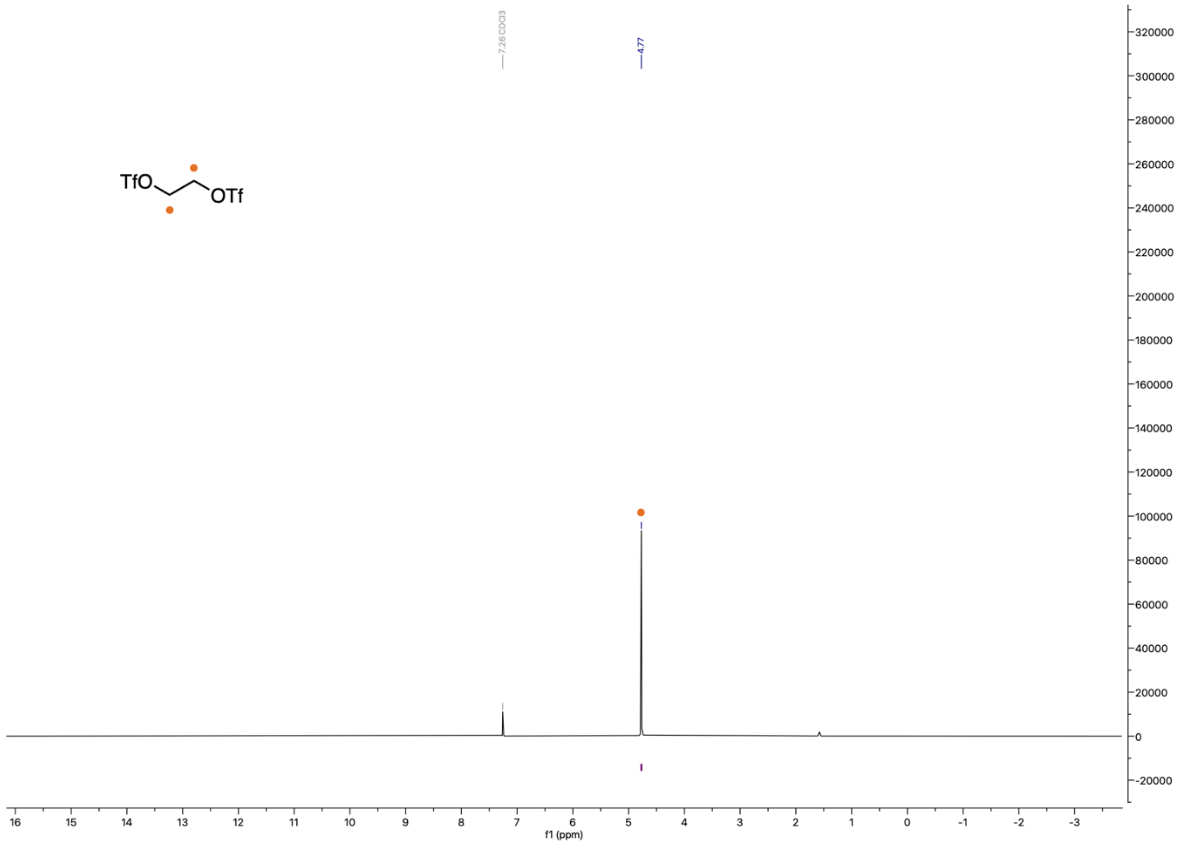

Figure 25: ^{1}H-NMR spectrum of **4** in $CDCl_3$.

Calix[4](-Et-Et-)imidazoliniumtrifluoromethansulfonate (H_4L5)

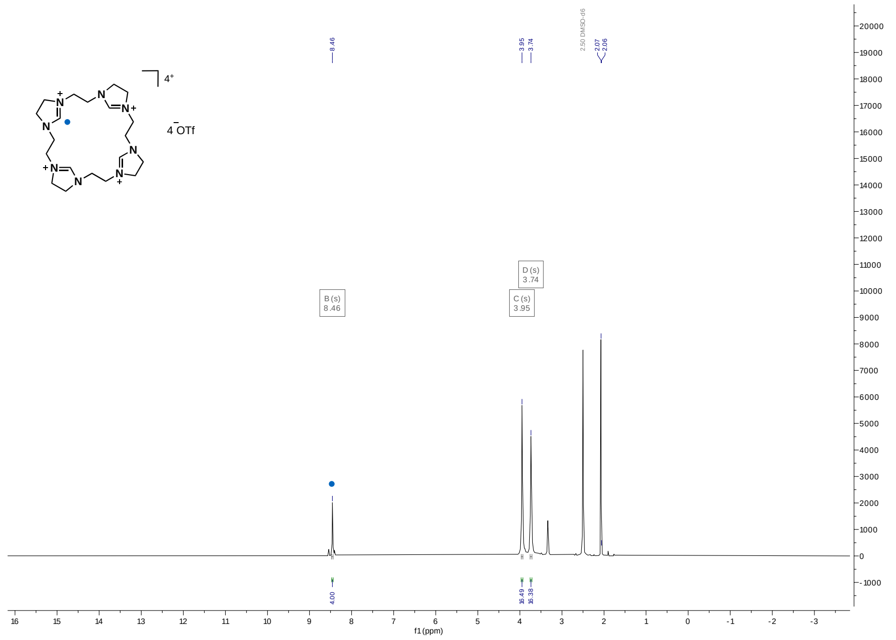

53

Figure 26: ^{1}H-NMR spectrum of **H_4L5** in DMSO-d_6. Note bridge- and backbone-protons cannot directly be assigned.

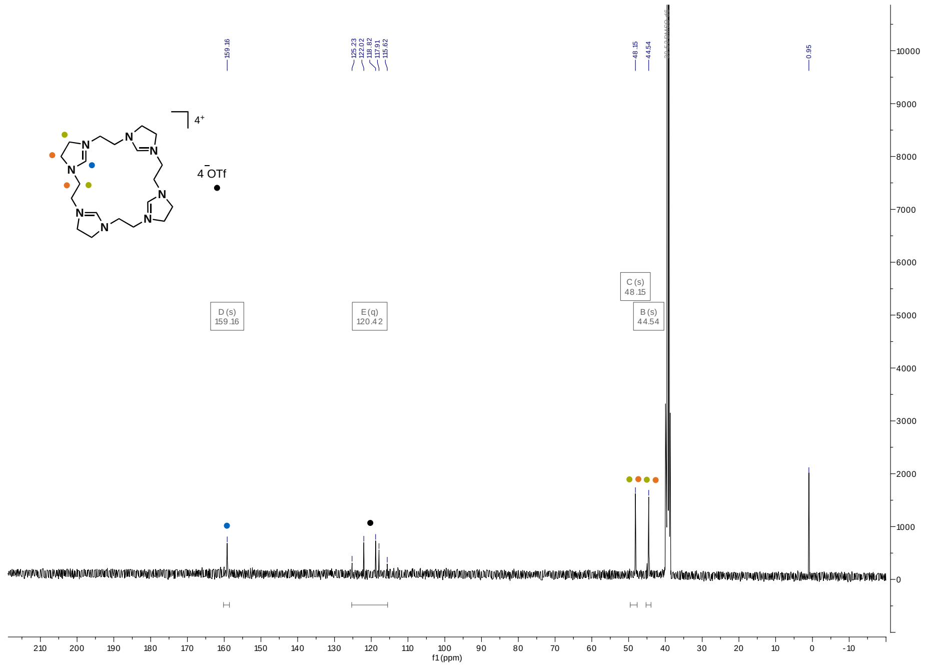

55

Figure 27: ^{13}C-NMR spectrum of **H_4L5** in DMSO-d_6. Note backbone-carbons and ethyl bridge-carbons cannot be assigned.

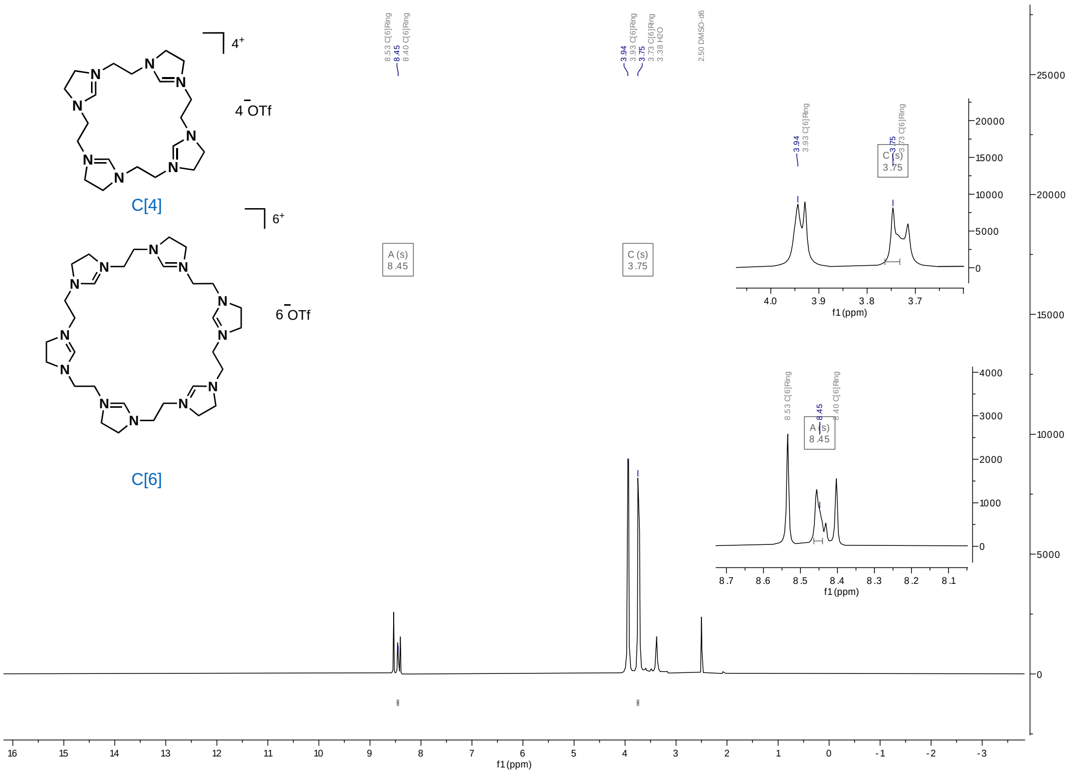

Figure 28. ^{1}H-NMR spectrum of **H$_4$L5** in DMSO-d$_6$ containing a mixture of C[4]- and C[6] units.

Calix[4](-Et-Et-)imidazoliniumhexafluorophosphat (H_4L6)

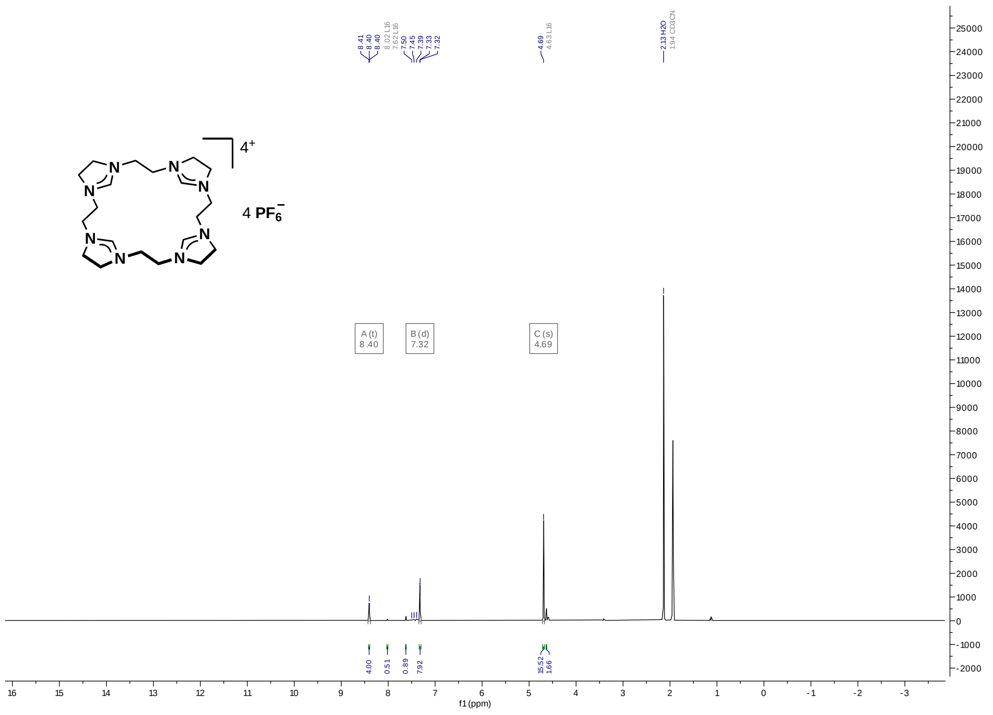

Figure 29: ^{1}H-NMR spectrum of $\mathbf{H_4L6}$ in CD_3CN. Impurity $\mathbf{H_4L6}$.

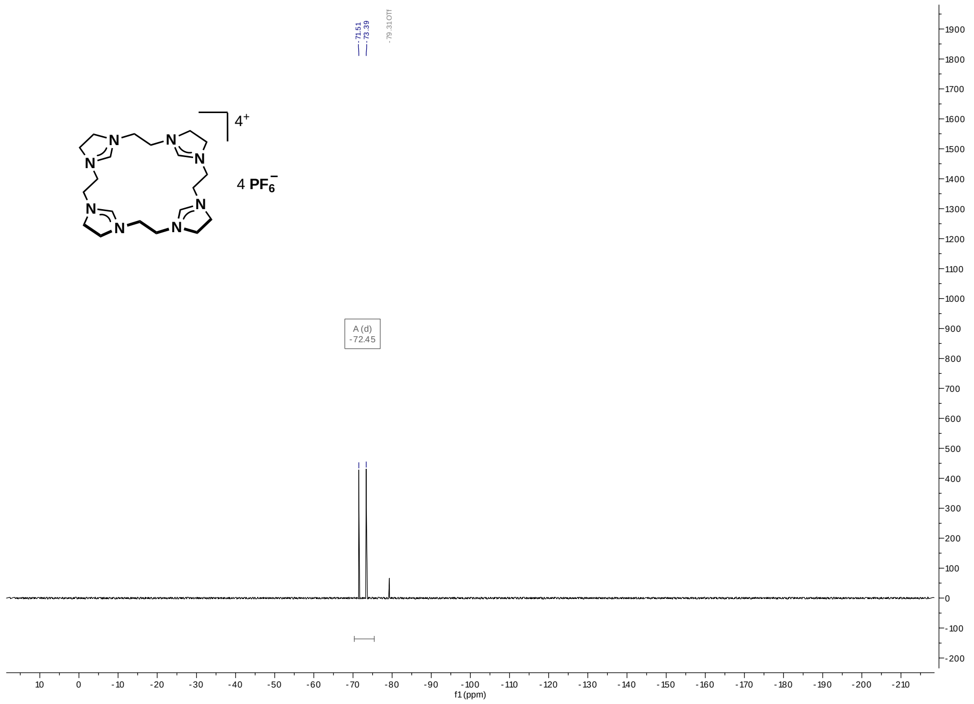

Figure 30: ^{19}F-NMR spectrum of $\mathbf{H_4L6}$ in CD_3CN. Impurity $\mathbf{H_4L5}$ OTf.

Pd[(cC^{Et}CC^{Et}C$_{imi}$)OTf] (PdL5)

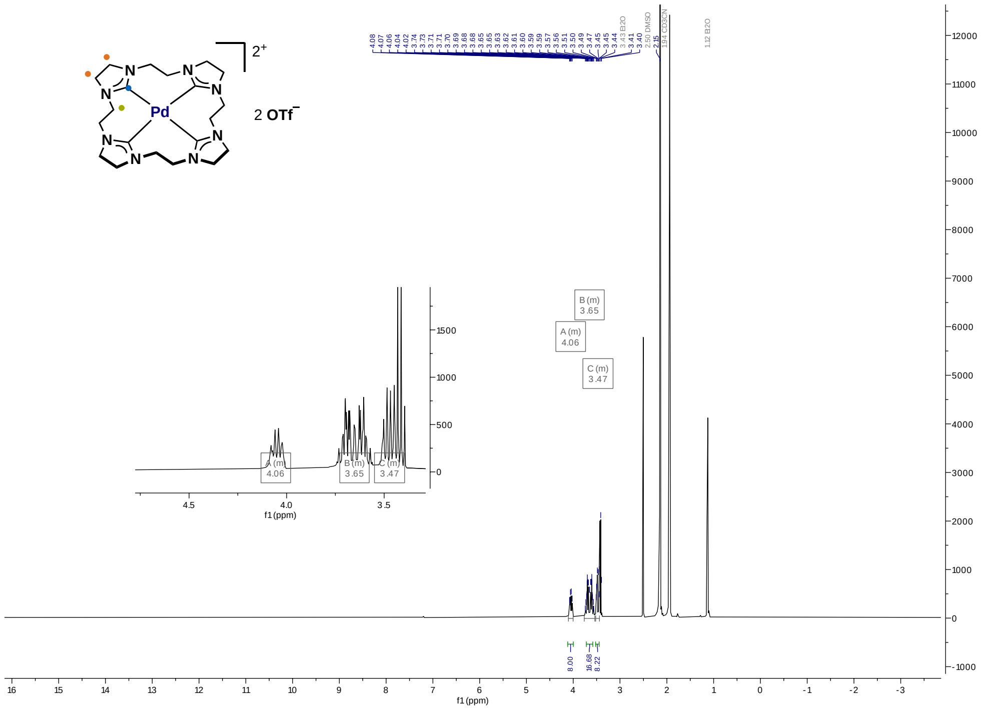

Figure 31: ^{1}H-NMR spectrum of **PdL5** in CD_3CN.

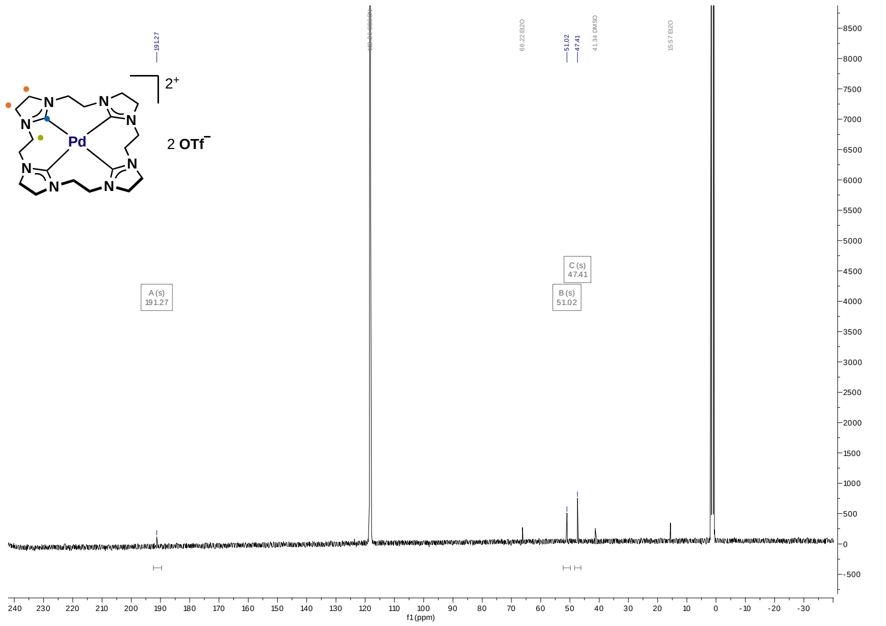

Figure 32: ^{13}C-NMR spectrum of **PdL5** in CD_3CN. Impurities Et_2O at 66.22 ppm, DMSO at 41.34 ppm and H_2O at 15.57 ppm.

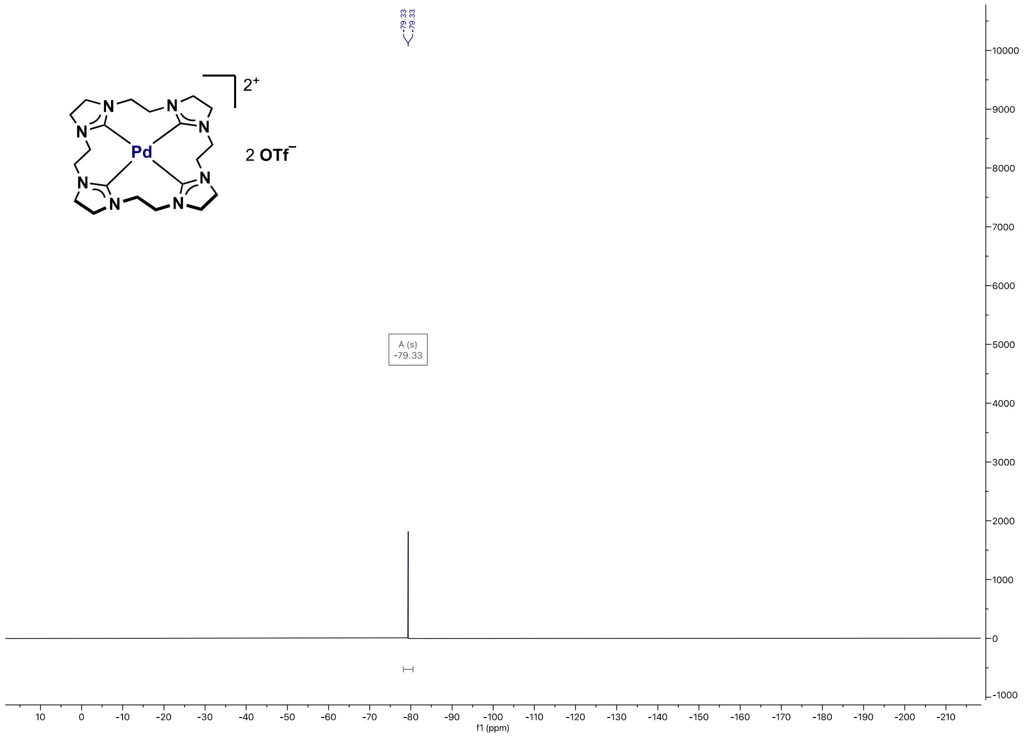

Figure 33: ^{19}F-NMR spectrum of **PdL5** in CD_3CN.

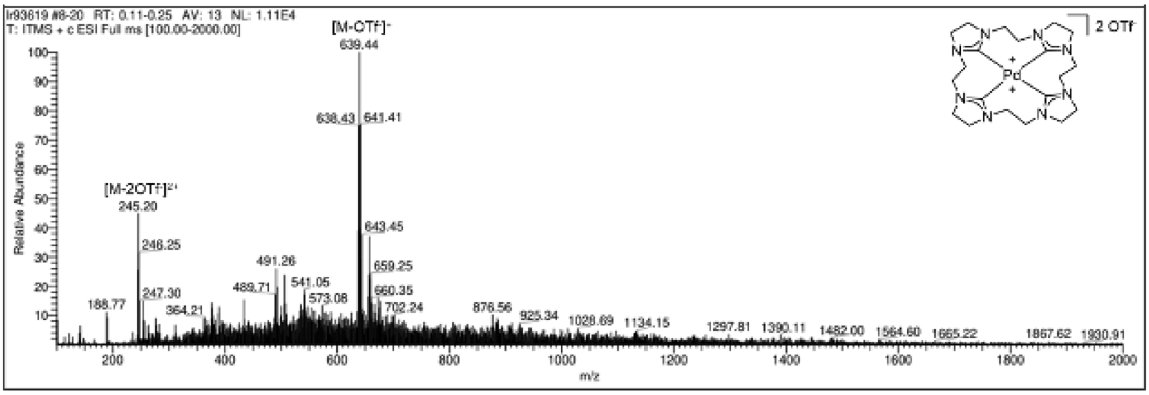

Figure 34: ESI-MS spectrum of **PdL5**.

Pd[(cC^{Et}CC^{Et}C$_{imi}$)PF$_6$] (PdL6)

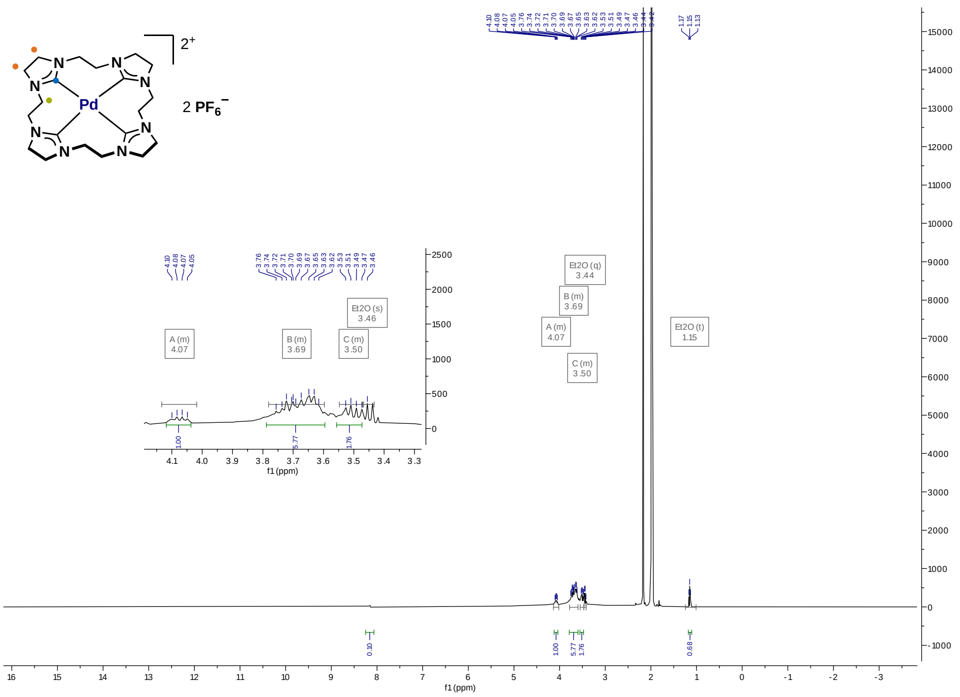

Figure 35: ^{1}H-NMR spectrum of **PdL6** in CD_3CN.

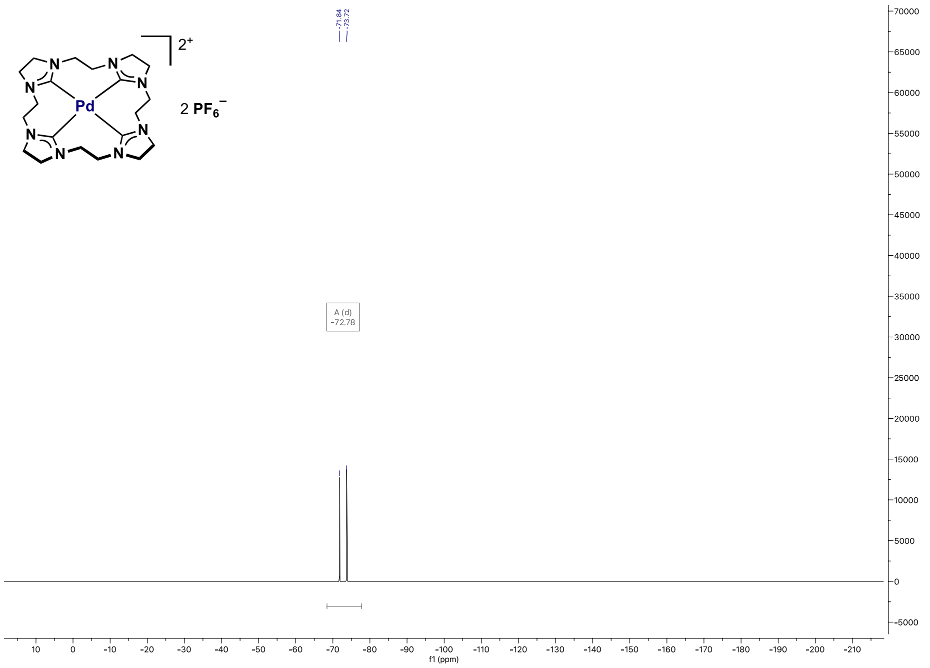

Figure 36: ^{19}F-NMR spectrum of **PdL6** in CD_3CN.

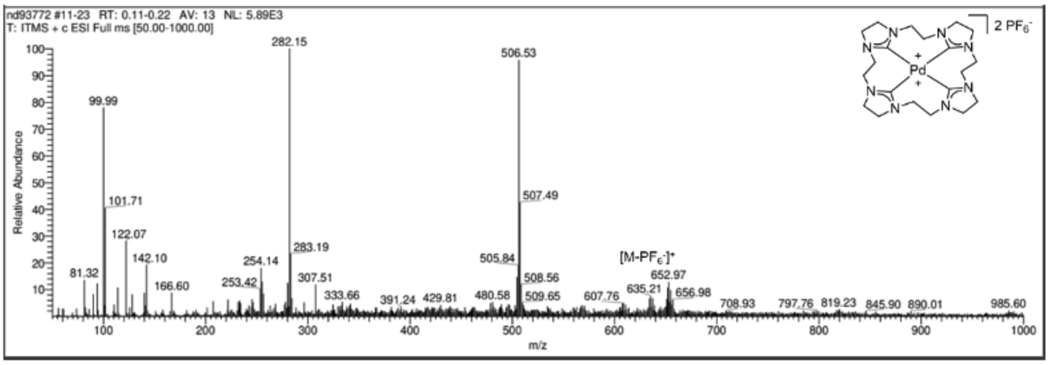

Figure 37: ESI-MS spectrum of **PdL6**.

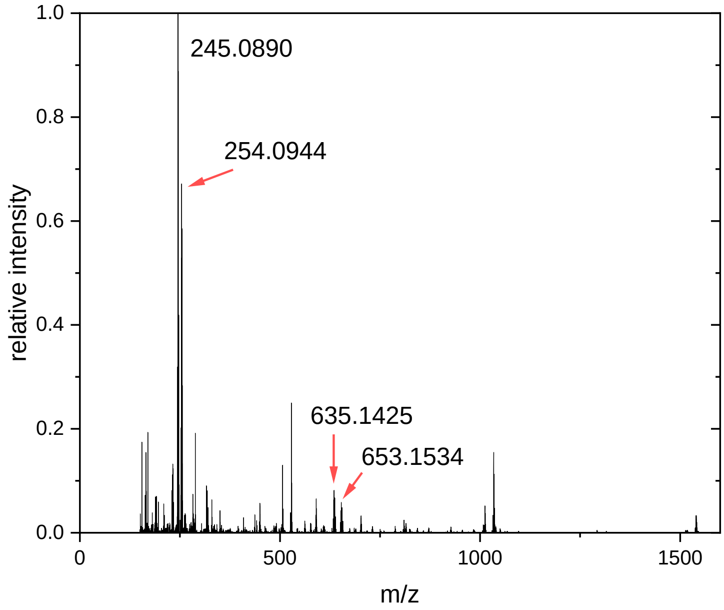

Figure 38. HR-ESI-MS spectrum of **PdL6**.

1,1’-Ethylenebis-1*H*-imidazolyl (7)

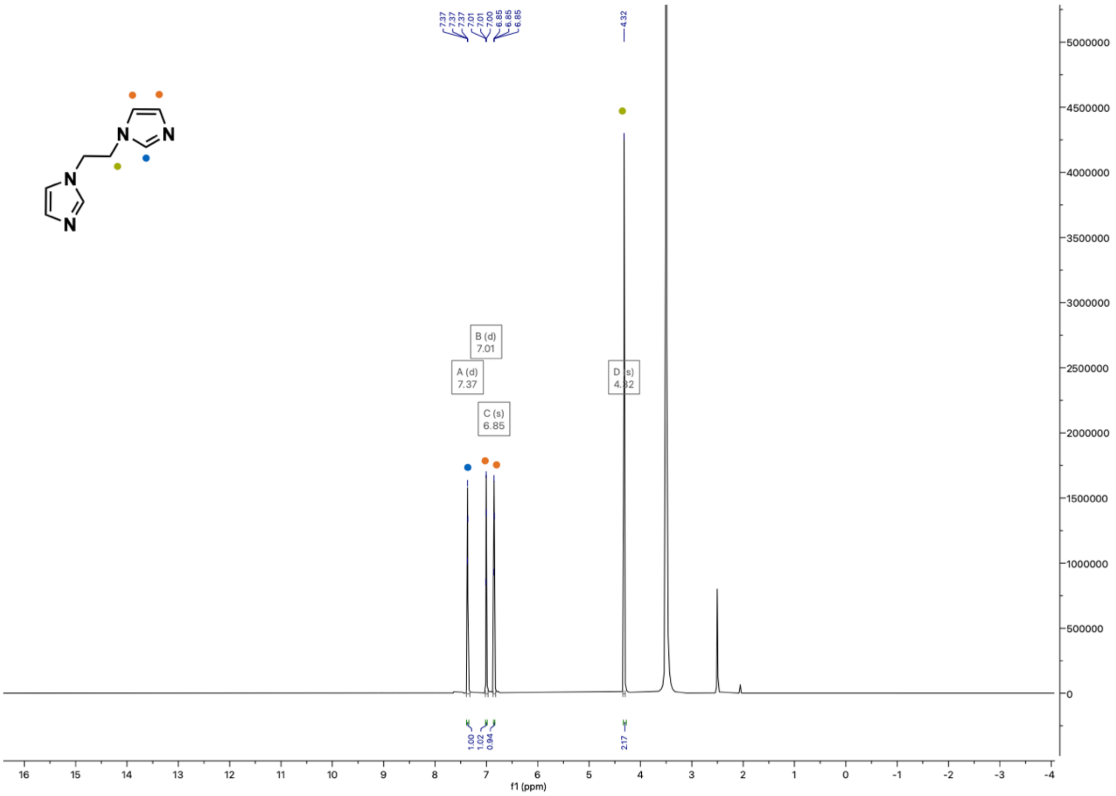

Figure 39: ^{1}H-NMR spectrum of **7** in DMSO-d_6.

Calix[4](-Et-Et-)imidazoliumtrifluoromethansulfonate (H4L8)

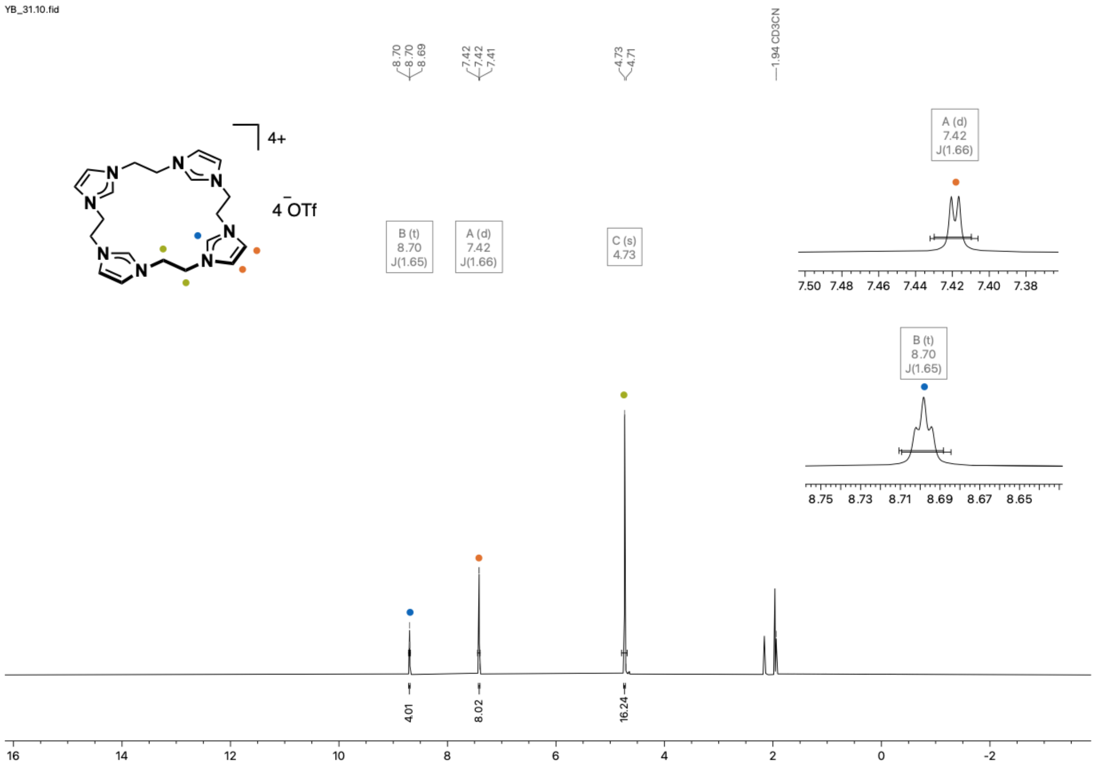

Figure 40: ^{1}H-NMR spectrum of **H$_4$L8** in CD$_3$CN.

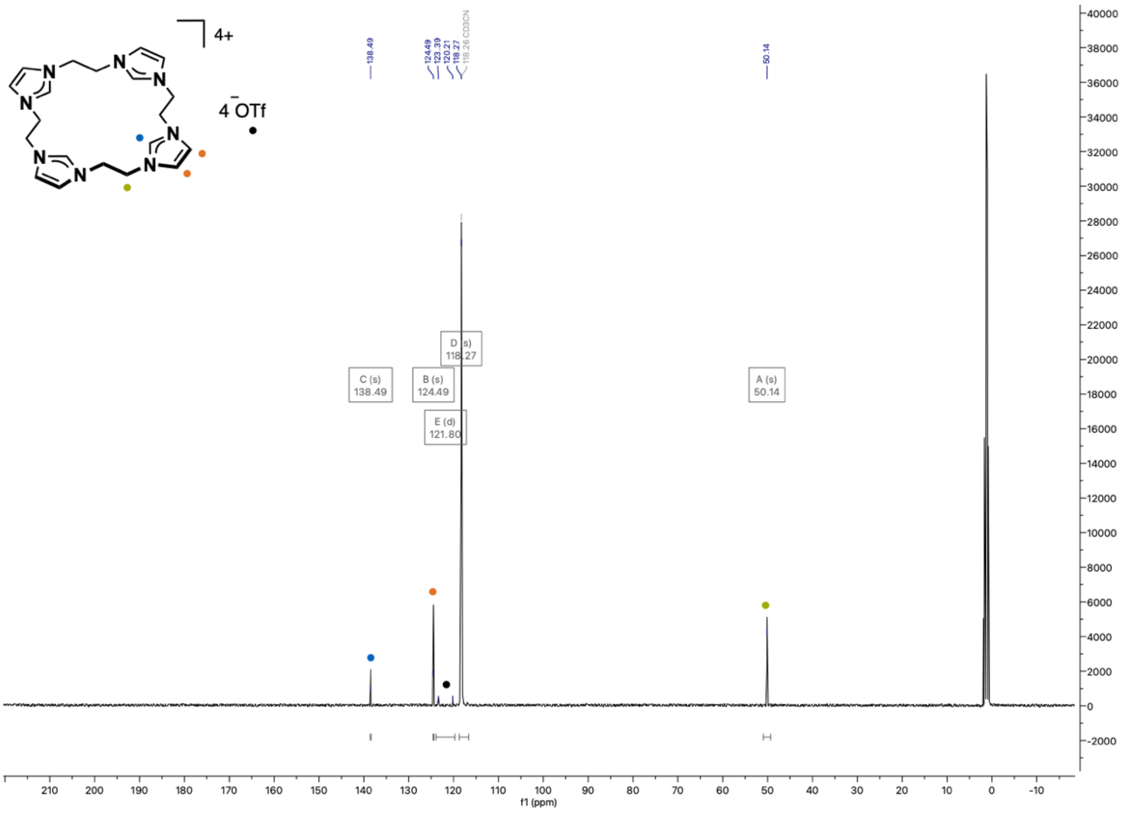

Figure 41: ^{13}C-NMR spectrum of **H$_4$L8** in CD$_3$CN.

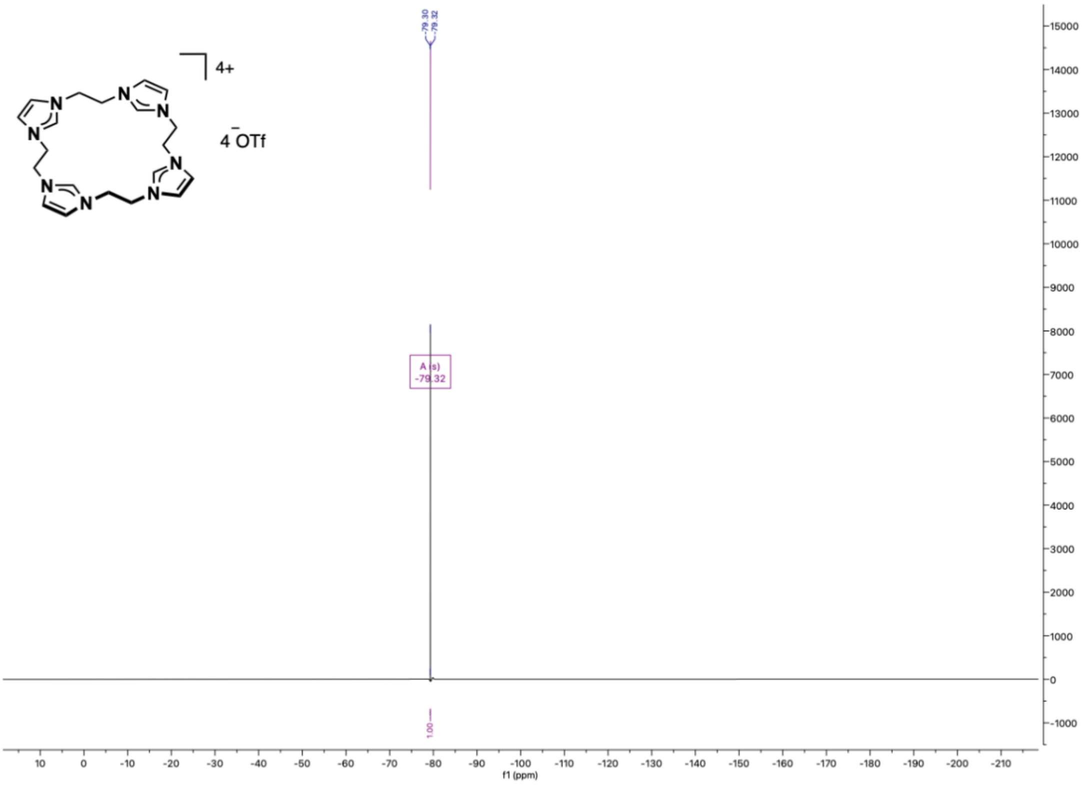

Figure 42: ^{19}F-NMR spectrum of **H_4L8** in CD_3CN.

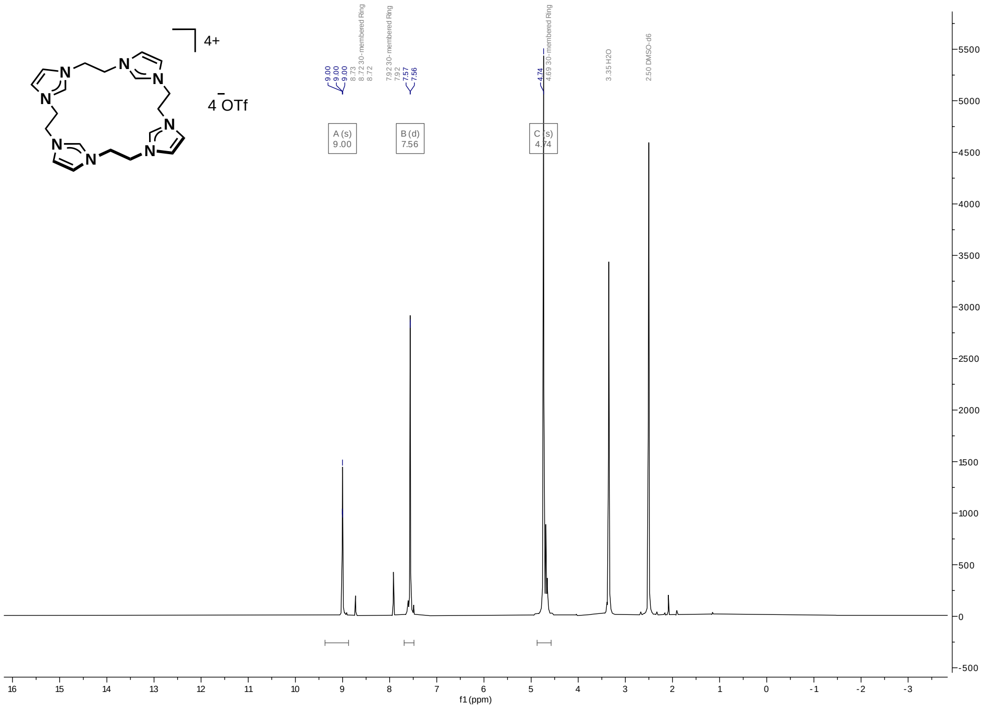

Figure 43: ^{1}H-NMR spectrum of **H_4L8** with the C[4]/[6] mixture in DMSO-d_6.

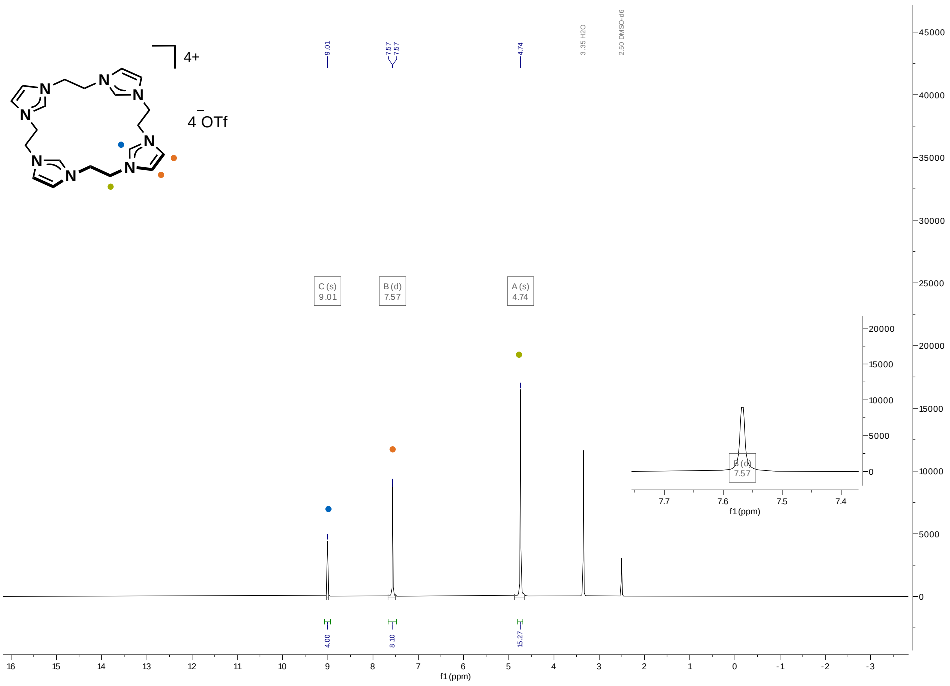

Figure 44: ^{1}H-NMR spectrum of **H_4L8** with the increased cooling period in DMSO-d_6.

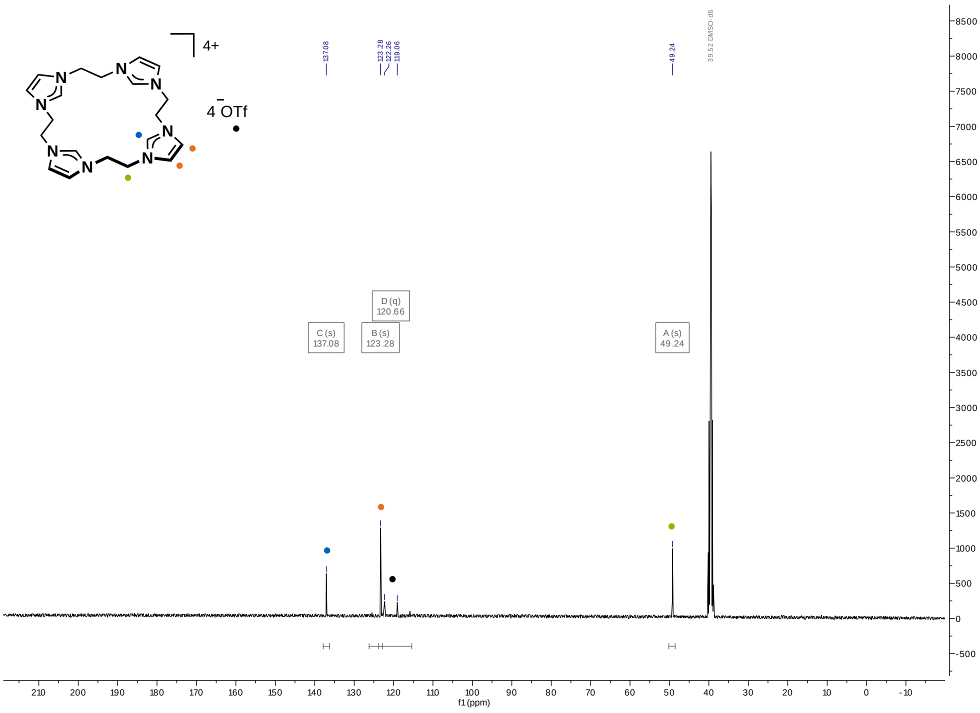

Figure 45: ^{13}C-NMR spectrum of **H_4L8** in DMSO-d_6.

Calix[4](-Et-Et-)imidazoliumhexafluorophosphate (H$_4$L9)

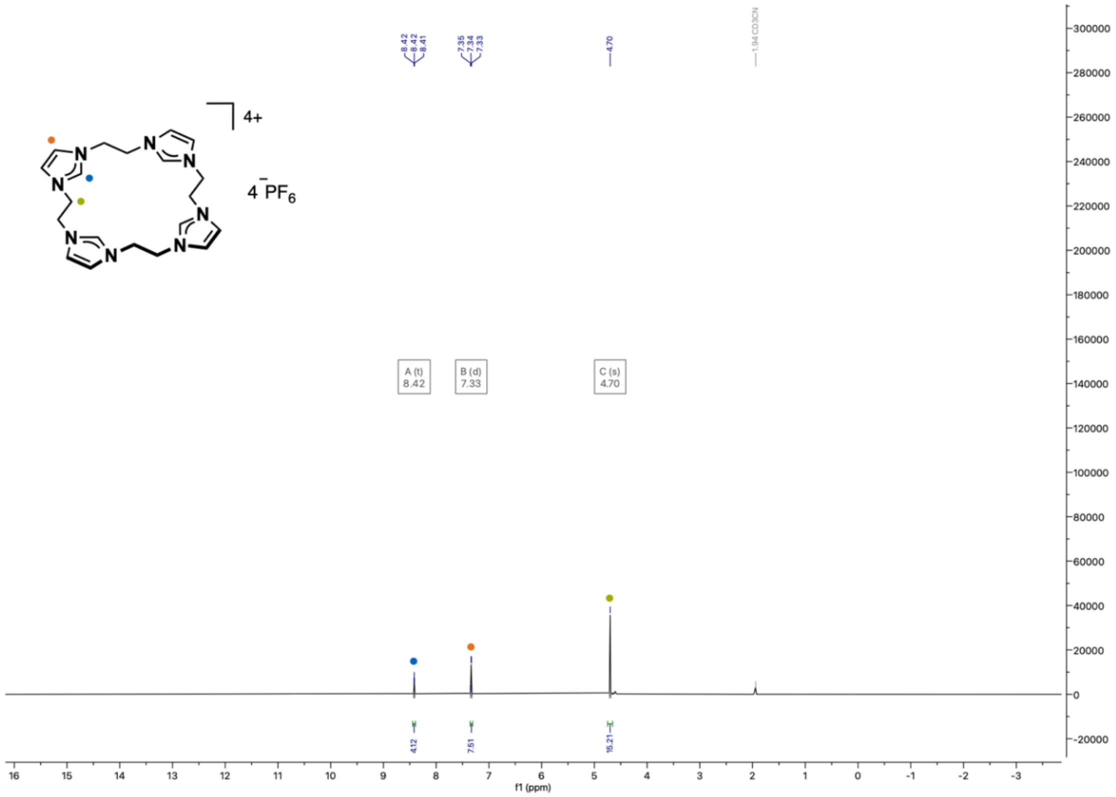

Figure 46: ^{1}H-NMR spectrum of **H$_4$L9** in CD_3CN.

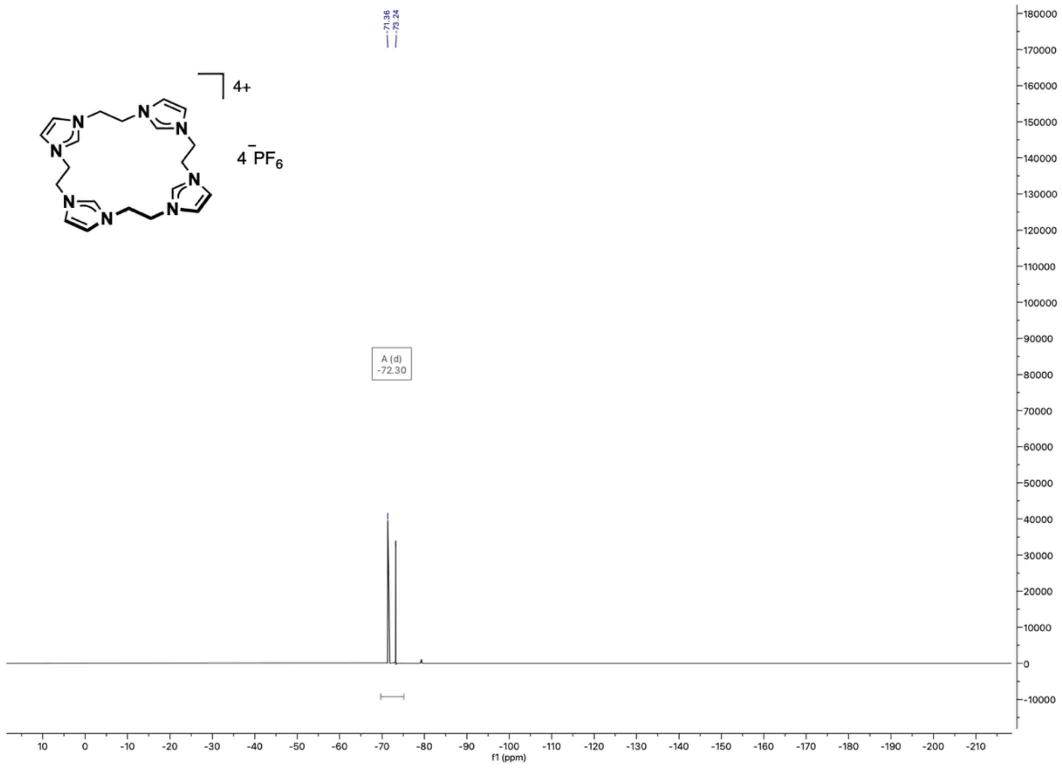

Figure 47: ^{19}F-NMR spectrum of **H$_4$L9** in CD_3CN.

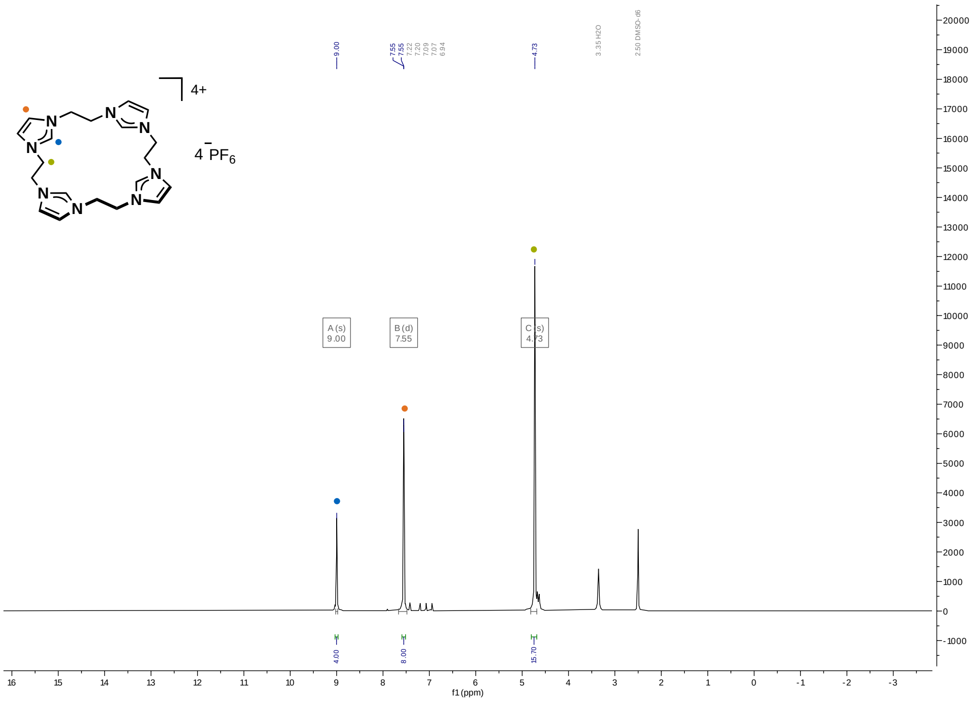

Figure 48. ^{1}H-NMR spectrum of **H_4L9** in DMSO-d_6, with Ammonium as impurity at 6.94–7.22 ppm.

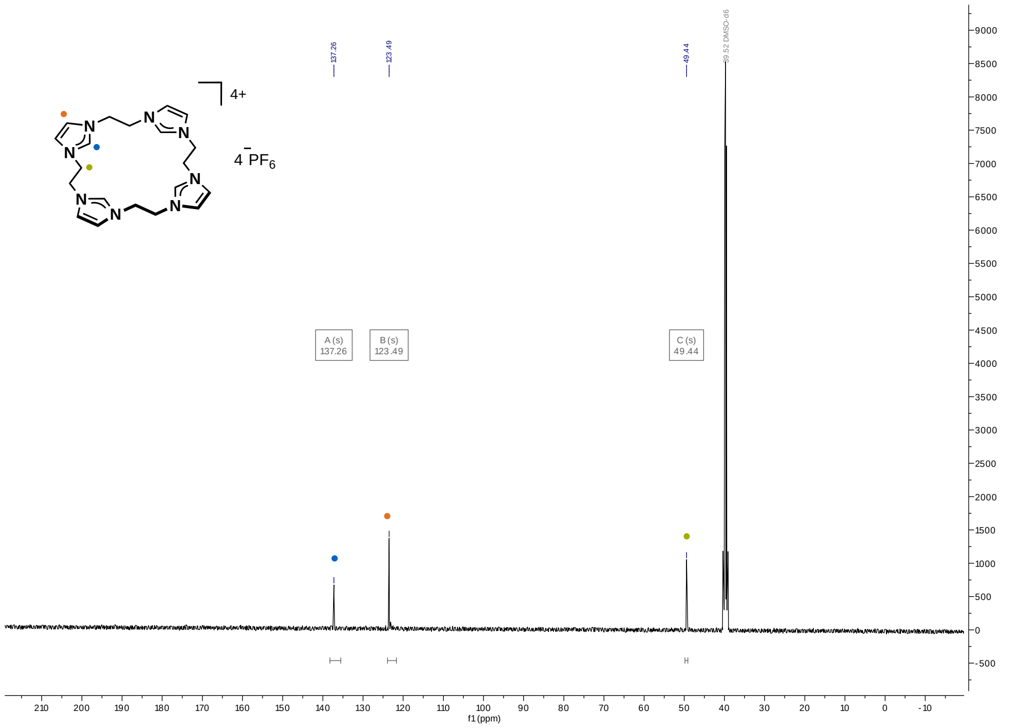

Figure 49. ^{13}C-NMR spectrum of **H_4L9** in DMSO-d_6.

Pd[(cC^{Et}CC^{Et}C)OTf] (PdL8)

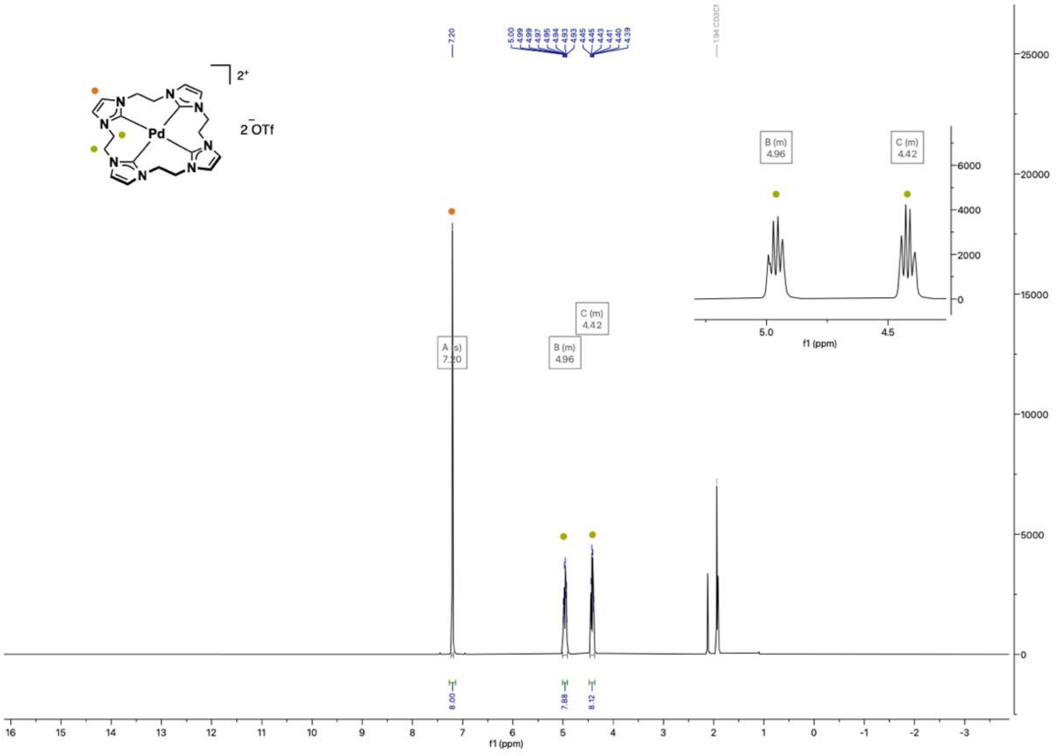

Figure 50: ^{1}H-NMR spectrum of **PdL8** in CD_3CN.

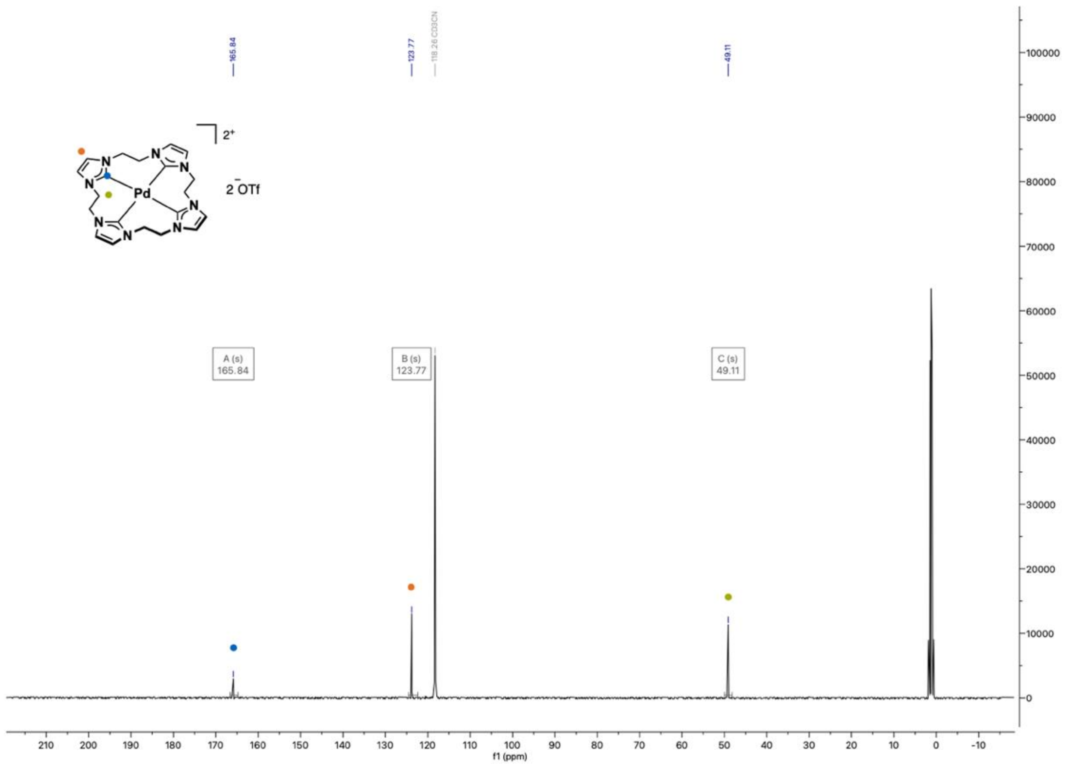

Figure 51: ^{13}C-NMR spectrum of **PdL8** in CD_3CN.

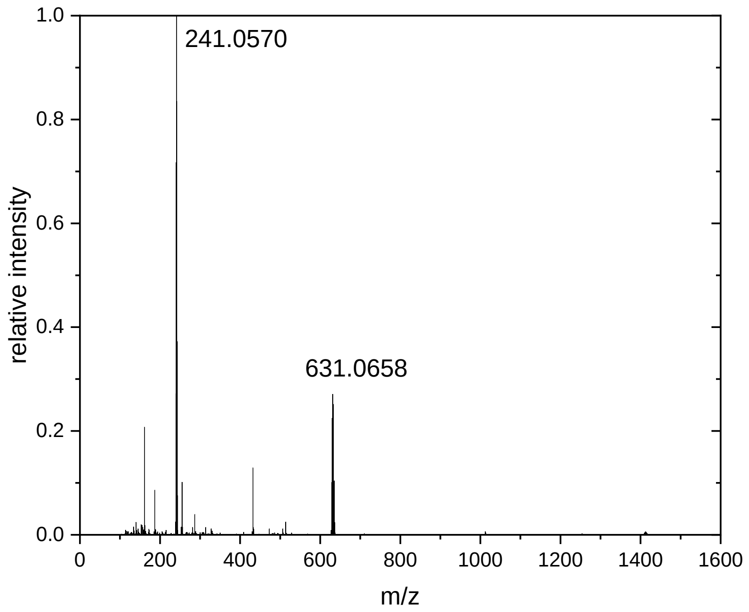

Figure 52. HR-ESI-MS spectrum of **PdL8**.

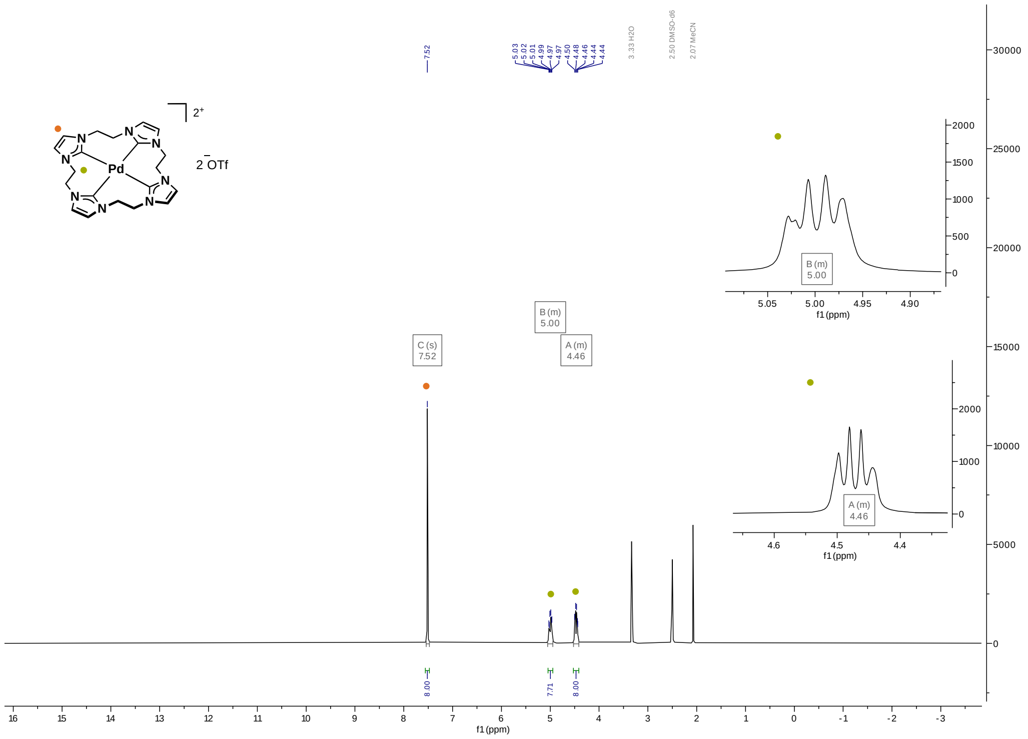

Figure 53. ^{1}H-NMR spectrum of **PdL8** in DMSO-d$_6$.

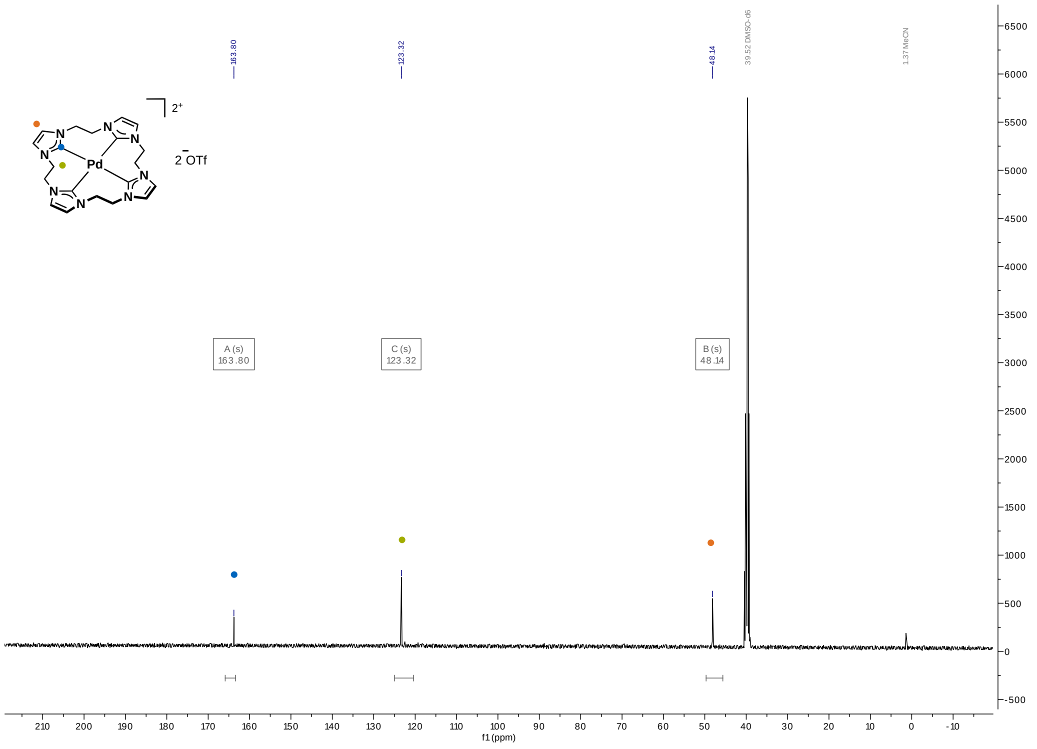

Figure 54. ^{13}C-NMR spectrum of **PdL8** in DMSO-d$_6$.

Pd[(cC^Et^CC^Et^C)PF$_6$] (PdL9)

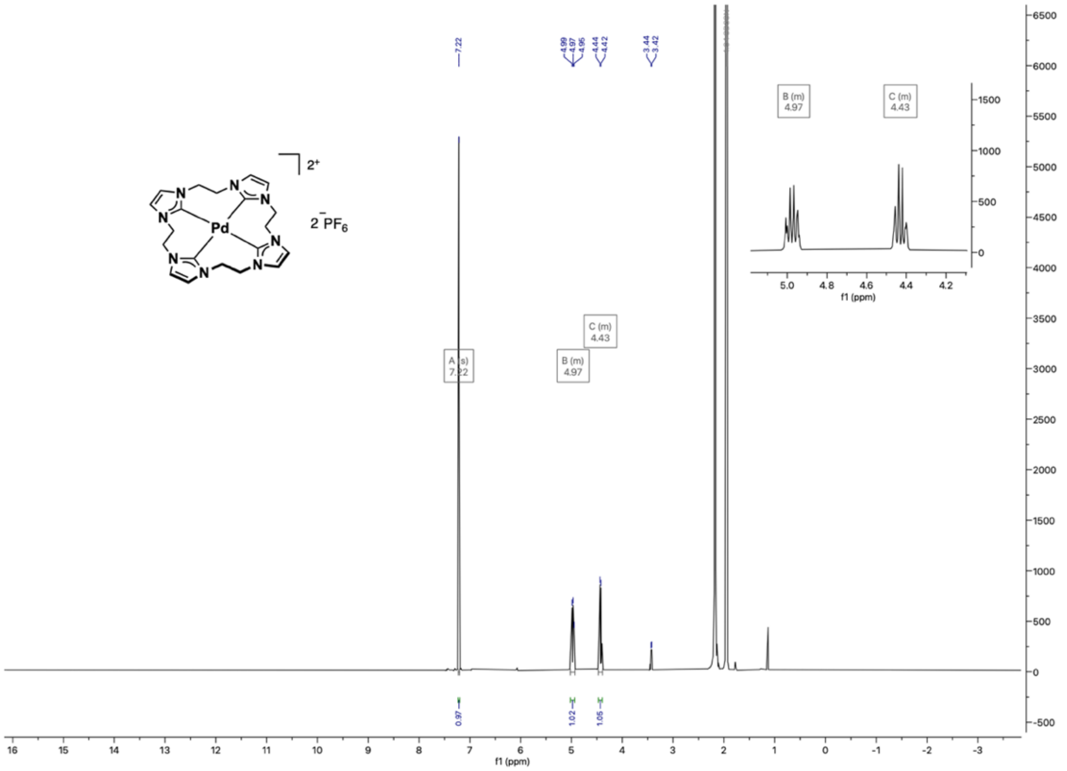

Figure 55: ^{1}H-NMR spectrum of **PdL9** in CD_3CN. Diethyl ether still visible at 3.41 ppm and 1.12 ppm.

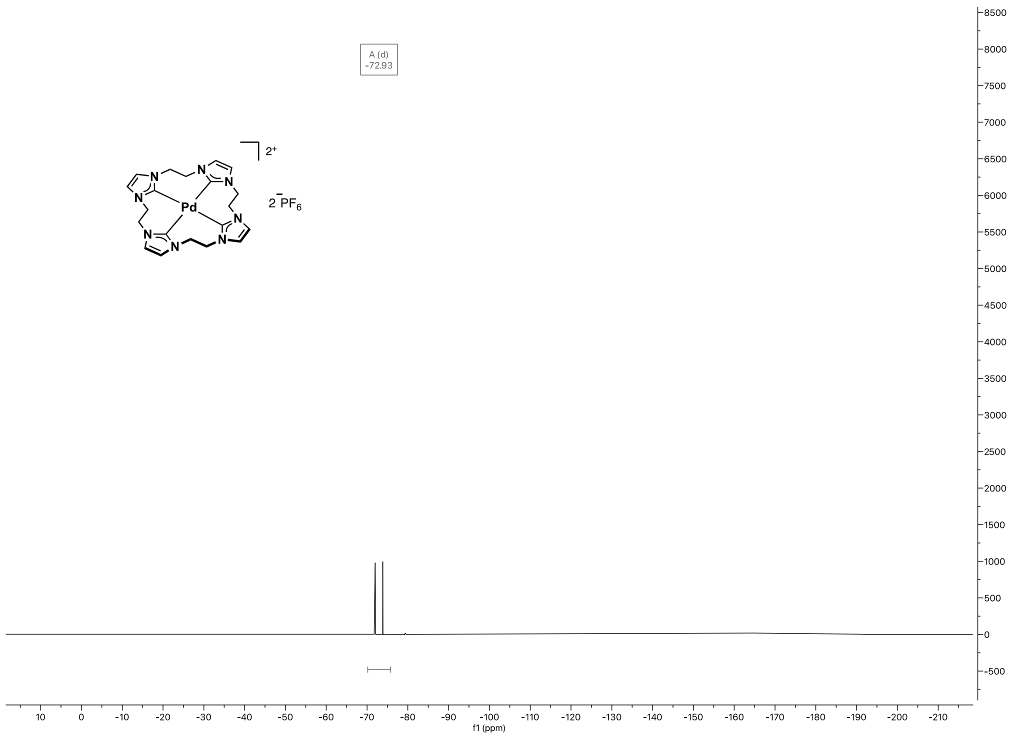

Figure 56: ^{19}F-NMR spectrum of **PdL9** in CD_3CN.

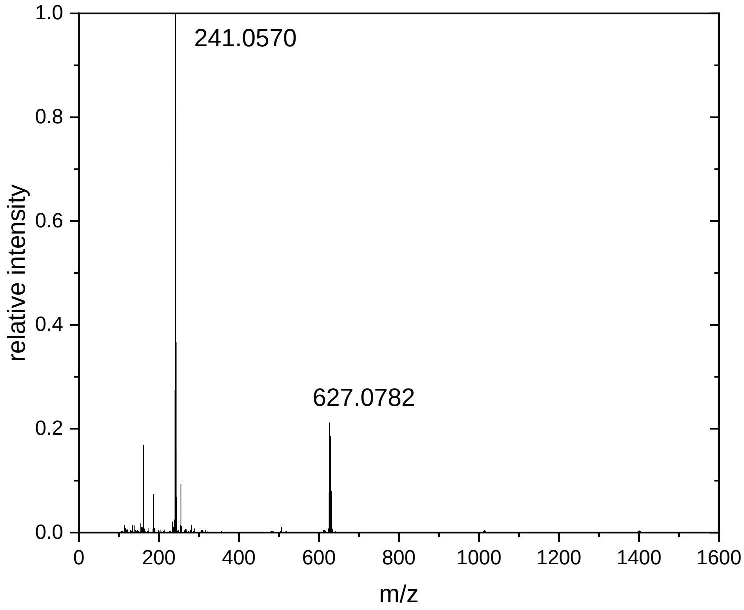

Figure 57. HR-ESI-MS spectrum of **PdL9**.

Au[(cC^{Et}CC^{Et}C)PF$_6$] (AuL9)

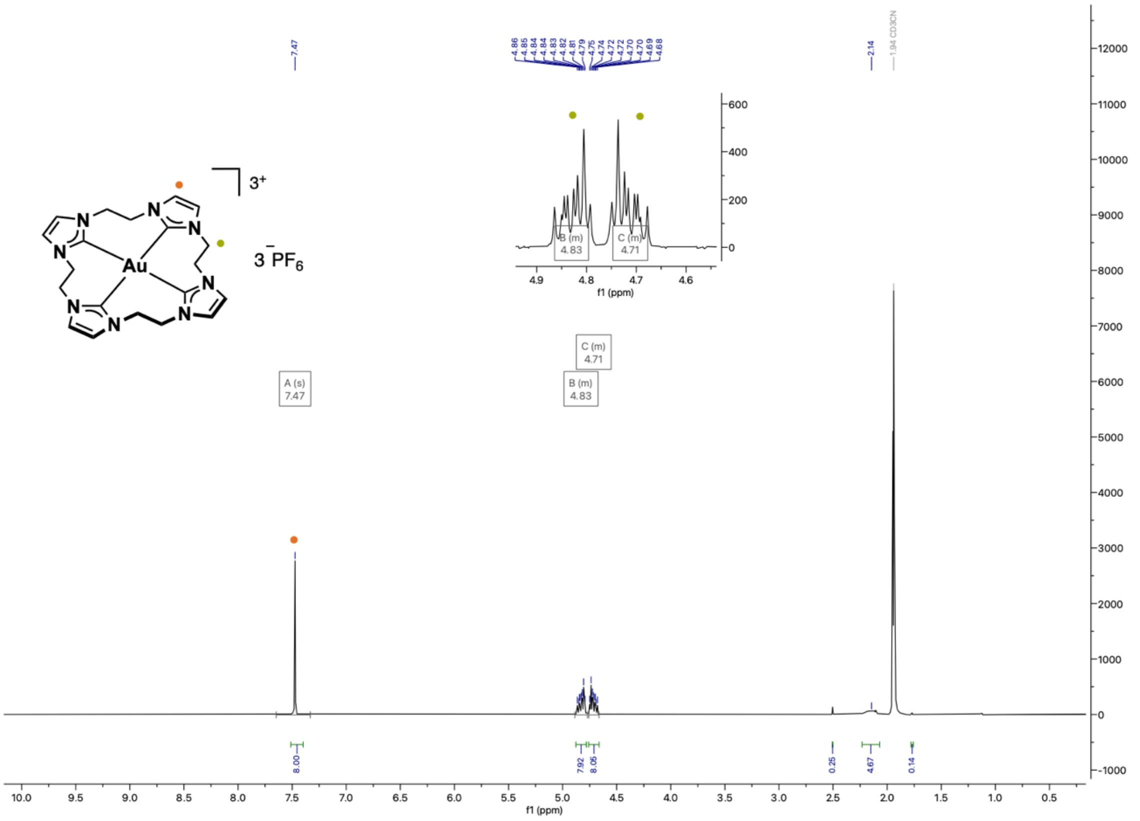

Figure 58: ^{1}H-NMR spectrum of **AuL9** in CD_3CN. With DMSO at 2.50 ppm and Water at 2.14 ppm as impuritys.

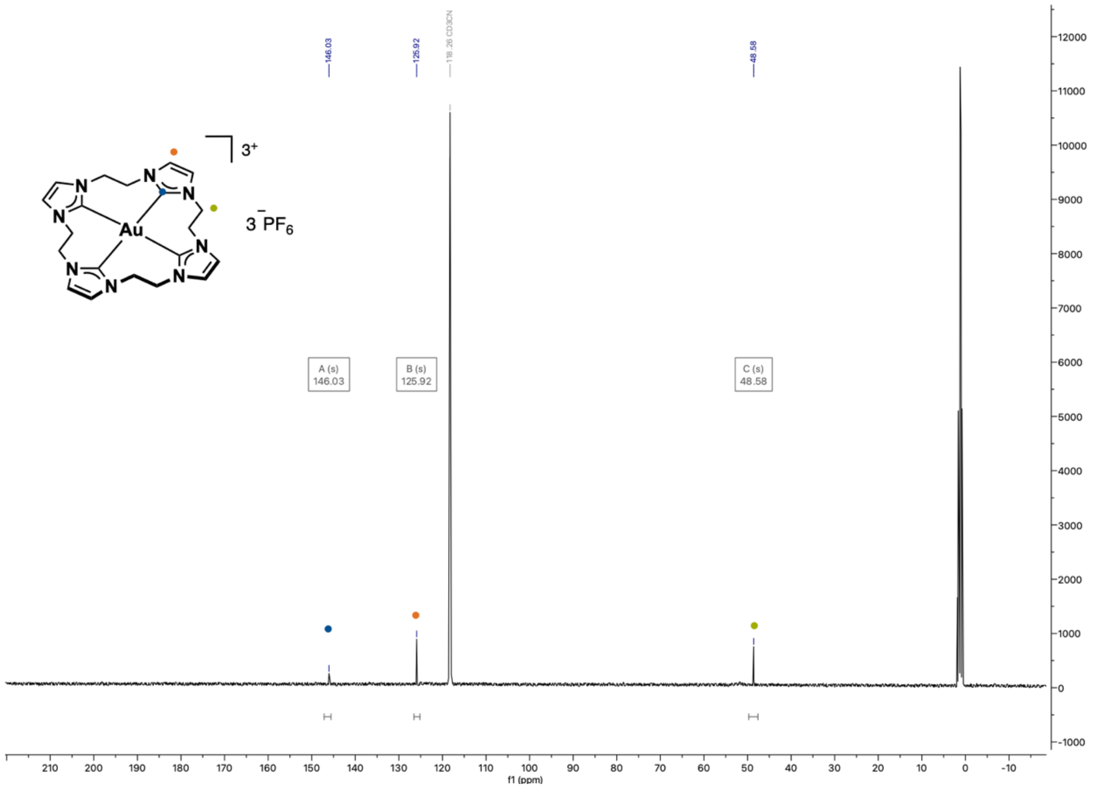

Figure 59: ^{13}C-NMR spectrum of **AuL9** in CD_3CN.

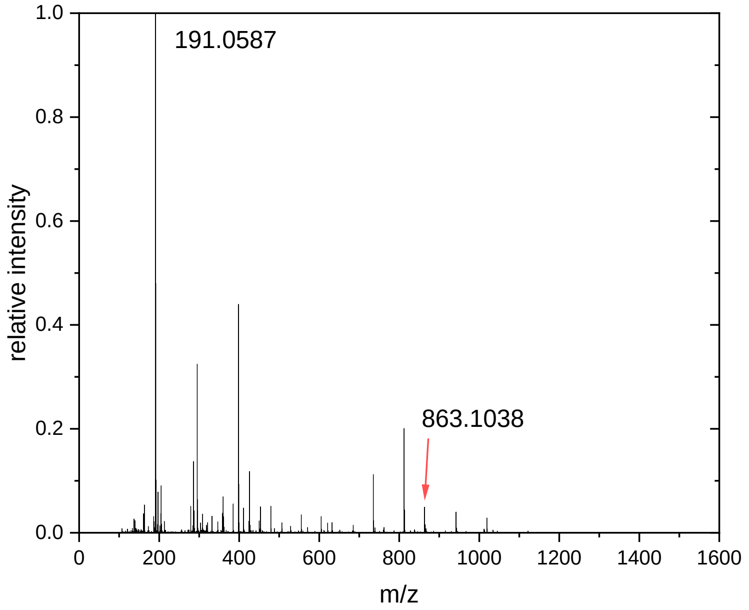

Figure 60. HR-ESI-MS spectrum of **AuL9**.

Pt[(cC^{Et}CC^{Et}C)OTf] (PtL8)

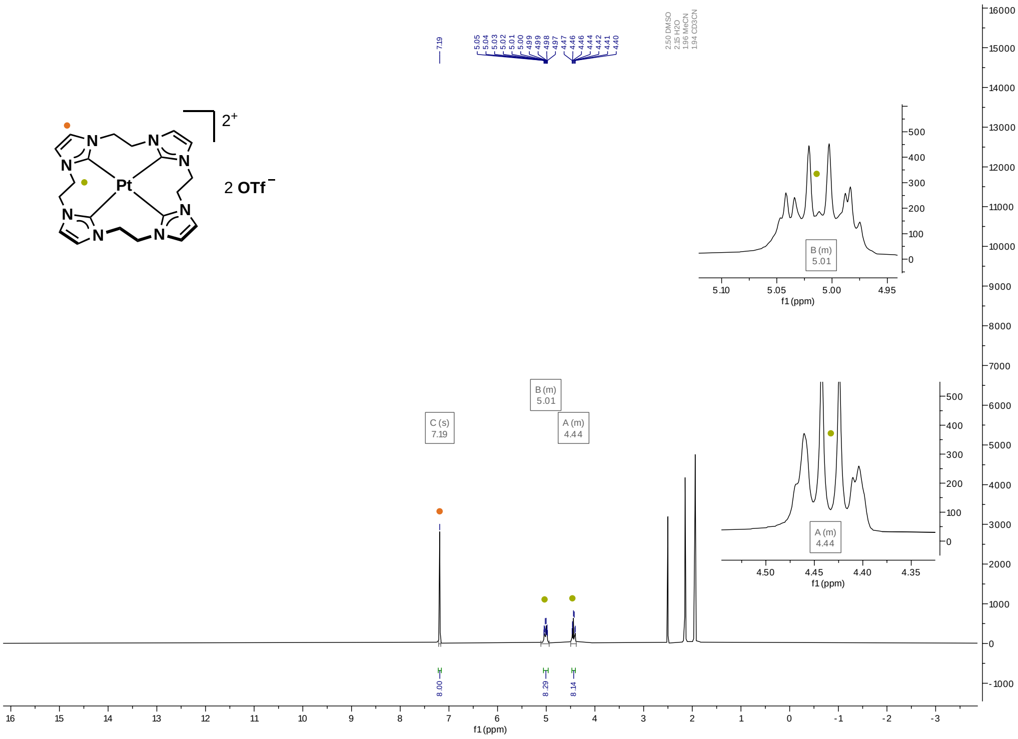

Figure 61: ^{1}H-NMR spectrum of **PtL8** in CD_3CN.

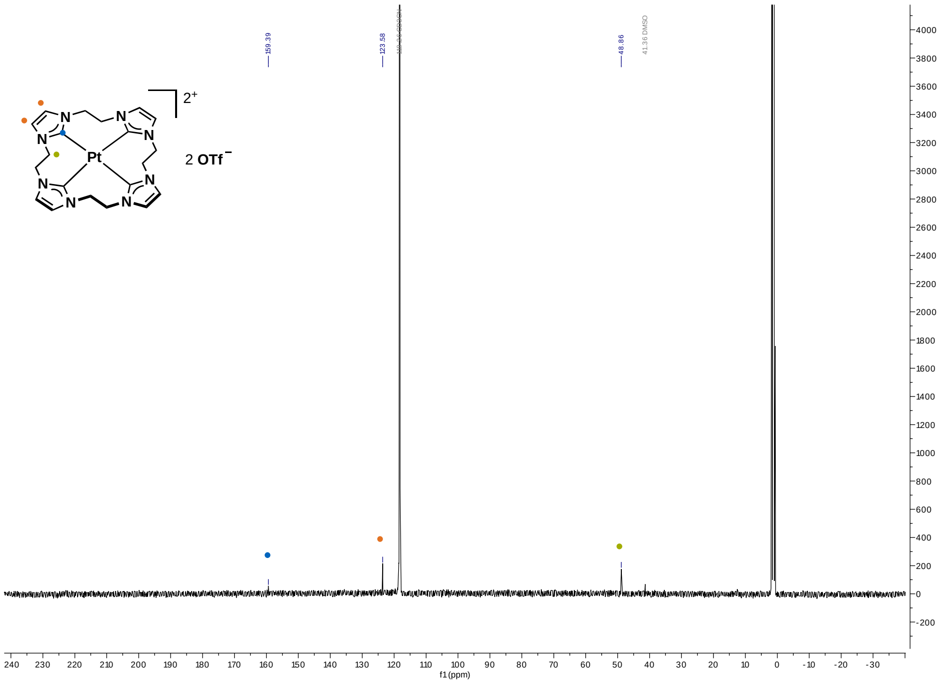

Figure 62: ^{13}C-NMR spectrum of **PtL8** in CD_3CN.

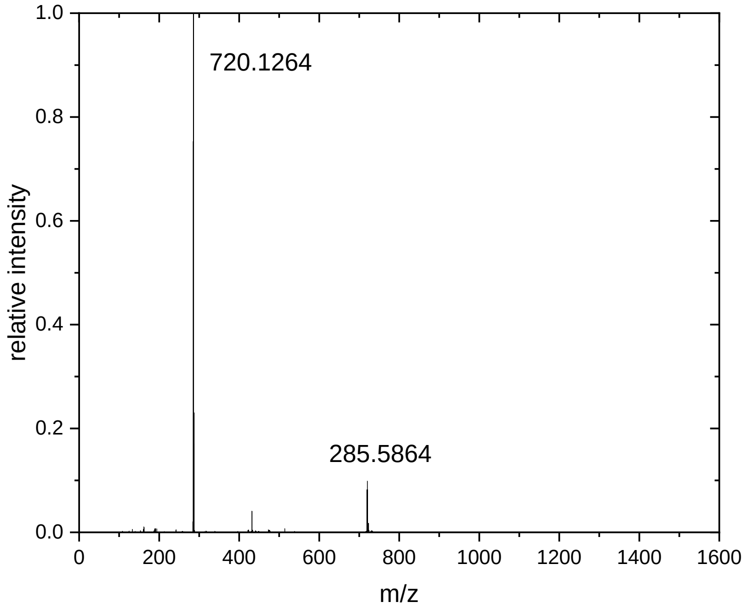

Figure 63. HR-ESI-MS spectrum of **PtL8**.

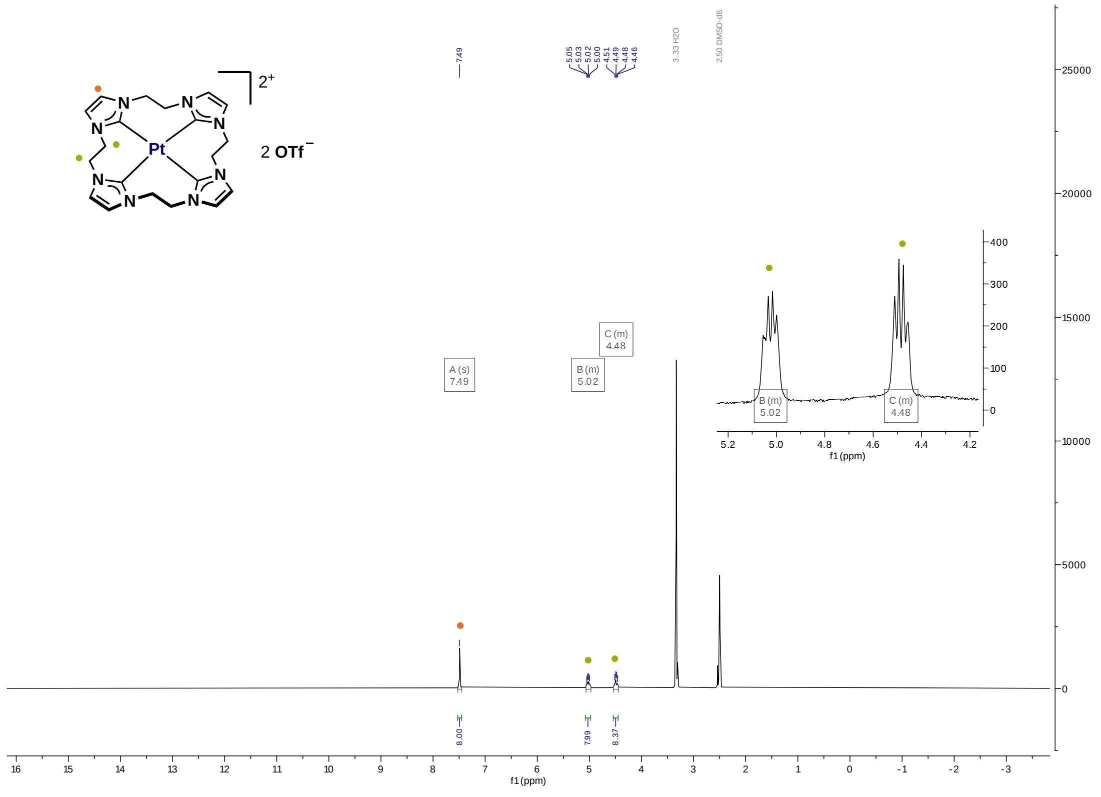

Figure 64. ¹H-NMR spectrum of **PtL8** in DMSO-d_6.

The analytic data of **10** to **17** are based on the master's thesis of T.P.S.[10]

2-Imidazoline (10)

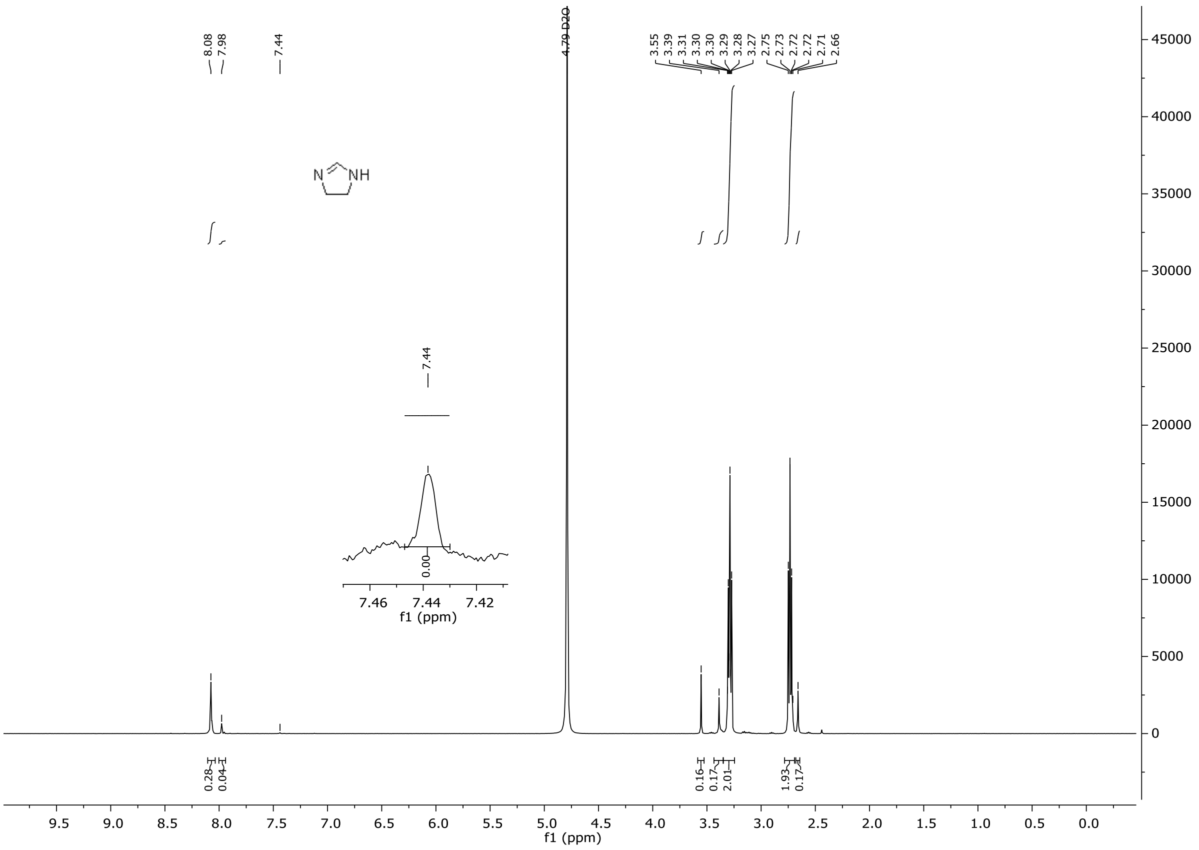

Figure 65. ^{1}H-NMR spectrum of **10** in D_2O.

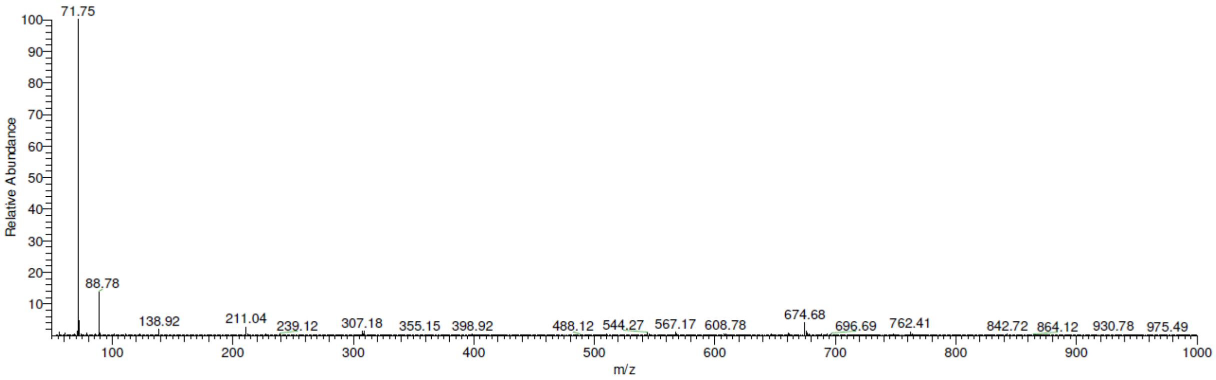

Figure 66. ESI-MS spectrum of **10**.

Synthetic attempts: bis(2-imidazolin-1-yl)methane (11)

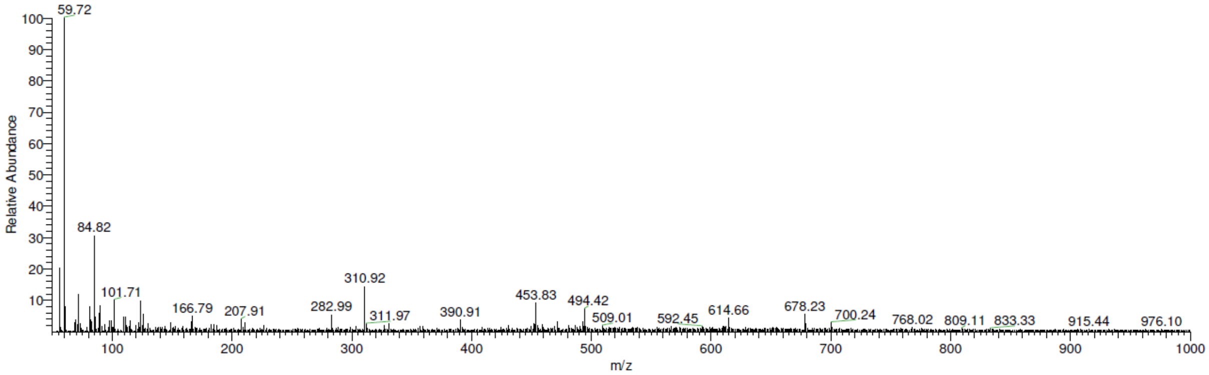

Figure 67. ESI-MS spectrum from the sublimed dried filtrate. The signal at 59.72 m/z can be attributed to acetamide (calculated m/z [M+H$^+$] = 60.04). The elemental analysis is also supporting this finding: Anal. calcd. for C_2H_5NO: C 40.67; H 8.53; N 23.71. Found: C 39.81; H 8.45; N 22.73.

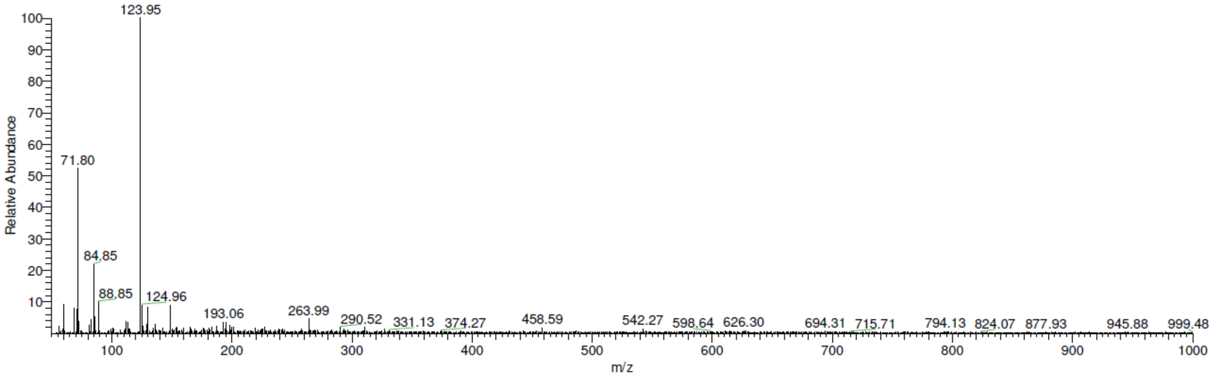

Figure 68. ESI-MS spectrum from the dried filtrate. The signal at 123.95 m/z can be assigned to *N*-acetylacetamide (calculated m/z [M+Na$^+$(sodium from sodium formate buffer used in the MS instrument)] = 124.04).

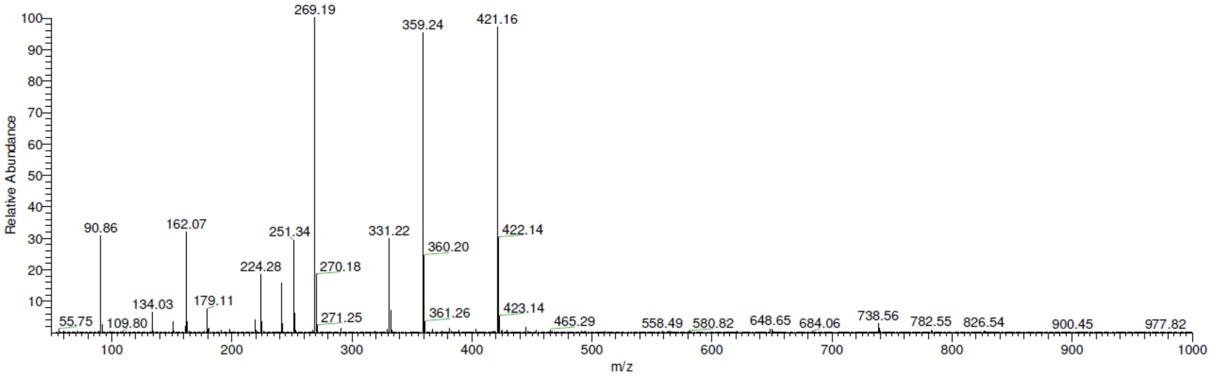

Figure 69. ESI-MS spectrum after extraction. Bis(benzyl) substituted **10** is present at m/z 251.34. **15** is present at m/z = 421.16 m/z.

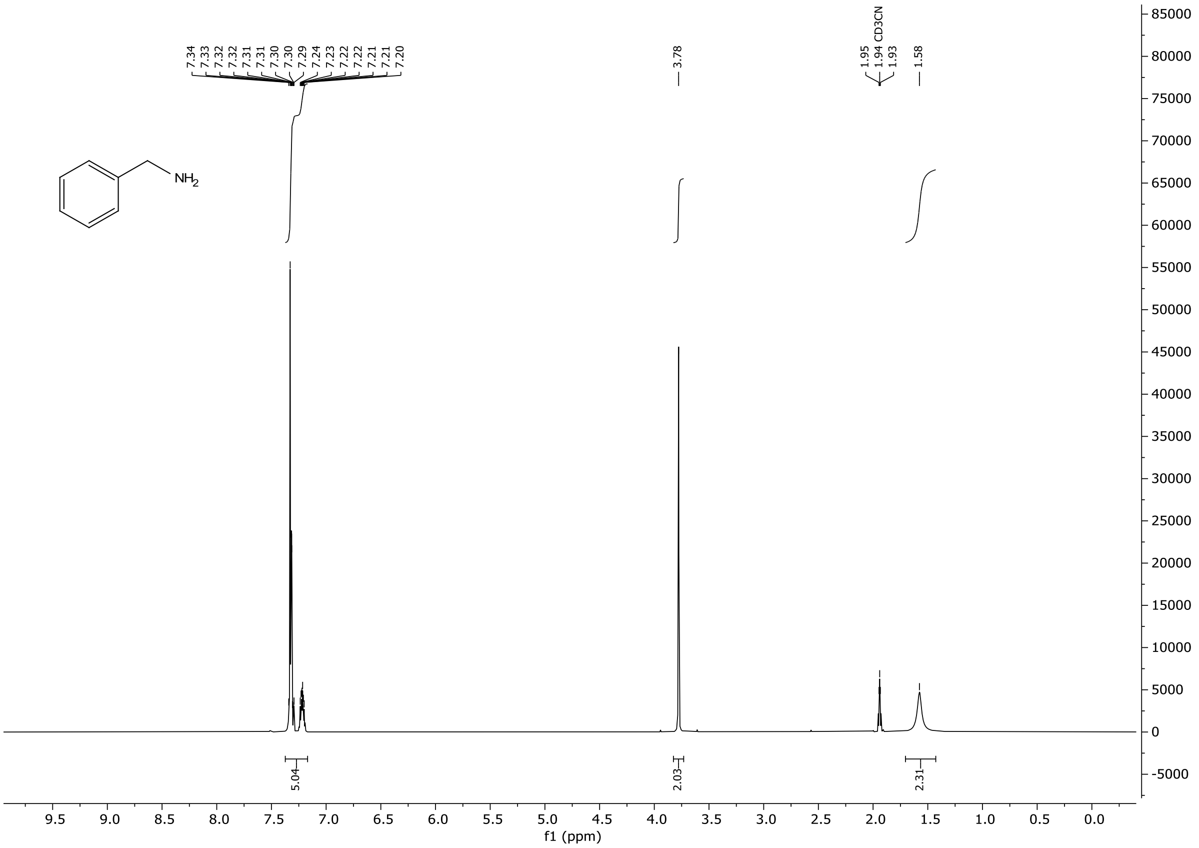

Figure 70. ^{1}H-NMR spectrum in CD_3CN after distillation. ^{1}H-NMR (400 MHz, CD_3CN): δ (ppm) = 7.30 (m, 5H, C_6H_5), 3.78 (s, 2H, CH_2), 1.58 (s, 2H, NH_2).

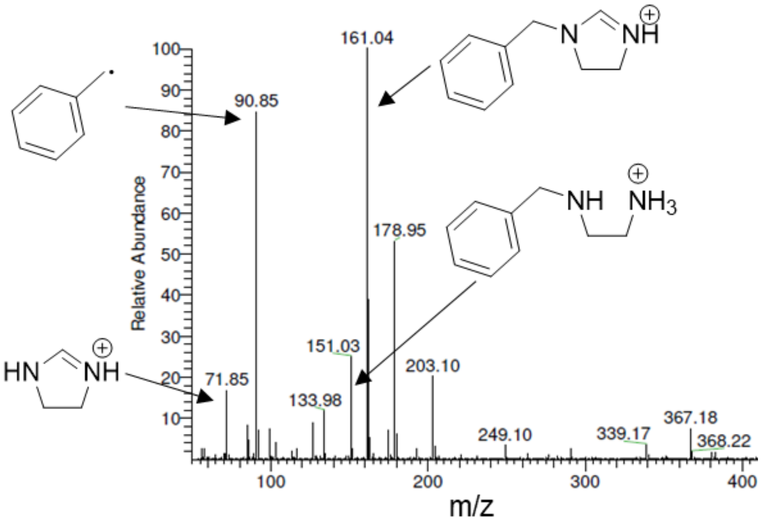

Figure 71. ESI-MS spectrum of the dried filtrate after 20 h.

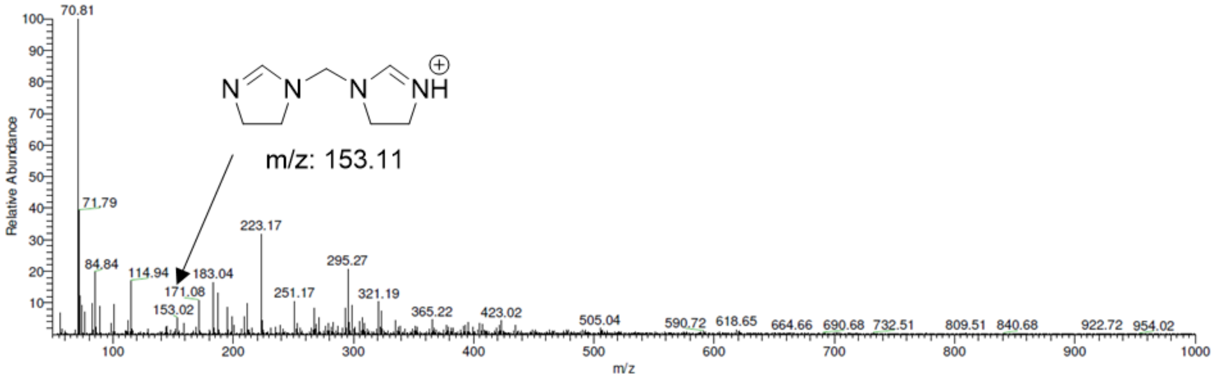

Figure 72. ESI-MS spectrum of **S1** from the reaction mixture.

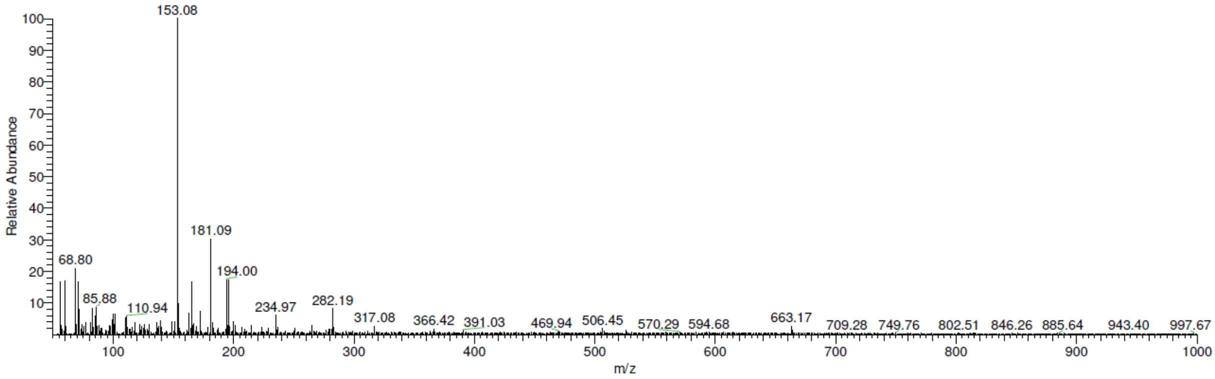

Figure 73. ESI-MS spectrum of **S2** from the dried yellow crude after reaction in MeCN.

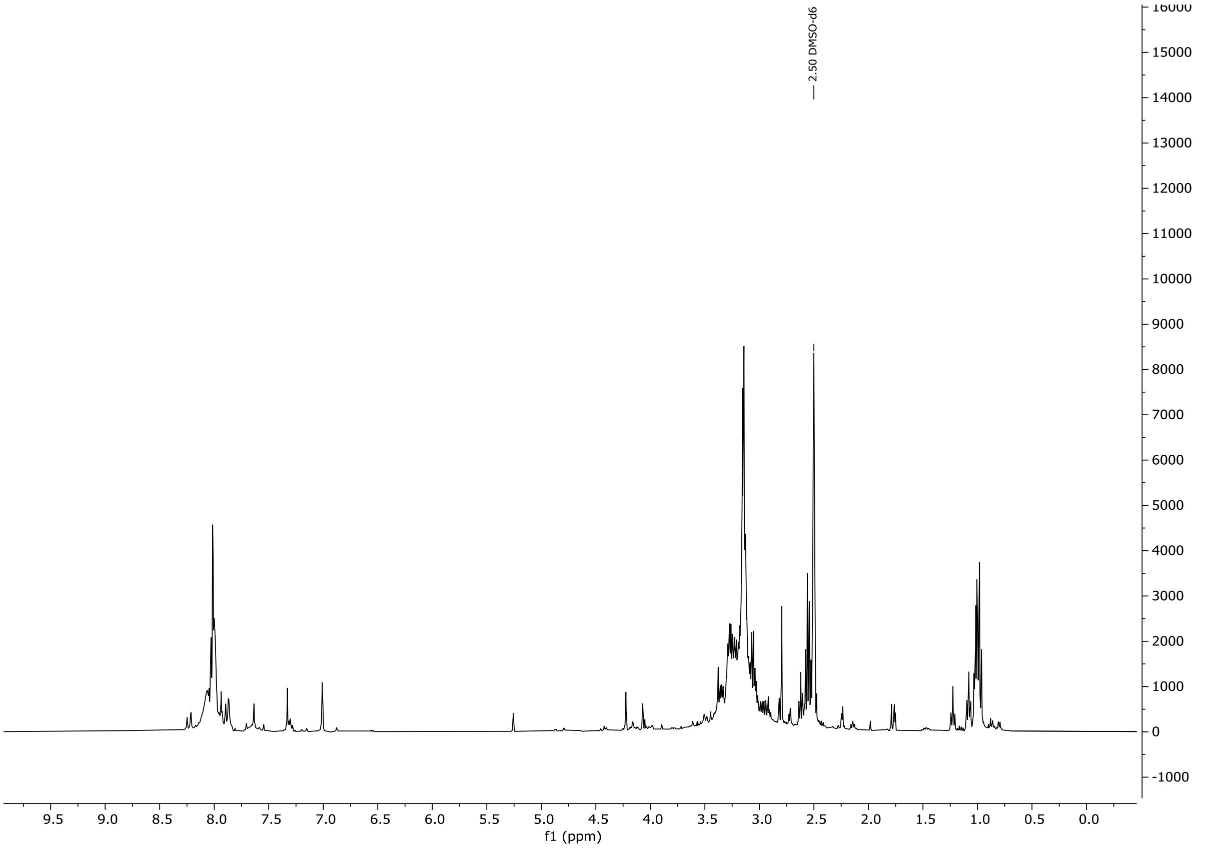

Figure 74. ^{1}H-NMR spectrum of **S2** in DMSO-d_6 from the dried extract after extraction of the aqueous phase (pH set to ~10) with EtOAc.

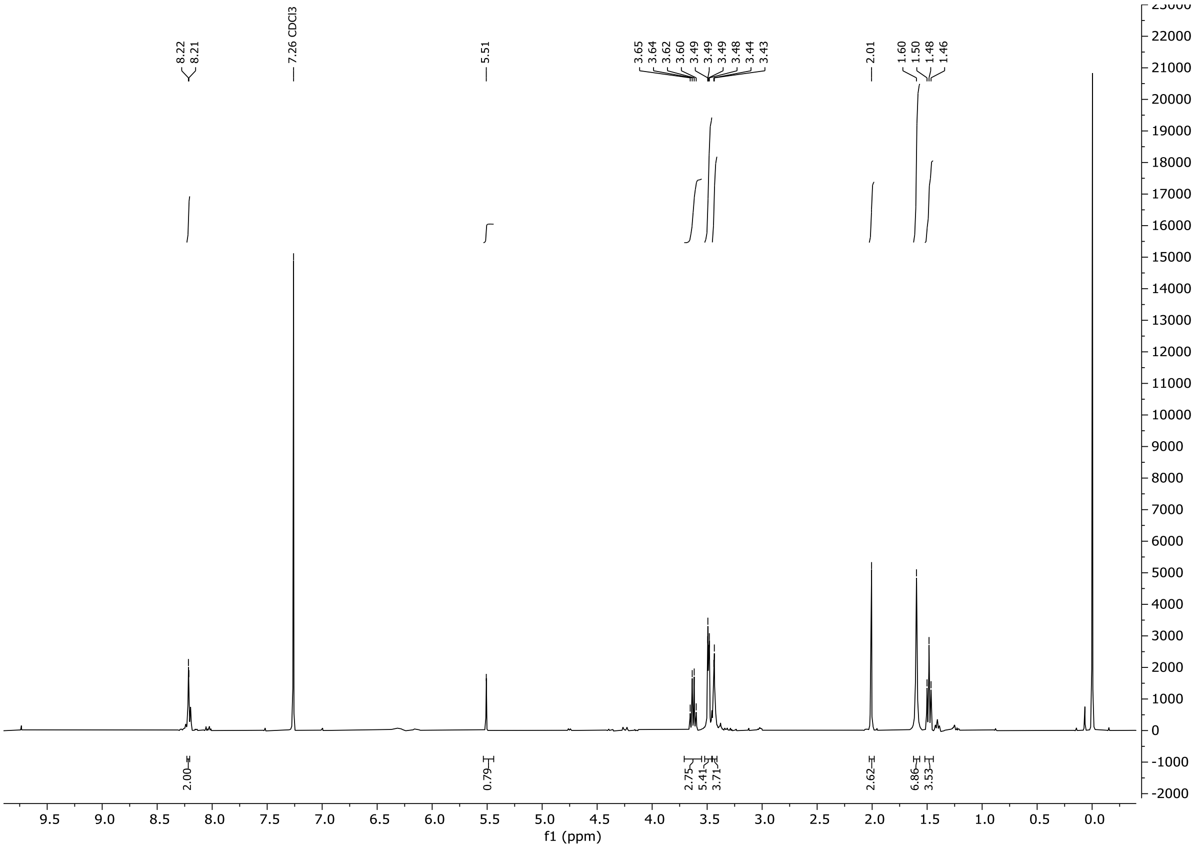

Figure 75. ^{1}H-NMR spectrum of **S2** in $CDCl_3$ from the dried first fraction after column chromatography of the dried aqueous phase (10 % MeOH in DCM (R_f = 0.27)). Peaks are assignable to a potential bis(2-imidazolin-1-yl)methane **11**.

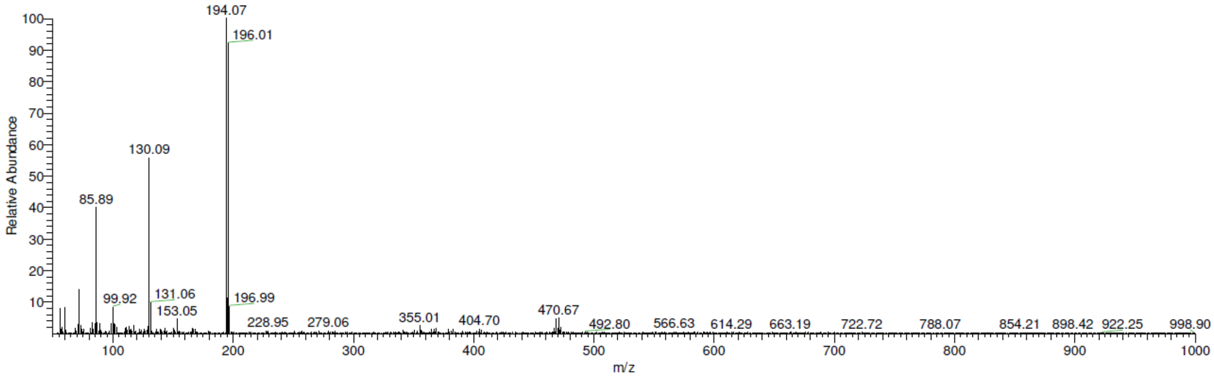

Figure 76. ESI-MS spectrum of **S2** from the dried first fraction after column chromatography.

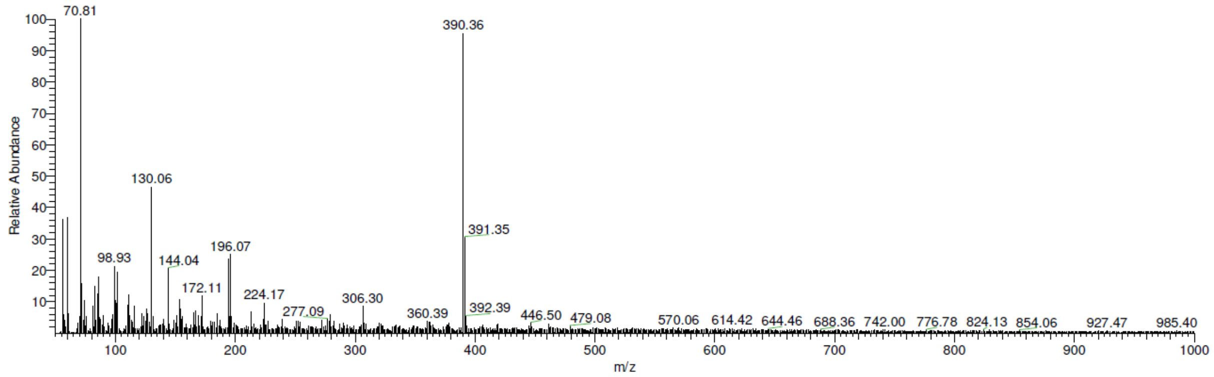

Figure 77. ESI-MS spectrum of **S3** from the dried crude in water.

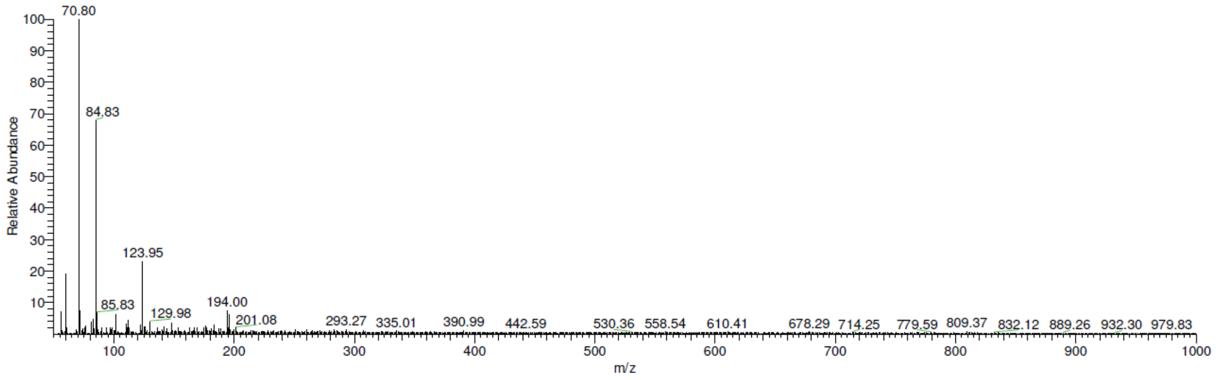

Figure 78. ESI-MS spectrum from the dried crude in MeCN. 3-(imidazolin-1-yl)propanenitrile (123.95 m/z) as potential side product.

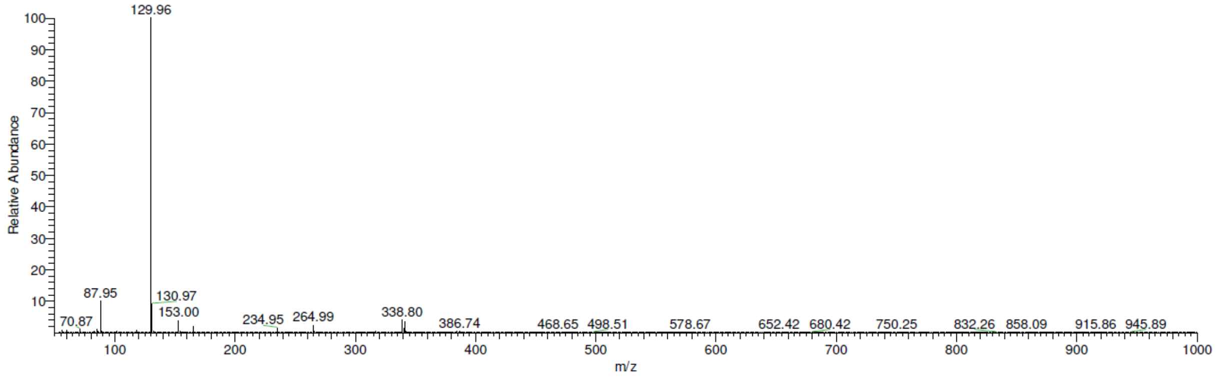

Figure 79. ESI-MS spectrum of **S4** from the dried crude in MeCN.

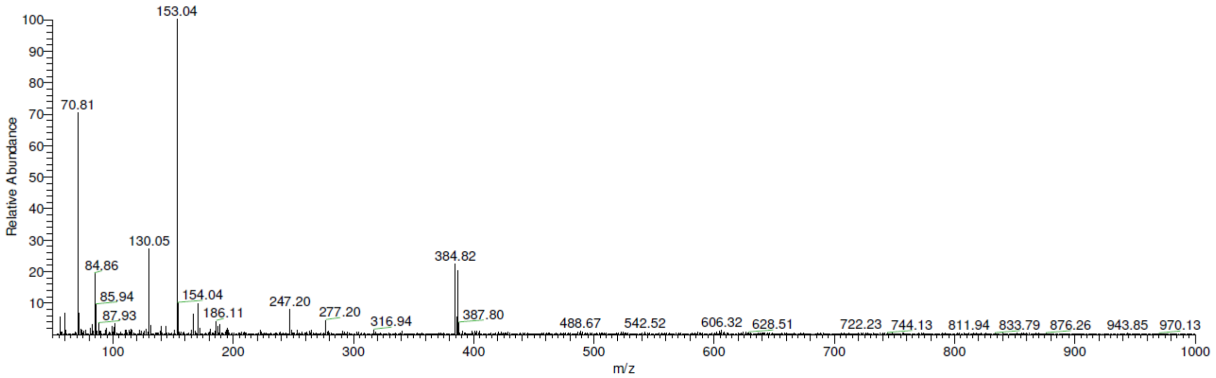

Figure 80. ESI-MS spectrum of **S4** from the second fraction after column chromatography (from DCM:MeCN:MeOH 7:2:1, R_f = 0.27 (of DIPEA · HBr) to DCM:MeOH 2:1). DIPEA hydrobromide salt is visible (calculated m/z [M + H^+ – Br^-] = 130.16) and remaining **10**.

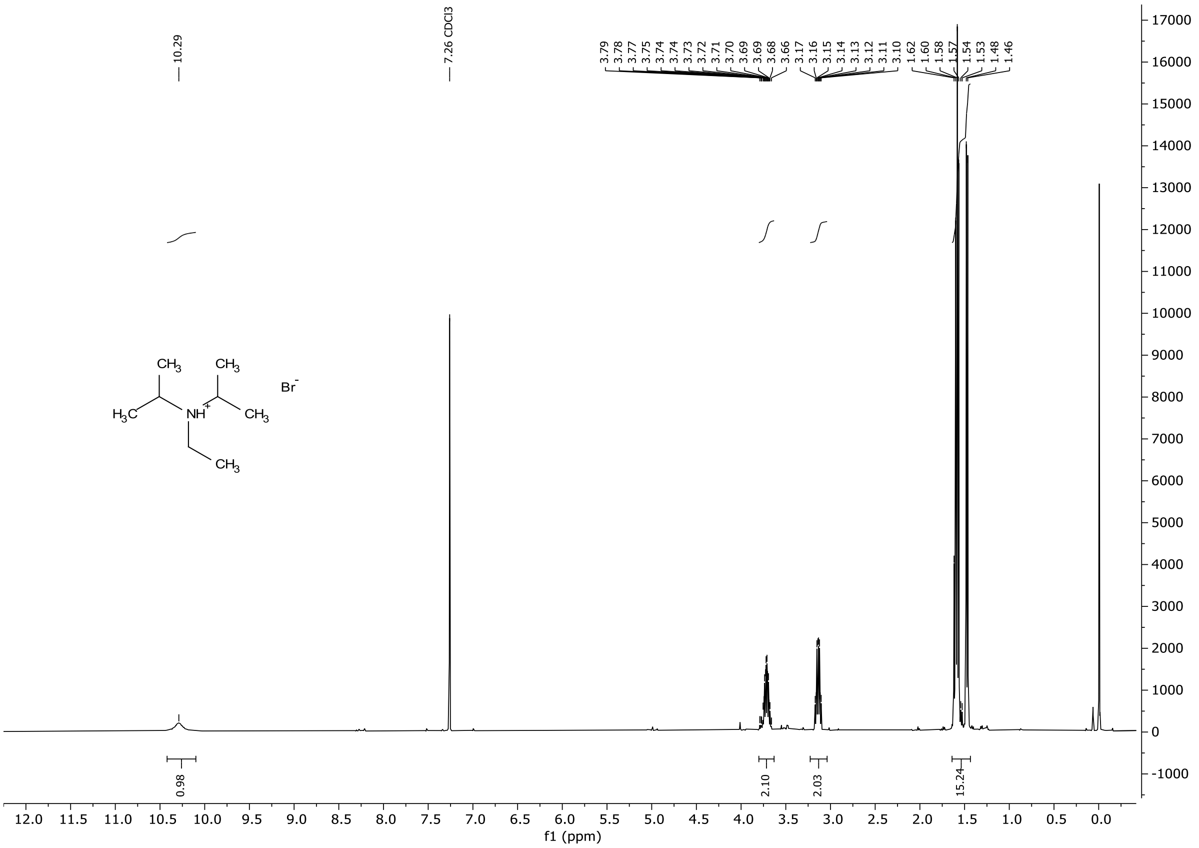

Figure 81. ^{1}H-NMR spectrum of **S5** in $CDCl_3$ from the white sublimed solid (DIPEA · HBr). ^{1}H-NMR (400.13 MHz, $CDCl_3$): δ (ppm) = 10.29 (s, 1H, NH), 3.72 (pd, 2H, CH, $^3J = 6.7$ Hz, $^3J = 4.2$ Hz), 3.14 (qd, 2H, CH_2, $^3J = 7.4$ Hz, $^3J = 4.4$ Hz), 1.55 (m, 15H, CH_3).

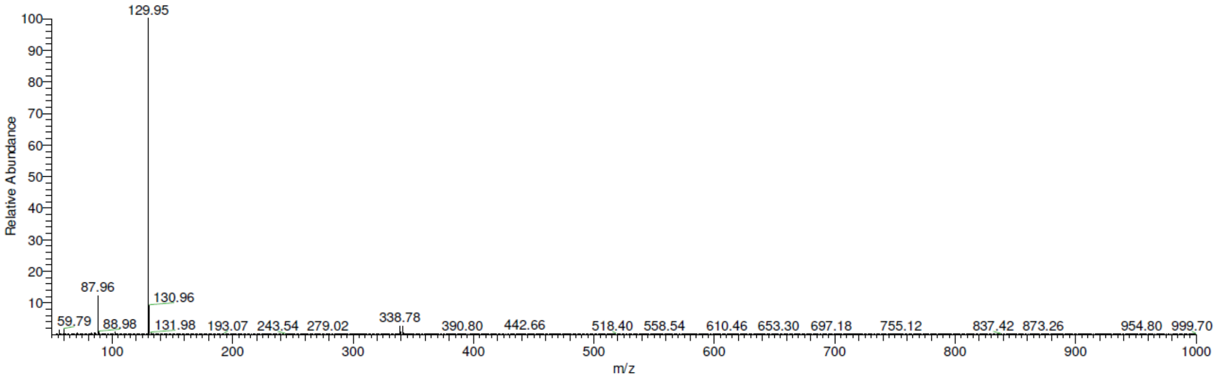

Figure 82. ESI-MS spectrum of **S5** from the white sublimed solid (DIPEA · HBr). Positive ionization mode. Calculated m/z $[M + H^+ - Br^-] = 130.16$.

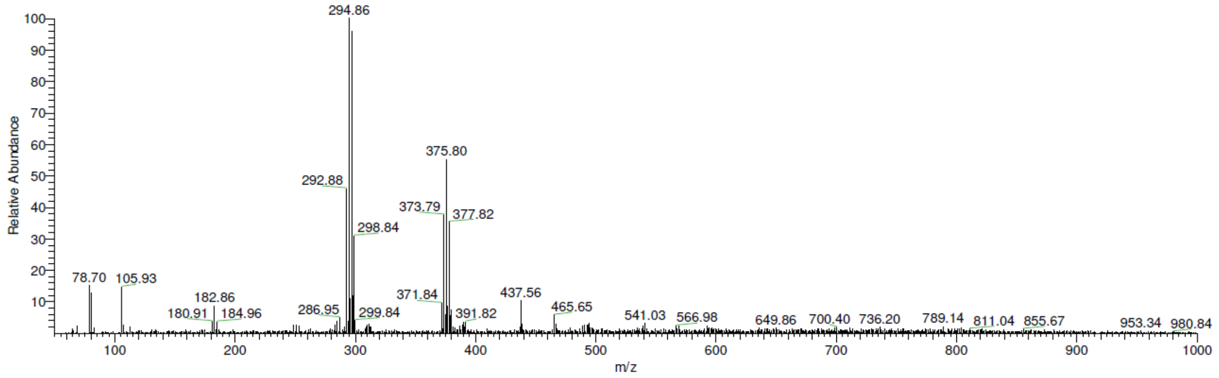

Figure 83. ESI-MS spectrum of **S5** from the white sublimed solid (DIPEA · HBr). Negative ionization mode. Calculated m/z $[Br^-]$ = 78.92 (100); 80.92 (97).

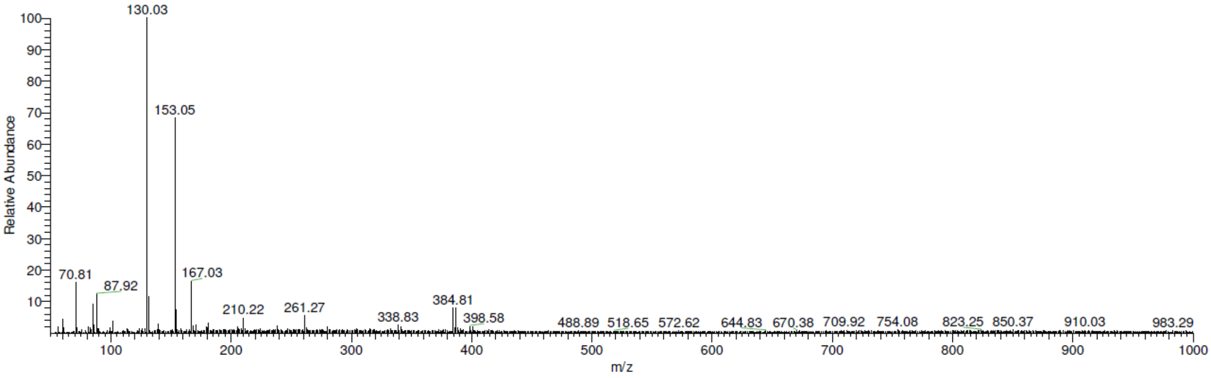

Figure 84. ESI-MS spectrum of **S5** from the black residue after sublimation.

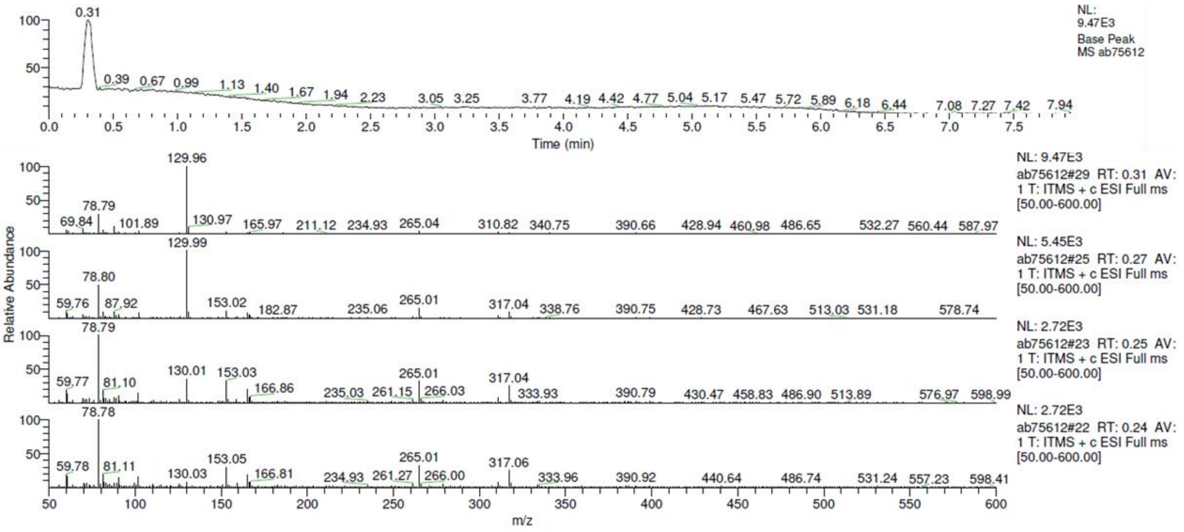

Figure 85. ESI-MS spectrum of **S5** from the crude product using a reversed phase column. The retention times (RT) are given on the right side of each mass spectrum. While the signal of bis(2-imidazolin-1-yl)methane **11** appears at 0.24 min indicating a higher polarity than the DIPEA salt, already at 0.25 min DIPEA hydrobromide is visible and rising in the intensity until 0.27 min. At a retention time of 0.31 min, no signal from bis(2-imidazolin-1-yl)methane is present anymore.

N-Benzylethylenediamine (12)

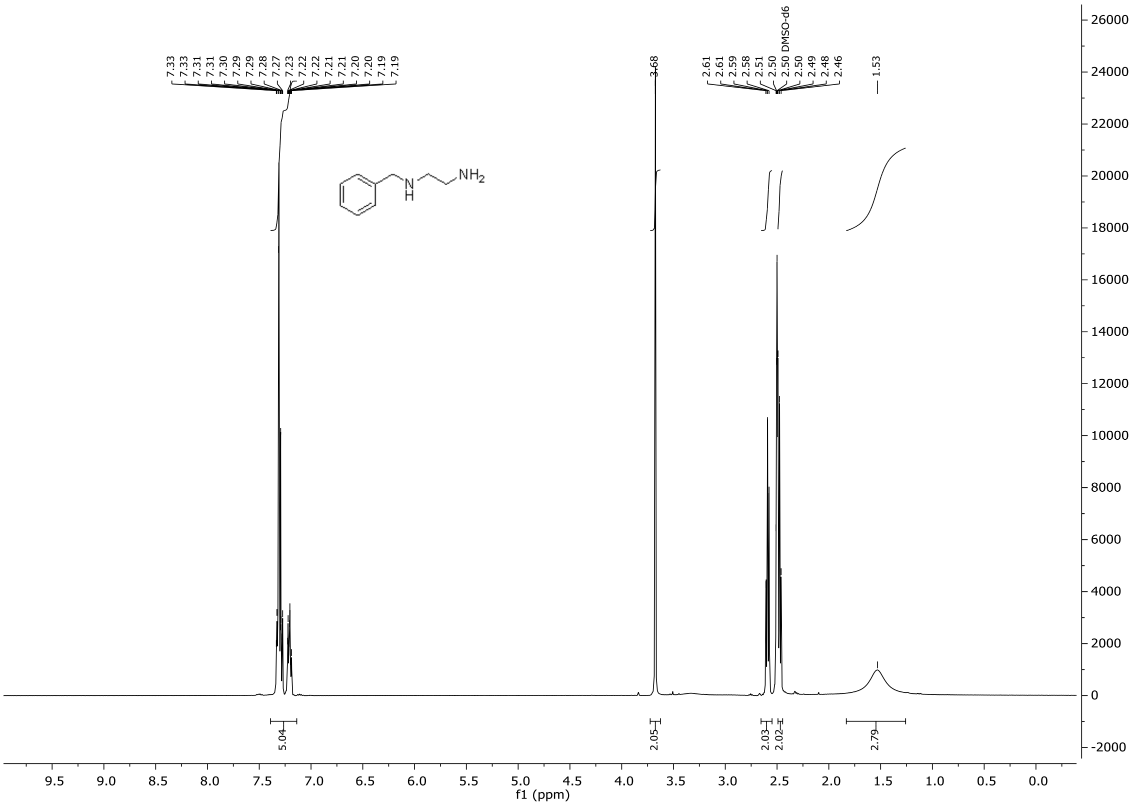

Figure 86. ^{1}H-NMR spectrum of **12** in DMSO-d$_6$.

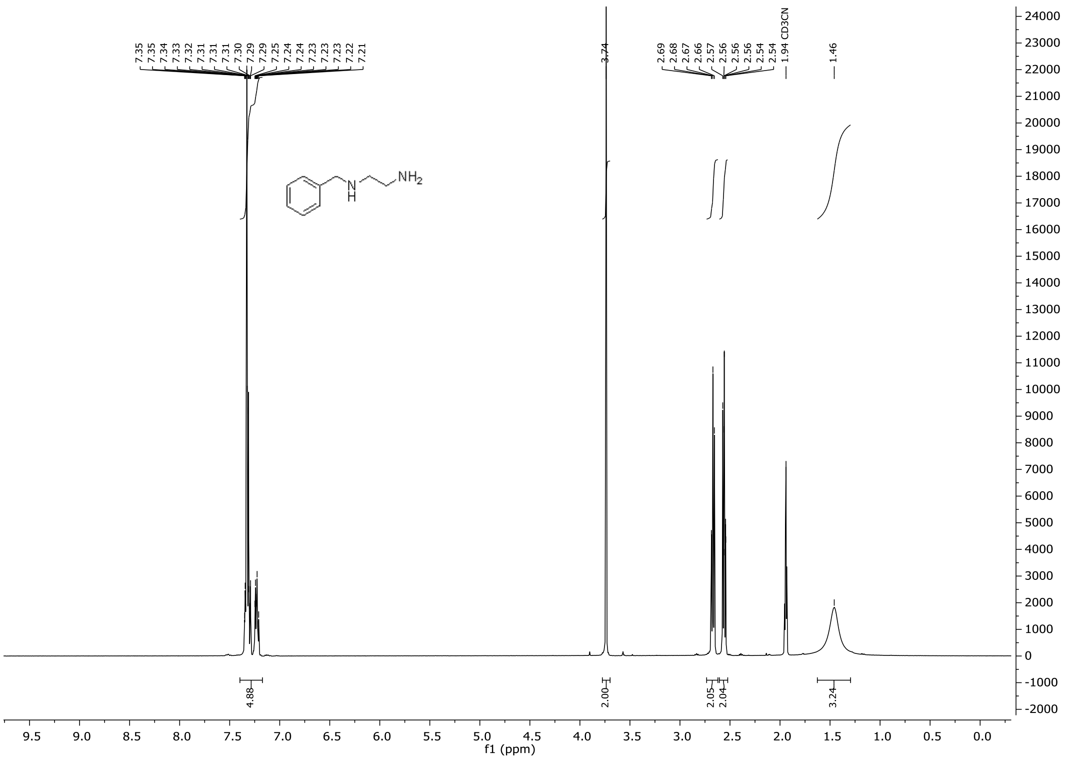

Figure 87. ^{1}H-NMR spectrum of **12** in CD_3CN.

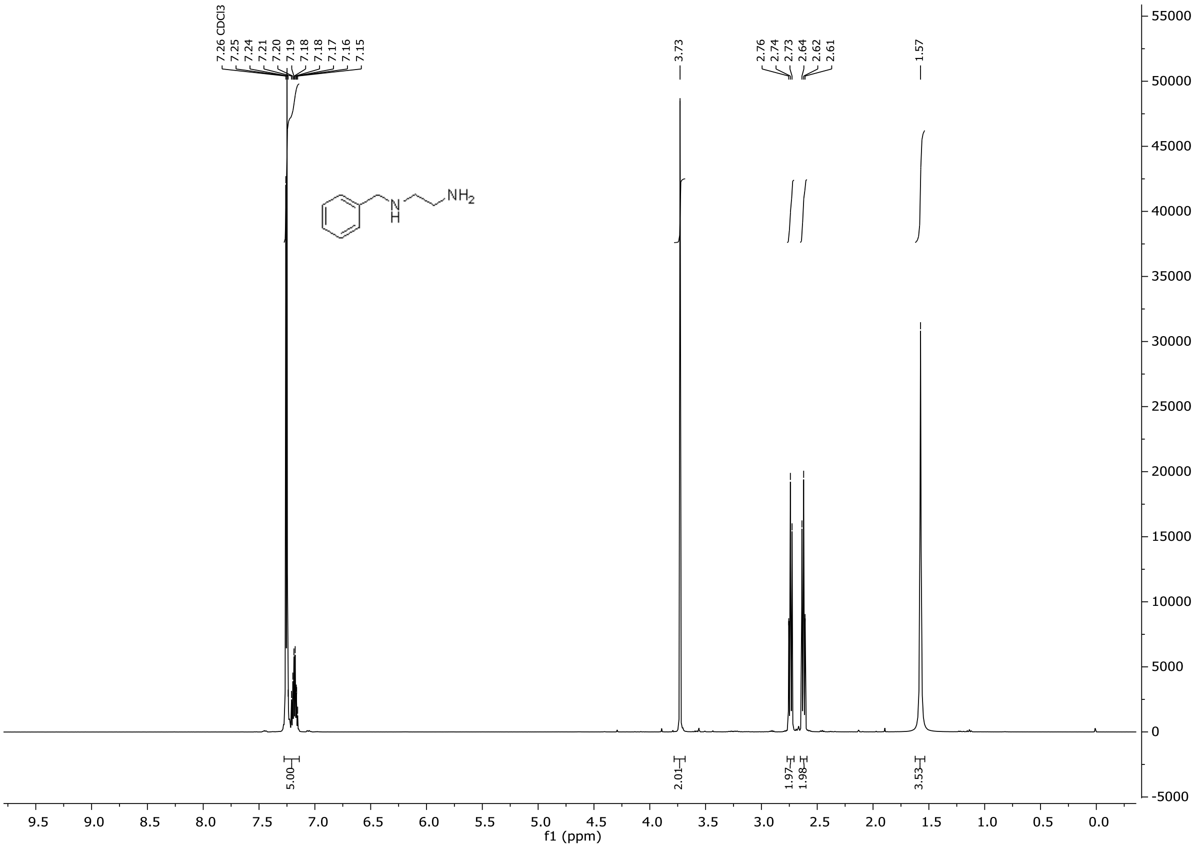

Figure 88. ^{1}H-NMR spectrum of **12** in $CDCl_3$.

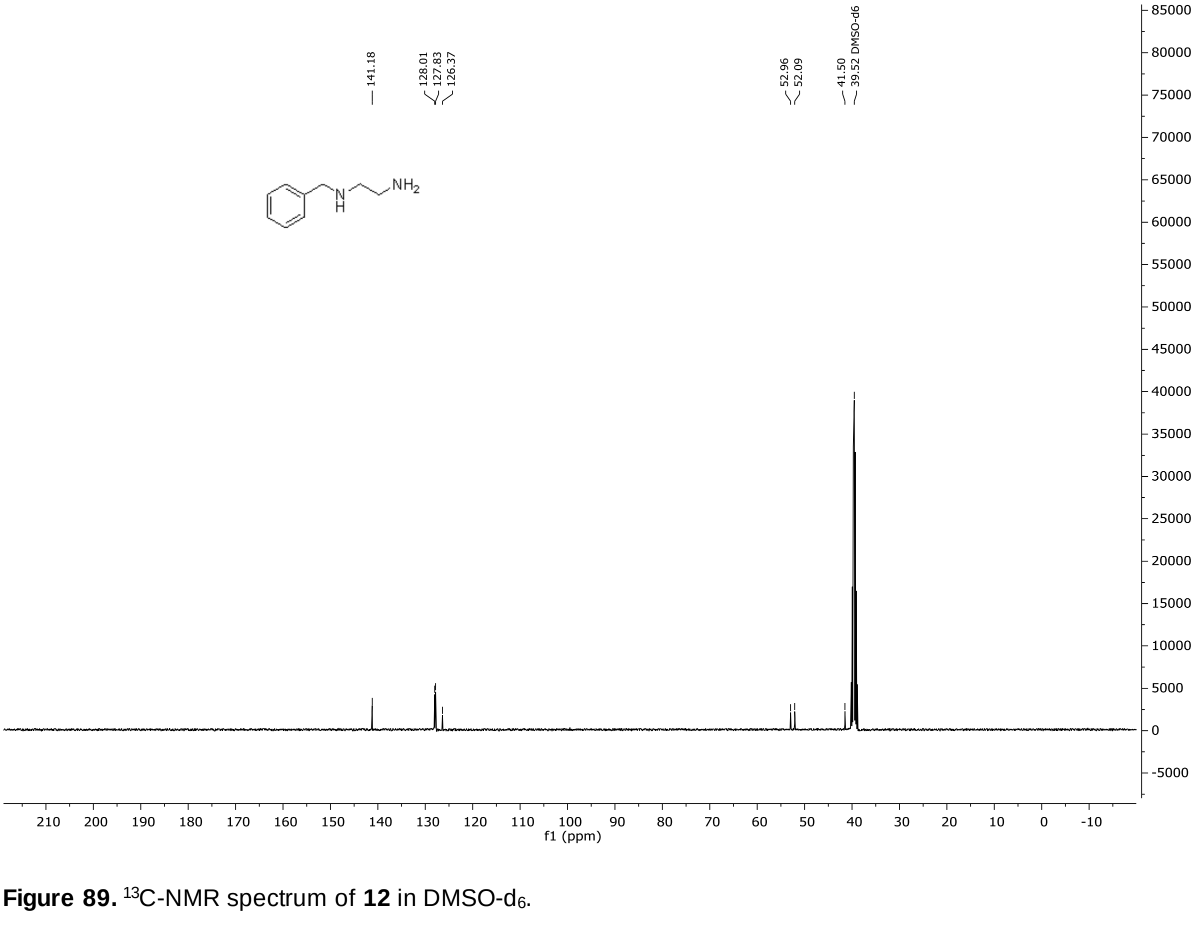

Figure 89. ^{13}C-NMR spectrum of **12** in DMSO-d_6.

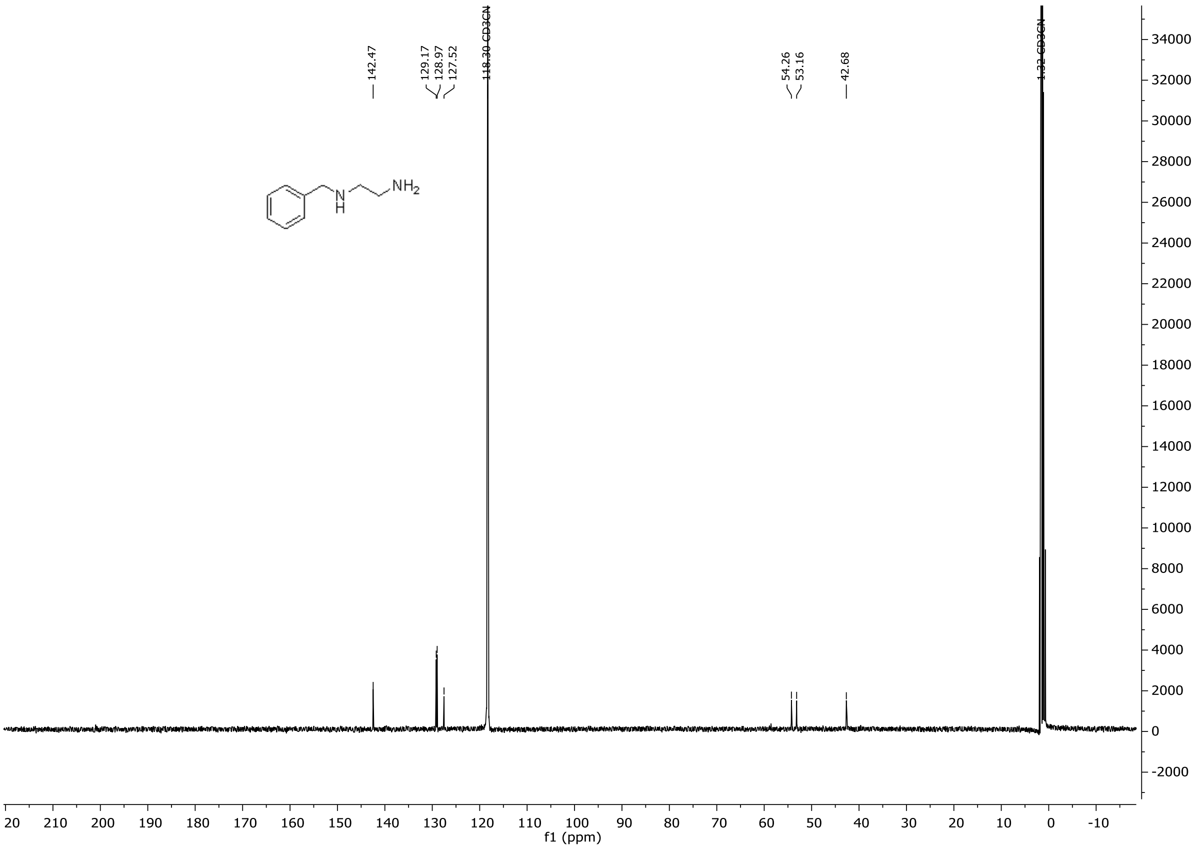

Figure 90. ^{13}C-NMR spectrum of **12** in CD_3CN.

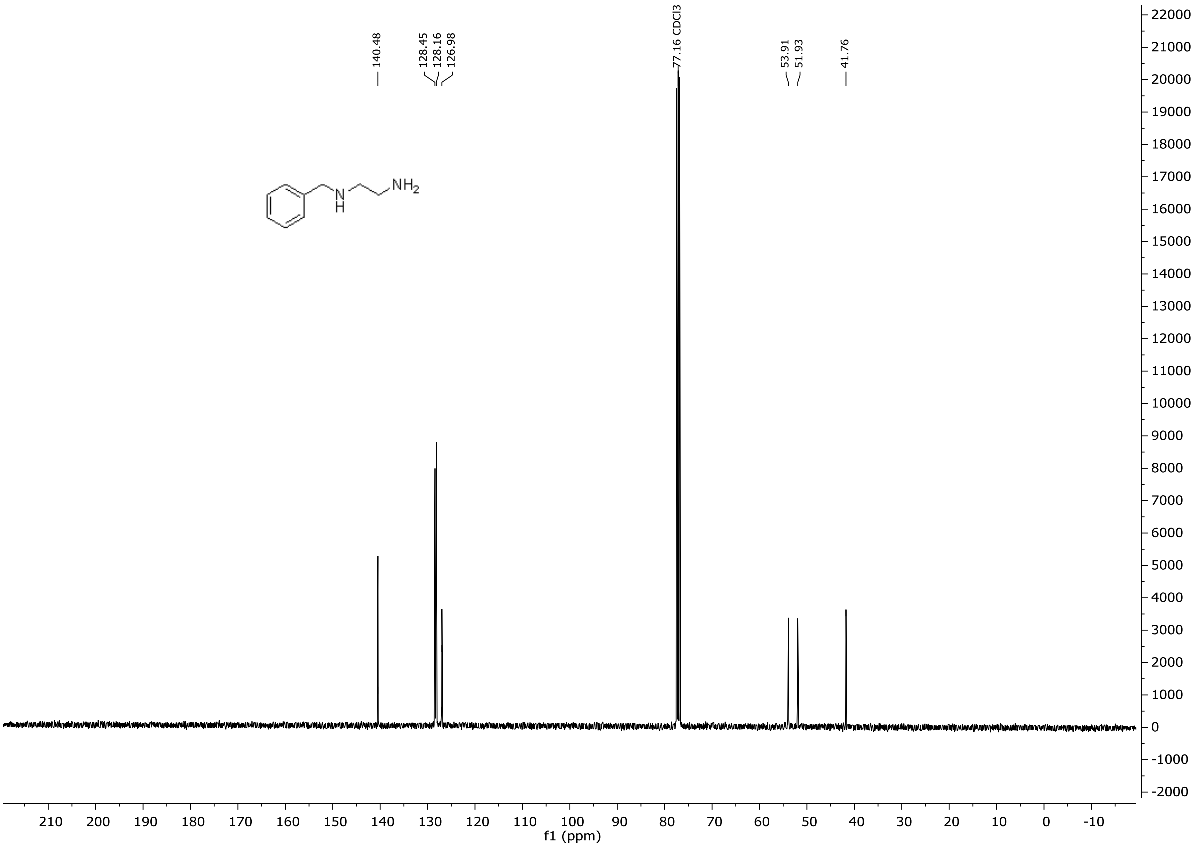

Figure 91. ^{13}C-NMR spectrum of **12** in $CDCl_3$.

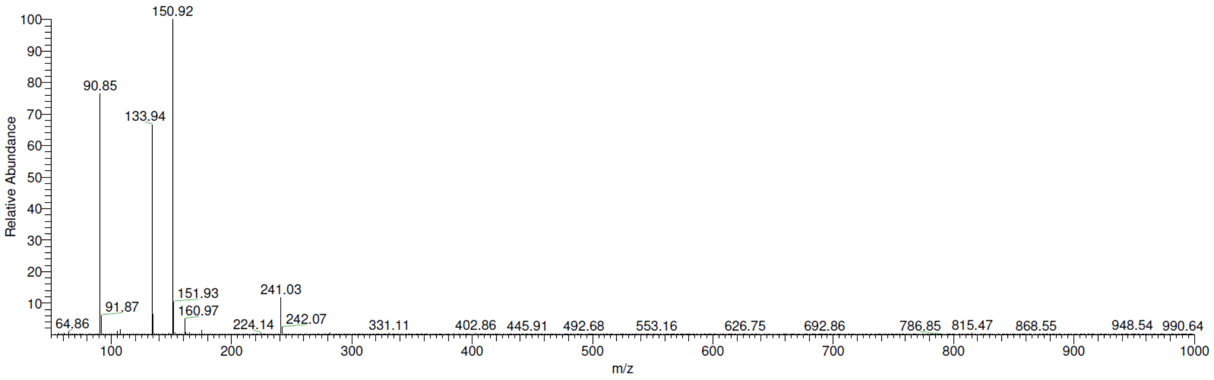

Figure 92. ESI-MS spectrum of **12**.

N-Benzyl-2-imidazoline (13)

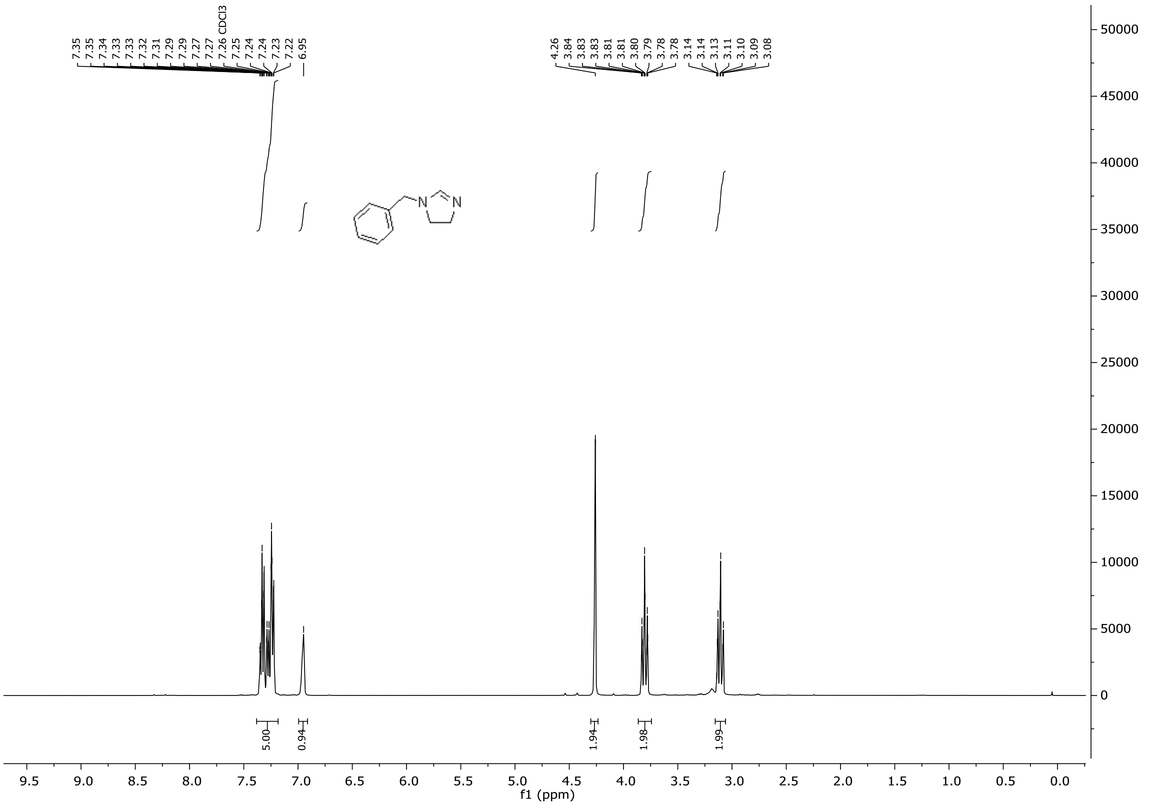

Figure 93. ^{1}H-NMR spectrum of **13** in $CDCl_3$.

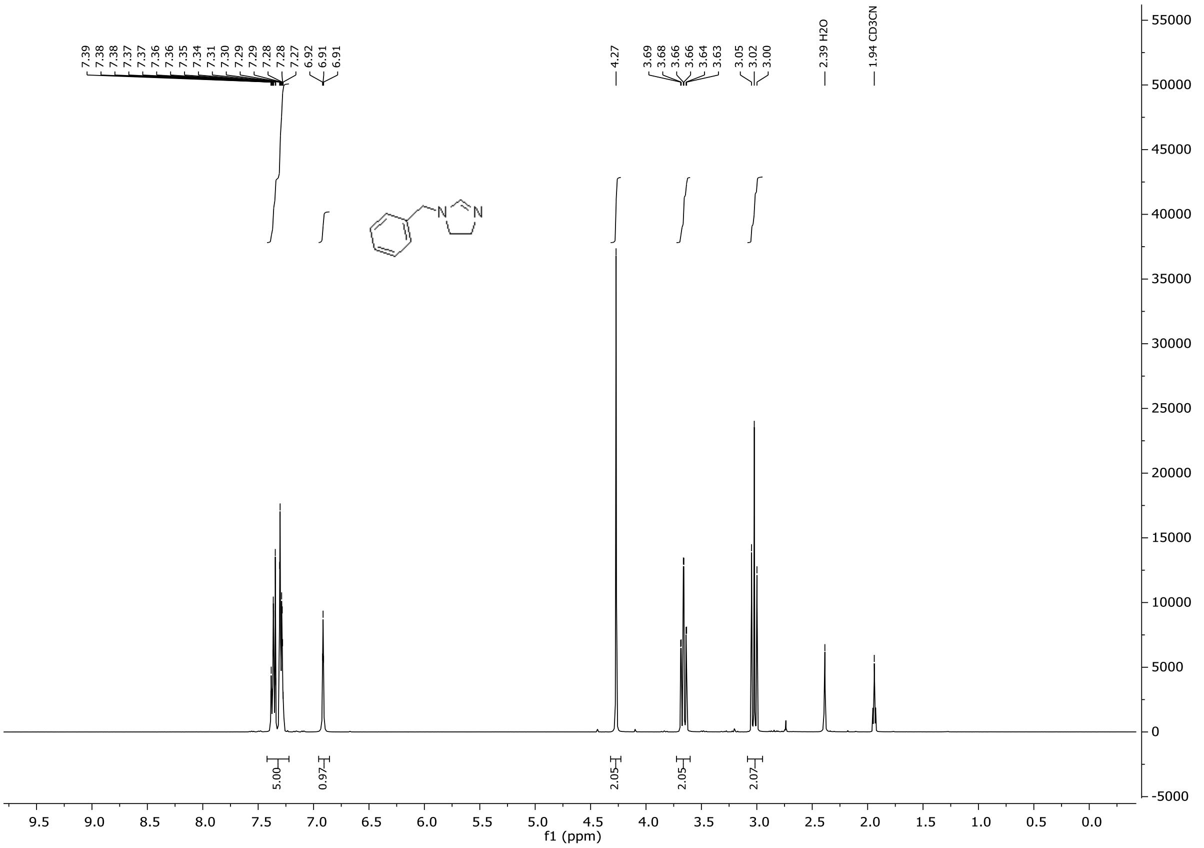

Figure 94. ^{1}H-NMR spectrum of **13** in CD_3CN.

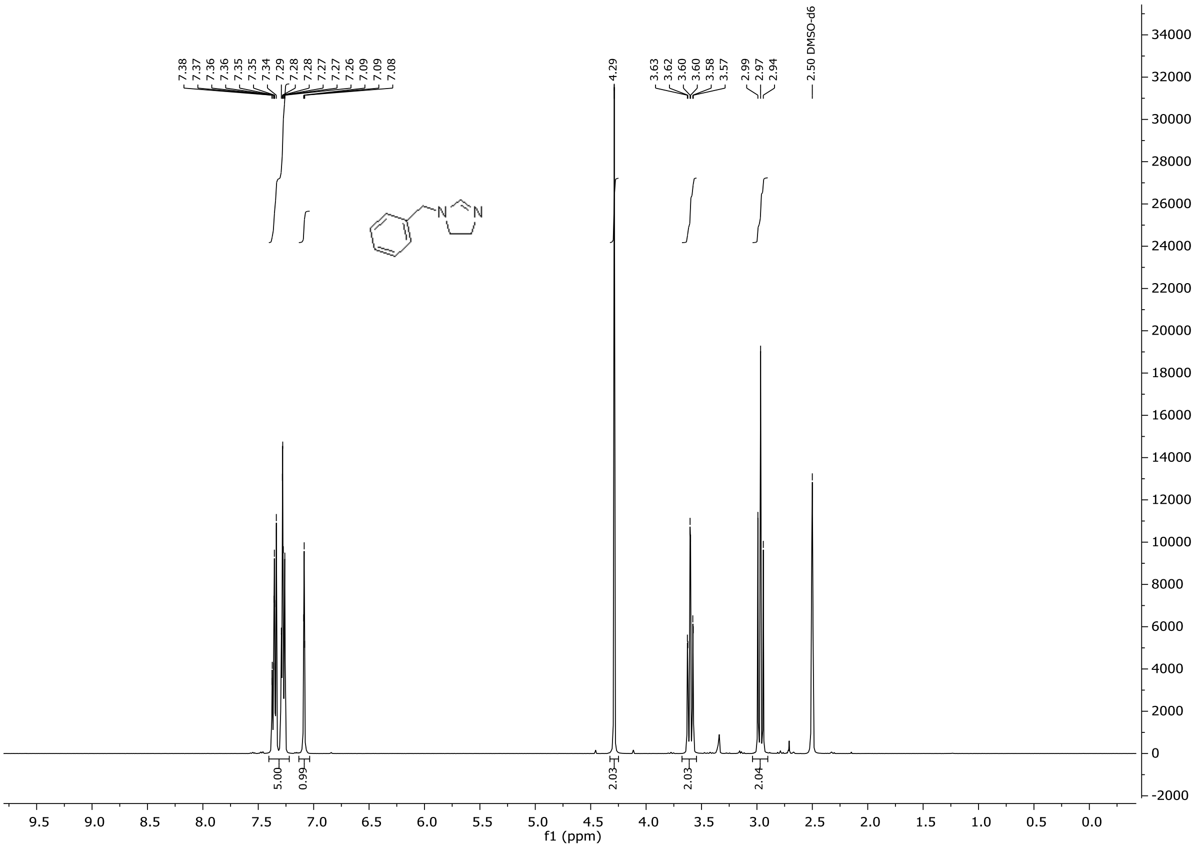

Figure 95. ^{1}H-NMR spectrum of **13** in DMSO-d_6.

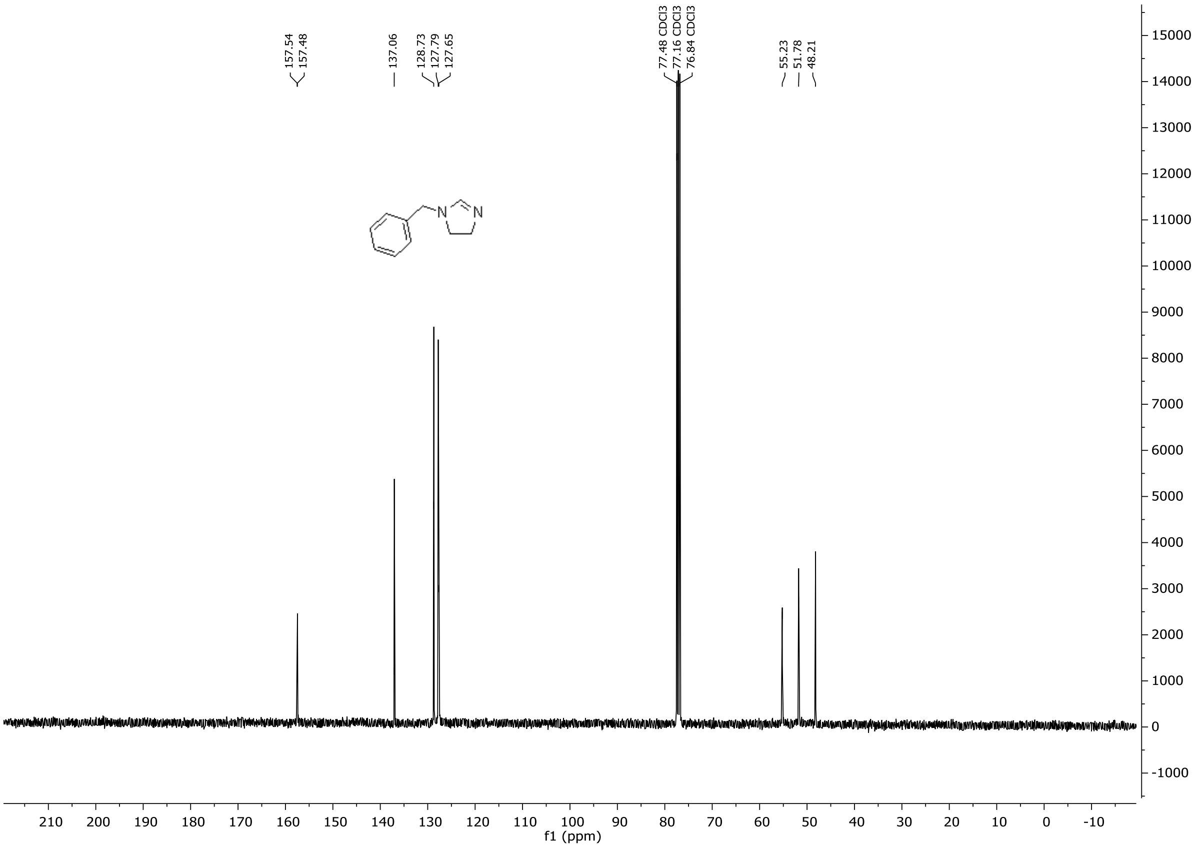

Figure 96. ^{13}C-NMR spectrum of **13** in $CDCl_3$. The CH carbon atom appears as two signals at 157.51 ppm.

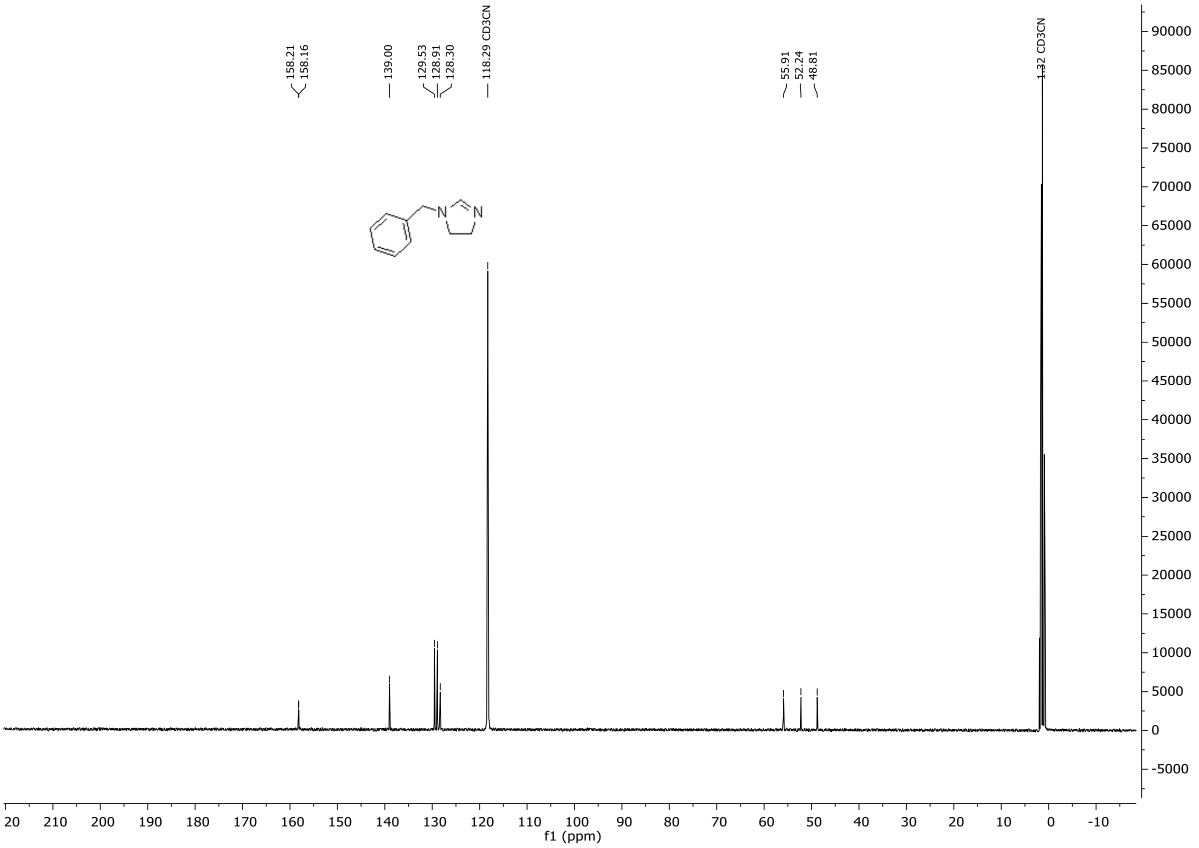

Figure 97. ^{13}C-NMR spectrum of **13** in CD_3CN. The CH carbon atom appears as two signals at 158.19 ppm.

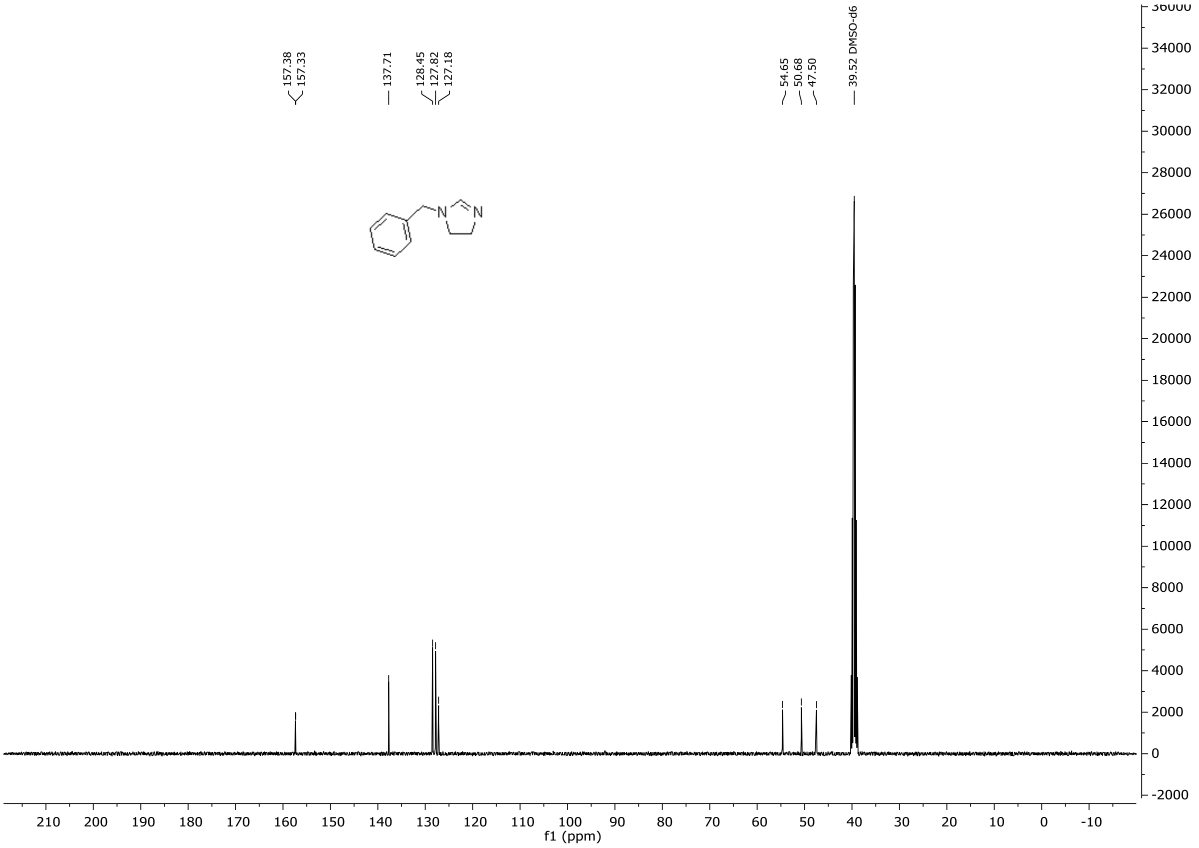

Figure 98. ^{13}C-NMR spectrum of **13** in DMSO-d_6. The CH carbon atom appears as two signals at 157.36 ppm.

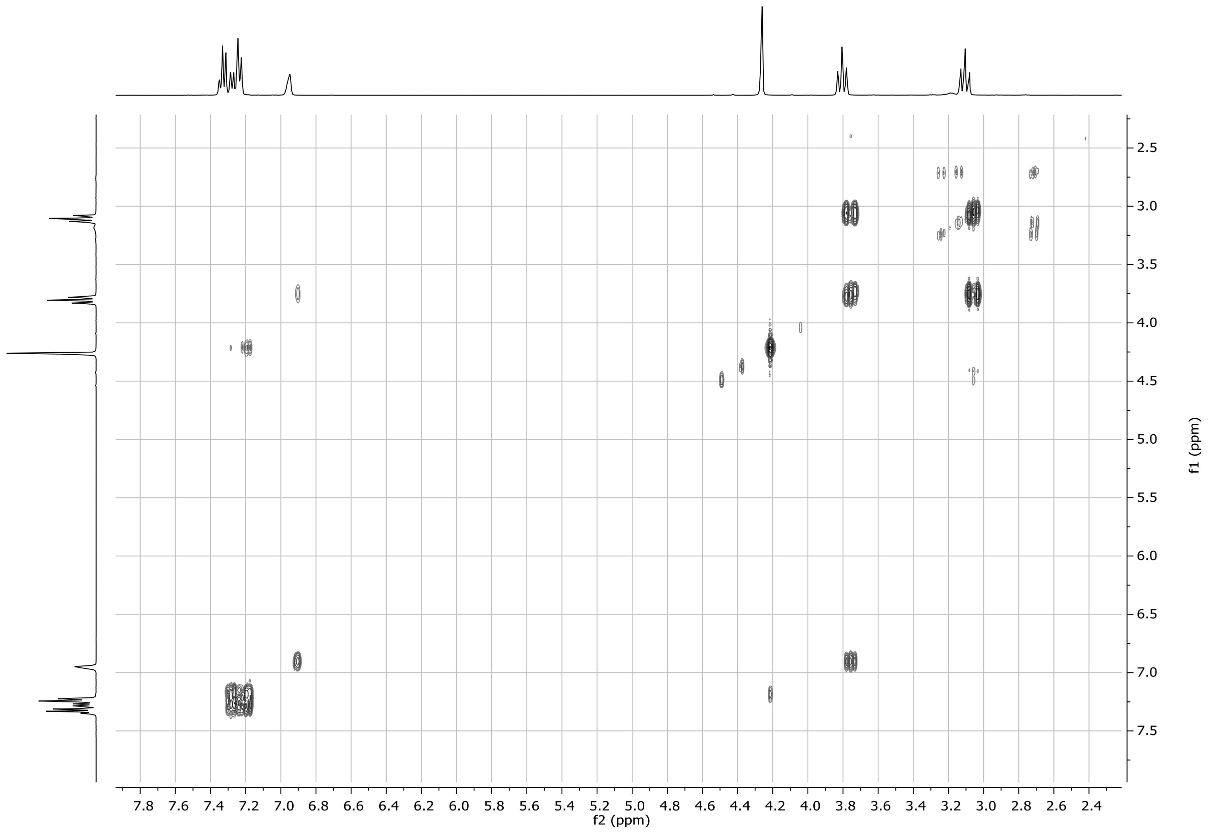

Figure 99. H,H-COSY-NMR spectrum of **13** in $CDCl_3$. The CH signal at 6.95 ppm is coupling with the CH_2 signal at 3.81 ppm, which hence can be assigned in the imidazoline ring: CH_2–CH_2–N–CH.

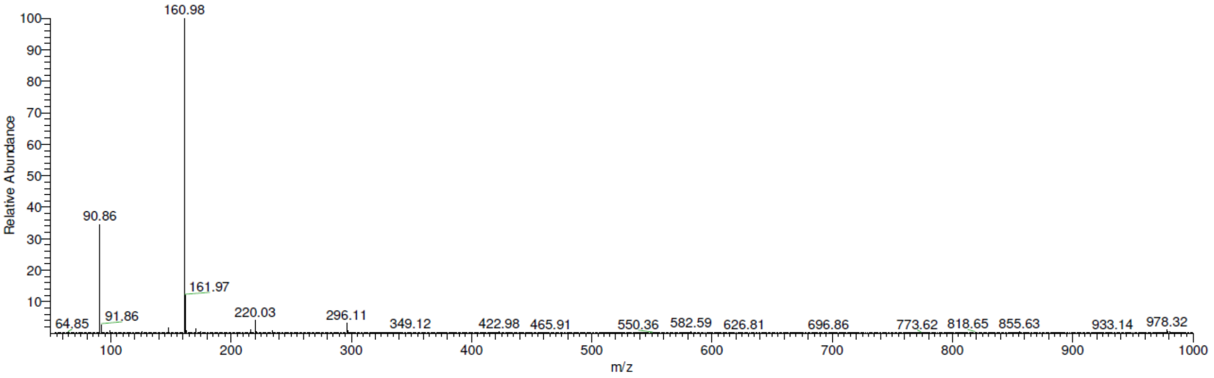

Figure 100. ESI-MS spectrum of **13**.

3,3'-Methylenebis(1-benzyl-2-imidazolinium) dibromide (14)

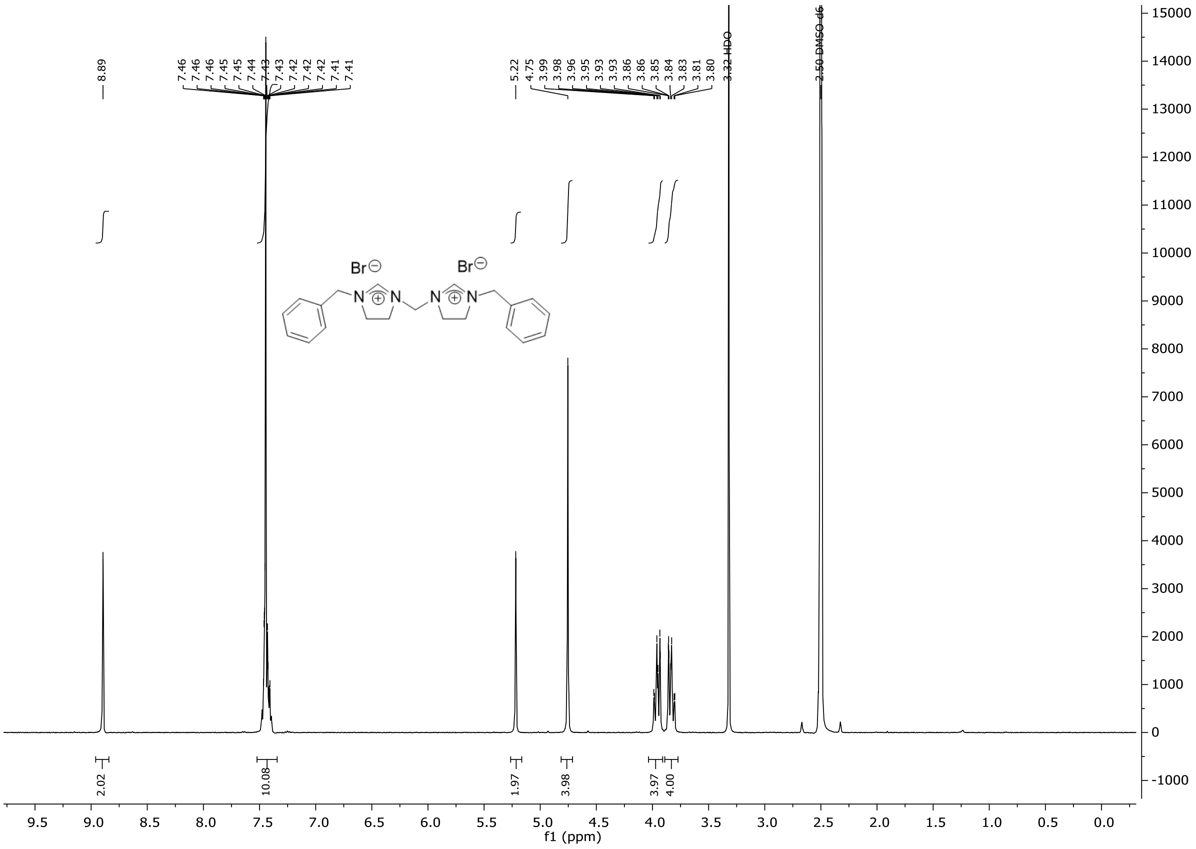

Figure 101. ^{1}H-NMR spectrum of **14** in DMSO-d$_6$.

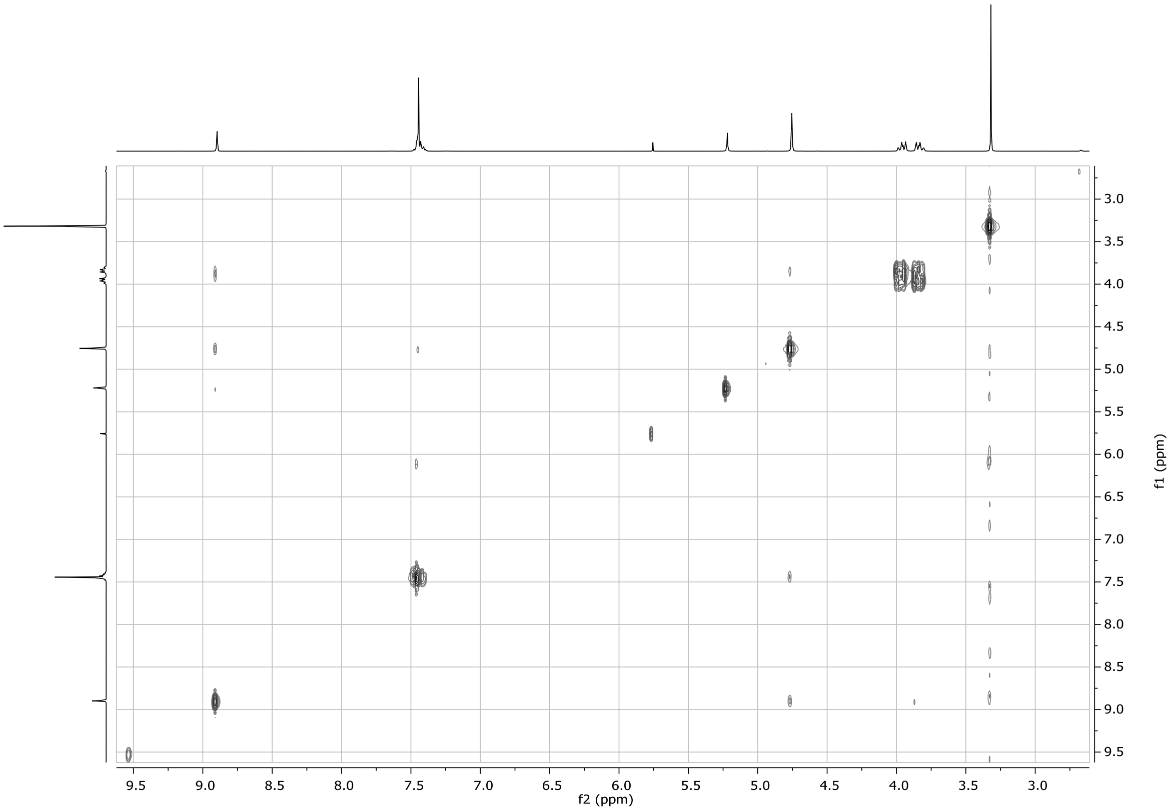

Figure 102. H,H-COSY-NMR spectrum of **14** in DMSO-d$_6$.

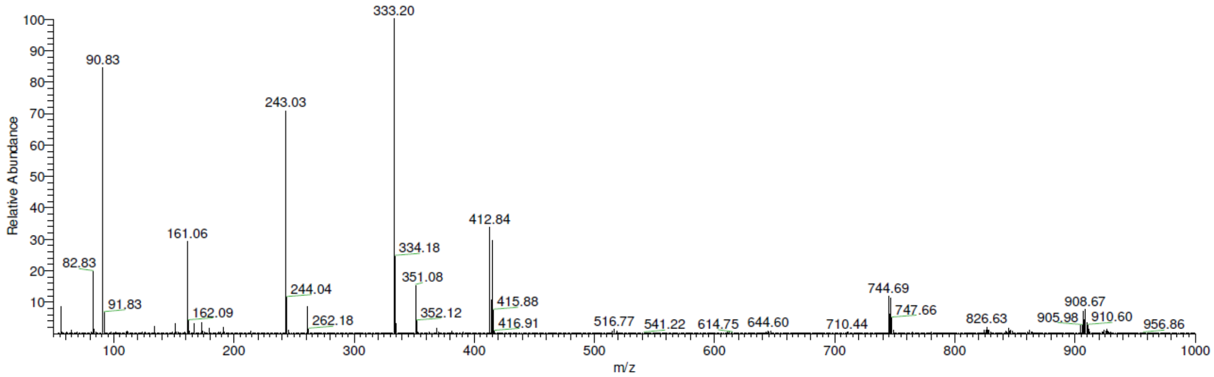

Figure 103. ESI-MS spectrum of **14**.

N^1,N^1,N^2,N^2-Tetrabenzylethane-1,2-diamine (15)

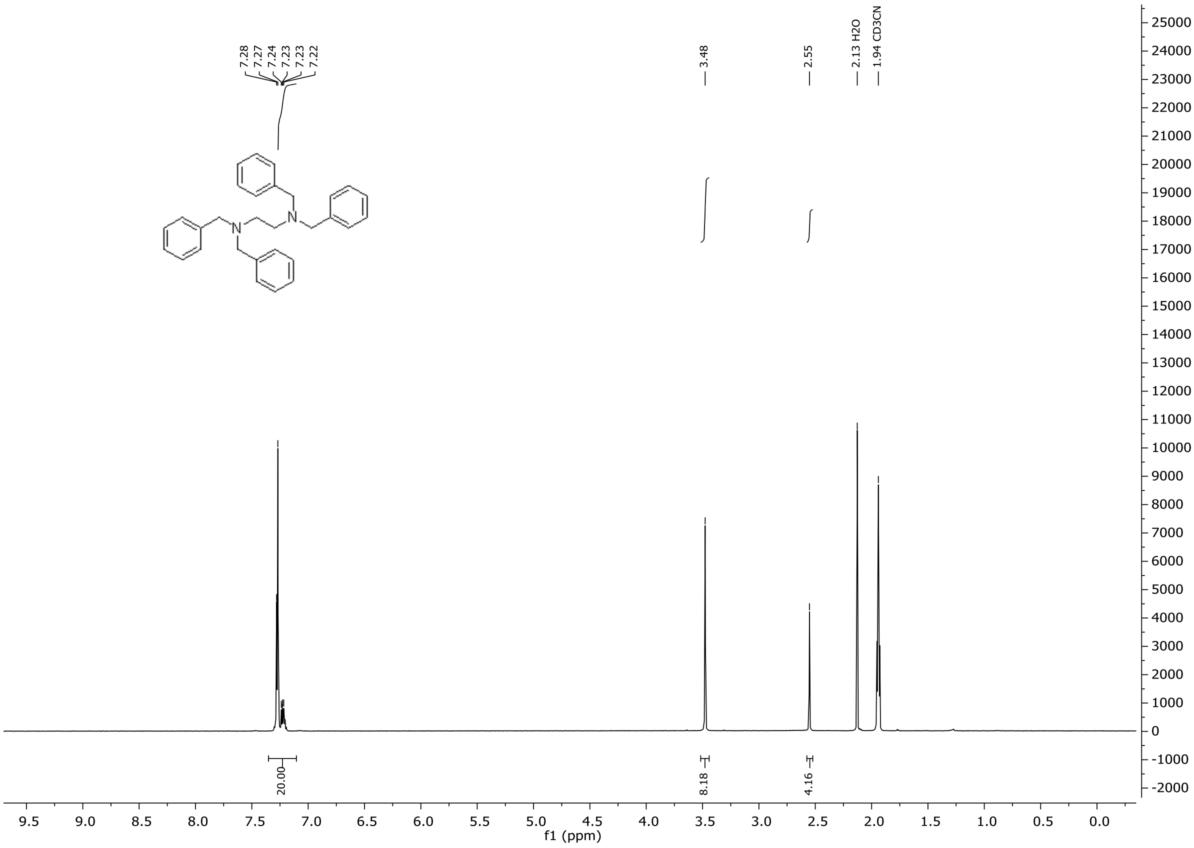

Figure 104. ^{1}H-NMR spectrum of **15** in CD_3CN.

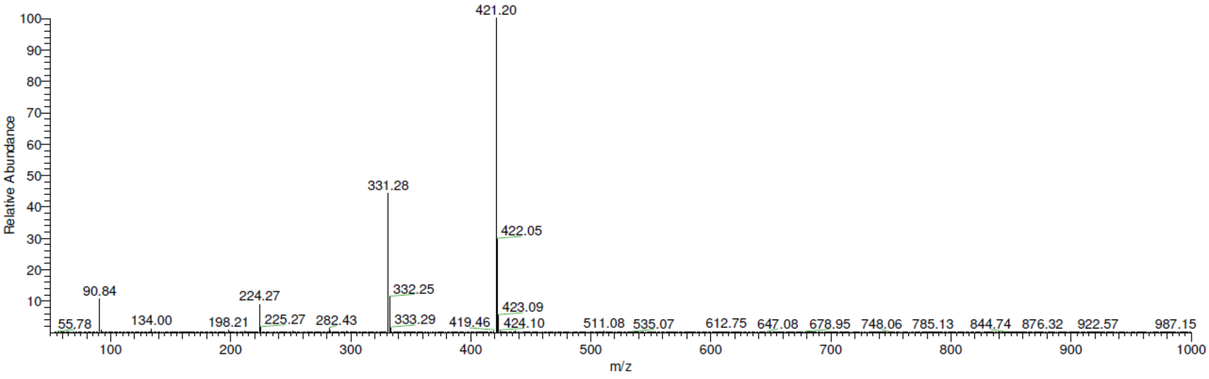

Figure 105. ESI-MS spectrum of **15**.

tert-Butyl (2-aminoethyl)carbamate (16)

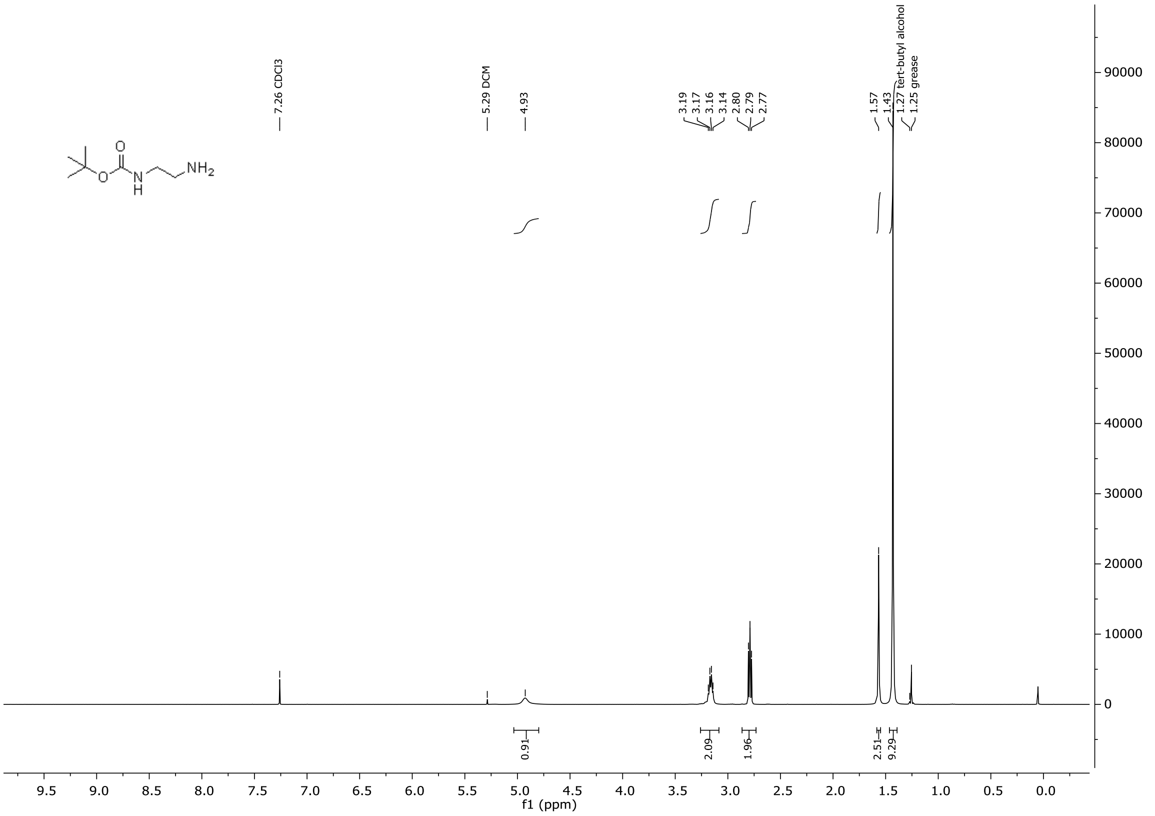

Figure 106. ^{1}H-NMR spectrum of **16** in $CDCl_3$.

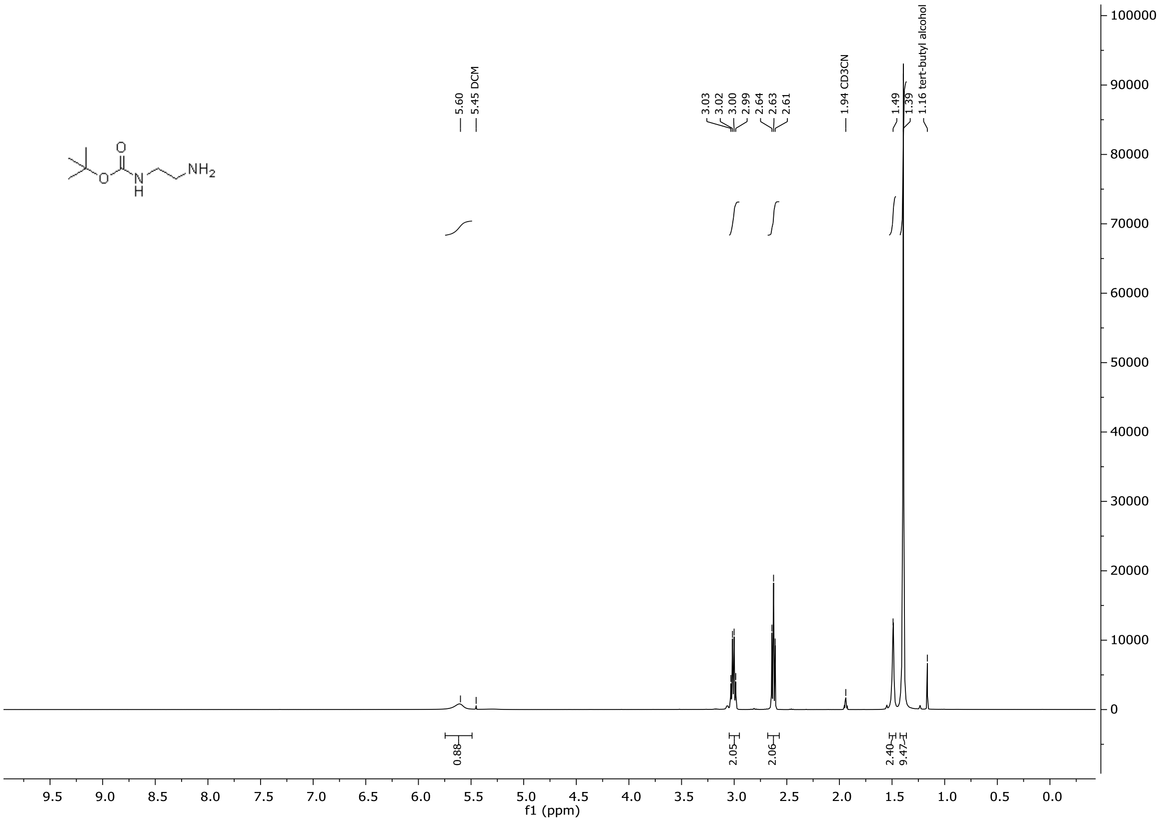

Figure 107. ^{1}H-NMR spectrum of **16** in CD_3CN.

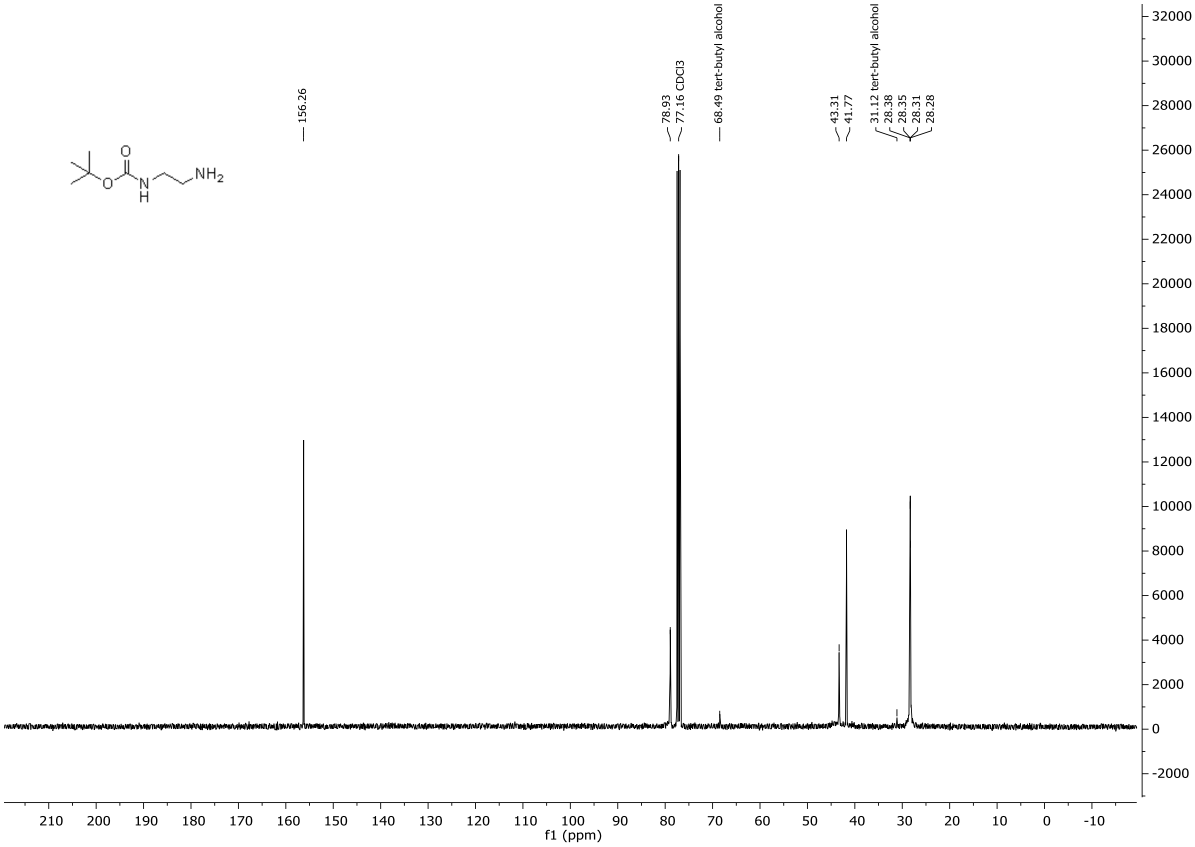

Figure 108. ^{13}C-NMR spectrum of **16** in $CDCl_3$. The carbon signal of $C(CH_3)_3$ appears as quartet at 28.32 ppm.

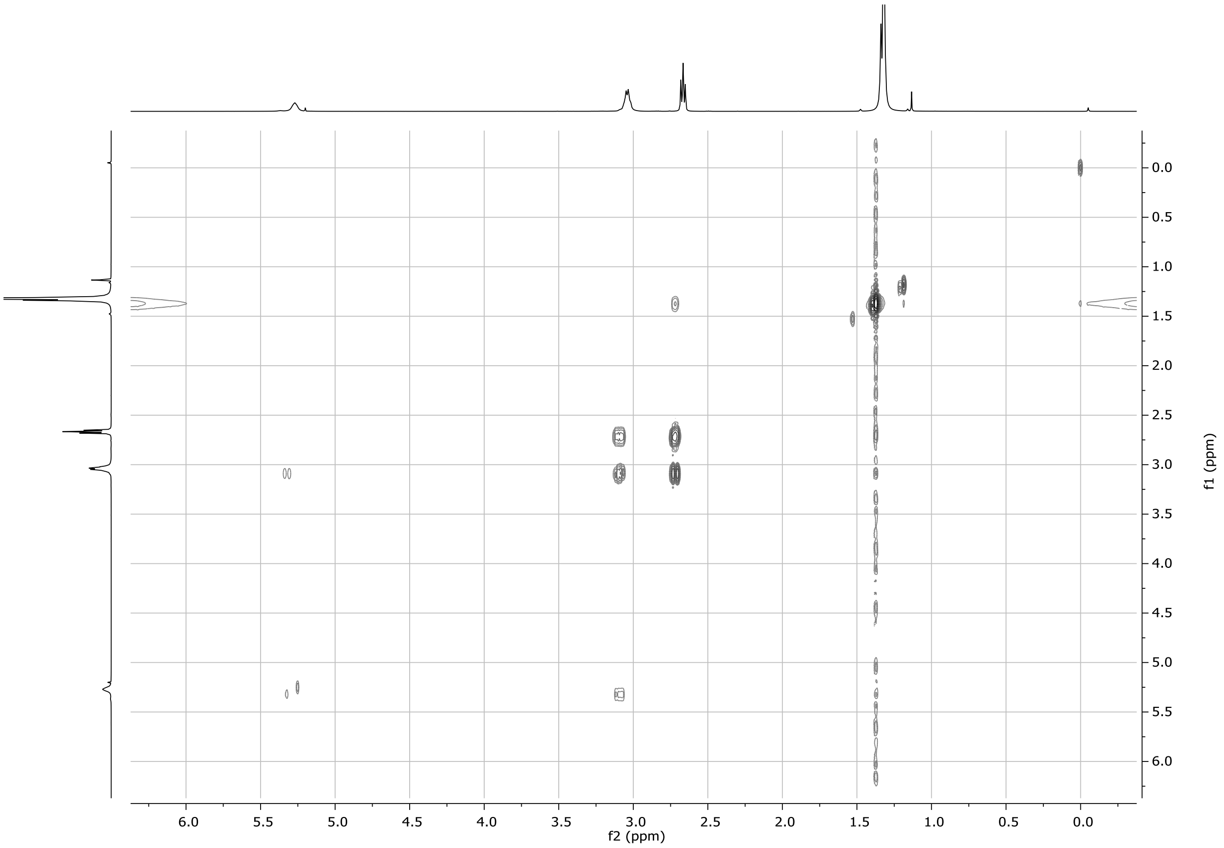

Figure 109. H,H-COSY-NMR spectrum of **16** in $CDCl_3$.

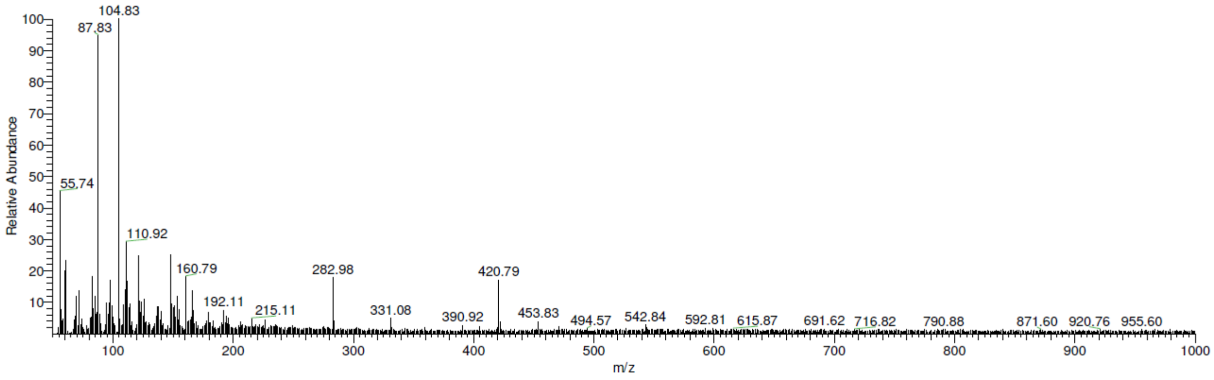

Figure 110. ESI-MS spectrum of **16**.

tert-Butyl 2-imidazoline-1-carboxylate (17) – Method A

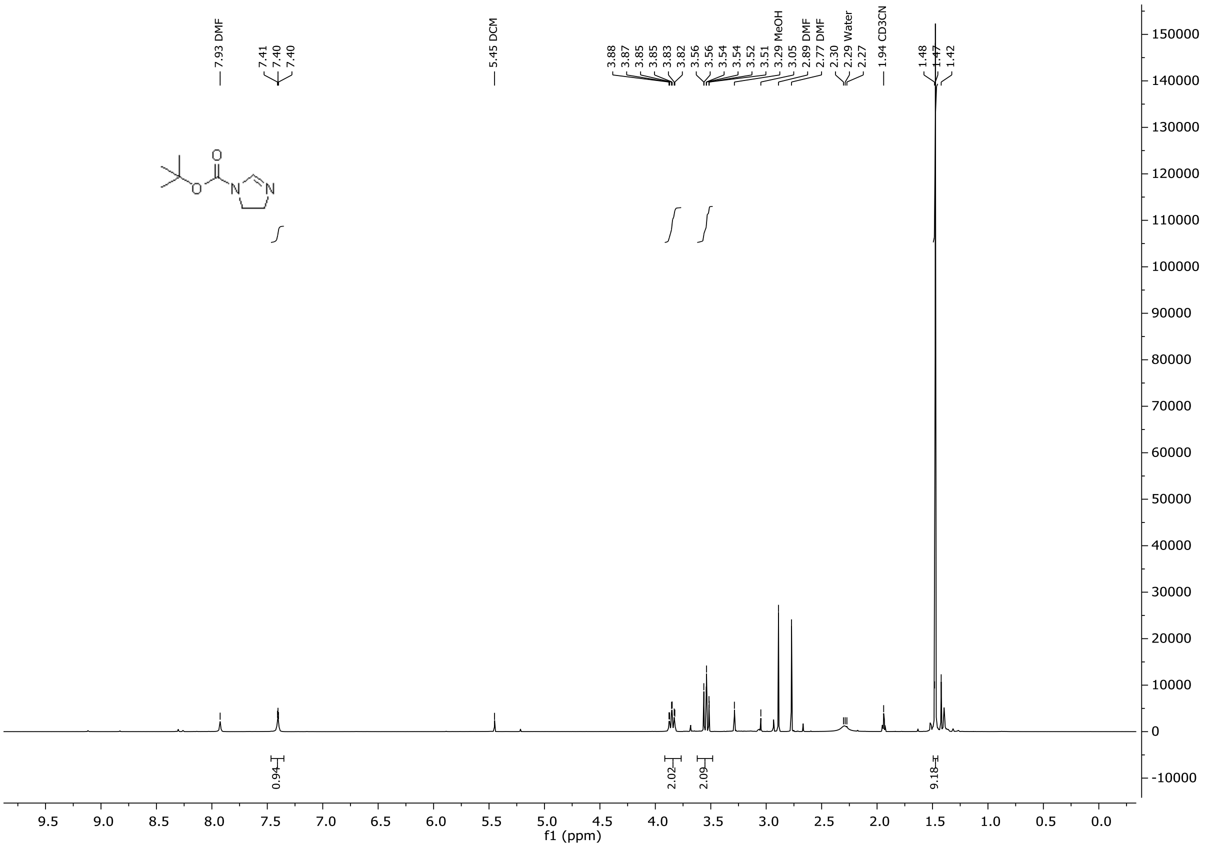

Figure 111. ^{1}H-NMR spectrum of **17** in CD_3CN.

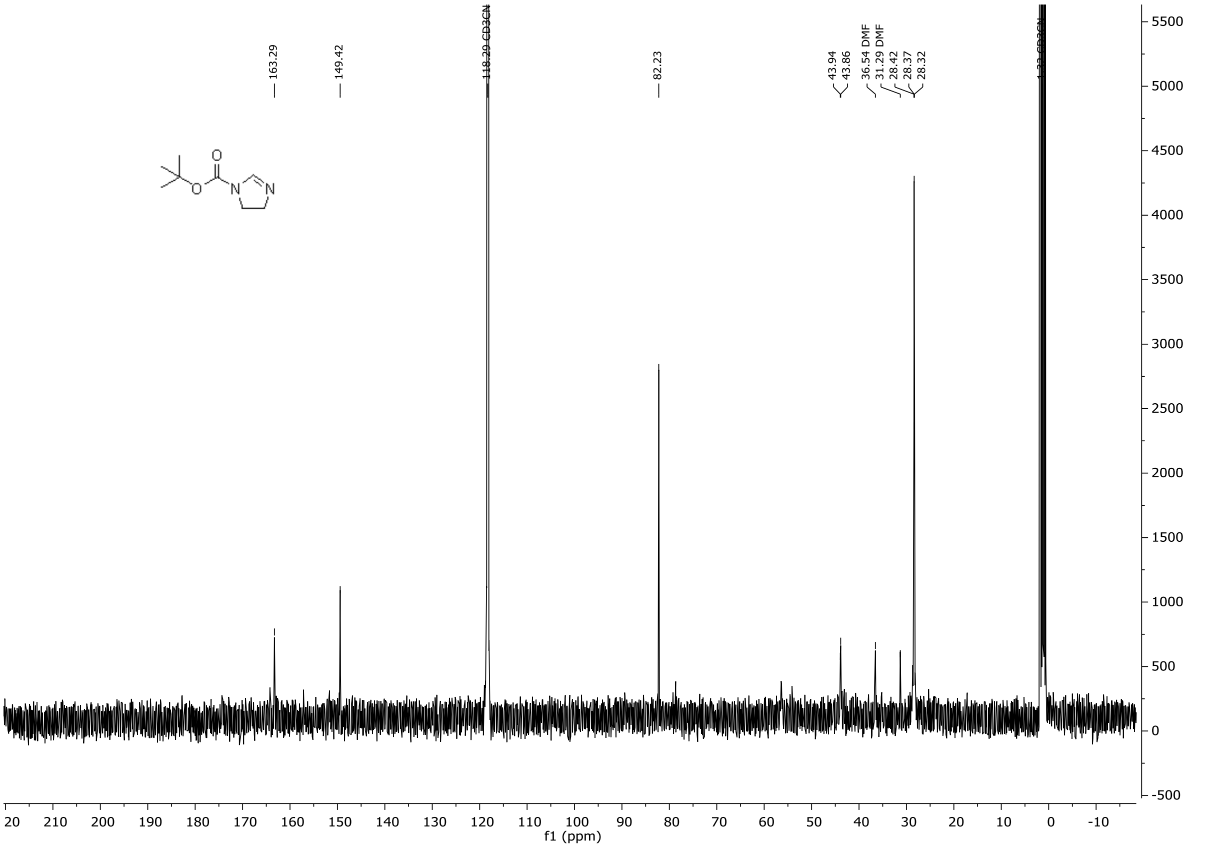

Figure 112. ^{13}C-NMR spectrum of **17** in CD_3CN. The carbon signal of $C(CH_3)_3$ appears as triplet at 28.37 ppm.

***tert*-Butyl 2-imidazoline-1-carboxylate (17) – Method B**

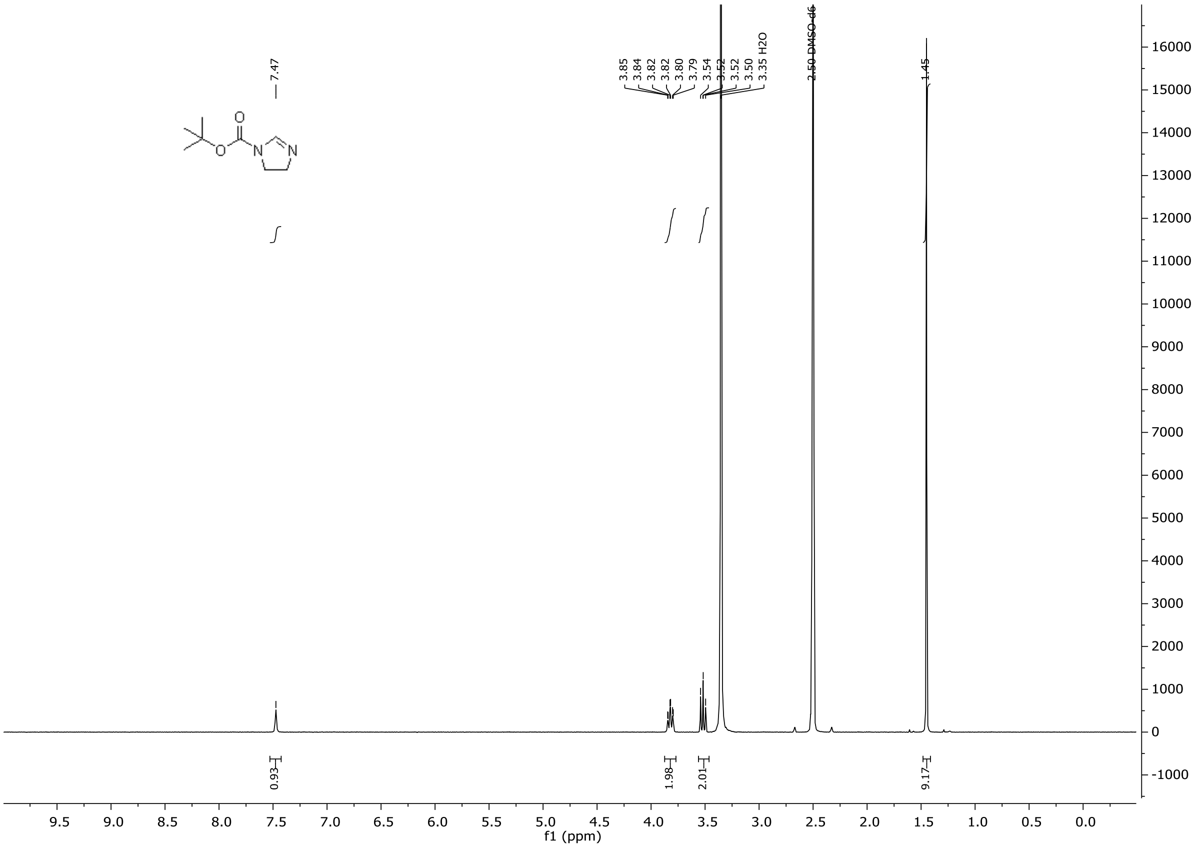

Figure 113. ^{1}H-NMR spectrum of **17** in DMSO-d_6.

5. Crystallographic data

X-ray crystallographic data was collected on a Bruker D8 Venture single crystal x-ray diffractometer, equipped either with a CMOS detector (κ-CMOS) and a TXS rotating anode or a CMOS detector (Bruker Photon-100) and a IMS microsource, both in conjunction with a Helios optic as setup using the APEX3 and APEX4 software package.[60] The measurement used MoK_{α} radiation (λ = 0.71073 Å) and was performed on single crystals coated with perfluorinated ether. The crystals were fixed on top of a micromount sample holder and frozen under a stream of cold nitrogen at 100 K. A matrix scan was used to determine the initial lattice parameters. Reflections were corrected for Lorentz and polarization effects, scan speed, and background using SAINT.[61] Absorption corrections, including odd and even ordered spherical harmonics were performed using SADABS.[62] Space group assignment was based upon systematic absences, E statistics, and successful refinement of the structure. The structure was solved by direct methods (SHELXT) with the aid of successive difference Fourier maps, and was refined against all data using SHELXL-2015 in conjunction with SHELXLE.[63-65] Hydrogen atoms were calculated in ideal positions as follows: Methyl hydrogen atoms were refined as part of rigid rotating groups, with a C–H distance of 0.98 Å and $U_{iso}(H) = 1.5 \cdot U_{eq}(C)$. Other H atoms were placed in calculated positions and refined using a riding model, with methylene, aromatic, and other C–H distances of 0.99 Å, 0.95 Å, and 1.00 Å, respectively and $U_{iso}(H) = 1.2 \cdot U_{eq}(C)$. Non-hydrogen atoms were refined with anisotropic displacement parameters. Full-matrix least-squares refinements were carried out by minimizing $\Sigma w(F_o^2–F_c^2)^2$ with the SHELXL weighting scheme.[65] Neutral atom scattering factors for all atoms and anomalous dispersion corrections for the non-hydrogen atoms were taken from *International Tables for Crystallography.*[66] The unit cell of **PdL9** contains four molecules of acetonitrile which were treated as a diffuse contribution to the overall scattering without specific atom positions using the PLATON/SQUEEZE procedure.[67] The images of the crystal structures were generated with Platon.[68] CCDC 2299374 (**PtL3**), CCDC 2299372 (**PdL3**) and CCDC 2299373 (**PdL9**) contain the supplementary crystallographic data for this paper. This data can be obtained free of charge *via* www.ccdc.cam.ac.uk/data_request/cif, or by emailing data_request@ccdc.cam.ac.uk, or by contacting The Cambridge Crystallographic Data Centre, 12 Union Road, Cambridge CB2 1EZ, UK; fax: +44 1223 336033.

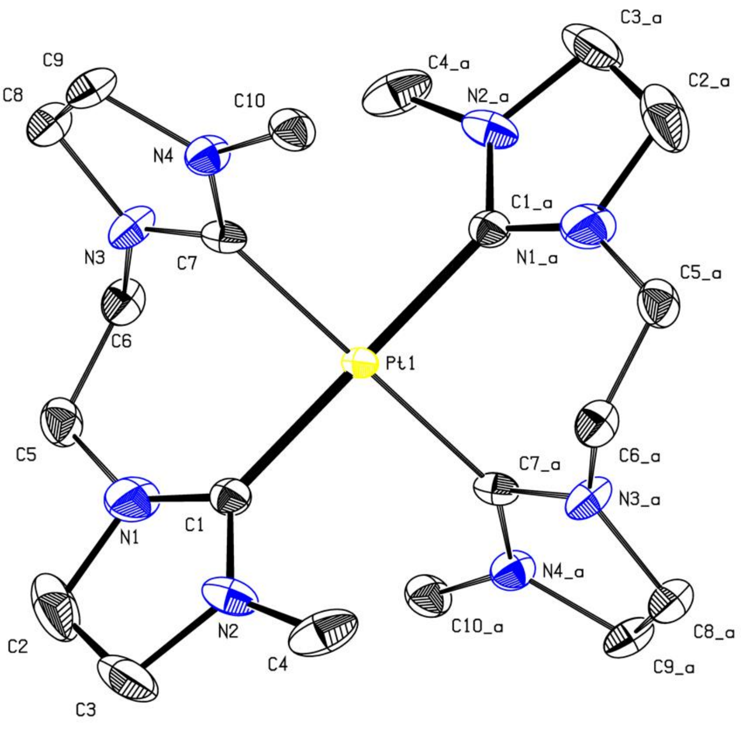

Figure 114: ORTEP-style representation of the cationic fragment of complex **PtL3**. Hydrogen atoms and hexafluorophosphate anions are omitted for clarity. Thermal ellipsoids are shown at a 50% probability level.

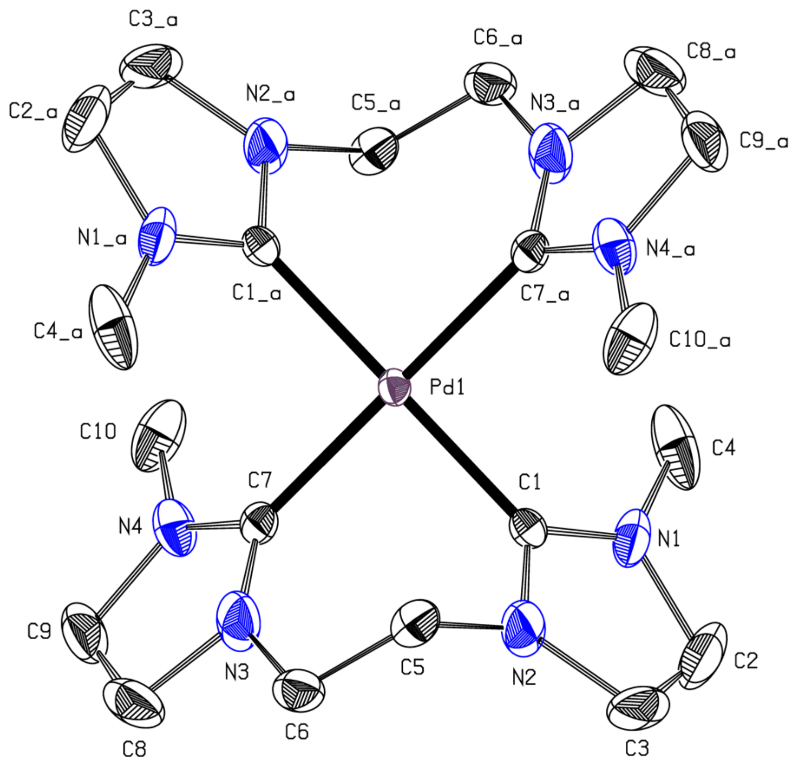

Figure 115: ORTEP-style representation of the cationic fragment of complex **PdL3**. Hydrogen atoms and hexafluorophosphate anions are omitted for clarity. Thermal ellipsoids are shown at a 50% probability level**.**

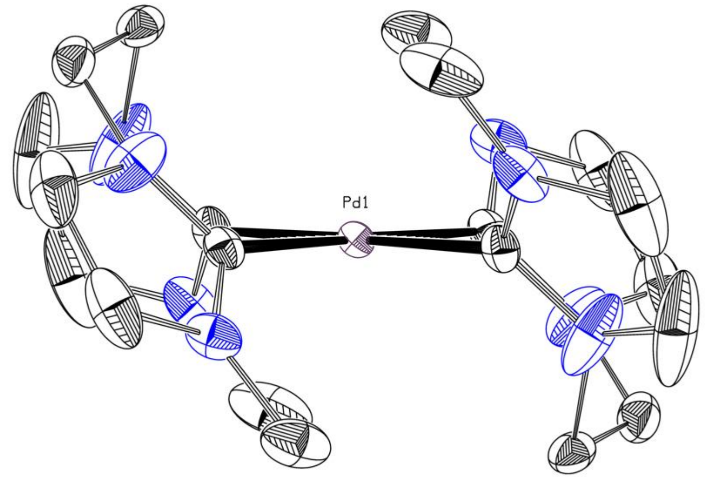

Figure 116: ORTEP-style representation of the cationic fragment of complex **PdL3**. Hydrogen atoms and hexafluorophosphate anions are omitted for clarity. Thermal ellipsoids are shown at a 50% probability level**.** Side Perspective.

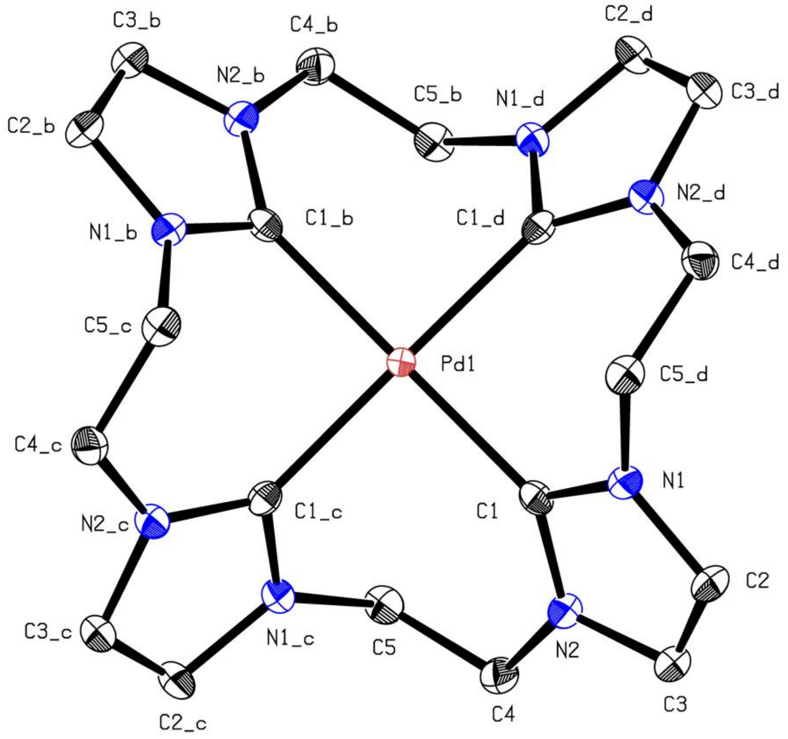

Figure 117: ORTEP-style representation of the cationic fragment of complex **PdL9**. Hydrogen atoms and hexafluorophosphate anions are omitted for clarity. Thermal ellipsoids are shown at a 50% probability level**.**

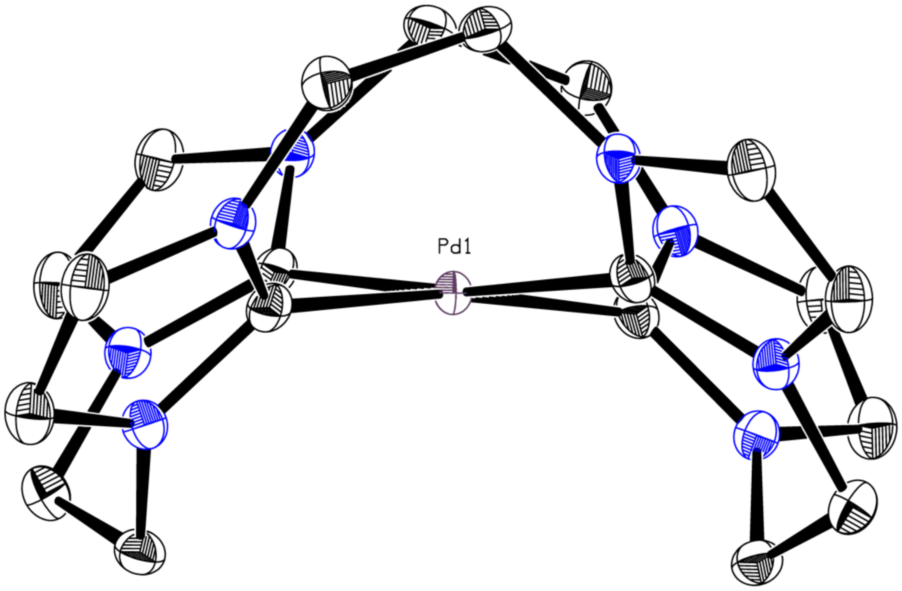

Figure 118: ORTEP-style representation of the cationic fragment of complex **PdL9**. Hydrogen atoms and hexafluorophosphate anions are omitted for clarity. Thermal ellipsoids are shown at a 50% probability level. Side perspective.

6. References SI

[1] P. S. Athey, G. E. Kiefer, *J. Org. Chem.* **2002**, *67*, 4081-4085, DOI: 10.1021/jo016111d.

[2] M. R. Anneser, S. Haslinger, A. Pöthig, M. Cokoja, J.-M. Basset, F. E. Kühn, *Inorg. Chem.* **2015**, *54*, 3797-3804, DOI: 10.1021/ic503043h.

[3] E. Lindner, G. von Au, H. J. Eberle, *Chem. Ber.* **1981**, *114*, 810-813,

[4] Z. Li, E. R. R. Mackie, P. Ramkissoon, J. C. Mather, N. Wiratpruk, T. P. Soares da Costa, P. J. Barnard, *Dalton Trans.* **2020**, *49*, 12820-12834, DOI: 10.1039/D0DT02225J.

[5] W. R. E. Büchele, master’s thesis, Technical University of Munich (Garching, Germany), **2021**.

[6] M. A. Bernd, E. B. Bauer, J. Oberkofler, A. Bauer, R. M. Reich, F. E. Kühn, *Dalton Trans.* **2020**, *49*, 14106-14114, DOI: 10.1039/D0DT02598D.

[7] Y. Chun, N. J. Singh, I. C. Hwang, J. W. Lee, S. U. Yu, K. S. Kim, *Nat. Commun.* **2013**, *4*, 1797, DOI: 10.1038/ncomms2758.

[8] C. Grundmann, A. Kreutzberger, *J. Am. Chem. Soc.* **1955**, *77*, 6559-6562, DOI: 10.1021/ja01629a041.

[9] K. Hofmann, *Imidazole and Its Derivatives,* Interscience Publishers Inc.: New York, **1953**.

[10] T. P. Schlachta, master's thesis, Technical University of Munich (Garching, Germany), **2020**.

[11] B. Alici, E. Çetinkaya, B. Çetinkaya, *Heterocycles* **1997**, *45*, 29-36, DOI: 10.3987/COM-96-7511.

[12] N. Aoyagi, Y. Furusho, Y. Sei, T. Endo, *Tetrahedron* **2013**, *69*, 5476-5480, DOI: 10.1016/j.tet.2013.04.110.

[13] L. Birkofer, H. P. Kühlthau, A. Ritter, *Chem. Ber.* **1960**, *93*, 2810-2813, DOI: 10.1002/cber.19600931208.

[14] H. Suzuki, M. Ohashi, K. Itoh, I. Matsuda, Y. Ishii, *Bull. Chem. Soc. Jpn.* **1975**, *48*, 1922-1924, DOI: 10.1246/bcsj.48.1922.

[15] G. Frachey, C. Crestini, R. Bernini, R. Saladino, E. Mincione, *Heterocycles* **1994**, *38*, 2621-2630, DOI: 10.3987/COM-94-6863.

[16] J. P. James, G. B. Quistad, J. E. Casida, *J. Agric. Food. Chem.* **1995**, *43*, 2530-2535, DOI: 10.1021/jf00057a039.

[17] W. H. Newsome, L. G. Panopio, *J. Agric. Food. Chem.* **1978**, *26*, 638-640, DOI: 10.1021/jf60217a036.

[18] W. D. Marshall, *J. Agric. Food. Chem.* **1979**, *27*, 295-299, DOI: 10.1021/jf60222a041.

[19] C. J. Grundmann, A. Kreutzberger, Patent US2841585A, **1958**.

[20] C. Grundmann, *Angew. Chem. Int. Ed.* **1963**, *2*, 309-323, DOI: 10.1002/anie.196303091.

[21] R. N. Butler, K. J. Fitzgerald, *J. Chem. Soc., Perkin Trans. 1* **1989**, 155-157, DOI: 10.1039/P19890000155.

[22] Y. Ito, Y. Inubushi, M. Zenbayashi, S. Tomita, T. Saegusa, *J. Am. Chem. Soc.* **1973**, *95*, 4447-4448, DOI: 10.1021/ja00794a065.

[23] B. S. Hickman, M. Mascal, J. J. Titman, I. G. Wood, *J. Am. Chem. Soc.* **1999**, *121*, 11486-11490, DOI: 10.1021/ja991401l.

[24] J. F. Schlagintweit, L. Nguyen, F. Dyckhoff, F. Kaiser, R. M. Reich, F. E. Kühn, *Dalton Trans.* **2019**, *48*, 14820-14828, DOI: 10.1039/C9DT03430G.

[25] E. Díez-Barra, A. de la Hoz, A. Sánchez-Migallón, J. Tejeda, *Heterocycles* **1992**, *34*, 1365-1373, DOI: 10.3987/COM-92-6024.

[26] M. M. Watts, *Journal of the American Oil Chemists' Society* **1990**, *67*, 993-995, DOI: 10.1007/BF02541864.

[27] B. G. Harnsberger, J. L. Riebsomer, *J. Heterocycl. Chem.* **1964**, *1*, 188-192, DOI: 10.1002/jhet.5570010408.

[28] C.-Y. Liao, K.-T. Chan, C.-Y. Tu, Y.-W. Chang, C.-H. Hu, H. M. Lee, *Chem. Eur. J.* **2009**, *15*, 405-417, DOI: 10.1002/chem.200801296.

[29] M. M. Joullie, G. M. J. Slusarczuk, A. S. Dey, P. B. Venuto, R. H. Yocum, *J. Org. Chem.* **1967**, *32*, 4103-4105, DOI: 10.1021/jo01287a100.

[30] M. E. Kuehne, F. Xu, *J. Org. Chem.* **1993**, *58*, 7490-7497, DOI: 10.1021/jo00078a030.

[31] R. L. Parsons, J. D. Berk, M. E. Kuehne, *J. Org. Chem.* **1993**, *58*, 7482-7489, DOI: 10.1021/jo00078a029.

[32] H. Zhou, X. Liao, J. M. Cook, *Org. Lett.* **2004**, *6*, 249-252, DOI: 10.1021/ol0362212.

[33] E. Riva, D. Comi, S. Borrelli, F. Colombo, B. Danieli, J. Borlak, L. Evensen, J. B. Lorens, G. Fontana, O. M. Gia, L. D. Via, D. Passarella, *Bioorganic & Medicinal Chemistry* **2010**, *18*, 8660-8668, DOI: 10.1016/j.bmc.2010.09.069.

[34] P. O. Nikiforov, S. Surade, M. Blaszczyk, V. Delorme, P. Brodin, A. R. Baulard, T. L. Blundell, C. Abell, *Organic & Biomolecular Chemistry* **2016**, *14*, 2318-2326, DOI: 10.1039/C5OB02630J.

[35] C. Marshall, M. F. Ward, J. M. S. Skakle, *Synthesis* **2006**, *2006*, 1040-1044, DOI: 10.1055/s-2006-926361.

[36] M. Bessel, F. Rominger, B. F. Straub, *Synthesis* **2010**, *2010*, 1459-1466, DOI: 10.1055/s-0029-1218702.

[37] A. Frost, A. Carlson, *The Journal of Organic Chemistry* **1959**, *24*, 1581-1582, DOI: 10.1021/jo01092a614.

[38] D. E. Goldberg, K. C. Patel, *Journal of Inorganic and Nuclear Chemistry* **1972**, *34*, 3583-3584, DOI: 10.1016/0022-1902(72)80260-4.

[39] L. W. Jenneskens, J. Mahy, E. M. M. de Brabander-van den Berg, I. van der Hoef, J. Lugtenburg, *Recueil des Travaux Chimiques des Pays-Bas* **1995**, *114*, 97-102, DOI: 10.1002/recl.19951140305.

[40] B. Çetinkaya, E. Çetinkaya, P. B. Hitchcock, M. F. Lappert, I. Özdemir, *Journal of the Chemical Society, Dalton Transactions* **1997**, 1359-1362, DOI: 10.1039/A608360I.

[41] R. C. F. Jones, J. R. Nichols, *Tetrahedron* **2013**, *69*, 4114-4119, DOI: 10.1016/j.tet.2013.03.040.

[42] R. C. F. Jones, K. J. Howard, J. R. Nichols, J. S. Snaith, *Journal of the Chemical Society, Perkin Transactions 1* **1998**, 2061-2072, DOI: 10.1039/A802048E.

[43] D. Muller, I. Zeltser, G. Bitan, C. Gilon, *The Journal of Organic Chemistry* **1997**, *62*, 411-416, DOI: 10.1021/jo961580e.

[44] A. Eisenführ, P. S. Arora, G. Sengle, L. R. Takaoka, J. S. Nowick, M. Famulok, *Bioorganic & Medicinal Chemistry* **2003**, *11*, 235-249, DOI: 10.1016/S0968-0896(02)00311-5.

[45] R. Kuwano, N. Kameyama, R. Ikeda, *J. Am. Chem. Soc.* **2011**, *133*, 7312-7315, DOI: 10.1021/ja201543h.

[46] X. Chen, G. Gong, X. Chen, R. Song, M. Duan, R. Qiao, Y. Jiao, J. Qi, Y. Chen, Y. Zhu, *Chem. Pharm. Bull.* **2019**, *67*, 1116-1122, DOI: 10.1248/cpb.c19-00425.

[47] S. Lupsor, F. Aonofriesei, M. Iovu, *Med. Chem. Res.* **2012**, *21*, 3035-3042, DOI: 10.1007/s00044-011-9839-2.

[48] S. Lupsor, V. Uivarosi, M. Iovu, *Rev. Chim.* **2010**, *61*, 333-335, DOI: n.a.

[49] G. R. Fulmer, A. J. M. Miller, N. H. Sherden, H. E. Gottlieb, A. Nudelman, B. M. Stoltz, J. E. Bercaw, K. I. Goldberg, *Organometallics* **2010**, *29*, 2176-2179, DOI: 10.1021/om100106e.

[50] H. M. Bass, S. A. Cramer, A. S. McCullough, K. J. Bernstein, C. R. Murdock, D. M. Jenkins, *Organometallics* **2013**, *32*, 2160-2167, DOI: 10.1021/om400043z.

[51] Z. Wang, S. M. Richter, M. J. Rozema, A. Schellinger, K. Smith, J. G. Napolitano, *Org. Process Res. Dev.* **2017**, *21*, 1501-1508, DOI: 10.1021/acs.oprd.7b00158.

[52] A. Raba, M. R. Anneser, D. Jantke, M. Cokoja, W. A. Herrmann, F. E. Kühn, *Tetrahedron Lett.* **2013**, *54*, 3384-3387, DOI: 10.1016/j.tetlet.2013.04.060.

[53] H. Kroth, N. Sreenivasachary, A. Hamel, P. Benderitter, Y. Varisco, V. Giriens, P. Paganetti, W. Froestl, A. Pfeifer, A. Muhs, *Bioorg. Med. Chem. Lett.* **2016**, *26*, 3330-3335, DOI: 10.1016/j.bmcl.2016.05.040.

[54] H. G. Selnick, J. F. Hess, C. Tang, K. Liu, J. B. Schachter, J. E. Ballard, J. Marcus, D. J. Klein, X. Wang, M. Pearson, M. J. Savage, R. Kaul, T.-S. Li, D. J. Vocadlo, Y. Zhou, Y. Zhu, C. Mu, Y. Wang, Z. Wei, C. Bai, J. L. Duffy, E. J. McEachern, *J. Med. Chem.* **2019**, *62*, 10062-10097, DOI: 10.1021/acs.jmedchem.9b01090.

[55] B. Almarzoqi, A. V. George, N. S. Isaacs, *Tetrahedron* **1986**, *42*, 601-607, DOI: 10.1016/S0040-4020(01)87459-7.

[56] K. Yan, B. Li, B. Wang, *Adv. Synth. Catal.* **2018**, *360*, 2272-2279, DOI: 10.1002/adsc.201800149.

[57] F. Guo, X. Wang, H.-y. Guan, H.-b. Yu, L. Li, S.-s. Chen, A. Famulari, J. Martí-Rujas, *Cryst. Growth Des.* **2015**, *15*, 2842-2852, DOI: 10.1021/acs.cgd.5b00272.

[58] I. Nicoletti, G. Migliorati, M. C. Pagliacci, F. Grignani, C. Riccardi, *Journal of Immunological Methods* **1991**, *139*, 271-279, DOI: 10.1016/0022-1759(91)90198-O.

[59] F. K.-M. Chan, K. Moriwaki, M. J. De Rosa, in *Immune Homeostasis: Methods and Protocols* (Eds.: A. L. Snow, M. J. Lenardo), Humana Press: Totowa, NJ, **2013**, pp. 65-70, DOI: 10.1007/978-1-62703-290-2_7.

[60] *APEX suite of crystallographic software*, APEX3, Version 2019-11.0, Bruker AXS Inc.: Madison, Wisconsin, USA, **2019**.

[61] *SAINT*, Version 8.38A, Bruker AXS Inc.: Madison, Wisconsin, USA, **2017**.

[62] *SADABS*, Version 2016/2, Bruker AXS Inc.: Madison, Wisconsin, USA, **2016**.

[63] G. M. Sheldrick, *Acta Cryst. C* **2015**, *71*, 3-8, DOI: 10.1107/S2053229614024218.

[64] G. M. Sheldrick, *Acta Cryst. A* **2015**, *71*, 3-8, DOI: 10.1107/S2053273314026370.

[65] C. B. Hübschle, G. M. Sheldrick, B. Dittrich, *J. Appl. Crystallogr.* **2011**, *44*, 1281-1284, DOI: 10.1107/S0021889811043202.

[66] A. J. Wilson, *International Tables for Crystallography,* Kluwer Academic Publishers: Dordrecht, The Nederlands, **1992**.

[67] A. Spek, *Acta Cryst. C* **2015**, *71*, 9-18, DOI: 10.1107/S2053229614024929.

[68] A. Spek, *Acta Cryst. D* **2009**, *65*, 148-155, DOI: 10.1107/S090744490804362X.
